# Epidemiological and demographic trends and projections in global health from 1970 to 2050: a descriptive analysis from the third *Lancet* Commission on Investing in Health, Global Health 2050

**DOI:** 10.1016/S0140-6736(25)00902-X

**Published:** 2025-09-30

**Authors:** Angela Y Chang, Sarah Bolongaita, Bochen Cao, Marcia C Castro, Omar Karlsson, Wenhui Mao, Ole F Norheim, Osondu Ogbuoji, Dean T Jamison

**Affiliations:** a Danish Institute for Advanced Study, University of Southern Denmark, Odense, Denmark; b Danish Centre for Health Economics, University of Southern Denmark, Odense, Denmark; c Bergen Centre for Ethics and Priority Setting (BCEPS), Department of Global Public Health and Primary Care, University of Bergen, Bergen, Norway; d Department of Data and Analytics, World Health Organization, Geneva, Switzerland; e Department of Global Health and Population, Harvard T H Chan School of Public Health, Boston, MA, USA; f Duke University Population Research Institute, Duke University, Durham, NC, USA; g Duke Global Health Innovation Center and Innovations in Healthcare, Duke University, Durham, NC, USA; h Centre for Policy Impact in Global Health, Duke University, Durham, NC, USA; i Department of Population Health, Duke School of Medicine, Duke University, Durham, NC, USA; j Centre for Economic Demography, School of Economics and Management, Lund University, Lund, Sweden; k Institute for Global Health Sciences, University of California, San Francisco, CA, USA

## Abstract

**Background:**

Systematic analyses of global health trends can provide an accurate narrative of progress and challenges. We analysed the impact of changing age-specific mortality (epidemiology) and age structure (demography) on crude death rates (CDRs) and causes of death with large or rising mortality to inform the third *Lancet* Commission on Investing in Health.

**Methods:**

Data from the World Population Prospects 2024 and Global Health Estimates 2021 were used to assess epidemiological and demographic trends, including CDR (defined as the total number of deaths divided by the total mid-year population, reported per 1000 population), all-cause age-specific mortality rates for 1970–2050, and selected cause-specific mortality rates from 2000–19. We excluded data for 2020–23 to avoid effects of the COVID-19 pandemic. For estimating decadal changes in cause-specific mortality rates, we combined the estimates into the following age groups: 0–14, 15–49, 50–69, and 70 years and older.

**Findings:**

Mortality rates declined substantially across age groups in most regions, with rapid improvements observed in recent decades. Between the 2000s (ie, 2000–10) and 2010s (ie, 2010–19), the mortality decline accelerated in China, central and eastern Europe, India, and Latin America and the Caribbean in ages 0–14 years and 15–49 years, but decelerated in the north Atlantic, the USA, and western Pacific and southeast Asia. For ages 50–69 years, mortality decline decelerated in all regions except sub-Saharan Africa. The USA experienced not only deceleration but increase in mortality rates in those aged 15–49 years and 50–69 years. Globally, the lowest CDR was reported in 2019. In the past, CDR has declined primarily because of decreasing age-specific mortality rates. Future trends suggest that changing population age structure will drive a large increase in CDR. Age-specific mortality rates from major diseases declined once population changes were accounted for. The exception was diabetes, with accelerating increase in age-specific death rates in all regions, with especially high rates in central and eastern Europe and India.

**Interpretation:**

There is reason for optimism regarding global health progress, but disparities and emerging challenges persist. Falling age-specific mortality rates show progress; however, rapid ageing brings new challenges. Slowing mortality declines in some regions require enhanced efforts. Rising mortality among middle-aged Americans emphasises that continuous improvements require concerted efforts. Key recommendations include prioritising interventions to address specific health challenges and adapting health-care systems to demographic transitions.

**Funding:**

The Norwegian Agency for Development Cooperation and the Bill & Melinda Gates Foundation.

## Introduction

Between 1970 and 2019, global age-specific mortality rates reached the lowest in history. Life expectancy at birth globally increased from 56 years in 1970 to 73 years in 2019.^1^ Advances in medical technology and public health initiatives continue to improve health.^2^ However, recent narratives in global health have been less optimistic. The COVID-19 pandemic, climate change, funding cuts, and rising geopolitical tensions have introduced uncertainty and dominate the agenda. As pressing as these issues are, another more predictable challenge is emerging. Because of improvements in survival rates and falling fertility rates, demographic changes are occurring, with the median age increasing from 20 years in 1970 to 29 years in 2019, and projected to reach 36 years by 2050. The global median age of death increased from 69 years in 1970 to 76 years in 2019, and is projected to reach 82 years by 2050, straining health budgets and increasing the burden from chronic conditions.^1^, ^3^, ^4^

It is therefore timely that we systematically analysed recent global and regional epidemiological and demographic trends to appropriately evaluate the past, plan for the future, and shape appropriate and constructive narratives about global health. Recent literature on these topics has focused mostly on the impact of the COVID-19 pandemic on mortality, on younger age groups, or on infectious diseases.^3^, ^5^ Lessons from countries and regions experiencing improvements in health outcomes can guide policy makers in developing appropriate policies and interventions. Likewise, the same analyses can highlight countries and regions that did not observe expected health improvements.

**Research in context**

**Evidence before this study**

We searched PubMed for articles that analysed global and regional trends in epidemiology and demography from Jan 1, 2000, to July 27, 2024. Our search criteria comprised the following terms: (“all cause” OR “cause specific” or “age specific”) AND (“mortality rate” OR “death rate”) AND (global OR worldwide) AND (trend*). We had no language restrictions. The Global Burden of Disease (GBD) study has published several papers that cover different aspects, including trends in demography, all-cause mortality rates, and cause-specific mortality rates. The publication in 2024 by the GBD Demographics Collaborators focused mostly on the impact of the COVID-19 pandemic on mortality. A study in 2022 estimated the causes and trends of mortality in children younger than 5 years between 2000 and 2019 and focused primarily on infectious and neonatal diseases. Additionally, a study from 2020 decomposed and attributed changes in the number of deaths between 1990 and 2017 to population growth, population ageing, and mortality change using the GBD 2017 dataset.

**Added value of this study**

This study summarises the epidemiological and demographic analyses conducted to inform the third *Lancet* Commission on Investing in Health, titled Global Health 2050. We report that for most age groups, the fastest declines in all-cause mortality rates were observed in the last two decades (2000–10 and 2010–19), and for some age groups even in 2010–19. We observed that the lowest crude death rate (CDR) for the world in human history was reported in 2019. Based on UN projections to the year 2100, all regions, except central Asia and sub-Saharan Africa, have already experienced their lowest CDR and are now experiencing rising CDRs. Beyond infectious, neonatal, and maternal health conditions, we expanded our focus to 15 priority conditions, seven of which are non-communicable diseases. We identified that atherosclerotic cardiovascular diseases, diabetes, and strongly tobacco-associated non-communicable diseases have had increased cause-specific death rates since 2000. Compared to the existing literature, there are two major differences. First, our study has focused attention to key questions—for example, what are the levels and trends of the 15 priority conditions? Is it true that mortality decline has slowed down since the Millenium Development Goals? Because we are not required to report on all datapoints, we are able to focus on some key messages. The second difference is with regard to how we treat population age structure. Existing studies, such as the GBD, report most estimates as age-standardised rates, which allows them to make comparisons over time and across regions. Our study reports age-specific rates to compare levels and trends within each age group. We put more focus on the relationship between demographic change and health outcomes—for example in our decomposition of the CDR and cause-specific deaths into parts that are due to demographic change versus actual cause-specific and age-specific change. We observed that the UN reports the lowest global CDR occurring in 2019 right before the pandemic, which is an important turning point.

**Implications of all the available evidence**

The world and several regions have experienced substantial reductions in age-specific and cause-specific mortality rates, providing reasons for optimism about the future of global health. Increased CDRs and median age of death will lead to greater demands on health financing and health-care provision. We recommend that governments focus on the prevention and treatment of 15 priority conditions, which will lead to large reductions in financing and health-care burdens, as well as in health inequality across regions.

In this study, we summarise the epidemiological and demographic analyses conducted to inform the third *Lancet* Commission on Investing in Health (CIH), titled “Global Health 2050”. We aimed to analyse the changes in all-cause mortality rates, in both age-specific and crude death rates (CDRs), and decomposed the trends of cause-specific mortality rates for selected priority conditions that contributed to the majority of the life expectancy gap between each region and the best performing region.

## Methods

### Regional definition

**Figure 1:**
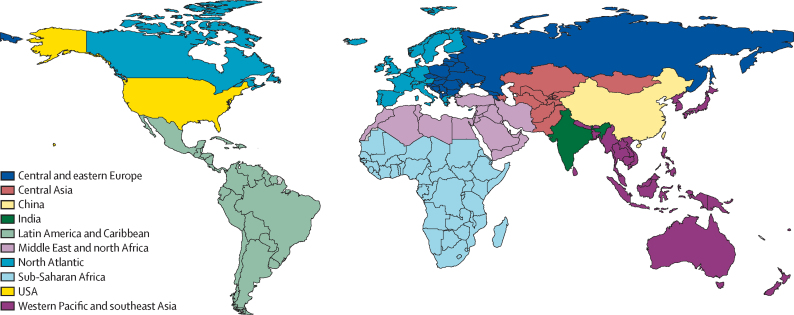
The third *Lancet* Commission on Investing in Health regions The list of locations by region is summarised in the appendix (pp 3–4).

We report results for the world and ten CIH regions: central and eastern Europe, central Asia, China, India, Latin America and Caribbean, Middle East and north Africa, north Atlantic, sub-Saharan Africa, USA, and western Pacific and southeast Asia (figure 1). China, India, and the USA were set as their own regions because of their large population and distinct health patterns compared with their geographical regions.

Countries were included in a CIH region if they were UN Member States with populations of at least 300 000 in 2024. For the CIH world region, if an input dataset contained a world region, those values were used for the CIH world region; if a dataset did not contain a world region, values for the CIH world region were calculated from all locations with available data, regardless of UN Member State status or population size. Mapping of countries to CIH regions is listed in the appendix (pp 3–4).

### Data

All-cause age-specific mortality rates, CDRs, and demographic data came from the UN World Population Prospects 2024. For most analyses, we used data from 1970 to 2019 by age, sex, and country. For CDRs, we included the median World Population Prospects (WPP) projection to 2050. Cause-specific mortality data came from WHO's Global Health Estimates (GHE) 2021, which provided age, sex, and cause-specific deaths for 2000–19 for the countries we included.^6^ For all analyses, we excluded data for 2020–23 to avoid COVID-19 effects, which were analysed separately.^7^

For WPP data, we divided the 1970–2050 period into decades. For all periods except the 2020s, the first year of the decadal period is the first year of the decade (ie, the year ending in zero; for example, for the 1970s, the first year is 1970); for the 2020s, the first year of the decadal period is 2019, rather than 2020, to avoid COVID-19 effects. For all periods except the 2010s, the last year of the decadal period is the first year of the subsequent decade (eg, for the 1970s, the last year of the decade is 1980). For the 2010s, the last year of the decadal period is 2019, rather than 2020, to avoid COVID-19 effects. Our analysis of the projected CDRs and population sizes from 2024 to 2050 are based on the projections made by the WPP. For analyses using GHE data (ie, cause-specific analyses), we used the 5-year age groups provided by GHE, with an open-ended group for 85 years and older. For estimating decadal changes in cause-specific mortality rates, we combined the estimates into larger age groups (ie, 0–14, 15–49, 50–69, and ≥70 years).

### CDR and decomposition

CDR is defined as the total number of deaths divided by the total mid-year population, reported per 1000 population. We decomposed single-age life tables globally and by region.

For projections into the future (2024–50), we used the projections conducted by WPP (and therefore their assumptions on mortality, fertility, and migration) and decomposed the changes in CDR into the fractions due to changes in population age structure and changes in age-specific mortality rates.

We first calculated C_x_, the proportion of the total population that is age x, for each region and year:

$$C_{x}=\frac{P_{x}}{\sum_{0}^{100+}P_{x}}$$

where *P*_x_ is the mid-year population that is single-age *x*. We then calculated the CDR for the first and final year of each decade and subtracted to calculate the decadal change in CDR, Δ.

$${\text{CDR}}^{R,Y}=\sum_{x=0}^{100+}C_{x}^{R,Y}M_{x}^{R,Y}$$

*R* is the region, *Y* is the year, *x* is the single-year age, and *M*_x_ is the age-specific mortality rate.

$$\Delta ={\text{CDR}}^{R,Y}1-{\text{CDR}}^{R,Y}0$$

*Y*_1_ is the final year and *Y*_0_ is the first year of the decade.

Lastly, we decomposed the decadal change in CDR into two components: the contribution of changes to the population structure and changes in age-specific mortality rates. The former is represented by the decadal difference in population structure,

$$C_{x}^{R,Y_{1}}-C_{x}^{R,Y_{0}}$$

multiplied by the decade average age-specific mortality rate,

$$\frac{M_{x}^{R,Y_{0}}+M_{x}^{R,Y_{1}}}{2}$$

summed across all ages. Similarly, the latter is represented by the decadal difference in age-specific mortality rates,

$$M_{x}^{R,Y_{1}}-M_{x}^{R,Y_{0}}$$

multiplied by the decade average population structure,

$$\frac{C_{x}^{R,Y_{0}}+C_{x}^{R,Y_{1}}}{2}$$

summed across all ages:

$$\Delta =\sum_{x=0}^{100+}(C_{x}^{R,Y_{1}}-C_{x}^{R,Y_{0}})\left[\frac{M_{x}^{R,Y_{0}}+M_{x}^{R,Y_{1}}}{2}\right]+\sum_{x=0}^{100+}(M_{x}^{R,Y_{1}}-M_{x}^{R,Y_{0}})\left[\frac{C_{x}^{R,Y_{0}}+C_{x}^{R,Y_{1}}}{2}\right]$$

### Priority conditions and decomposition of number of deaths

In the third CIH report and elsewhere, we showed that in 2019 a relatively narrow set of conditions contribute to about 80% of the life expectancy gap between most regions and the best performing region, which was the north Atlantic (comprising Canada and western European countries).^7^, ^8^ These 15 priority conditions comprised eight infections and neonatal and maternal health conditions (the so-called I-8) and seven non-communicable diseases (NCDs) and injuries (the so-called NCDI-7).

In a previous study,^8^ we quantified how much 145 causes of death available in the GHE contributed to the life expectancy gap in global regions and countries compared with the north Atlantic, the best performing region.^7^ The 15 priority conditions included the major diseases that accounted for largest shares of the life expectancy gap. We also included road injury and suicide (self-harm) to reflect the burden of injuries and mental health disorders (since by design mental health disorders account for little of the deaths in GHE). Specifically, I-8 includes childhood-cluster diseases (including whooping cough, diphtheria, measles, and tetanus), diarrhoeal diseases, HIV/AIDS, lower respiratory tract infections, malaria, maternal conditions, neonatal conditions, and tuberculosis. NCDI-7 refers to atherosclerotic cardiovascular disease (CVD), diabetes, haemorrhagic stroke, infection-associated NCDs, road injury, strongly tobacco-associated NCDs, and suicide. The specific causes of death included in each NCDI-7 group are shown in the appendix (p 5).

We estimated the cause-specific and age-specific mortality rates for each condition. For comparisons over time, cause-specific mortality rates were age standardised using WHO's standard population.^9^ Similar to the CDR above, we performed two sets of decomposition analyses for 2000s and 2010s to attribute the changes in CDR to changes in demography (population size and age) or cause-age-specific mortality. The first set decomposed the average annual rate of change (AARC) of cause-specific deaths into changes in mortality rates versus population size. The second set decomposed the number of deaths as the product of three factors: population size, population age structure, and cause-specific and age-specific mortality rates, which correspond to the three terms shown below:

$${\text{Deaths}}_{c,\gamma }=\sum_{a}\text{pop}\;{\text{size}}_{\gamma }\cdot \frac{\text{pop}\;{\text{age}}_{a,\gamma }}{\text{pop}\;{\text{size}}_{\gamma }}\cdot \frac{{\text{death}}_{c,a,\gamma }}{\text{pop}\;\text{age}{\;}_{a,\gamma }}$$

where *a* is the 5-year age group, *c* is the cause of death, and *y* represents the year. The deaths associated with each of the three components sum to the total change in deaths over the period. This decomposition approach measures the additive contribution of each factor to changes in the number of deaths by cause and does not capture the interactions between factors.^10^

### Calculation of AARC

The AARC quantifies the consistent annual rate at which a particular health measure grows or declines across the period under consideration. If we assume discrete changes by a constant fraction of AARC at the end of each year over a period of *t* years, and that the initial and final levels of the indicator are *N*_0_ and *N*_1_, then:

$$\text{AARC}={\left(\frac{N_{1}}{N_{0}}\right)}^{\frac{1}{t}}-1$$

### Role of the funding source

The funders of the study had no role in study design, data collection, data analysis, data interpretation, or writing of the report.

## Results

**Figure 2:**
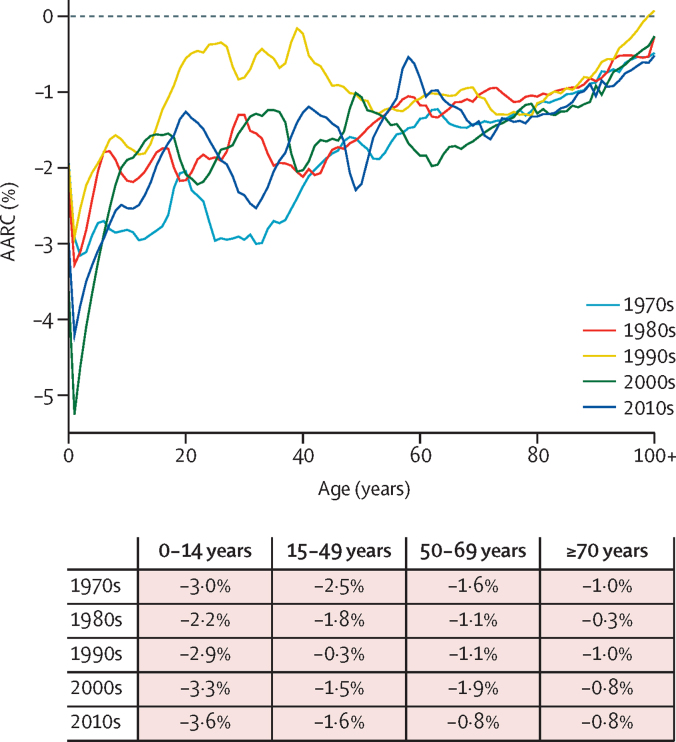
Global average annual rate of change in all-cause mortality by decade and age AARC=average annual rate of change.

Between 1970 and 2019 large decreases in age-specific all-cause mortality rates were observed worldwide. Global age-specific mortality for ages 0–14 years was 15 per 1000 population in 1970 and 3 per 1000 population in 2019, representing a near 80% decline (appendix pp 6–8). For the other age groups, the reductions were 53% for ages 15–49 years, 48% for ages 50–69 years, and 32% for ages 70 years and older. Figure 2 depicts the AARC by age and decade for the world. The largest declines were observed in the younger ages. For example, for age zero the AARCs were –1·9% in the 1970s, –3·6% in the 2000s, and –3·0% in the 2010s. Similar patterns are observed in other ages (figure 2). For many ages, the highest rates of mortality decline have occurred during the last two decades. This finding has occurred despite age-specific mortality rates having mostly started at lower levels at the beginning of the recent decades. The appendix shows the results by region (pp 6–8, 10–14) and by sex (pp 9, 15).

**Figure 3:**
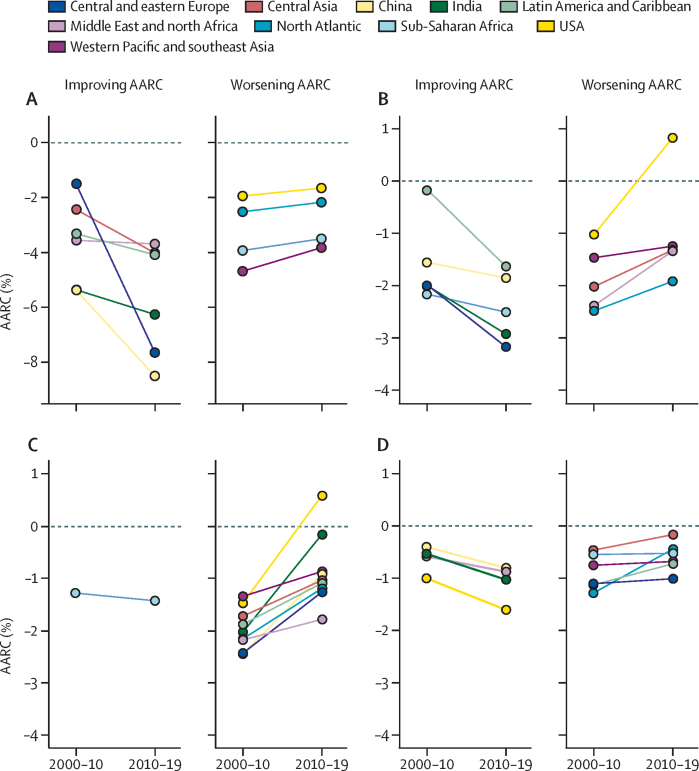
AARC by decade, age group, and region (A) 0–14 years. (B) 15–49 years. (C) 50–69 years. (D) ≥70 years. AARC=average annual rate of change.

Focusing specifically on the most recent two decades, we compared the AARC of all-cause mortality rates in 2000–10 (2000s) and 2010–19 (2010s) by age and region (figure 3; the appendix summarises the results by sex [pp 16–17] and a comparison of 2010–14 and 2015–19 [p 18]). In the 2000s, global AARC ranged from –3·3% in the age group 0–14 years to –0·8% in the age group 70 years and older. In the 2010s, AARC ranged from –3·6% in the age group 0–14 years to –0·8% in the age group 70 years and older. As points of reference, AARC of –2·3% will lead to halving of mortality rates in 30 years, and –3·5% will halve mortality in 20 years. We observed faster declines in the 2010s than in the 2000s in many regions and age groups, and slower declines in some regions and age groups.

First, in ages 0–14 years, six regions had faster declines in mortality rates in the 2010s than in the 2000s, whereas four regions had slower declines (figure 3A). China, central and eastern Europe, and India had the largest improvements, with AARCs of –8·5%, –7·6%, and –6·3% in the 2010s, respectively. Latin America and the Caribbean and central Asia had slightly slower declines in the 2010s, although remaining at approximately –4% per year. For the age group 15–49 years, five regions had faster declines in the 2010s, most notably central and eastern Europe, India, and sub-Saharan Africa, whereas four regions had slower declines (figure 3B). The outlier is the USA with a positive AARC, indicating an increase in the mortality rate of the age group 15–49 years. Although all other regions have continued to have decreasing mortality rates, the USA has reversed its trajectory and now has increasing rates. For the age group 50–69 years, only sub-Saharan Africa had a faster decline in the 2010s compared with the 2000s (figure 3C). The USA again had a positive increase in the mortality rate of the age group 50–69 years. For the age group 70 years and older, four regions had faster declines in the 2010s than the 2000s, with the USA having the highest decline (figure 3D).

**Figure 4:**
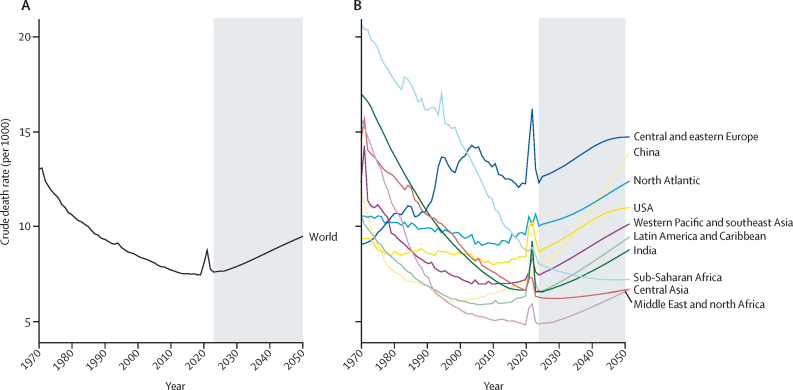
Crude death rate between 1970 and 2050 (A) Global crude death rate. (B) Crude death rate by regions. The grey shaded areas show projections.

Figure 4 shows the trend in CDR from 1970 to 2023, and the projection from 2024 to 2050. Globally, the lowest CDR historically was reported in 2019, at 7·5 per 1000 population. All regions, except central Asia and sub-Saharan Africa, have experienced their lowest CDR and are now experiencing rising rates. Based on the median WPP projection, central Asia is expected to hit its lowest CDR in 2028, whereas sub-Saharan Africa is not expected to reach its lowest CDR until 2047.

The decomposition analysis of the change in CDR into changes in population structure and age-specific mortality rates is presented in the appendix (pp 19–28). Population structure is influenced primarily by the dynamics between fertility and increased longevity (ie, ageing). Recent trends in the population structure as shown in population pyramids in selected countries are summarised in the appendix (p 29). Until recently, the effect of decreasing age-specific mortality rates had outweighed the impact of population ageing. The WPP projects CDR to continue increasing from 2019 to 2050, up to 9·5 per 1000 population. Our analysis shows that, according to the WPP's projections, changes in population structure will place positive or increasing pressure on the CDR. The timing of this effect varies by region—for example, this positive pressure begins in sub-Saharan Africa only in the 2040s—but WPP predicts it will happen for all regions eventually. Changes to age-specific mortality rates, however, will place a negative or decreasing pressure on the CDR. In other words, changes in population structure will have a dominating effect on the CDR because it exerts a pressure that is (1) in the opposite direction to the pressure coming from changing age-specific mortality rates and (2) larger than the pressure of changing age-specific mortality rates. Thus, all net change to the CDR will be due to changes in population structure for all regions eventually (by the 2030s for central Asia and by the 2050s for sub-Saharan Africa), unless major accelerations occur in the decline of age-specific mortality rates. Regional decomposition analysis is presented in the appendix (pp 19–28, 30–32).

The appendix (pp 33–34) shows the global age-standardised cause-specific death rates from 2000 to 2019. Death rates for the 15 priority conditions have all decreased over time except for diabetes. Among I-8, childhood-cluster diseases had the highest proportional decrease over the period (76%), followed by HIV/AIDS (66%), and tuberculosis (65%; appendix p 35). The lowest declines were for lower respiratory tract infections (34%), malaria (40%), and neonatal conditions (41%). Among NCDI-7, haemorrhagic stroke (33%) and suicide (32%) had the highest proportional decrease during the period, followed by infection-associated NCDs (31%) and strongly tobacco-associated NCDs (25%). Among all 15 conditions, only diabetes had increased age-standardised death rate of 9% from 2000 to 2019. We report the percentage change by narrower periods of 2000–09, 2010–14, and 2015–19 in the appendix (p 35).

Proportionally, the 15 priority conditions have accounted for a large share of all deaths globally (appendix pp 36–38). 72% of all deaths in 2000 and 65% in 2019 were from the priority conditions. Across regions, the total proportion of deaths due to the priority conditions declined modestly from 2000 to 2019 except in the north Atlantic and the USA where the decline is more pronounced. The proportion of deaths due to I-8 decreased from 29% to 16%, whereas the proportion due to NCDI-7 increased from 43% to 49%. The proportion of deaths due to the 15 conditions declined the most in north Atlantic (from 57% to 43%) and the USA (from 58% to 45%), due to the proportion of deaths from other causes rising. In sub-Saharan Africa, the proportion of deaths from NCDI-7 has increased by about 60% (from 13% to 21%) and in India it has increased by 80% (from 29% to 52%). The appendix (p 39) further shows the global proportion of deaths by each of the 15 priority conditions over time.

The decomposition analyses of the global AARC for each condition into changes in population size and cause-specific mortality rates are presented in the appendix (pp 40–45). I-8 deaths have declined in the last two decades largely due to declines in cause-specific mortality rates outpacing increases in population size. The one exception is lower respiratory tract infections in 2010s, in which the number of deaths increased. Between 2010 and 2019, the fastest declines in the number of deaths were found for HIV/AIDS (–7·1%), childhood-cluster diseases (–6·6%), and tuberculosis (–5·2%). The slowest declines were found for lower respiratory tract infections (–1·0%), malaria (–3·5%), and neonatal conditions (–3·8%). Among regions with a high proportion of I-8 deaths (>10% of total deaths), the largest declines in 2010–19 were observed in India for childhood-cluster diseases (–15·7%) and central Asia for malaria (–15·1%; appendix pp 46–144). The largest decline in sub-Saharan Africa was for HIV/AIDS (–8·9%), in the Middle East and north Africa for diarrhoeal diseases (–7·8%), and in Latin America and the Caribbean for neonatal conditions (–3·5%). Notably, however, increases in the number of deaths between 2010 and 2019 were observed for HIV/AIDS in central Asia (9·4%), and lower respiratory tract infections in Latin America and the Caribbean (3·1%) and western Pacific and southeast Asia (0·1%).

**Figure 5:**
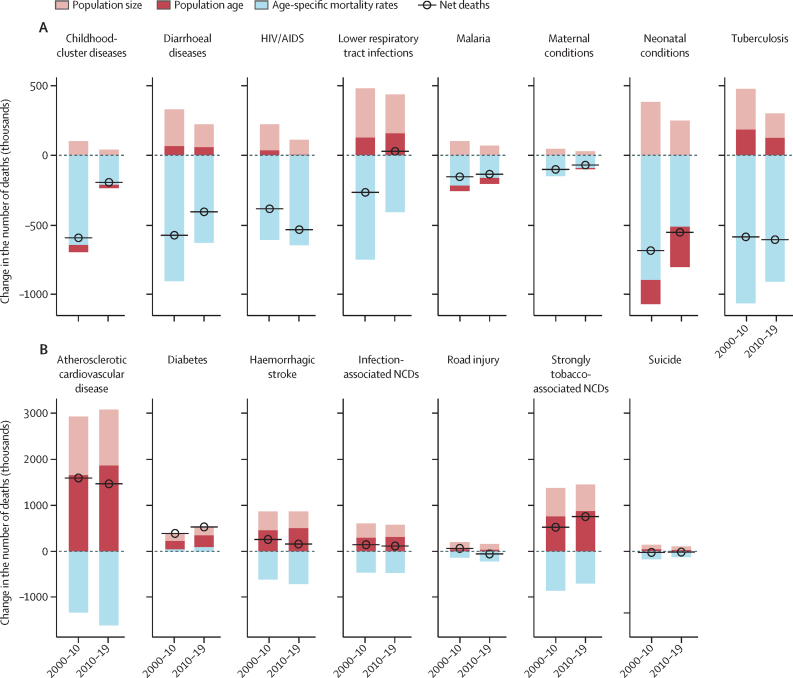
Global decomposition of the number of deaths due to the 15 priority conditions into changes in age-specific mortality rates and changes in population size and structure between 2000–10 and 2010–19 (A) I-8. (B) NCDI-7. I-8=eight infections and neonatal and maternal health conditions. NCDs=non-communicable diseases. NCDI-7=seven NCDs and injuries.

The decomposition of I-8 deaths is presented in figure 5 and in the table; results by region and sex are summarised in the appendix (pp 233–42). The largest contributor to the decline in the number of I-8 deaths is age-specific mortality, followed by changes in population size.

**Table:** Global decomposition of I-8 and NCDI-7 deaths into component (changes in population size, population structure, and age-specific mortality rates) contributions in 2000–10 and 2010–19

		Deaths, year 1*	**Change in deaths**	**Number of deaths contributed by component (N) and share of total component effects (%) over the decade**		
				Population size	Population structure	Age-specific mortality rates
**I-8 deaths**						
2000–10						
	Childhood-cluster diseases	1080	−593	+103 (13%)	−51 (6%)	−644 (81%)
	Diarrhoeal diseases	2300	−574	+264 (21%)	+66 (5%)	−904 (73%)
	HIV/AIDS	1630	−384	+189 (23%)	+35 (4%)	−608 (73%)
	Lower respiratory tract infections	2870	−266	+355 (29%)	+128 (10%)	−749 (61%)
	Malaria	867	−153	+103 (29%)	−39 (11%)	−217 (60%)
	Maternal conditions	410	−101	+47 (24%)	0	−148 (76%)
	Neonatal conditions	3300	−685	+384 (26%)	−175 (12%)	−894 (62%)
	Tuberculosis	2520	−586	+293 (19%)	+185 (12%)	−1060 (69%)
	I-8	15 000	−3340	+1740 (24%)	+148 (2%)	−5230 (74%)
2010–19						
	Childhood-cluster diseases	485	−193	+42 (15%)	−26 (9%)	−209 (76%)
	Diarrhoeal diseases	1730	−406	+165 (19%)	+58 (7%)	−628 (74%)
	HIV/AIDS	1250	−534	+107 (14%)	+5 (1%)	−646 (85%)
	Lower respiratory tract infections	2600	+29	+279 (33%)	+158 (19%)	−408 (48%)
	Malaria	713	−135	+69 (25%)	−44 (16%)	−160 (58%)
	Maternal conditions	310	−70	+30 (23%)	−10 (8%)	−89 (69%)
	Neonatal conditions	2610	−553	+250 (24%)	−291 (28%)	−512 (49%)
	Tuberculosis	1930	−606	+177 (15%)	+125 (10%)	−909 (75%)
	I-8	11 600	−2470	+1120 (24%)	−25 (1%)	−3560 (76%)
**NCDI-7 deaths**						
2000–10						
	Atherosclerotic CVD	8990	+1600	+1270 (30%)	+1660 (39%)	−1330 (31%)
	Diabetes	1080	+392	+164 (42%)	+180 (46%)	+48 (12%)
	Haemorrhagic stroke	3060	+261	+414 (28%)	+457 (31%)	−611 (41%)
	Infection-associated NCDs	2310	+146	+309 (29%)	+296 (28%)	−460 (43%)
	Road injury	1180	+67	+157 (47%)	+45 (13%)	−136 (40%)
	Strongly tobacco-associated NCDs	4530	+526	+621 (28%)	+757 (34%)	−851 (38%)
	Suicide	771	−22	+99 (31%)	+48 (15%)	−168 (53%)
	NCDI-7	21 900	+2970	+3030 (30%)	+3440 (34%)	−3510 (35%)
2010–19						
	Atherosclerotic CVD	10 600	+1470	+1210 (26%)	+1870 (40%)	−1610 (34%)
	Diabetes	1470	+533	+184 (35%)	+258 (48%)	+91 (17%)
	Haemorrhagic stroke	3330	+161	+365 (23%)	+503 (32%)	−708 (45%)
	Infection-associated NCDs	2460	+119	+270 (26%)	+311 (30%)	−462 (44%)
	Road injury	1250	−54	+131 (34%)	+32 (8%)	−217 (57%)
	Strongly tobacco-associated NCDs	5050	+758	+580 (27%)	+874 (41%)	−696 (32%)
	Suicide	749	−14	+80 (34%)	+30 (13%)	−123 (53%)
	NCDI-7	24 900	+2970	+2820 (27%)	+3880 (37%)	−3720 (36%)

CVD=cardiovascular disease. I-8=eight infections and neonatal and maternal health conditions. NCDs=non-communicable diseases. NCDI-7=seven NCDs and injuries.

* The first year of the decadal period (2000 for 2000s, 2010 for 2010s). Deaths in thousands.

The majority of the NCDI-7 had a positive AARC (ie, increasing deaths) since 2000s. This observation is largely due to increases in population size for most of the NCDI-7 (appendix pp 43–45). Once increase in population size is accounted for, cause-specific mortality rates have in fact been decreasing over time for most of the NCDI-7. The exceptions are atherosclerotic CVD (0·3% in 2010s), diabetes (2·3%), and strongly tobacco-associated NCDs (0·4%; appendix pp 145–232). The AARC of diabetes deaths is particularly high, at about 3·5% in 2010s, accelerating from 3·1% in 2000s. Regionally, the fastest decreases in cause-specific mortality rates in the 2010s were found for road injury in central and eastern Europe (–4·3%), north Atlantic (–3·1%), Middle East and north Africa (–2·6%), western Pacific and southeast Asia (–2·3%), and Latin America and the Caribbean (–2·1%). In China and India, the largest declines in cause-specific mortality rates were found for suicide (–2·1% and –1·4%, respectively). Central Asia had the largest decreases in haemorrhagic stroke (–2%), and the USA in atherosclerotic CVD (–0·7%; appendix pp 145–232). Even after adjusting for population change, increasing death rates for diabetes are observed in all regions in 2010s but are particularly high in central and eastern Europe (8·2%) and India (4·8%). The second decomposition further shows that changes in deaths have increased largely because of changes in population age structure (population ageing) and size (figure 5). For all NCDI-7 except diabetes, age-specific mortality rates have in fact been decreasing over time. Diabetes stands out because of its continuous increase in the number of deaths even after accounting for population age and size (appendix pp 243–46). Increasing diabetes age-specific mortality rates have occurred in 2010s in central and eastern Europe, India, Middle East and north Africa, the USA, and western Pacific and southeast Asia. Beyond diabetes, we observed positive increases in age-specific mortality rates only for suicide in Latin America and the Caribbean and the USA, atherosclerotic CVD and strongly tobacco-associated NCDs in India, and road injury in the USA.

## Discussion

Our study summarises some of the major epidemiological and demographic trends in global health between 1970 and 2050, with a particular focus on the last two decades (2000s and 2010s). The most rapid declines in age-specific all-cause mortality rates for most age groups have been observed in the last two decades, but the patterns vary by region. In some regions, such as China and India, the rate of mortality decline for most age groups has continued to accelerate, whereas in the USA mortality rates in the middle age groups increased. Globally, the historically lowest CDR occurred in 2019, and CDR is projected to continue to increase unless substantial technological changes occur. Cause-specific death rates from I-8 have continued to fall, but most countries need to focus on reducing death rates from diabetes, atherosclerotic CVDs, and strongly tobacco-related NCDs.

On the basis of these results, we derived three key messages to inform the third CIH: optimism, inevitability, and focus. First, there is reason to be optimistic about the future of global health. Impressive reductions have been made in age-specific and cause-specific mortality rates across the geographical and income spectrum. This observation is consistent with the conclusions from the first and second CIH.^11^, ^12^ However, some caveats exist. This paper focuses the analyses on both sexes combined, but we observed large sex differences in some health outcomes in certain regions and countries. All analyses are reported by sex in the appendix. As reported elsewhere, we observed that life expectancy among males in every country in central and eastern Europe is much lower than among females. For example, in Russia, Ukraine, Belarus, and Georgia in 2019, the female-to-male life expectancy gap was about 10 years.^13^ In Viet Nam and Thailand, the gap was about 9 years. In our cause-specific analyses, we found that atherosclerotic CVD death rates are increasing among males in sub-Saharan Africa and the USA, but not among females. Strongly tobacco-associated NCD death rates are increasing among Chinese males but not females. In contrast, we observed increasing mortality rates due to infection-associated NCDs among Indian females but not males. Additionally, females had increasing mortality rates due to strongly tobacco-associated NCDs in central and eastern Europe, north Atlantic, and sub-Saharan Africa, whereas these rates were declining for males. Furthermore, although the gaps in life expectancy and other health outcomes between regions and the best performing region have narrowed rapidly, the gaps remain high.^8^ For example, the gap in life expectancy between sub-Saharan Africa and north Atlantic decreased from 27 years in 2000 to 22 years in 2019. Despite the impressive reduction over time, a gap of 22 years in 2019 is still not acceptable.

Second, we need to confront the inevitability of demographic change. The combination of declining fertility rates and substantial improvements in survival have led to shifts in population size and age structure, with large increases in the proportion of older age groups and older deaths.^7^ Nearly 50% of deaths in 2019 occurred in those aged 70 years or older, and this share is projected to increase to nearly 70% in 2050 (64% in India, and close to 90% in China and the north Atlantic by 2050). This demographic change has large implications for health financing and health-care provision. Improved survival will also likely lead to increasing burden of non-fatal outcomes, which will create further demands for health services and financing. Even with continuing health-care advancements, cause-specific mortality rates for NCDs in particular will likely increase, underscoring the dominant influence of demographic shifts on demands on the health system into the future.^14^ Furthermore, the narrowing base of the population structure due to the decline in fertility rates means that an insufficient tax base to support a growing older age population might occur.^7^

Third, and related to the second point, with increasing demands on health financing and health-care provision, there is a need for the health sector to focus. We proposed a strategy focused on the prevention and treatment of the 15 priority conditions, I-8 and NCDI-7, which account for a large proportion of all deaths and the gap in life expectancy between each region and north Atlantic (appendix pp 36–39).^8^ Focusing on these conditions will lead to large reductions in health inequality across countries. We have further identified several causes with large disease burden and with increasing cause-specific mortality, such as atherosclerotic CVD, diabetes, and strongly tobacco-associated NCDs (appendix pp 40–42). For some conditions, mortality reductions are decelerating, including diarrhoeal diseases, lower respiratory tract infections, maternal conditions, childhood-cluster diseases, and suicide (appendix pp 40–42).^15^ Focusing on reversing these detrimental trends will contribute to reducing disease burden as well as financing and health-care system burdens.

This study has several limitations. First, we have relied on different data sources for different sets of analyses: WPP2024 for all-cause mortality and demographic analyses, and GHE2021 for cause-specific mortality analysis. One data source is preferred to ensure consistency; however, because of the scope and limitations of each, our different sets of analyses might not sum up. Second, WPP and GHE depend on the quality of mortality data from countries, and a large proportion of the world does not have reliable data sources, which affects data quality. WPP projections involve their expert assumptions about future fertility, mortality, and migration trends, which our analyses reflect. Issues related to death certificates, including whether disease trends observed are due to real epidemiological change or changes in what is captured through death certificates, are relevant for our study.^16^ Our analyses are bounded by the limitations of the data sources themselves. Third, our analyses are descriptive and not inferential—ie, we do not seek to explain the reasons why these trends exist beyond decomposition. The analyses would be more powerful if the reasons could be pinpointed—for example, how certain regions reduced their mortality rates by introducing certain health policies or interventions. Fourth, the decomposition analyses do not account for the interactions between epidemiological and demographic changes, even though in reality the change in the population structure itself is partly influenced by the change in underlying mortality rates.

While there is reason for optimism about global health progress, we also highlight persistent disparities and emerging challenges. Key recommendations include prioritising interventions to address specific health challenges and recognising the need to adapt health systems to demographic changes.

### Contributors

### Data sharing

All data used in the paper are publicly available. All processed data are presented in this Article and the appendix.

## Declaration of interests

We declare no competing interests.
