# Supplementary material for: Epidemiological and demographic trends and projections in global health from 1970 to 2050: a descriptive analysis from the third Lancet Commission on Investing in Health, Global Health 2050
Source: Lancet. 2025 Sep 30;406(10506):940–9. doi: 10.1016/S0140-6736(25)00902-X (PMC12394659; doi:10.1016/S0140-6736(25)00902-X)
Supplement: Supplementary appendix [file mmc1.pdf]

THE LANCET

Supplementary appendix

This appendix formed part of the original submission and has been peer reviewed. We post it as supplied by the authors.

Supplement to: Chang AY, Bolongaita S, Cao B, et al. Epidemiological and demographic trends and projections in global health from 1970 to 2050: a descriptive analysis from the third Lancet Commission on Investing in Health, Global Health 2050. *Lancet* 2025; **406:** 940–49.

Epidemiological and demographic trends and projections in global health from 1970 to 2050: descriptive analysis from the 3rd *Lancet* Commission on Investing in Health, Global Health 2050

Appendix Tables and Figures

Angela Y. Chang[1,2]

Sarah Bolongaita[3]

Bochen Cao[4]

Marcia C. Castro[5]

Omar Karlsson[6,7]

Wenhui Mao[8,9]

Ole F. Norheim[3,5]

Osondu Ogbuoji[9,10]

Dean T. Jamison[11]

[1] Danish Institute for Advanced Study, University of Southern Denmark, Denmark

[2] Danish Centre for Health Economics, University of Southern Denmark, Denmark

[3] Bergen Centre for Ethics and Priority Setting (BCEPS), Department of Global Public Health and Primary Care, University of Bergen, Norway

[4] Data, Analytics and Delivery for Impact Unit, World Health Organization

[5] Department of Global Health and Population, Harvard T.H. Chan School of Public Health, USA

[6] Duke University Population Research Institute, Duke University, USA

[7] Centre for Economic Demography, School of Economics and Management, Lund University, Sweden

[8] Duke Global Health Innovation Center and Innovations in Healthcare, Duke University, USA

[9] Centre for Policy Impact in Global Health, Duke University, USA

[10] Department of Population Health, Duke School of Medicine, Duke University, Durham, NC, USA.

[11] Institute for Global Health Sciences, University of California, San Francisco, USA

Table of Contents

Table A1. Countries by 3rd Lancet Commission on Investing in Health region. 3

Table A2. Causes of death, according to the World Health Organization's Global Health Estimates (GHE) 2021, included in the I-8 and NCD-7 conditions. 5

Table A3. All-cause mortality rates and decadal average annual rates of change (AARC) by age group and region, 1970-2019. 6

Table A4. Global average annual rate of change in all-cause mortality rates by decade, age group, and sex. 9

Figure A1. Annual rate of change in all-cause mortality rates by decade, age, and region. 10

Figure A2. Global average annual rate of change in all-cause mortality by decade, age, and sex. 15

Figure A3. Average annual rate of change (AARC) by sex, age group, decade, and region. 16

Figure A4. Average annual rate of change (AARC) by age group and region, 2010-2014 and 2015-2019. 18

Figure A5. Decomposition of decadal changes in the crude death rate per 1000 population per year into component (changes in population structure, changes in age-specific mortality rates) contributions for the world and by region, 1970-2050. 19

Figure A6. Population pyramids for selected countries: Nigeria, India, United States, and Japan, 1970, 2019, and 2050. 29

Table A5. Decomposition of decadal changes crude death rate (CDR) per 1000 per year into component (changes in population structure, changes in age-specific mortality rates) contributions by region, 1970-1950. 30

Figure A7. Age-standardized death rates of I-8 and NCDI-7 conditions for the World, 2000-2019, for both sexes and by sex. 33

Table A6. Age-standardized death rates of I-8 and NCDI-7 conditions for the World, 2000, 2019. 35

Figure A8. Proportion of I-8 and NCDI-7 deaths by region and by sex in 2000, 2010, 2019. 36

Figure A9. Proportion of deaths by priority conditions, World in 2000, 2010, and 2019. 39

Table A7. Decomposition of I-8 and NCDI-7 conditions into component (changes in population size and age-specific mortality rates), globally for (A) both sexes, (B) females and (C) males 2000-2010, 2010-2019. 40

Figure A10. Decomposition of the average annual rate of change of the 15 priority conditions into changes in cause-specific mortality rates and changes in population size, World, 2000-2010, 2010-2019. 43

Table A8. Rates of change in population size and cause-specific number of deaths and death rates for I-8 conditions, 2000-2010, 2010-2019, and 2000-2019. 46

Table A9. Rates of change in population size and cause-specific number of deaths and death rates for NCDI-7 conditions, 2000-2010, 2010-2019, and 2000-2019. 145

Table A10. Decomposition of I-8 and NCDI-7 deaths into component (changes in population size, population structure, and age-specific mortality rates) contributions, by sex for World, 2000-2010, 2010-2019. 233

Figure A11. Decomposition of the number of deaths due to the 15 priority conditions into changes in age-specific mortality rates and changes in population size and structure, by sex for the World, 2000-2010, 2010-2019. 237

Table A11. Decomposition I-8 deaths into component (changes in population size, population structure, and age-specific mortality rates) contributions by region, 2000-2010 and 2010-2019. 239

Table A12. Decomposition NCD-7 deaths into component (changes in population size, population structure, and age-specific mortality rates) contributions by region, 2000-2010 and 2010-2019. 243

Table A1. Countries by 3rd Lancet Commission on Investing in Health region.

Central Asia		
Afghanistan	Azerbaijan	Kazakhstan
Kyrgyz Republic	Mongolia	Pakistan
Tajikistan	Turkmenistan	Uzbekistan
Central and Eastern Europe		
Albania	Armenia	Belarus
Bosnia and Herzegovina	Bulgaria	Croatia
Czech Republic	Estonia	Georgia
Hungary	Latvia	Lithuania
Moldova	Montenegro	North Macedonia
Poland	Romania	Russian Federation
Serbia	Slovak Republic	Slovenia
Ukraine		
China		
India		
Latin America and Caribbean		
Argentina	Bahamas, The	Belize
Bolivia	Brazil	Chile
Colombia	Costa Rica	Cuba
Dominican Republic	Ecuador	El Salvador
Guatemala	Guyana	Haiti
Honduras	Jamaica	Mexico
Nicaragua	Panama	Paraguay
Peru	Suriname	Trinidad and Tobago
Uruguay	Venezuela, RB	
Middle East and North Africa		
Algeria	Bahrain	Egypt, Arab Rep.
Iran, Islamic Rep.	Iraq	Israel
Jordan	Kuwait	Lebanon
Libya	Morocco	Oman
Qatar	Saudi Arabia	Syrian Arab Republic
Tunisia	Türkiye	United Arab Emirates
Yemen, Rep.		
North Atlantic		
Austria	Belgium	Canada
Cyprus	Denmark	Finland
France	Germany	Greece
Iceland	Ireland	Italy
Luxembourg	Malta	Netherlands
Norway	Portugal	Spain
Sweden	Switzerland	United Kingdom
Sub-Saharan Africa		
Angola	Benin	Botswana
Burkina Faso	Burundi	Cabo Verde

Cameroon	Central African Republic	Chad
Comoros	Congo, Dem. Rep.	Congo, Rep.
Côte d'Ivoire	Djibouti	Equatorial Guinea
Eritrea	Eswatini	Ethiopia
Gabon	Gambia, The	Ghana
Guinea	Guinea-Bissau	Kenya
Lesotho	Liberia	Madagascar
Malawi	Mali	Mauritania
Mauritius	Mozambique	Namibia
Niger	Nigeria	Rwanda
Senegal	Sierra Leone	Somalia
South Africa	South Sudan	Sudan
Tanzania	Togo	Uganda
Zambia	Zimbabwe	
United States		
Western Pacific and Southeast Asia		
Australia	Bangladesh	Bhutan
Brunei Darussalam	Cambodia	Fiji
Indonesia	Japan	Korea, Dem. People's Rep.
Korea, Rep.	Lao PDR	Malaysia
Maldives	Myanmar	Nepal
New Zealand	Papua New Guinea	Philippines
Singapore	Solomon Islands	Sri Lanka
Thailand	Timor-Leste	Vanuatu
Vietnam		

Note: Countries were included in a CIH region if they were United Nations Member States with populations of at least 300 000 in 2024. For the CIH World region, if an input dataset contained a World region, those values were used for the CIH World region; if a dataset did not contain a World region, values for the CIH World region were calculated from all locations with available data, regardless of UN Member State status or population size.

Table A2. Causes of death, according to the World Health Organization's Global Health Estimates (GHE) 2021, included in the I-8 and NCD-7 conditions.

I-8	GHE causes of death
1 Childhood-cluster diseases	Childhood-cluster diseases<br>(whooping cough, diphtheria, measles, tetanus)
2 Diarrheal diseases	Diarrheal diseases
3 HIV/AIDS	HIV/AIDS
4 Lower respiratory infections	Lower respiratory infections
5 Malaria	Malaria
6 Maternal conditions	Maternal conditions
7 Neonatal conditions	Neonatal conditions
8 Tuberculosis	Tuberculosis
**NCDI-7**	**GHE causes of death**
1 Atherosclerotic CVD[a]	Ischemic heart disease<br>Ischemic stroke
2 Diabetes	Chronic kidney disease due to diabetes<br>Diabetes mellites
3 Hemorrhagic stroke	Hemorrhagic stroke
4 Infection-associated NCDs[b]	Cervical cancer<br>Cirrhosis due to hepatitis B<br>Cirrhosis due to hepatitis C<br>Liver cancer secondary to hepatitis B<br>Liver cancer secondary to hepatitis C<br>Rheumatic heart disease<br>Stomach cancer
5 Road injury	Road injury
6 Strongly tobacco-associated NCDs[b]	Chronic obstructive pulmonary disease<br>Larynx cancer<br>Mouth and oropharynx cancer<br>Trachea, bronchus, and lung cancer
7 Suicide	Self-harm

[a] Cardiovascular disease
[b] Noncommunicable diseases

Table A3. All-cause mortality rates and decadal average annual rates of change (AARC) by age group and region, 1970-2019.

	0-14		15-49		50-69		70+		All ages (CDR)	
	First year mortality rate	Decade AARC	First year mortality rate	Decade AARC	First year mortality rate	Decade AARC	First year mortality rate	Decade AARC	First year mortality rate	Decade AARC
World										
1970s	14.7	-3.0%	4.5	-2.5%	21.5	-1.6%	90.6	-1.0%	13.0	-2.0%
1980s	10.8	-2.2%	3.4	-1.8%	18.2	-1.1%	81.6	-0.3%	10.6	-1.3%
1990s	8.6	-2.9%	2.9	-0.3%	16.4	-1.1%	78.9	-1.0%	9.3	-1.0%
2000s	6.4	-3.3%	2.8	-1.4%	14.7	-1.9%	71.7	-0.8%	8.5	-0.9%
2010s	4.6	-3.6%	2.4	-1.6%	12.1	-0.8%	66.3	-0.8%	7.7	-0.4%
2019	3.3		2.1		11.2		61.6		7.5	
Central and Eastern Europe										
1970s	2.7	-1.0%	2.7	+1.4%	16.9	+0.1%	83.0	-0.3%	9.1	+1.7%
1980s	2.4	-3.8%	3.1	-1.4%	17.0	+0.2%	80.6	+0.7%	10.7	+0.4%
1990s	1.6	-3.7%	2.7	+3.9%	17.3	+1.8%	86.4	-0.3%	11.1	+2.1%
2000s	1.1	-1.5%	3.9	-2.0%	20.7	-2.4%	83.6	-1.1%	13.6	-0.2%
2010s	1.0	-7.6%	3.2	-3.2%	16.2	-1.3%	74.8	-1.0%	13.3	-0.9%
2019	0.5		2.4		14.5		68.2		12.3	
Central Asia										
1970s	18.8	-1.6%	5.2	-2.6%	23.3	-1.4%	99.5	-0.6%	14.6	-1.4%
1980s	16.1	-2.3%	4.0	-2.4%	20.2	-0.6%	94.0	+0.1%	12.6	-1.7%
1990s	12.7	-3.4%	3.1	-0.1%	18.9	+0.4%	94.6	-0.5%	10.6	-1.7%
2000s	9.0	-2.4%	3.1	-2.0%	19.6	-1.7%	89.8	-0.5%	9.0	-1.7%
2010s	7.0	-4.0%	2.5	-1.3%	16.5	-1.0%	85.7	-0.2%	7.6	-1.4%
2019	4.8		2.2		15.0		84.4		6.7	
China										
1970s	12.2	-8.1%	4.2	-4.2%	26.5	-3.2%	109	-2.2%	11.9	-4.4%
1980s	5.3	-0.2%	2.8	-3.3%	19.1	-2.3%	87.9	-1.1%	7.6	-1.0%
1990s	5.1	-6.4%	2.0	-2.2%	15.1	-2.5%	78.3	-1.5%	6.9	-1.2%
2000s	2.6	-5.4%	1.6	-1.6%	11.7	-2.4%	67.3	-0.4%	6.1	+0.6%
2010s	1.5	-8.5%	1.4	-1.8%	9.1	-0.9%	64.7	-0.8%	6.5	+1.0%

	0-14		15-49		50-69		70+		All ages (CDR)	
	First year mortality rate	Decade AARC	First year mortality rate	Decade AARC	First year mortality rate	Decade AARC	First year mortality rate	Decade AARC	First year mortality rate	Decade AARC
2019	0.7		1.1		8.4		60.1		7.1	
India										
1970s	22.4	-2.5%	6.2	-3.2%	26.8	-0.8%	100	-0.7%	17.0	-2.1%
1980s	17.4	-3.6%	4.5	-2.9%	24.7	-1.2%	93.9	-0.1%	13.8	-2.4%
1990s	12.1	-4.0%	3.4	-0.7%	21.8	-1.4%	92.9	-1.1%	10.7	-2.1%
2000s	8.0	-5.3%	3.1	-2.0%	19.0	-2.0%	83.3	-0.5%	8.6	-1.8%
2010s	4.6	-6.2%	2.5	-2.9%	15.5	-0.2%	79.0	-1.0%	7.2	-0.8%
2019	2.6		1.9		15.3		71.9		6.7	
Latin America and Caribbean										
1970s	11.6	-4.1%	4.1	-2.2%	20.5	-1.5%	92.9	-1.0%	10.6	-2.4%
1980s	7.6	-4.7%	3.3	-2.2%	17.7	-1.1%	83.5	-0.5%	8.3	-1.8%
1990s	4.7	-5.3%	2.6	-1.3%	15.8	-1.6%	79.4	-1.1%	6.9	-1.2%
2000s	2.7	-3.3%	2.3	-0.2%	13.5	-1.9%	71.3	-1.1%	6.1	0.0%
2010s	1.9	-4.1%	2.3	-1.6%	11.2	-1.1%	63.6	-0.7%	6.1	+0.4%
2019	1.3		2.0		10.2		59.5		6.4	
Middle East and North Africa										
1970s	21.0	-5.0%	5.0	-3.1%	22.6	-1.3%	98.5	-0.6%	15.8	-3.6%
1980s	12.5	-6.6%	3.7	-3.4%	20.0	-1.0%	93.0	-0.4%	11.0	-3.9%
1990s	6.3	-6.1%	2.6	-3.4%	18.0	-2.0%	89.0	-1.2%	7.4	-2.7%
2000s	3.4	-3.5%	1.8	-2.4%	14.8	-2.2%	79.2	-0.6%	5.6	-0.9%
2010s	2.4	-3.7%	1.4	-1.3%	11.9	-1.8%	74.6	-0.9%	5.1	-0.5%
2019	1.7		1.3		10.1		68.9		4.8	
North Atlantic										
1970s	2.0	-5.9%	1.8	-1.7%	15.6	-1.9%	81.5	-1.0%	10.6	-0.3%
1980s	1.1	-3.8%	1.5	-1.2%	12.9	-1.6%	73.6	-0.4%	10.2	-0.3%
1990s	0.7	-4.8%	1.3	-1.3%	11.0	-2.4%	70.7	-1.4%	9.9	-0.4%
2000s	0.5	-2.5%	1.2	-2.5%	8.6	-2.1%	61.5	-1.3%	9.5	-0.4%
2010s	0.4	-2.2%	0.9	-1.9%	6.9	-1.2%	54.0	-0.4%	9.1	+0.6%

	0-14		15-49		50-69		70+		All ages (CDR)	
	First year mortality rate	Decade AARC	First year mortality rate	Decade AARC	First year mortality rate	Decade AARC	First year mortality rate	Decade AARC	First year mortality rate	Decade AARC
2019	0.3		0.8		6.2		51.9		9.6	
Sub-Saharan Africa										
1970s	28.7	-1.9%	8.4	-1.6%	26.9	-0.9%	108	-0.7%	20.7	-1.6%
1980s	23.6	-1.5%	7.2	+0.1%	24.6	+0.1%	100	-0.1%	17.7	-0.9%
1990s	20.2	-2.1%	7.2	+0.2%	25.0	+0.1%	99.4	-0.3%	16.2	-1.2%
2000s	16.4	-3.9%	7.4	-2.2%	25.3	-1.3%	96.2	-0.6%	14.3	-2.7%
2010s	11.0	-3.5%	5.9	-2.5%	22.2	-1.4%	91.0	-0.5%	10.9	-2.4%
2019	8.0		4.7		19.5		86.8		8.8	
United States										
1970s	1.9	-3.5%	2.4	-2.8%	16.6	-1.7%	76.0	-1.2%	9.4	-0.8%
1980s	1.3	-2.3%	1.8	0.0%	13.9	-1.0%	67.5	-0.6%	8.7	-0.1%
1990s	1.0	-4.0%	1.8	-1.0%	12.6	-2.4%	63.4	+0.4%	8.6	0.0%
2000s	0.7	-1.9%	1.6	-1.0%	9.9	-1.5%	65.9	-1.0%	8.6	-0.7%
2010s	0.6	-1.6%	1.4	+0.8%	8.5	+0.6%	59.6	-1.6%	8.0	+0.5%
2019	0.5		1.6		9.0		51.5		8.4	
Western Pacific and Southeast Asia										
1970s	14.5	-3.5%	4.8	-3.3%	20.9	-2.2%	91.4	-1.3%	12.3	-2.5%
1980s	10.1	-3.7%	3.4	-2.3%	16.7	-1.2%	80.0	-0.6%	9.6	-1.7%
1990s	6.9	-4.3%	2.7	-0.9%	14.8	-0.8%	75.2	-1.3%	8.1	-1.1%
2000s	4.4	-4.7%	2.5	-1.5%	13.6	-1.3%	65.9	-0.8%	7.2	-0.3%
2010s	2.8	-3.8%	2.1	-1.2%	11.9	-0.9%	61.1	-0.7%	7.0	+0.3%
2019	1.9		1.9		11.0		57.5		7.2	

Notes: First year mortality rate = The mortality rate, reported per 1000 population, during the first year of the decadal period. For all periods, the first year of the decadal period is the first year of the decade (i.e., the year ending in -0; e.g., for the 1970s, the first year is 1970). For all periods except the 2010s, the last year of the decadal period is the first year of the subsequent decade (e.g., for the 1970s, the last year is 1980). For the 2010s, the last year of the decadal period is 2019, rather than 2020, to avoid COVID-19 impacts. CDR = Crude death rate.

Table A4. Global average annual rate of change in all-cause mortality rates by decade, age group, and sex.

Decade	0-14	15-49	50-69	70+
Females				
1970-1980	-3.0%	-3.1%	-1.8%	-1.2%
1980-1990	-2.2%	-2.1%	-1.2%	-0.3%
1990-2000	-3.0%	-0.4%	-1.2%	-0.8%
2000-2010	-3.3%	-1.6%	-2.2%	-0.8%
2010-2019	-3.7%	-1.9%	-0.8%	-0.8%
Males				
1970-1980	-3.0%	-2.1%	-1.5%	-0.8%
1980-1990	-2.2%	-1.7%	-1.0%	-0.4%
1990-2000	-2.9%	-0.2%	-1.1%	-1.2%
2000-2010	-3.2%	-1.3%	-1.8%	-0.8%
2010-2019	-3.5%	-1.4%	-0.8%	-0.9%

Figure A1. Annual rate of change in all-cause mortality rates by decade, age, and region.

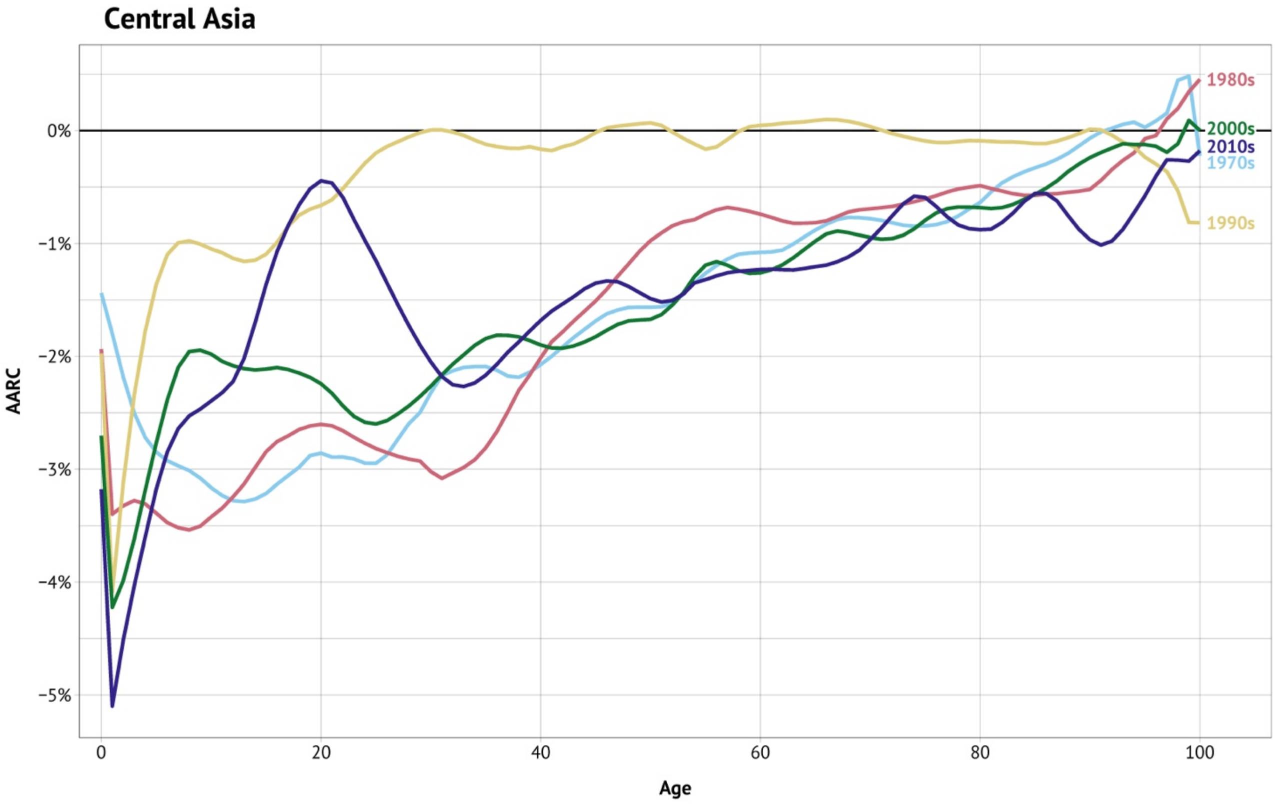

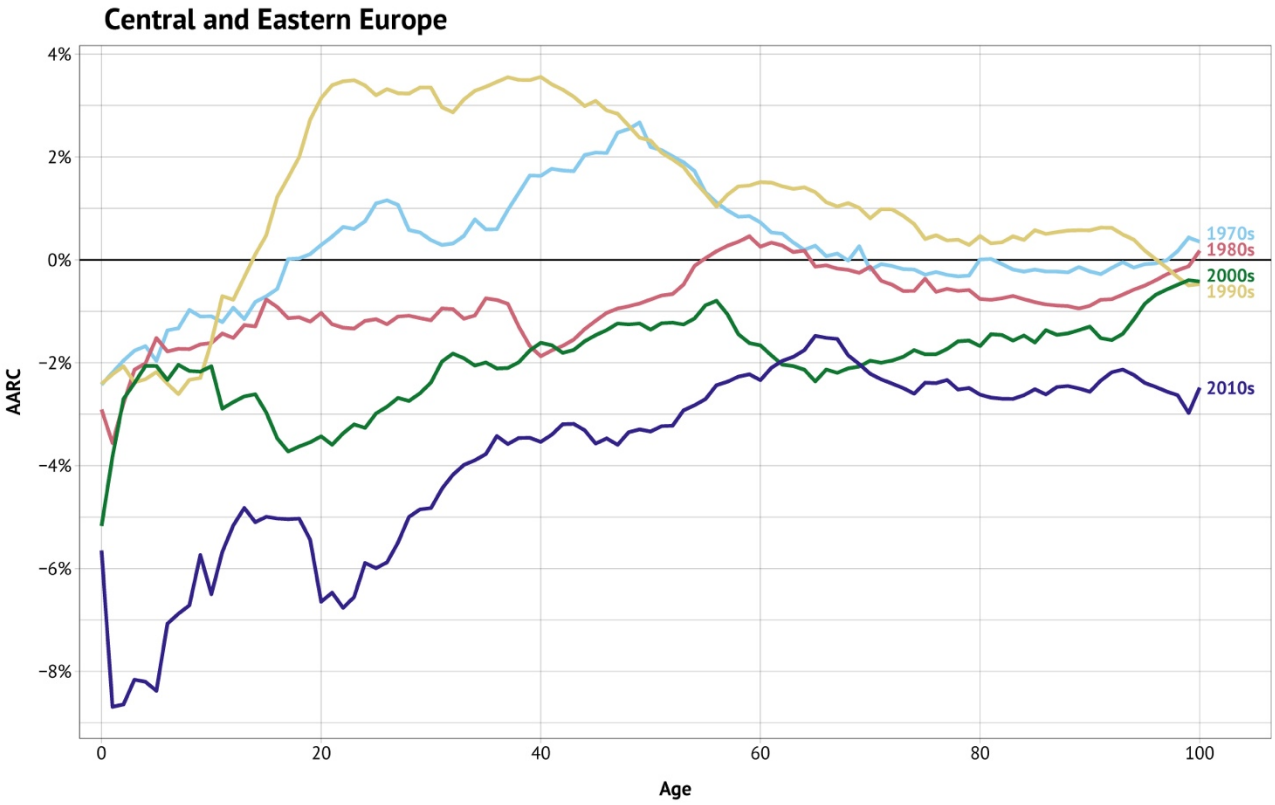

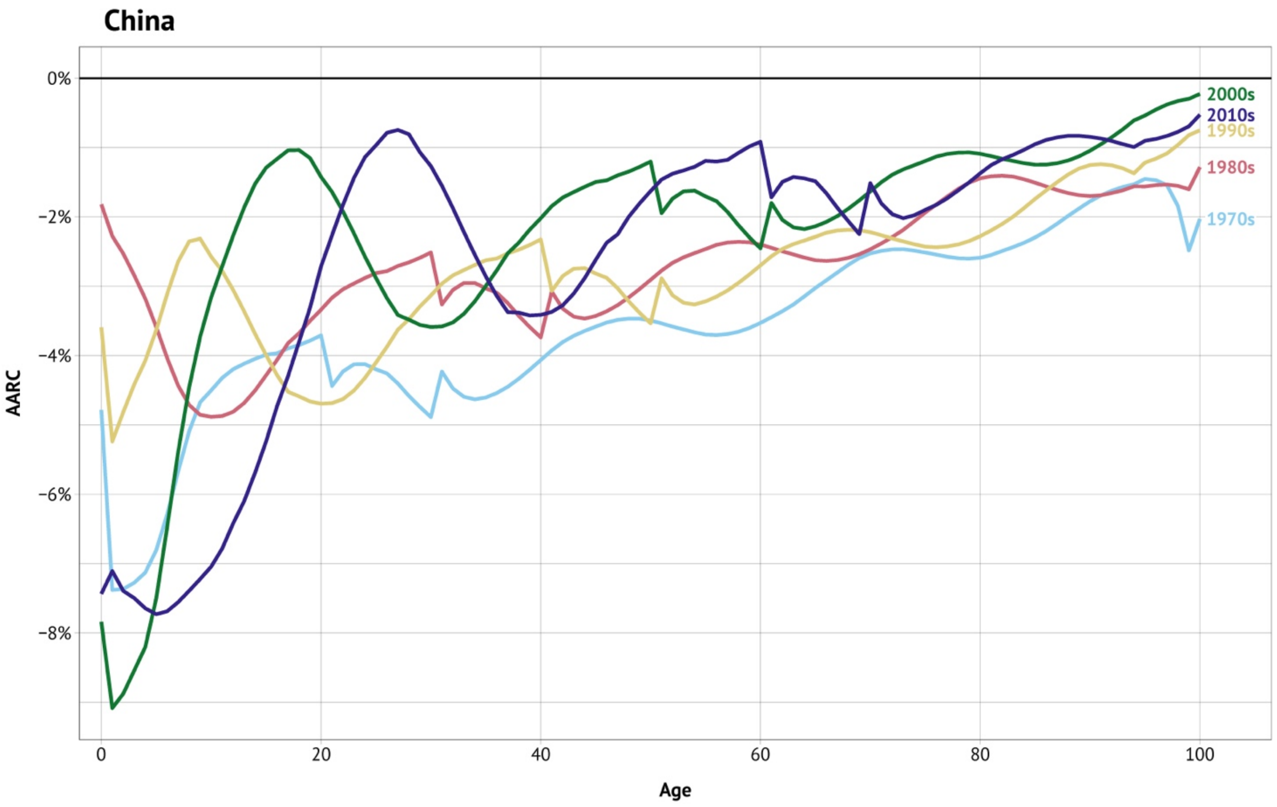
China
AARC
0%
-2%
-4%
-6%
-8%
0
20
40
60
80
100
Age
2000s
2010s
1990s
1980s
1970s

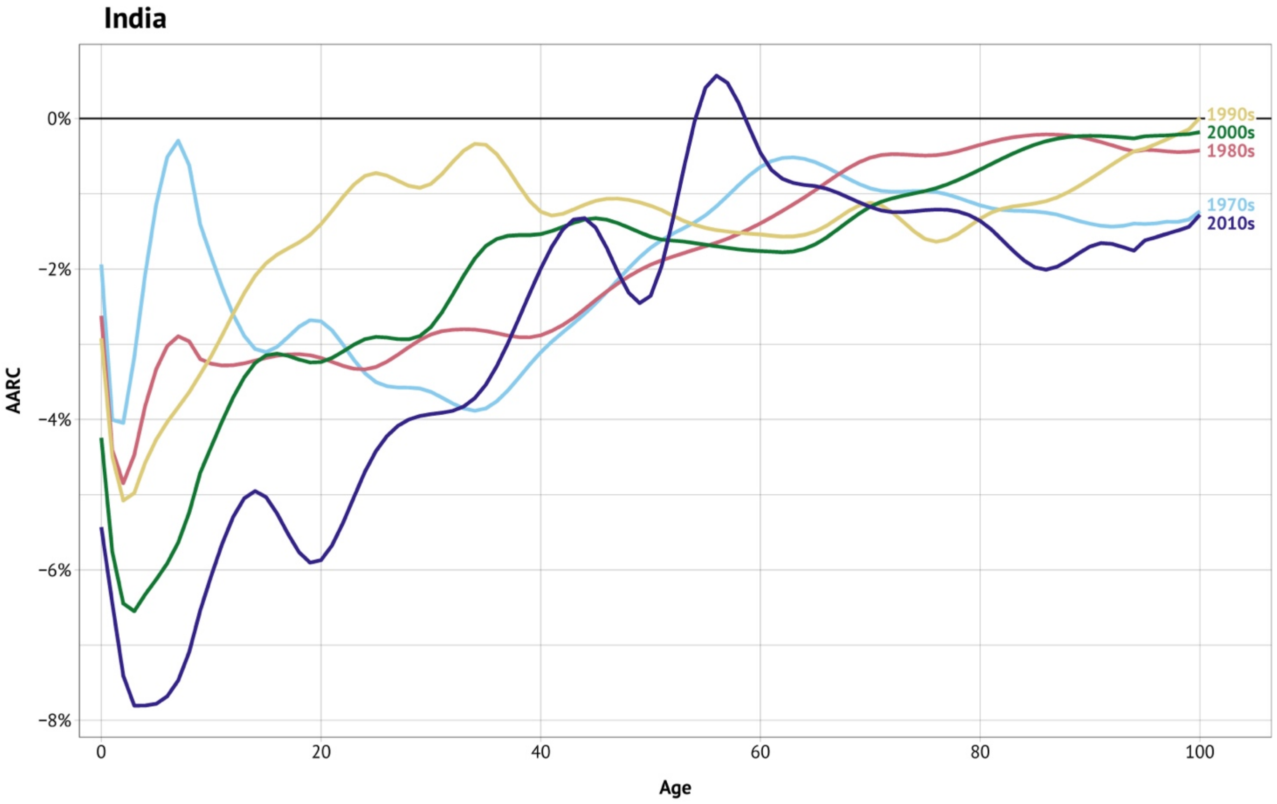
India
AARC
0%
-2%
-4%
-6%
-8%
0
20
40
60
80
100
Age
1990s
2000s
1980s
1970s
2010s

Latin America and Caribbean

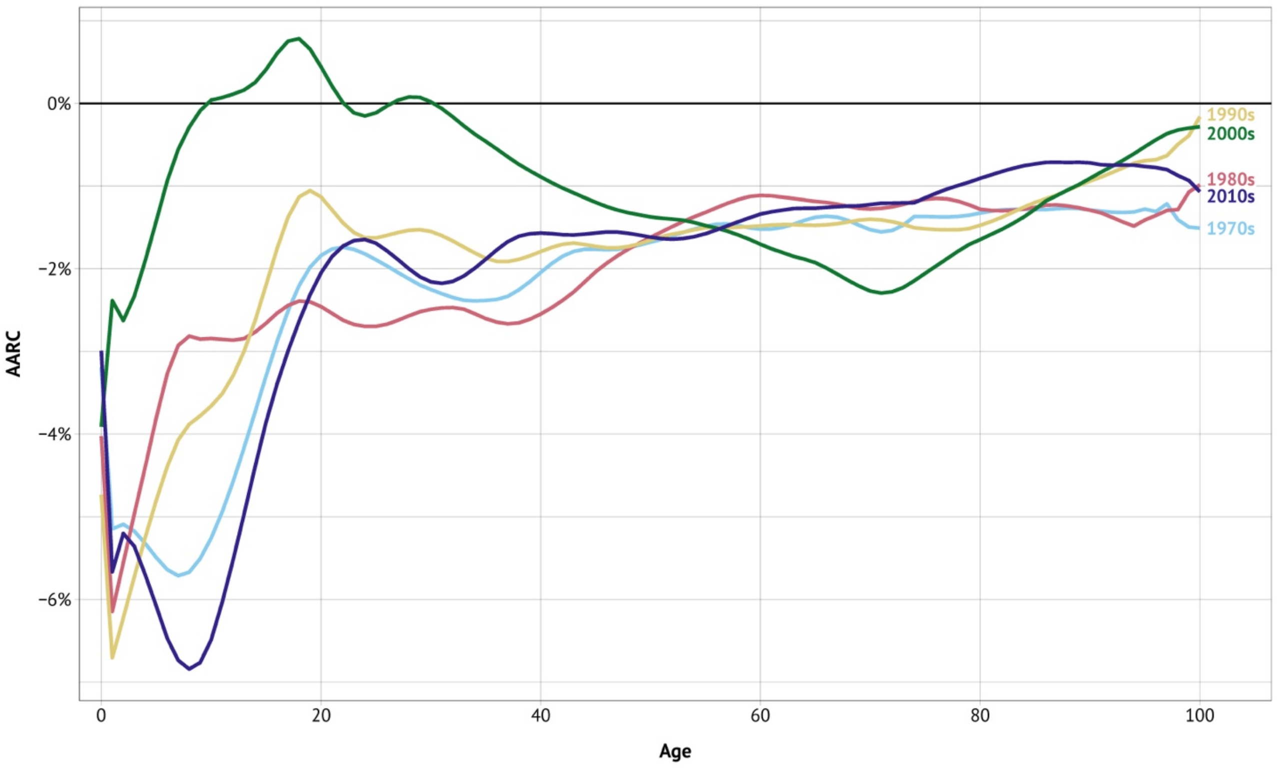

Middle East and North Africa

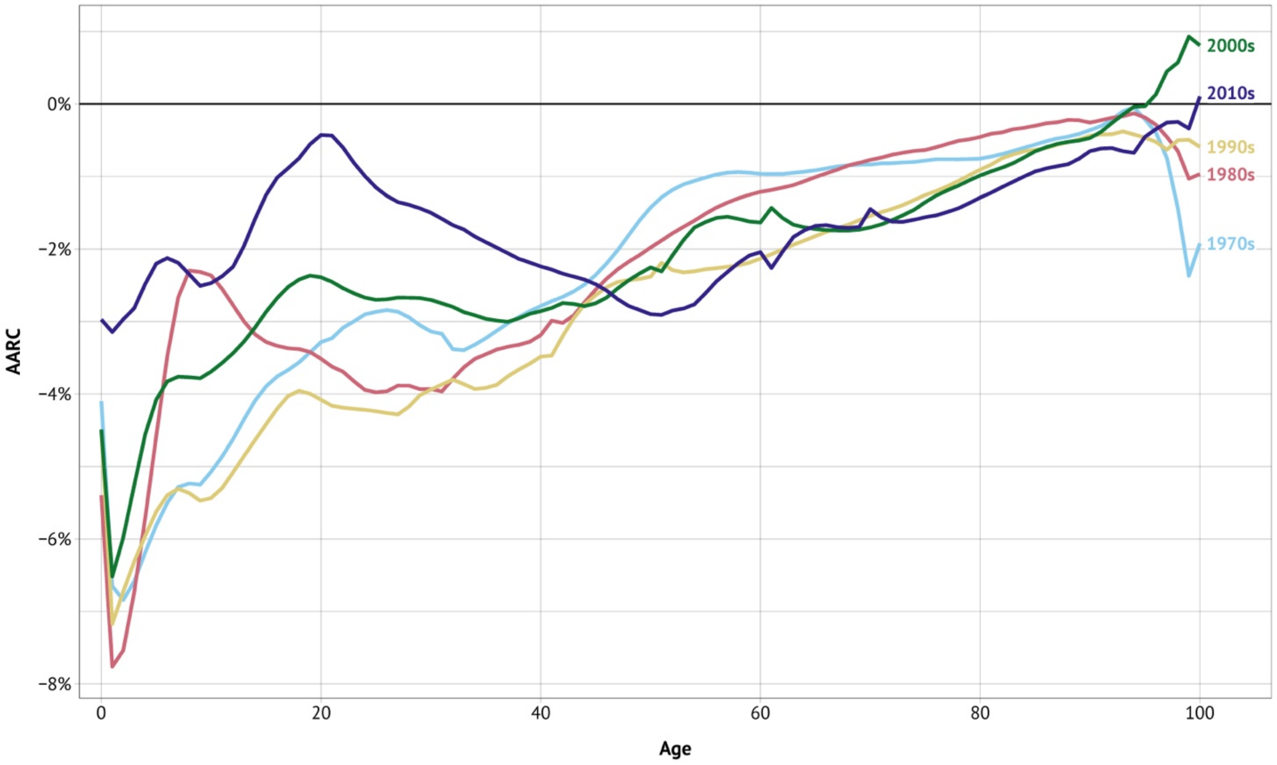

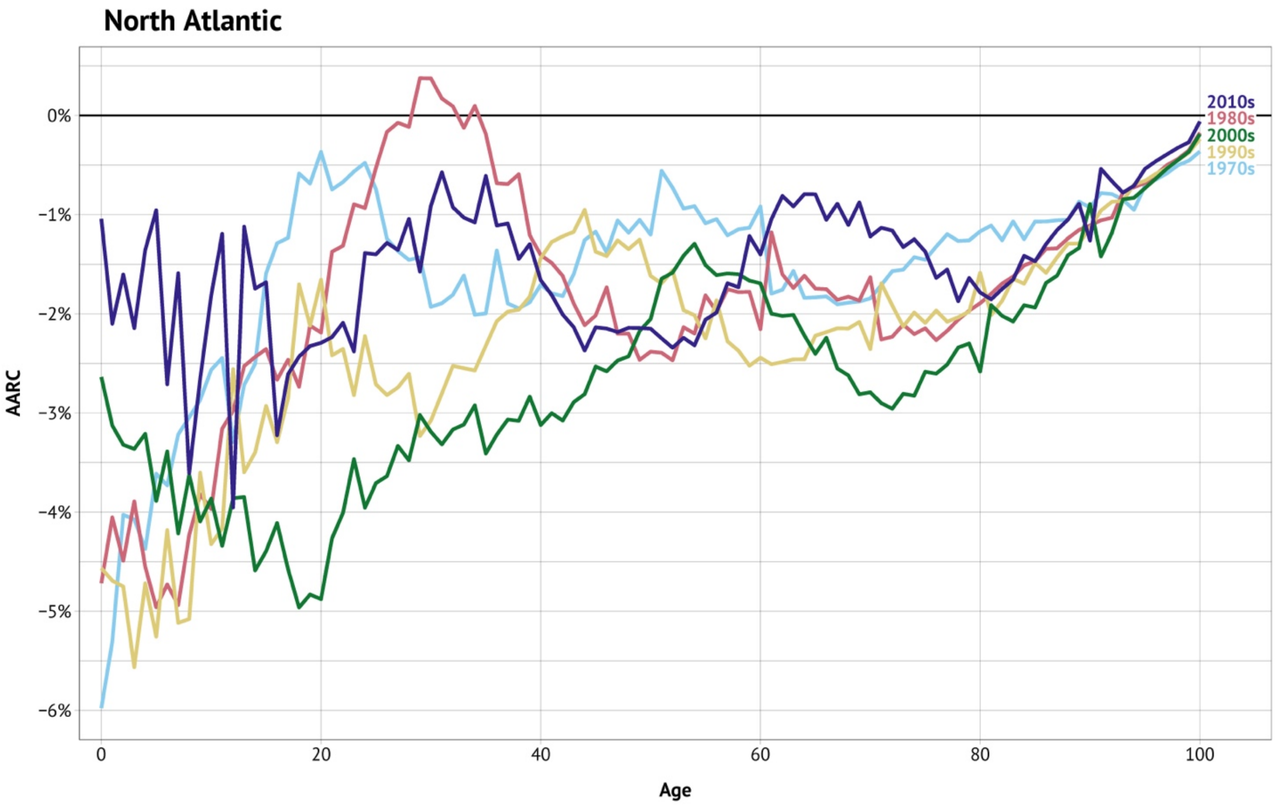
North Atlantic
0%
-1%
-2%
-3%
-4%
-5%
-6%
AARC
0
20
40
60
80
100
Age
2010s
1980s
2000s
1990s
1970s

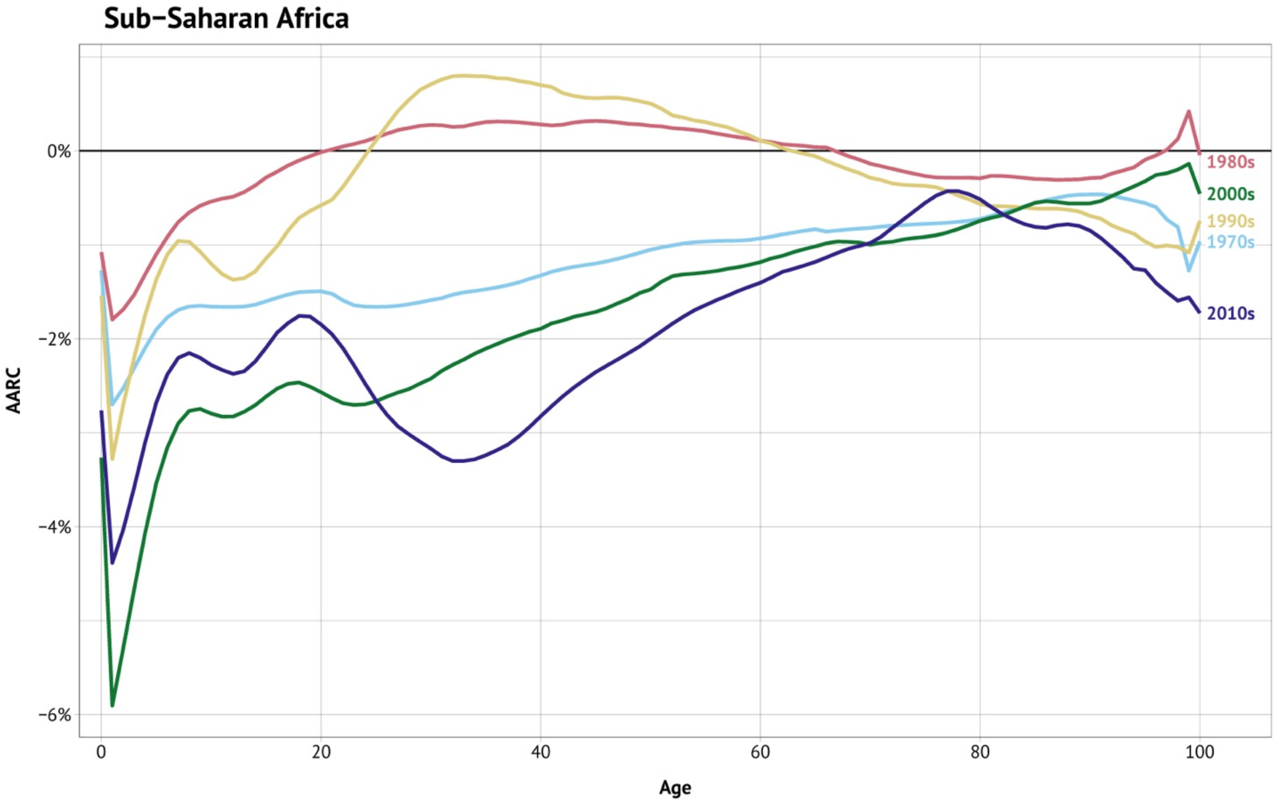
Sub−Saharan Africa
0%
−2%
−4%
−6%
AARC
0
20
40
60
80
100
Age
1980s
2000s
1990s
1970s
2010s

United States

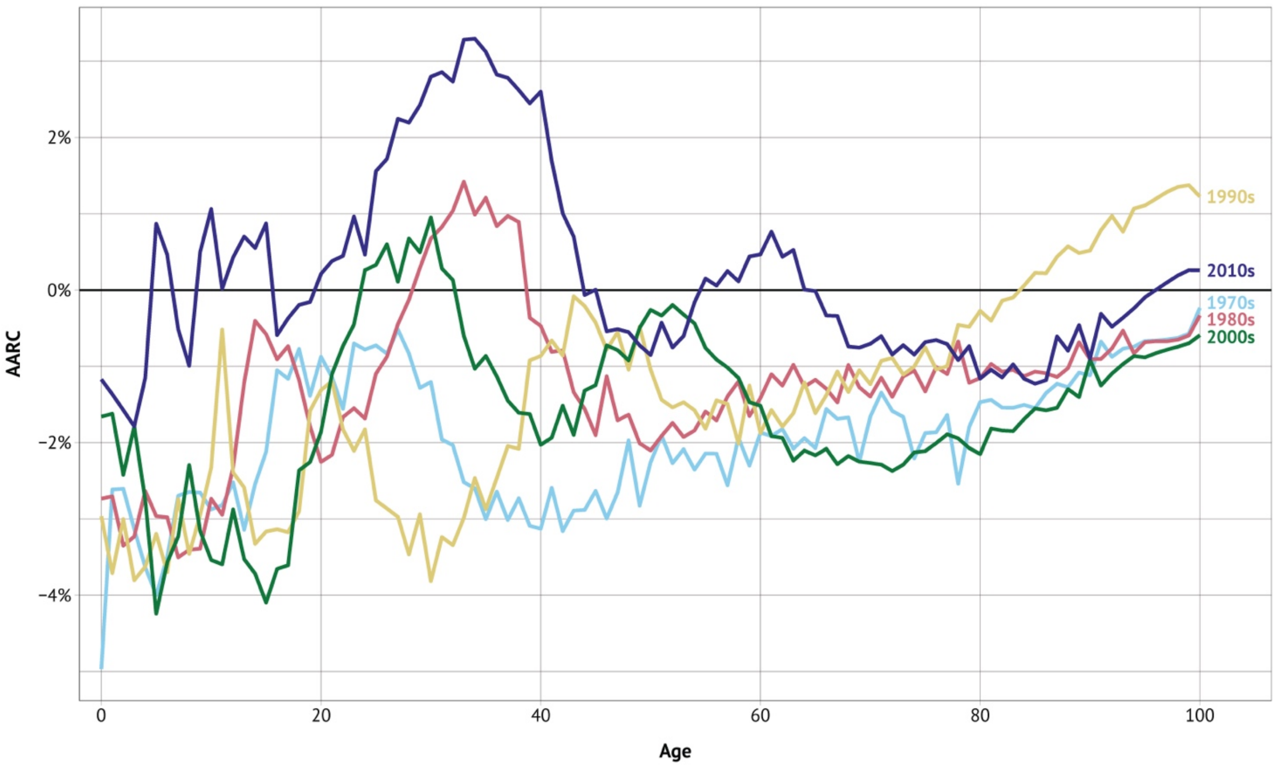

Western Pacific and Southeast Asia

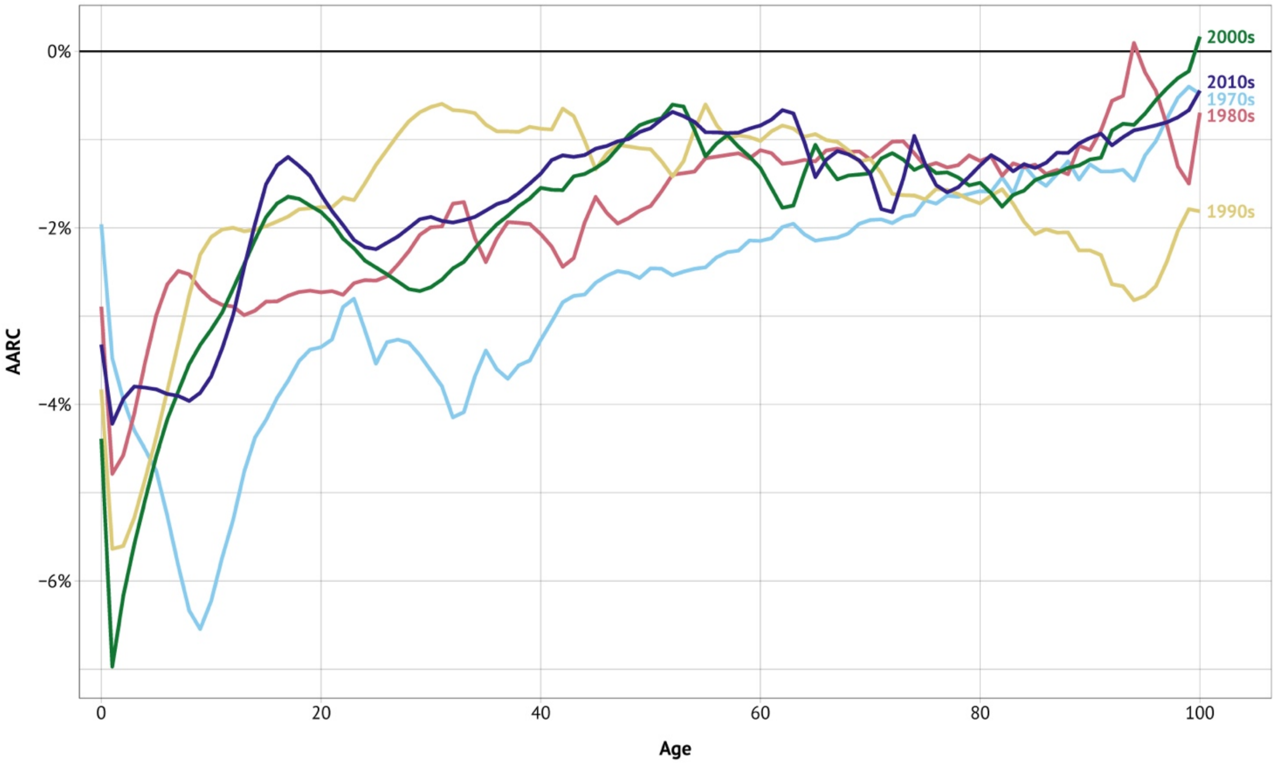

Figure A2. Global average annual rate of change in all-cause mortality by decade, age, and sex.

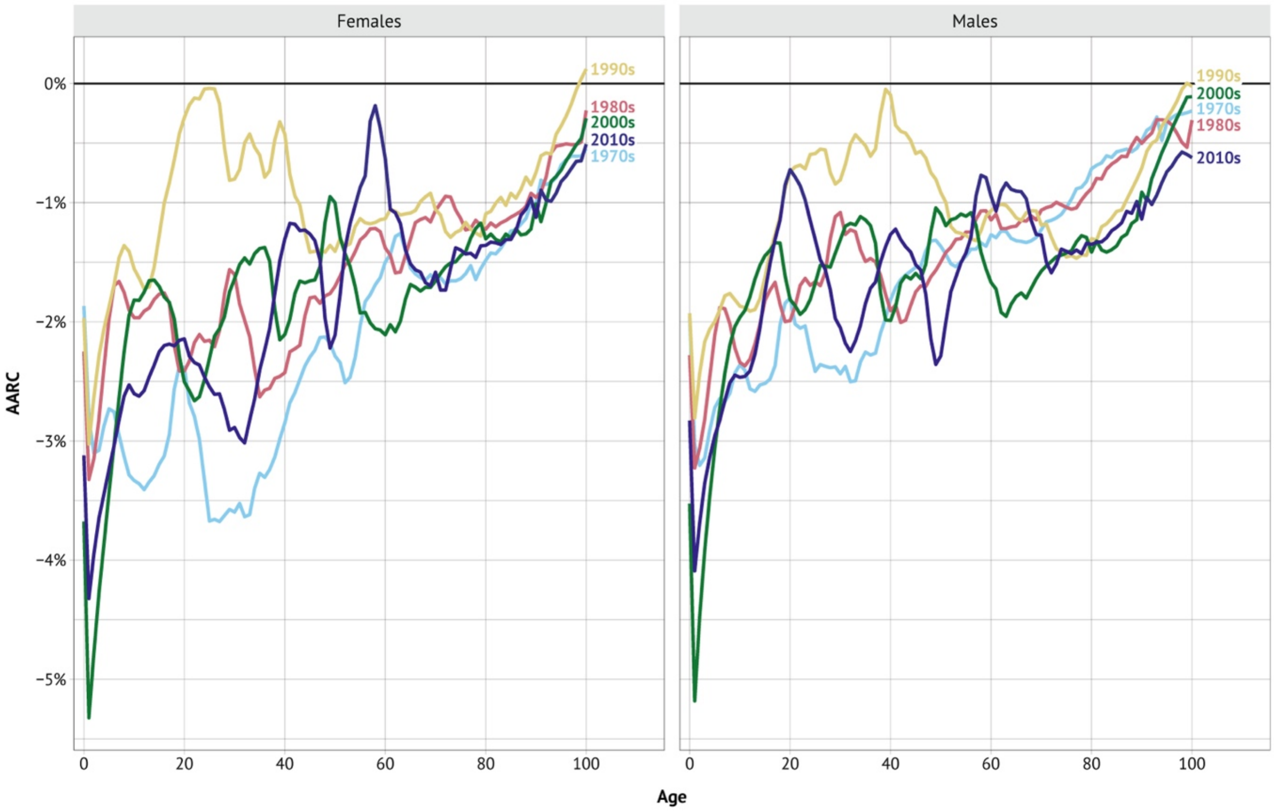

Figure A3. Average annual rate of change (AARC) by sex, age group, decade, and region.

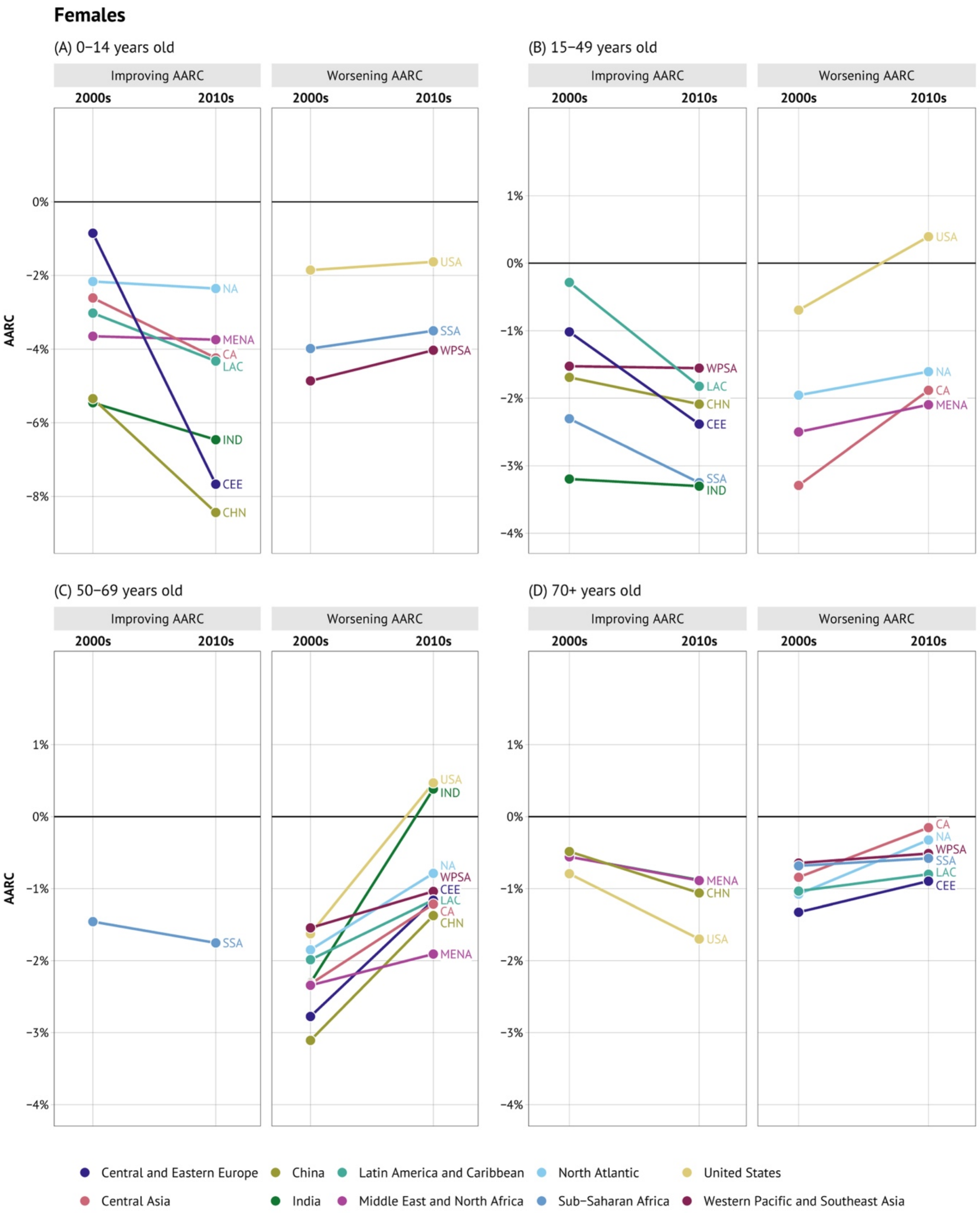

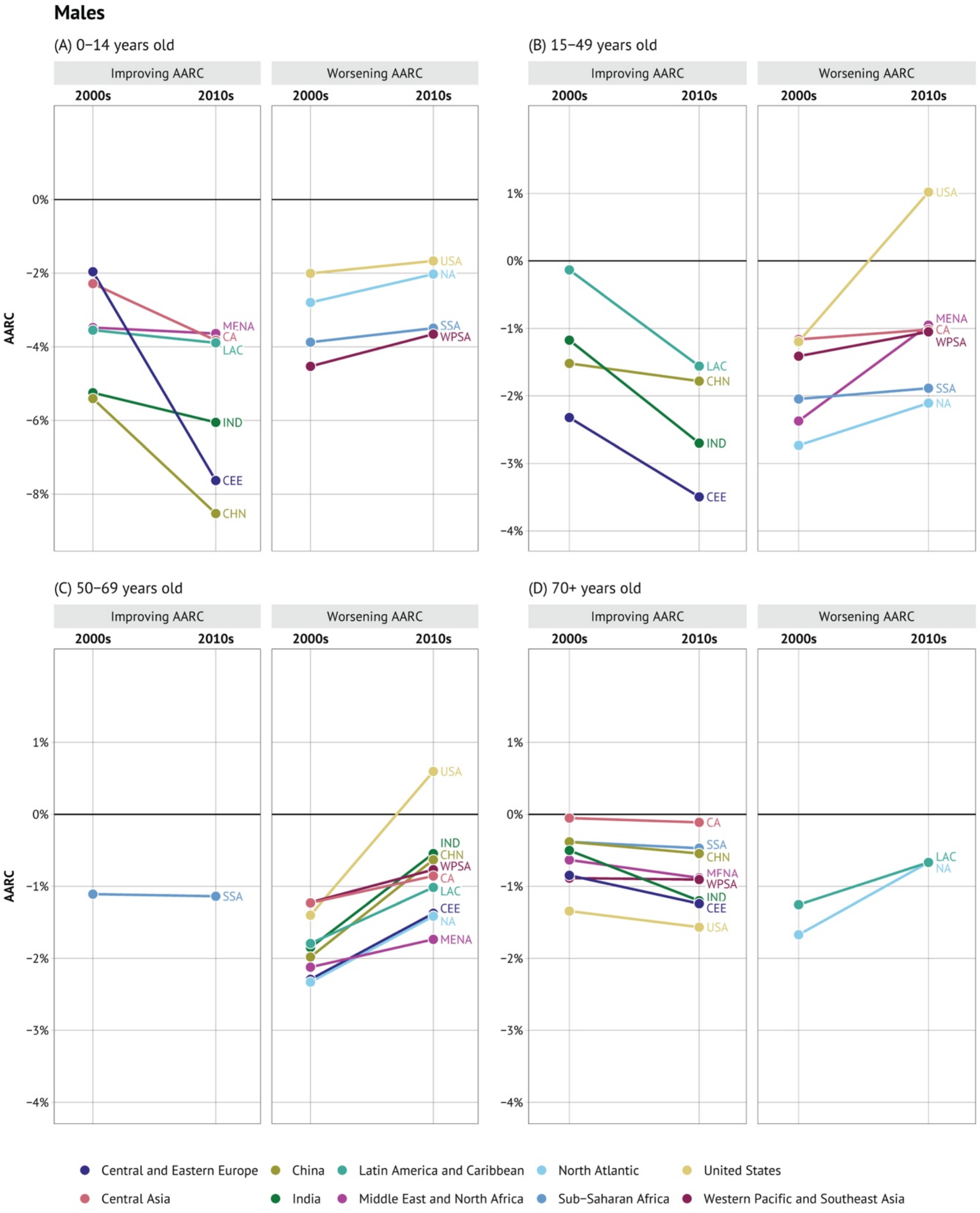
Males
(A) 0–14 years old
(B) 15–49 years old
(C) 50–69 years old
(D) 70+ years old
Improving AARC
Worsening AARC
2000s
2010s
AARC
Central and Eastern Europe
Central Asia
China
India
Latin America and Caribbean
Middle East and North Africa
North Atlantic
Sub-Saharan Africa
United States
Western Pacific and Southeast Asia

Figure A4. Average annual rate of change (AARC) by age group and region, 2010-2014 and 2015-2019.

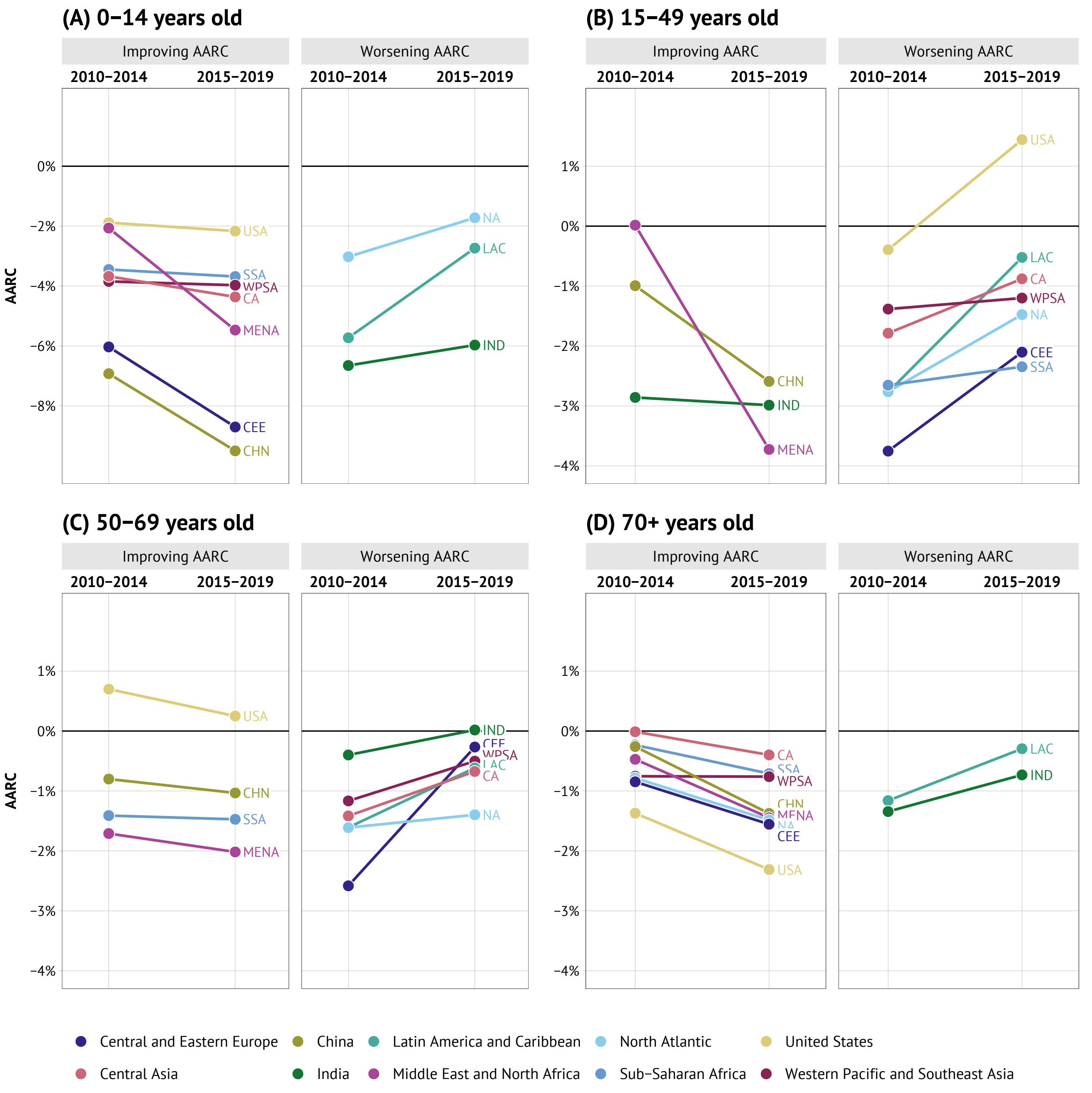

Figure A5. Decomposition of decadal changes in the crude death rate per 1000 population per year into component (changes in population structure, changes in age-specific mortality rates) contributions for the world and by region, 1970-2050.

Note: 1970s = 1970-1980; 1980s = 1980-1990; 1990s = 1990-2000, 2000s = 2000-2010; 2010s = 2010-2019; 2020s = 2019-2030; 2030s = 2030-2040; 2040s = 2040-2050 (2019 is substituted for 2020 to avoid COVID-19 impacts

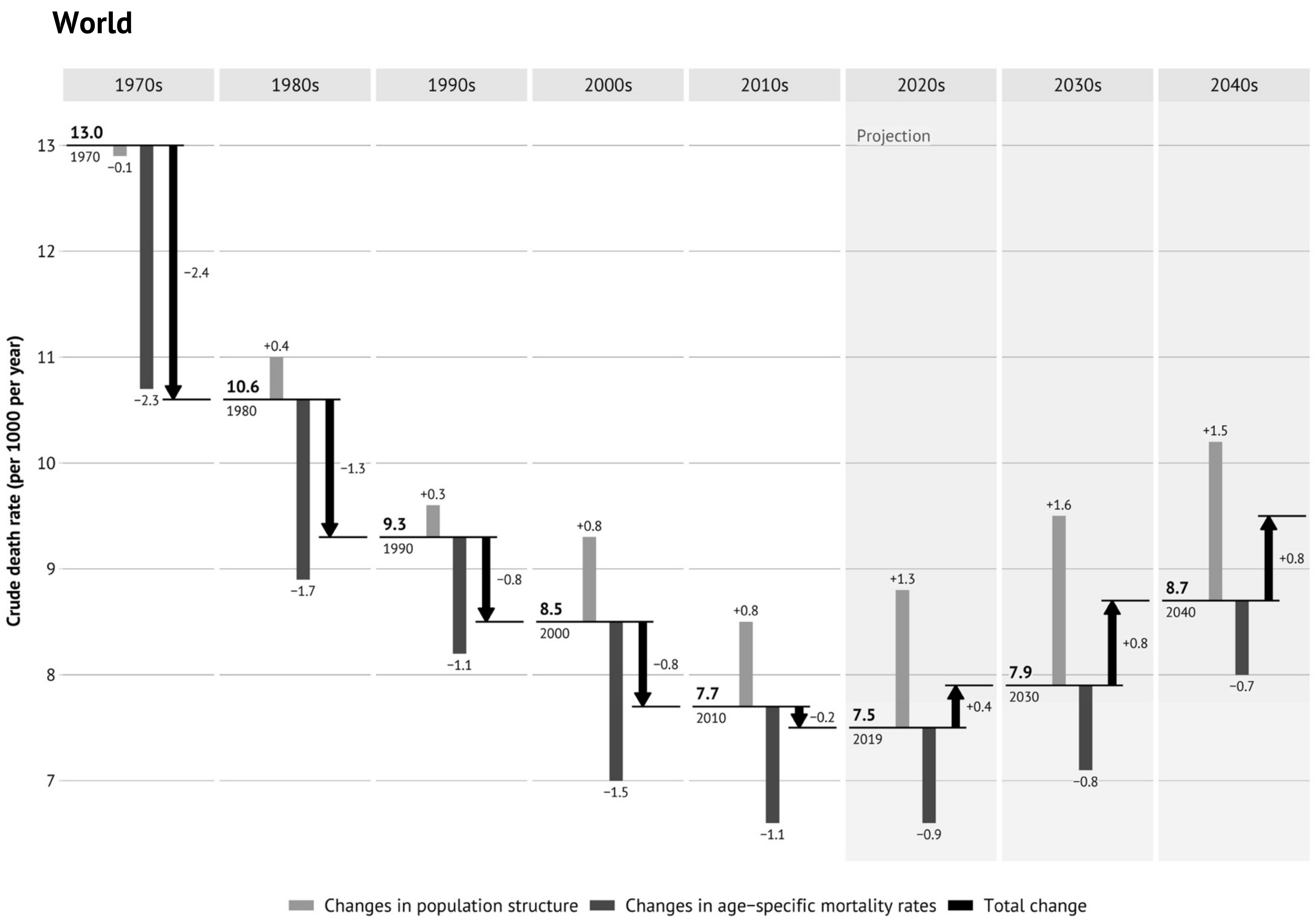

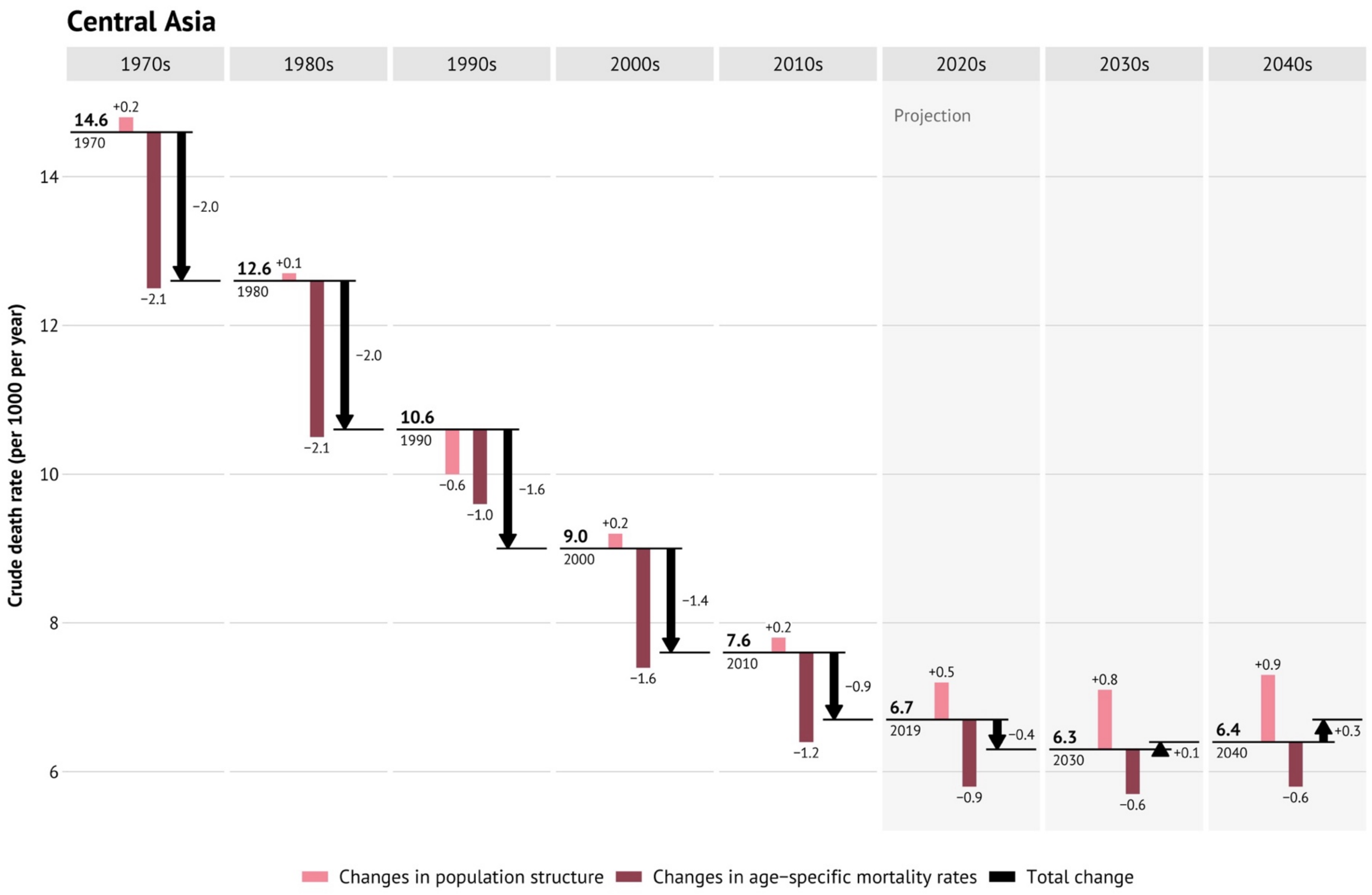
Central Asia
1970s
1980s
1990s
2000s
2010s
2020s
2030s
2040s
Projection
Crude death rate (per 1000 per year)
14.6
1970
+0.2
−2.1
−2.0
12.6
1980
+0.1
−2.1
−2.0
10.6
1990
−0.6
−1.0
−1.6
9.0
2000
+0.2
−1.6
−1.4
7.6
2010
+0.2
−1.2
−0.9
6.7
2019
+0.5
−0.9
−0.4
6.3
2030
+0.8
−0.6
+0.1
6.4
2040
+0.9
−0.6
+0.3
Changes in population structure
Changes in age−specific mortality rates
Total change

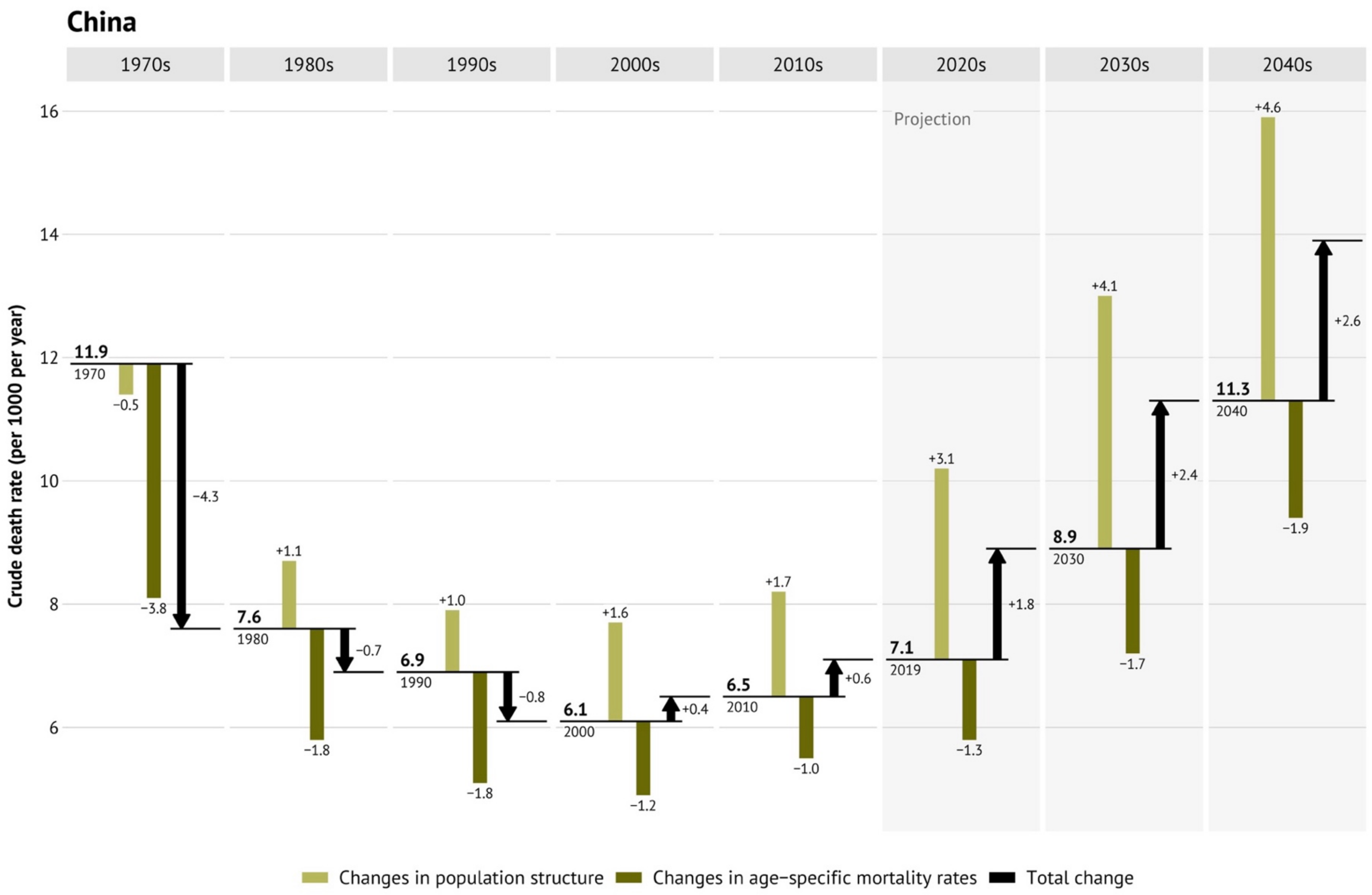
China
1970s
1980s
1990s
2000s
2010s
2020s
2030s
2040s
Projection
Crude death rate (per 1000 per year)
16
14
12
10
8
6
11.9
1970
−0.5
−3.8
−4.3
7.6
1980
+1.1
−1.8
−0.7
6.9
1990
+1.0
−1.8
−0.8
6.1
2000
+1.6
−1.2
+0.4
6.5
2010
+1.7
−1.0
+0.6
7.1
2019
+3.1
−1.3
+1.8
8.9
2030
+4.1
−1.7
+2.4
11.3
2040
+4.6
−1.9
+2.6
Changes in population structure
Changes in age−specific mortality rates
Total change

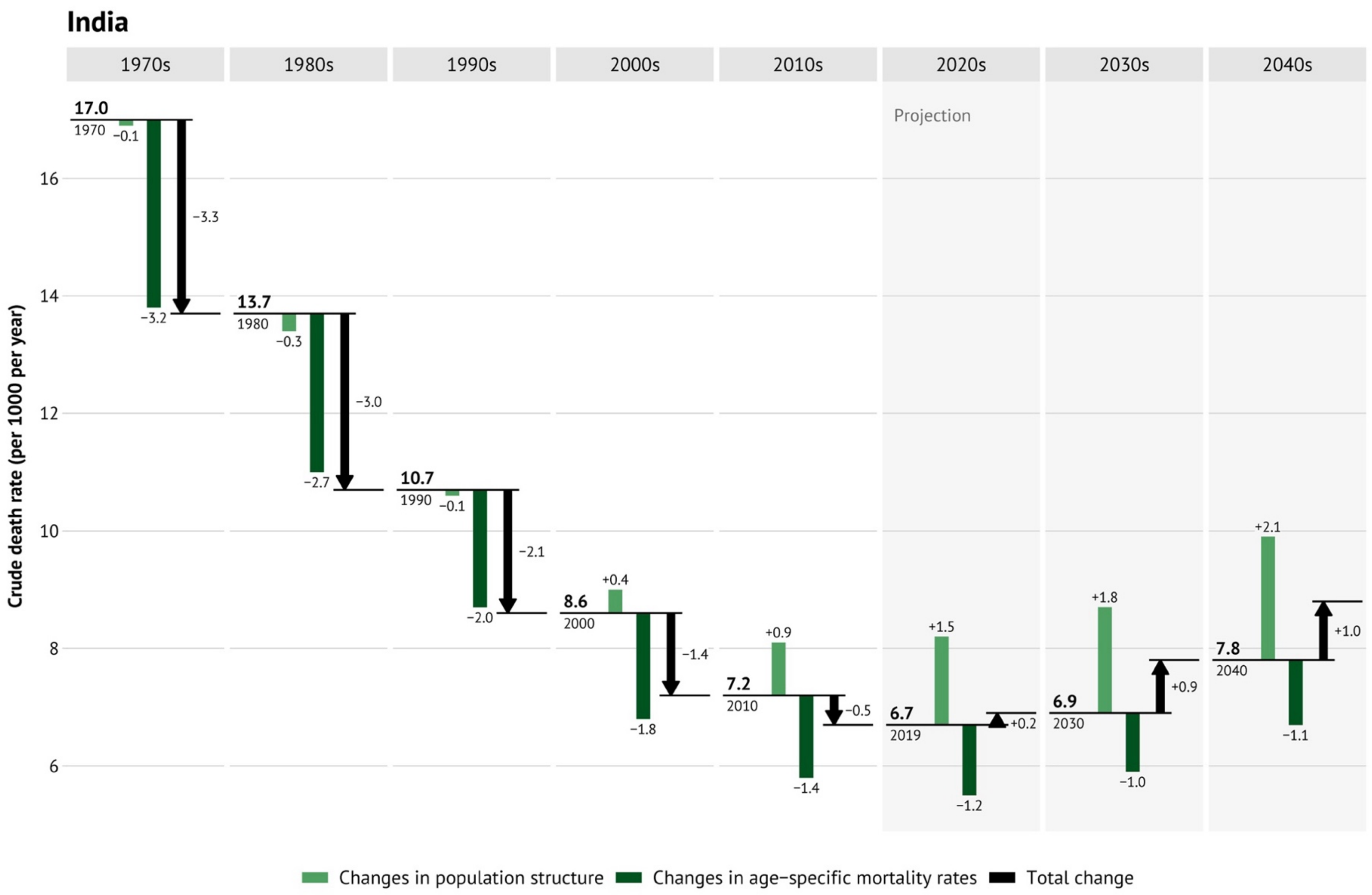
India
1970s
1980s
1990s
2000s
2010s
2020s
2030s
2040s
Projection
Crude death rate (per 1000 per year)
17.0
1970
−0.1
−3.2
−3.3
13.7
1980
−0.3
−2.7
−3.0
10.7
1990
−0.1
−2.0
−2.1
8.6
2000
+0.4
−1.8
−1.4
7.2
2010
+0.9
−1.4
−0.5
6.7
2019
+1.5
−1.2
+0.2
6.9
2030
+1.8
−1.0
+0.9
7.8
2040
+2.1
−1.1
+1.0
Changes in population structure
Changes in age−specific mortality rates
Total change

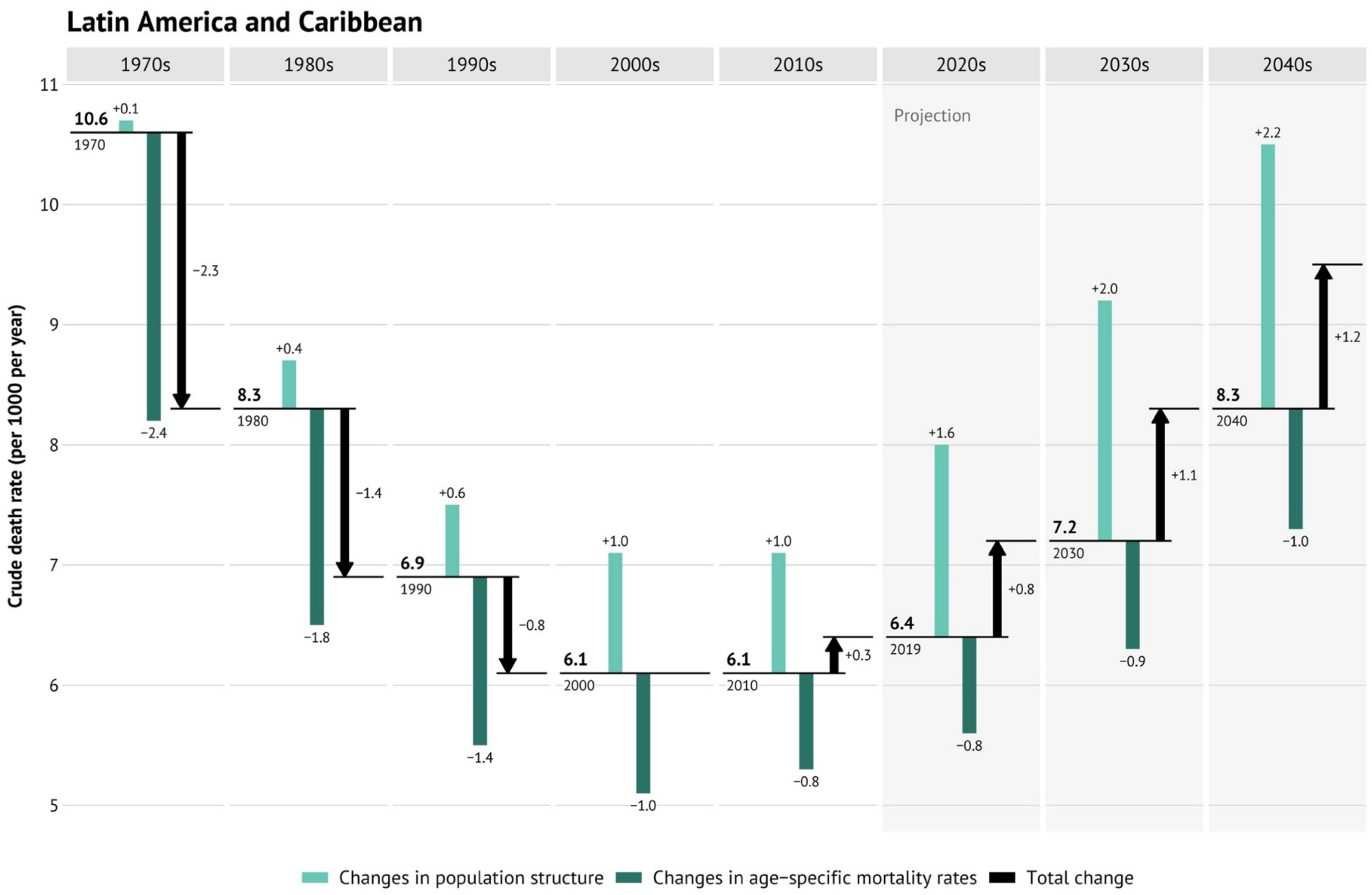
Latin America and Caribbean
1970s
1980s
1990s
2000s
2010s
2020s
2030s
2040s
Projection
Crude death rate (per 1000 per year)
11
10
9
8
7
6
5
10.6
1970
+0.1
−2.4
−2.3
8.3
1980
+0.4
−1.8
−1.4
6.9
1990
+0.6
−1.4
−0.8
6.1
2000
+1.0
−1.0
6.1
2010
+1.0
−0.8
+0.3
6.4
2019
+1.6
−0.8
+0.8
7.2
2030
+2.0
−0.9
+1.1
8.3
2040
+2.2
−1.0
+1.2
Changes in population structure
Changes in age−specific mortality rates
Total change

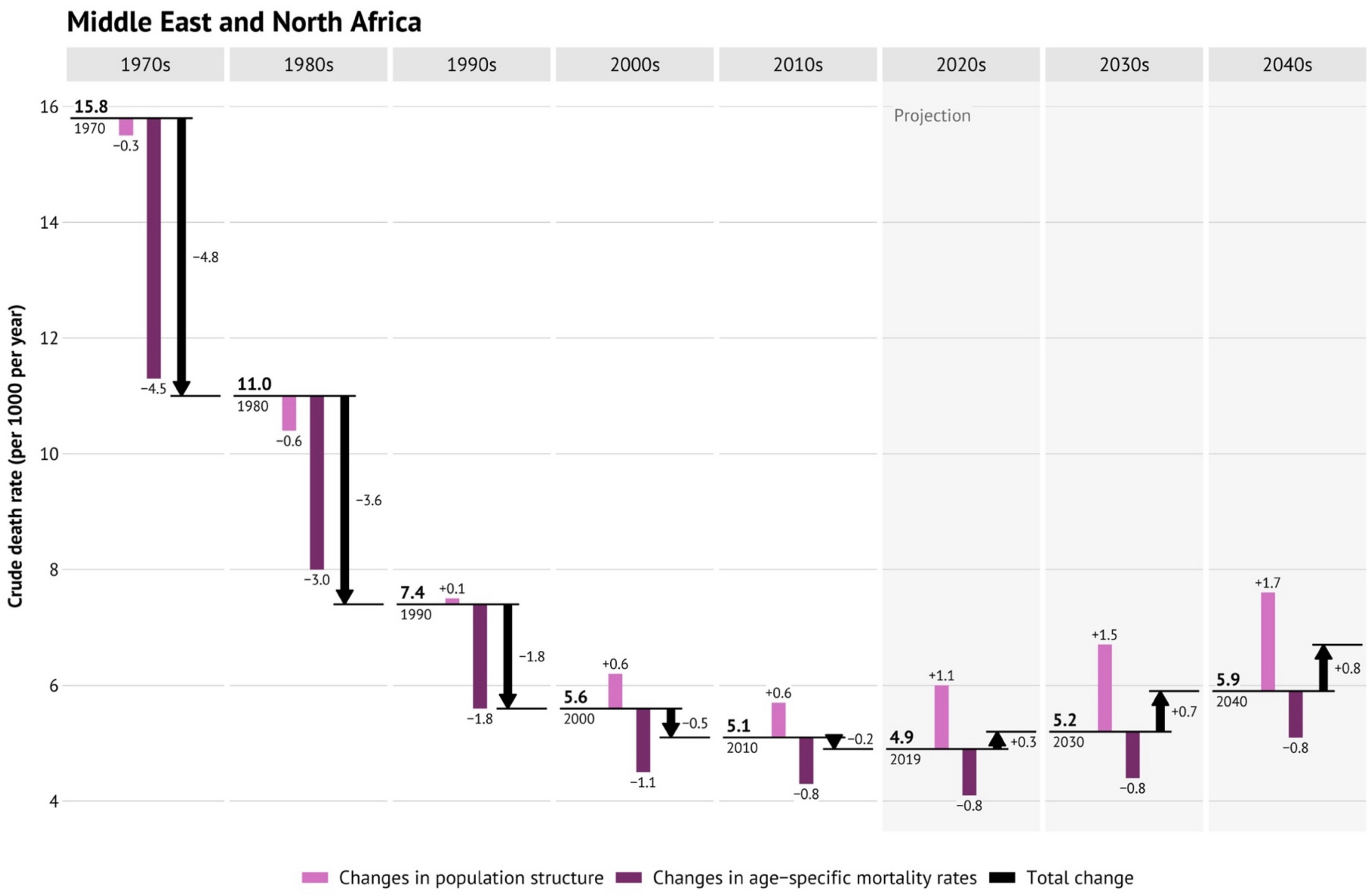

Middle East and North Africa
1970s
1980s
1990s
2000s
2010s
2020s
2030s
2040s
Projection
Crude death rate (per 1000 per year)
16
14
12
10
8
6
4
15.8
1970
−0.3
−4.5
−4.8
11.0
1980
−0.6
−3.0
−3.6
7.4
1990
+0.1
−1.8
−1.8
5.6
2000
+0.6
−1.1
−0.5
5.1
2010
+0.6
−0.8
−0.2
4.9
2019
+1.1
−0.8
+0.3
5.2
2030
+1.5
−0.8
+0.7
5.9
2040
+1.7
−0.8
+0.8
Changes in population structure
Changes in age−specific mortality rates
Total change

North Atlantic

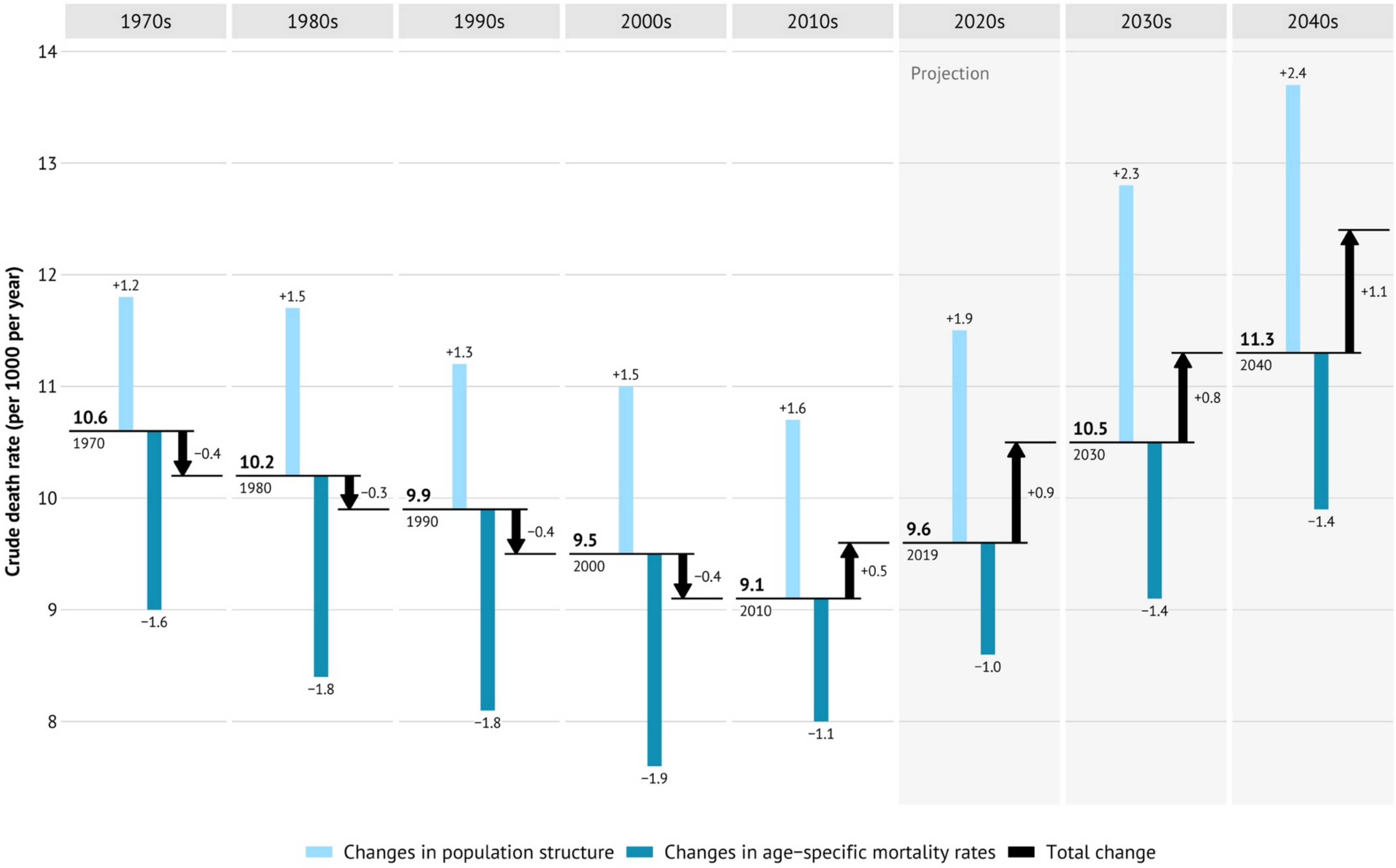

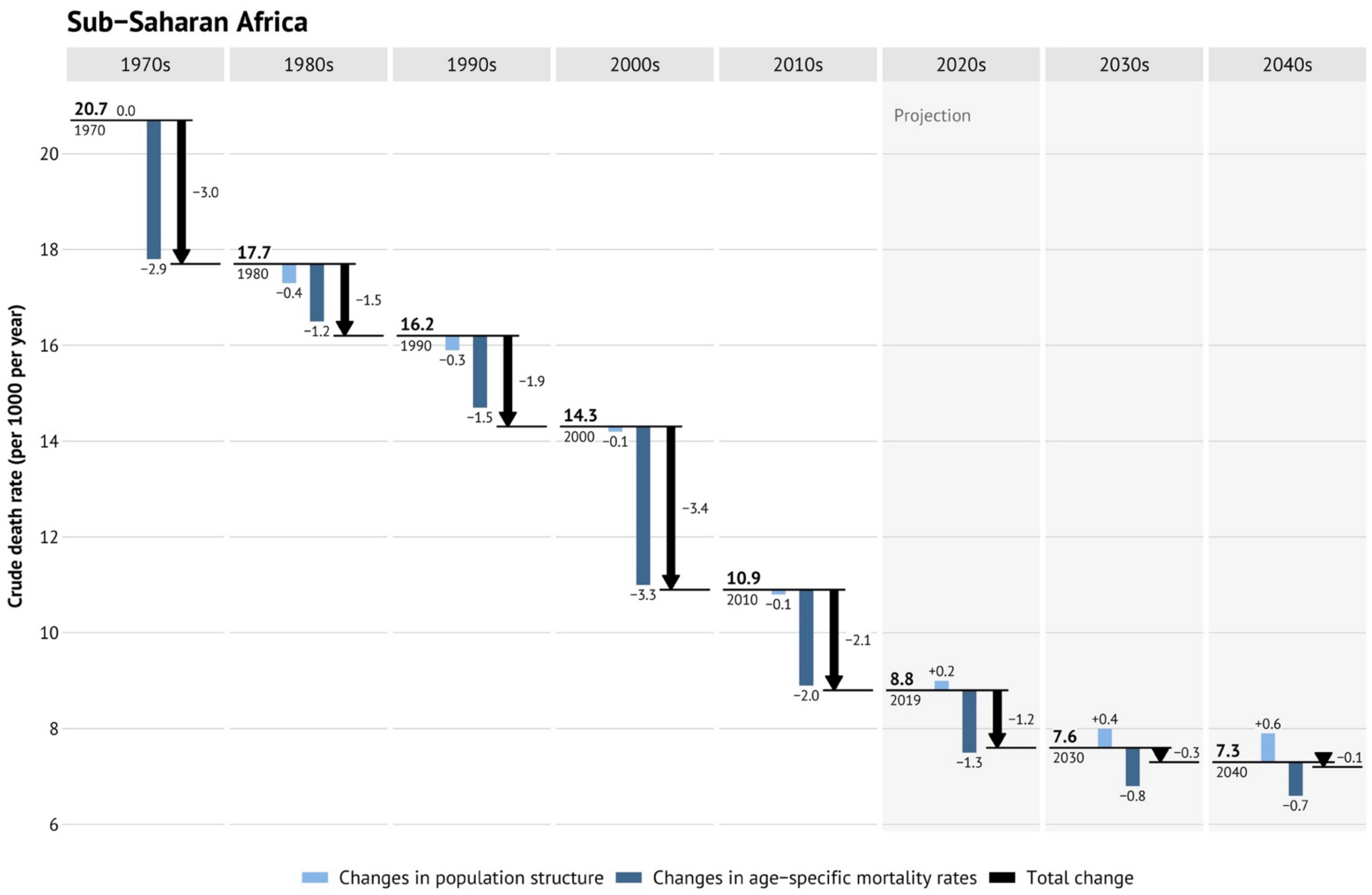

Sub–Saharan Africa
1970s
1980s
1990s
2000s
2010s
2020s
2030s
2040s
Projection
Crude death rate (per 1000 per year)
20.7 0.0
1970
−3.0
−2.9
17.7
1980
−0.4
−1.5
−1.2
16.2
1990
−0.3
−1.9
−1.5
14.3
2000 −0.1
−3.4
−3.3
10.9
2010 −0.1
−2.1
−2.0
8.8 +0.2
2019
−1.2
−1.3
7.6 +0.4
2030
−0.3
−0.8
7.3 +0.6
2040
−0.1
−0.7
Changes in population structure
Changes in age−specific mortality rates
Total change

United States

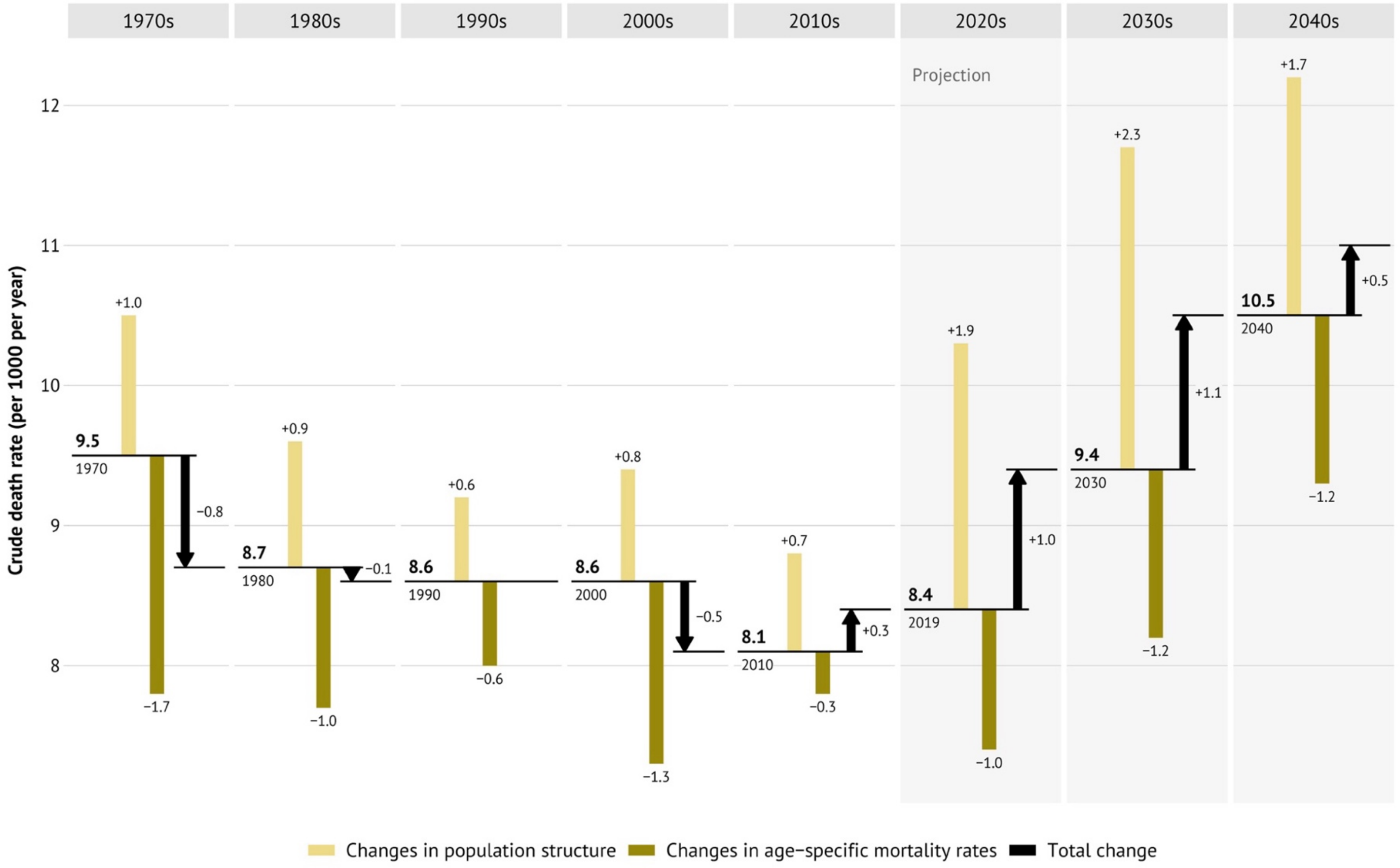

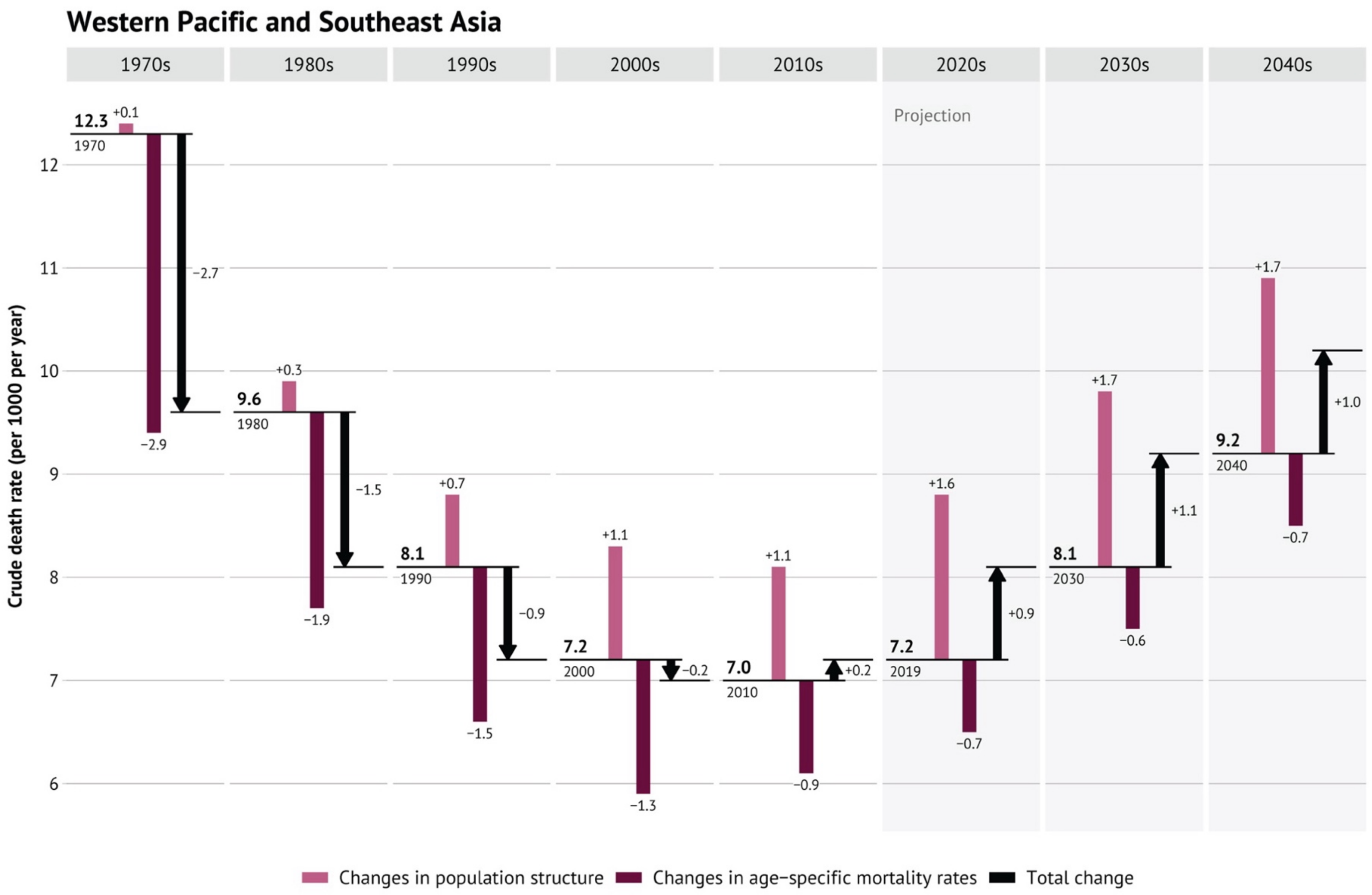

Western Pacific and Southeast Asia
1970s
1980s
1990s
2000s
2010s
2020s
2030s
2040s
Projection
Crude death rate (per 1000 per year)
12.3
1970
+0.1
-2.9
-2.7
9.6
1980
+0.3
-1.9
-1.5
8.1
1990
+0.7
-1.5
-0.9
7.2
2000
+1.1
-1.3
-0.2
7.0
2010
+1.1
-0.9
+0.2
7.2
2019
+1.6
-0.7
+0.9
8.1
2030
+1.7
-0.6
+1.1
9.2
2040
+1.7
-0.7
+1.0
Changes in population structure
Changes in age-specific mortality rates
Total change

Figure A6. Population pyramids for selected countries: Nigeria, India, United States, and Japan, 1970, 2019, and 2050.

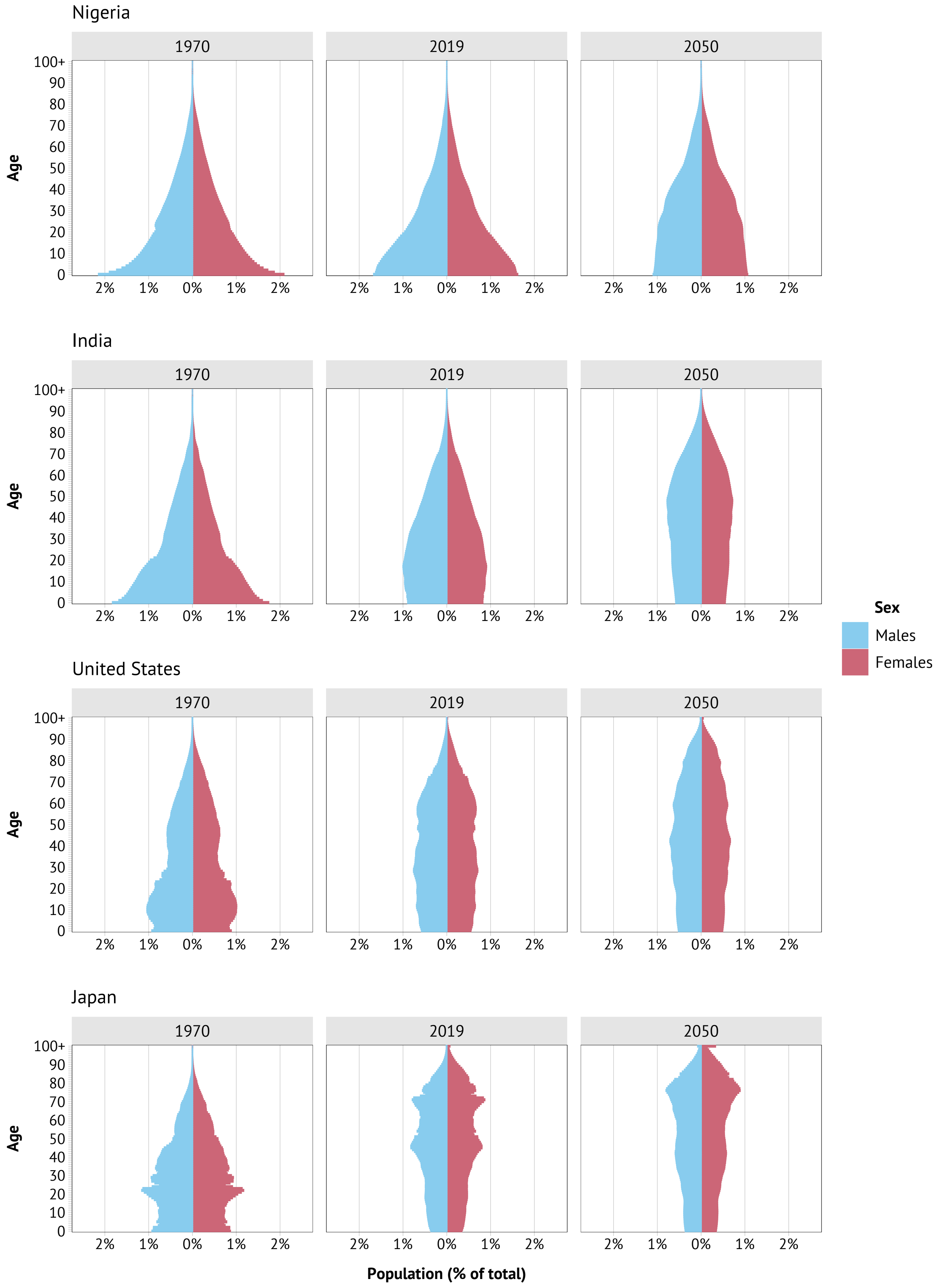

Table A5. Decomposition of decadal changes crude death rate (CDR) per 1000 per year into component (changes in population structure, changes in age-specific mortality rates) contributions by region, 1970-1950.

Note: Net contribution = 0% does not mean that the factor does not contribute at all, but rather it is cancelled out (or overpowered by) the pressure coming from the other factor.

	CDR (per 1000 per year)			Changes in population structure		Changes in age-specific mortality rates	
	First year	Last year	Difference	Per 1000 per year	Net contribution to difference	Per 1000 per year	Net contribution to difference
World							
1970s	13.0	10.6	-2.4	-0.13	6%	-2.26	94%
1980s	10.6	9.3	-1.3	+0.36	0%	-1.65	100%
1990s	9.3	8.5	-0.9	+0.26	0%	-1.14	100%
2000s	8.5	7.7	-0.7	+0.79	0%	-1.53	100%
2010s	7.7	7.5	-0.3	+0.81	0%	-1.07	100%
2020s	7.5	7.9	+0.4	+1.29	100%	-0.85	0%
2030s	7.9	8.7	+0.8	+1.56	100%	-0.76	0%
2040s	8.7	9.5	+0.8	+1.52	100%	-0.72	0%
2050s	9.5	10.2	+0.7	+1.41	100%	-0.69	0%
Central and Eastern Europe							
1970s	9.1	10.7	+1.6	+1.44	88%	+0.20	12%
1980s	10.7	11.1	+0.4	+1.06	100%	-0.67	0%
1990s	11.1	13.6	+2.5	+1.18	47%	+1.33	53%
2000s	13.6	13.3	-0.3	+2.06	0%	-2.36	100%
2010s	13.3	12.3	-1.1	+1.97	0%	-3.02	100%
2020s	12.3	13.2	+0.9	+2.27	100%	-1.38	0%
2030s	13.2	14.3	+1.1	+2.74	100%	-1.61	0%
2040s	14.3	14.7	+0.4	+2.16	100%	-1.72	0%
2050s	14.7	14.9	+0.2	+1.82	100%	-1.61	0%
Central Asia							
1970s	14.6	12.6	-2.0	+0.17	0%	-2.13	100%
1980s	12.6	10.6	-2.0	+0.07	0%	-2.08	100%
1990s	10.6	9.0	-1.7	-0.61	37%	-1.04	63%
2000s	9.0	7.6	-1.4	+0.16	0%	-1.55	100%
2010s	7.6	6.7	-0.9	+0.24	0%	-1.15	100%
2020s	6.7	6.2	-0.4	+0.47	0%	-0.88	100%
2030s	6.2	6.4	+0.2	+0.80	100%	-0.61	0%
2040s	6.4	6.7	+0.3	+0.85	100%	-0.57	0%
2050s	6.7	7.2	+0.5	+1.03	100%	-0.56	0%
China							
1970s	11.9	7.6	-4.3	-0.45	11%	-3.83	89%
1980s	7.6	6.9	-0.7	+1.06	0%	-1.78	100%
1990s	6.9	6.1	-0.8	+0.98	0%	-1.76	100%
2000s	6.1	6.5	+0.4	+1.61	100%	-1.23	0%

	CDR (per 1000 per year)			Changes in population structure		Changes in age-specific mortality rates	
	First year	Last year	Difference	Per 1000 per year	Net contribution to difference	Per 1000 per year	Net contribution to difference
2010s	6.5	7.1	+0.6	+1.65	100%	-1.04	0%
2020s	7.1	8.9	+1.8	+3.08	100%	-1.31	0%
2030s	8.9	11.3	+2.4	+4.12	100%	-1.70	0%
2040s	11.3	13.9	+2.6	+4.55	100%	-1.94	0%
2050s	13.9	16.6	+2.6	+4.80	100%	-2.15	0%
India							
1970s	17.0	13.7	-3.3	-0.09	3%	-3.18	97%
1980s	13.7	10.7	-3.0	-0.26	9%	-2.75	91%
1990s	10.7	8.6	-2.1	-0.07	3%	-2.03	97%
2000s	8.6	7.2	-1.5	+0.35	0%	-1.81	100%
2010s	7.2	6.7	-0.5	+0.87	0%	-1.37	100%
2020s	6.7	7.0	+0.3	+1.51	100%	-1.23	0%
2030s	7.0	7.8	+0.8	+1.83	100%	-1.00	0%
2040s	7.8	8.8	+1.0	+2.09	100%	-1.06	0%
2050s	8.8	10.0	+1.2	+2.36	100%	-1.20	0%
Latin America and Caribbean							
1970s	10.6	8.3	-2.3	+0.09	0%	-2.42	100%
1980s	8.3	6.9	-1.4	+0.40	0%	-1.82	100%
1990s	6.9	6.1	-0.8	+0.62	0%	-1.38	100%
2000s	6.1	6.1	0.0	+1.01	100%	-0.98	0%
2010s	6.1	6.4	+0.2	+1.03	100%	-0.81	0%
2020s	6.4	7.2	+0.8	+1.57	100%	-0.77	0%
2030s	7.2	8.3	+1.1	+1.97	100%	-0.87	0%
2040s	8.3	9.5	+1.2	+2.18	100%	-0.97	0%
2050s	9.5	10.7	+1.2	+2.25	100%	-1.06	0%
Middle East and North Africa							
1970s	15.8	11.0	-4.8	-0.34	7%	-4.47	93%
1980s	11.0	7.4	-3.6	-0.59	16%	-3.01	84%
1990s	7.4	5.6	-1.8	+0.07	0%	-1.85	100%
2000s	5.6	5.1	-0.5	+0.64	0%	-1.15	100%
2010s	5.1	4.8	-0.2	+0.57	0%	-0.80	100%
2020s	4.8	5.2	+0.3	+1.07	100%	-0.76	0%
2030s	5.2	5.9	+0.7	+1.49	100%	-0.76	0%
2040s	5.9	6.7	+0.8	+1.65	100%	-0.82	0%
2050s	6.7	7.7	+1.0	+1.83	100%	-0.85	0%
North Atlantic							
1970s	10.6	10.2	-0.3	+1.22	0%	-1.55	100%
1980s	10.2	9.9	-0.3	+1.52	0%	-1.81	100%
1990s	9.9	9.5	-0.4	+1.34	0%	-1.77	100%
2000s	9.5	9.1	-0.4	+1.47	0%	-1.89	100%
2010s	9.1	9.6	+0.5	+1.57	100%	-1.08	0%

	CDR (per 1000 per year)			Changes in population structure		Changes in age-specific mortality rates	
	First year	Last year	Difference	Per 1000 per year	Net contribution to difference	Per 1000 per year	Net contribution to difference
2020s	9.6	10.5	+0.9	+1.91	100%	-1.05	0%
2030s	10.5	11.3	+0.9	+2.29	100%	-1.40	0%
2040s	11.3	12.4	+1.1	+2.43	100%	-1.36	0%
2050s	12.4	12.9	+0.5	+1.88	100%	-1.37	0%
Sub-Saharan Africa							
1970s	20.7	17.7	-3.0	-0.04	1%	-2.95	99%
1980s	17.7	16.2	-1.6	-0.36	23%	-1.19	77%
1990s	16.2	14.3	-1.8	-0.33	18%	-1.52	82%
2000s	14.3	10.9	-3.4	-0.09	3%	-3.35	97%
2010s	10.9	8.8	-2.1	-0.10	5%	-2.01	95%
2020s	8.8	7.6	-1.2	+0.16	0%	-1.33	100%
2030s	7.6	7.3	-0.3	+0.41	0%	-0.75	100%
2040s	7.3	7.2	0.0	+0.64	0%	-0.67	100%
2050s	7.2	7.4	+0.2	+0.87	100%	-0.65	0%
United States							
1970s	9.5	8.7	-0.7	+1.00	0%	-1.74	100%
1980s	8.7	8.6	-0.1	+0.88	0%	-1.00	100%
1990s	8.6	8.6	0.0	+0.57	100%	-0.56	0%
2000s	8.6	8.1	-0.6	+0.76	0%	-1.32	100%
2010s	8.1	8.4	+0.3	+0.67	100%	-0.33	0%
2020s	8.4	9.4	+1.0	+1.94	100%	-0.98	0%
2030s	9.4	10.5	+1.1	+2.26	100%	-1.15	0%
2040s	10.5	11.0	+0.6	+1.72	100%	-1.16	0%
2050s	11.0	10.7	-0.3	+0.81	0%	-1.10	100%
Western Pacific and Southeast Asia							
1970s	12.3	9.6	-2.7	+0.12	0%	-2.87	100%
1980s	9.6	8.1	-1.5	+0.33	0%	-1.85	100%
1990s	8.1	7.2	-0.8	+0.71	0%	-1.54	100%
2000s	7.2	7.0	-0.2	+1.10	0%	-1.33	100%
2010s	7.0	7.2	+0.2	+1.13	100%	-0.91	0%
2020s	7.2	8.1	+0.9	+1.56	100%	-0.68	0%
2030s	8.1	9.2	+1.1	+1.66	100%	-0.60	0%
2040s	9.2	10.2	+1.0	+1.72	100%	-0.72	0%
2050s	10.2	11.1	+1.0	+1.81	100%	-0.85	0%

Notes: First year = The first year of the decadal period. For all periods except the 2020s, the first year of the decadal period is the first year of the decade (i.e., the year ending in -0; e.g., for the 1970s, the first year is 1970). For the 2020s, the first year of the decadal period is 2019, rather than 2020, to avoid COVID-19 impacts. Last year = The last year of the decadal period. For all periods except the 2010s, the last year of the decadal period is the first year of the subsequent decade (e.g., for the 1970s, the last year is 1980). For the 2010s, the last year of the decadal period is 2019, rather than 2020, to avoid COVID-19 impacts. Difference = The change in CDR between the last year and first year of the decade as defined above.

Figure A7. Age-standardized death rates of I-8 and NCDI-7 conditions for the World, 2000-2019, for both sexes and by sex.

Both sexes

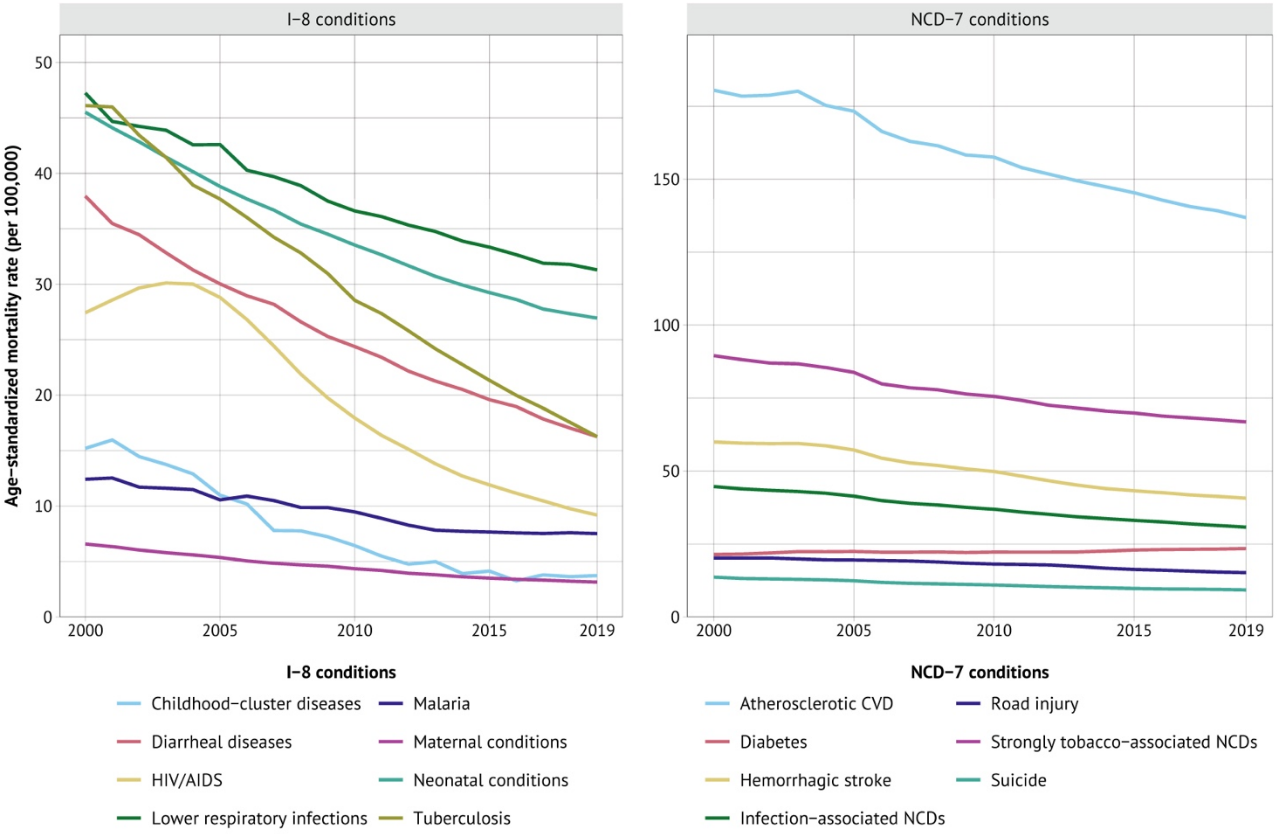

Females

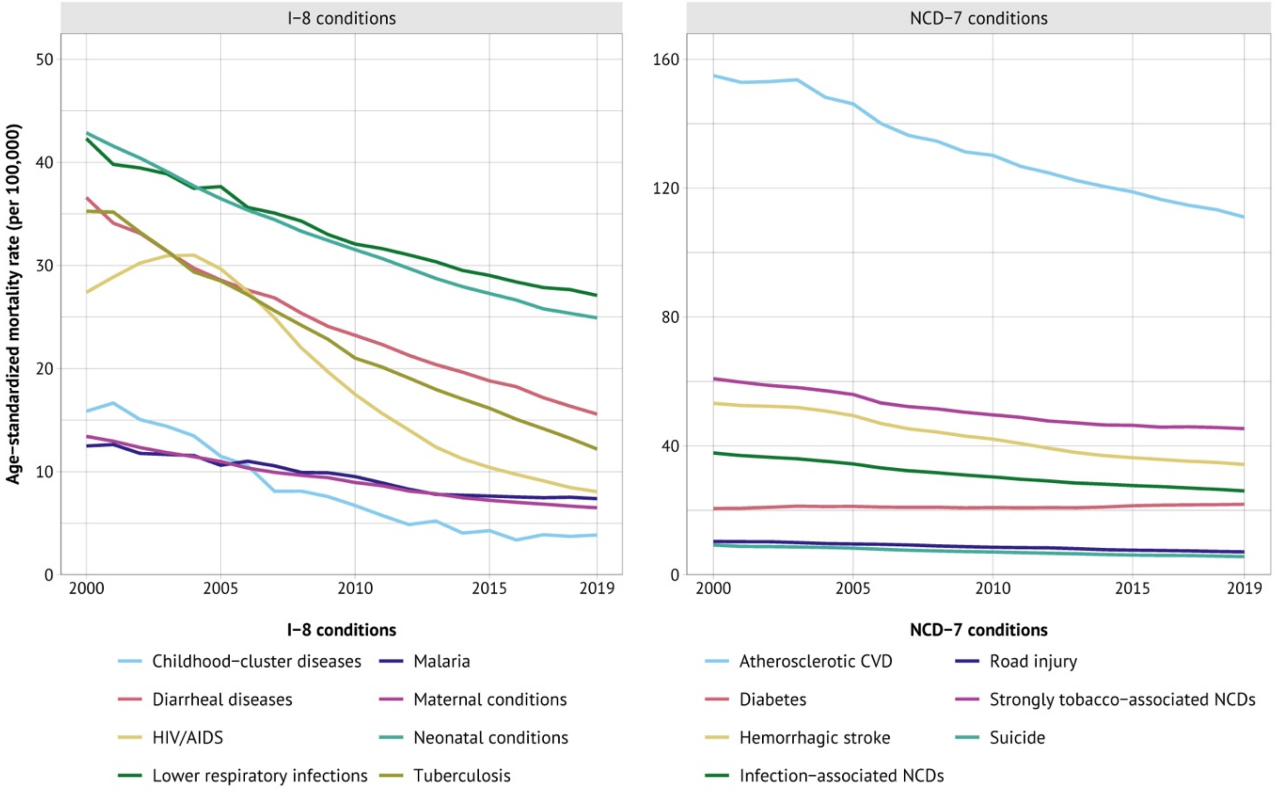

Males

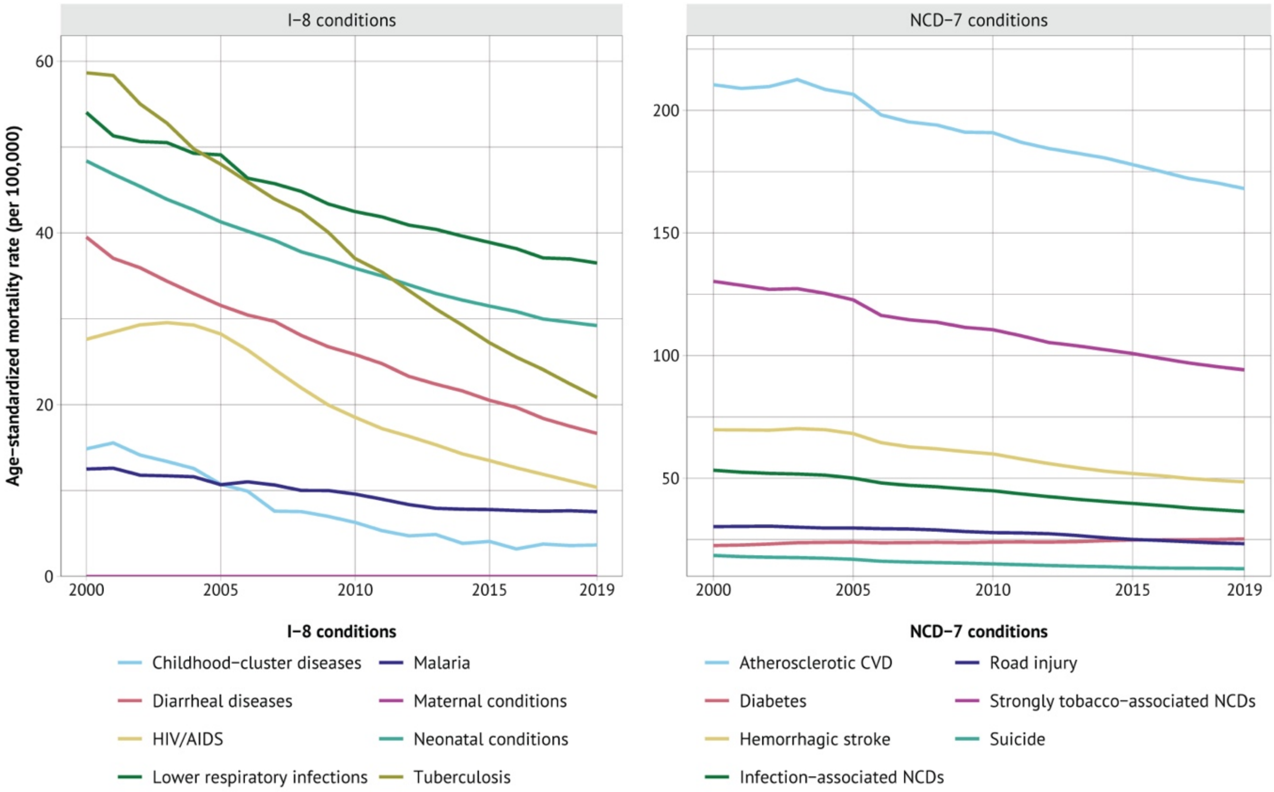

I–8 conditions
NCD–7 conditions
Age–standardized mortality rate (per 100,000)
0
20
40
60
50
100
150
200
2000
2005
2010
2015
2019
I–8 conditions
Childhood–cluster diseases
Malaria
Diarrheal diseases
Maternal conditions
HIV/AIDS
Neonatal conditions
Lower respiratory infections
Tuberculosis
NCD–7 conditions
Atherosclerotic CVD
Road injury
Diabetes
Strongly tobacco–associated NCDs
Hemorrhagic stroke
Suicide
Infection–associated NCDs

Table A6. Age-standardized death rates of I-8 and NCDI-7 conditions for the World, 2000, 2019.

	Age-standardized death rate (per 100,000)		Percent change			
	2000	2019	2000-2019	2000-2009	2010-2014	2015-2019
I-8 conditions						
Childhood-cluster diseases	15.3	3.8	-76%	-53%	-39%	-10%
Diarrheal diseases	37.8	16.1	-57%	-33%	-16%	-18%
HIV/AIDS	27.4	9.2	-66%	-28%	-29%	-23%
Lower respiratory infections	47.2	31.2	-34%	-21%	-7%	-6%
Malaria	12.5	7.5	-40%	-20%	-19%	-3%
Maternal conditions	6.6	3.2	-52%	-30%	-17%	-10%
Neonatal conditions	45.7	27.1	-41%	-24%	-11%	-8%
Tuberculosis	45.9	16.3	-65%	-33%	-20%	-24%
NCDI-7 conditions						
Atherosclerotic CVD	180.7	137.0	-24%	-12%	-6%	-6%
Diabetes	21.4	23.4	+9%	+3%	+2%	+2%
Hemorrhagic stroke	60.5	40.7	-33%	-16%	-12%	-6%
Infection-associated NCDs	44.8	30.8	-31%	-16%	-9%	-7%
Road injury	20.2	15.2	-25%	-9%	-8%	-7%
Strongly tobacco-associated NCDs	90.5	66.8	-26%	-15%	-7%	-5%
Suicide	13.7	9.3	-32%	-18%	-9%	-5%

Figure A8. Proportion of I-8 and NCDI-7 deaths by region and by sex in 2000, 2010, 2019.

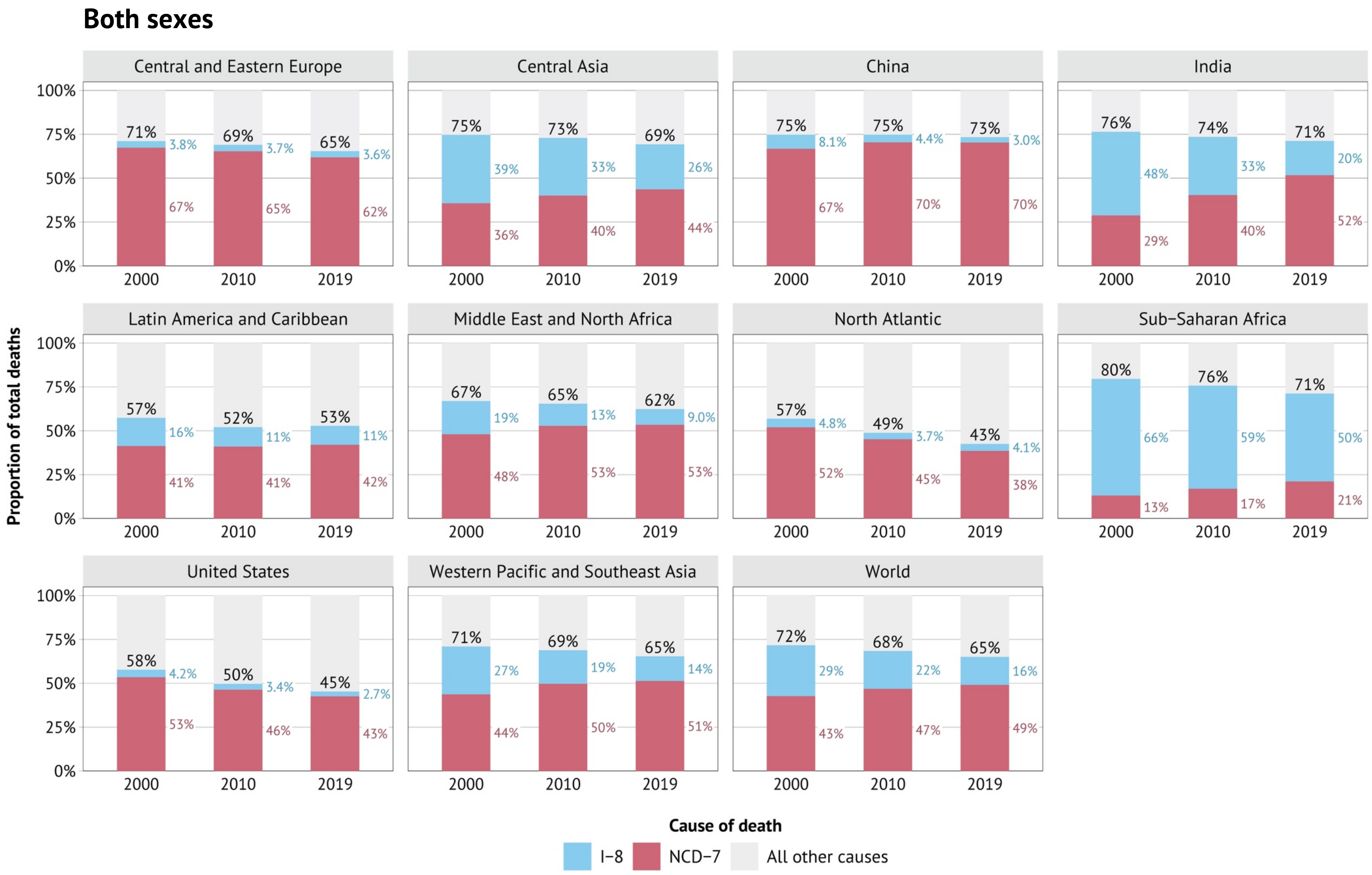

Females

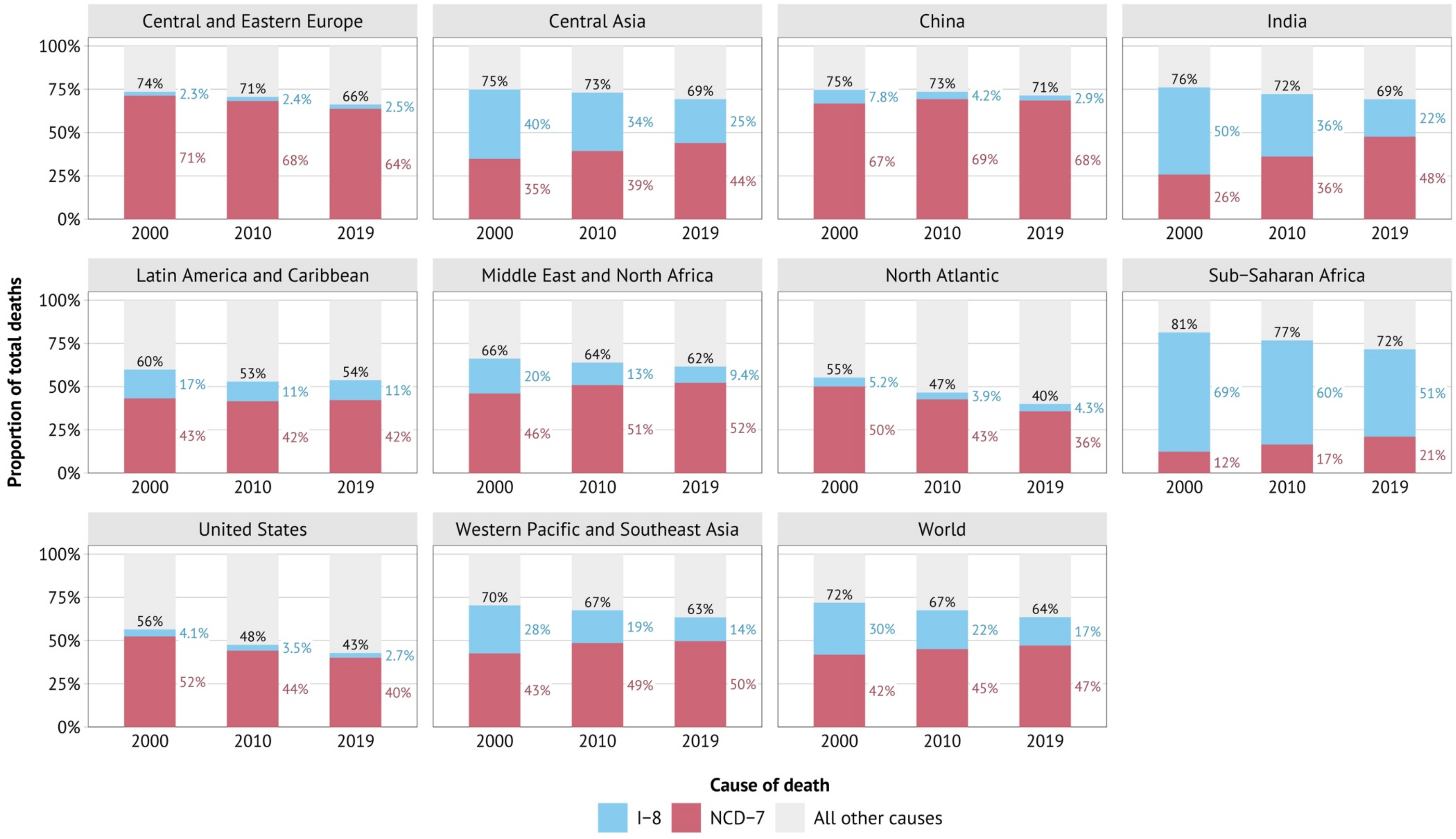
Central and Eastern Europe
2000: 74%, 2.3%, 71%
2010: 71%, 2.4%, 68%
2019: 66%, 2.5%, 64%
Central Asia
2000: 75%, 40%, 35%
2010: 73%, 34%, 39%
2019: 69%, 25%, 44%
China
2000: 75%, 7.8%, 67%
2010: 73%, 4.2%, 69%
2019: 71%, 2.9%, 68%
India
2000: 76%, 50%, 26%
2010: 72%, 36%, 36%
2019: 69%, 22%, 48%
Latin America and Caribbean
2000: 60%, 17%, 43%
2010: 53%, 11%, 42%
2019: 54%, 11%, 42%
Middle East and North Africa
2000: 66%, 20%, 46%
2010: 64%, 13%, 51%
2019: 62%, 9.4%, 52%
North Atlantic
2000: 55%, 5.2%, 50%
2010: 47%, 3.9%, 43%
2019: 40%, 4.3%, 36%
Sub–Saharan Africa
2000: 81%, 69%, 12%
2010: 77%, 60%, 17%
2019: 72%, 51%, 21%
United States
2000: 56%, 4.1%, 52%
2010: 48%, 3.5%, 44%
2019: 43%, 2.7%, 40%
Western Pacific and Southeast Asia
2000: 70%, 28%, 43%
2010: 67%, 19%, 49%
2019: 63%, 14%, 50%
World
2000: 72%, 30%, 42%
2010: 67%, 22%, 45%
2019: 64%, 17%, 47%
Proportion of total deaths
0% 25% 50% 75% 100%
Cause of death
I–8
NCD–7
All other causes

Males

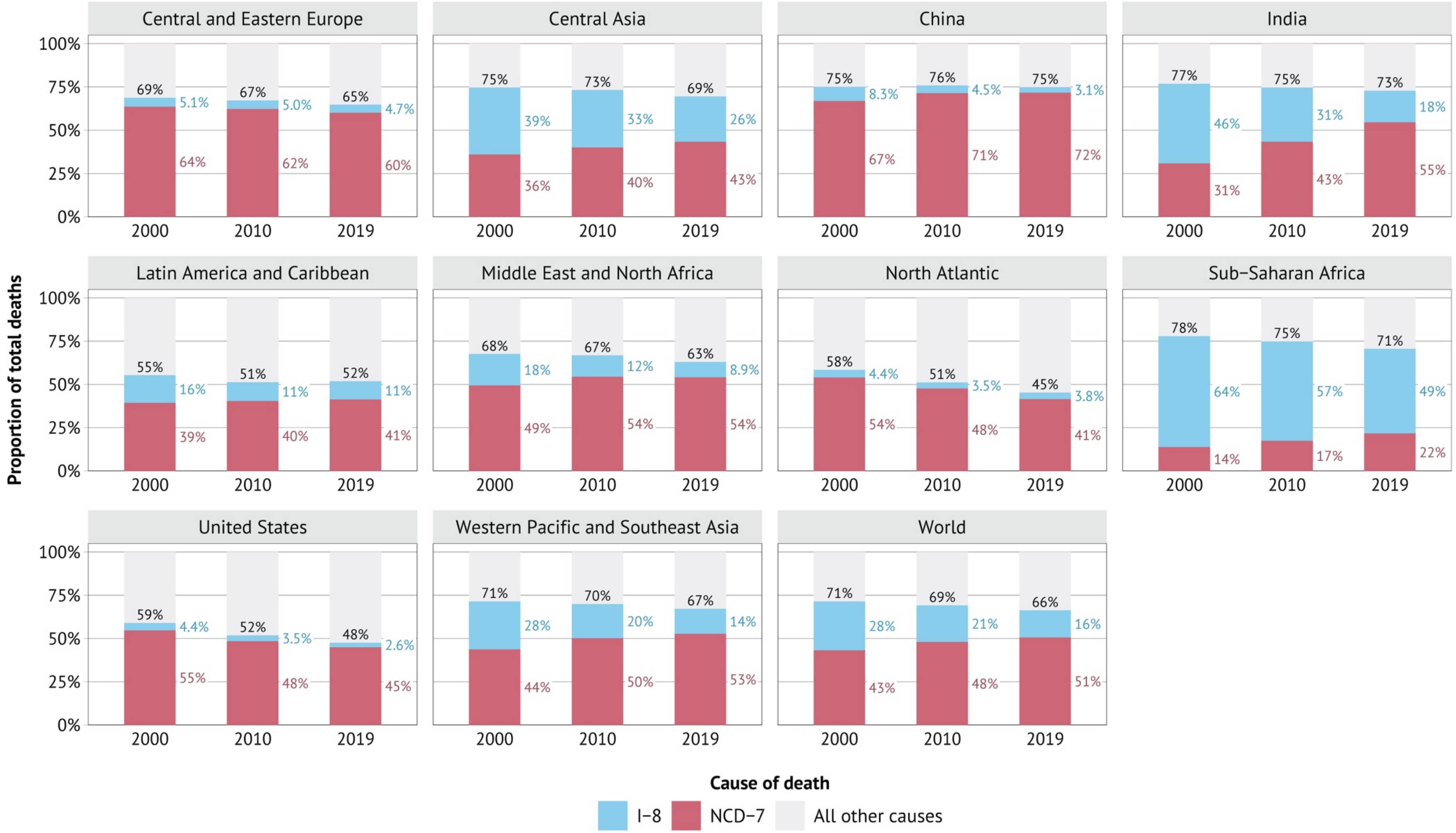
Central and Eastern Europe
Central Asia
China
India
Latin America and Caribbean
Middle East and North Africa
North Atlantic
Sub−Saharan Africa
United States
Western Pacific and Southeast Asia
World
Proportion of total deaths
100%
75%
50%
25%
0%
2000
2010
2019
Cause of death
I−8
NCD−7
All other causes

Figure A9. Proportion of deaths by priority conditions, World in 2000, 2010, and 2019.

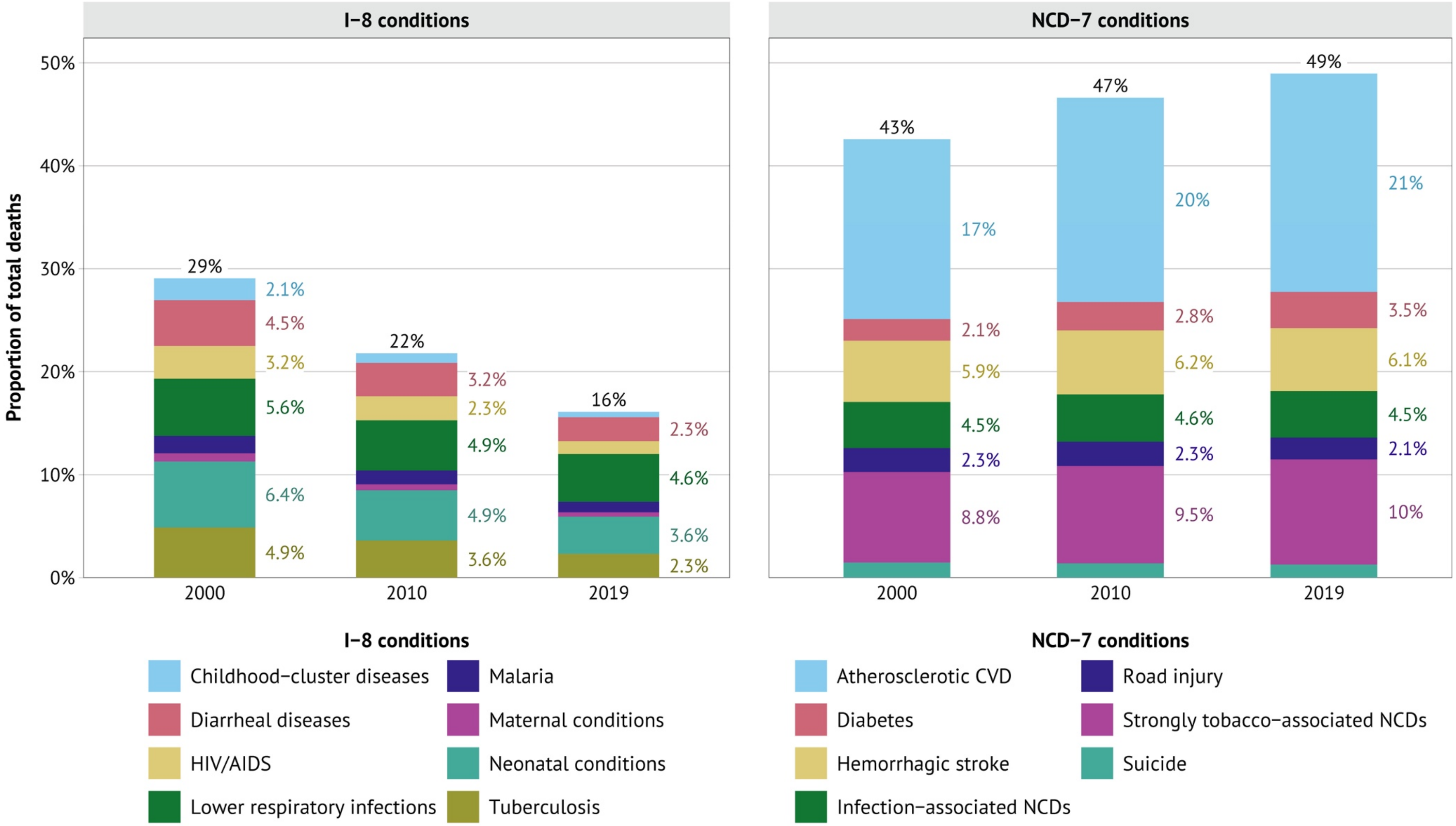

Notes: The percentage of total deaths attributed to a specific cause is only shown to the right of the bar if that cause accounted for at least 2% of total deaths in that year.

Table A7. Decomposition of I-8 and NCDI-7 conditions into component (changes in population size and age-specific mortality rates), globally for (A) both sexes, (B) females and (C) males 2000-2010, 2010-2019.

(A) Both sexes	Deaths (thousands)			Deaths AARC		Population size AARC		Death rate AARC	
	2000	2010	2019	2000s	2010s	2000s	2010s	2000s	2010s
I-8	15 000	11 600	9 170	-2.5%	-2.6%	+1.3%	+1.2%	-3.7%	-3.8%
Childhood-cluster diseases	1 080	485	291	-7.7%	-5.5%	+1.3%	+1.2%	-8.9%	-6.6%
Diarrheal diseases	2 300	1 730	1 320	-2.8%	-2.9%	+1.3%	+1.2%	-4.1%	-4.1%
HIV/AIDS	1 630	1 250	715	-2.6%	-6.0%	+1.3%	+1.2%	-3.9%	-7.1%
Lower respiratory infections	2 870	2 600	2 630	-1.0%	+0.1%	+1.3%	+1.2%	-2.2%	-1.0%
Malaria	867	713	578	-1.9%	-2.3%	+1.3%	+1.2%	-3.2%	-3.5%
Maternal conditions	410	310	240	-2.8%	-2.8%	+1.3%	+1.2%	-4.0%	-3.9%
Neonatal conditions	3 300	2 610	2 060	-2.3%	-2.6%	+1.3%	+1.2%	-3.5%	-3.8%
Tuberculosis	2 520	1 930	1 330	-2.6%	-4.1%	+1.3%	+1.2%	-3.9%	-5.2%
NCDI-7	21 900	24 900	27 900	+1.3%	+1.3%	+1.3%	+1.2%	0.0%	+0.1%
Atherosclerotic CVD	8 990	10 600	12 100	+1.6%	+1.5%	+1.3%	+1.2%	+0.3%	+0.3%
Diabetes	1 080	1 470	2 010	+3.1%	+3.5%	+1.3%	+1.2%	+1.8%	+2.3%
Hemorrhagic stroke	3 060	3 330	3 490	+0.8%	+0.5%	+1.3%	+1.2%	-0.5%	-0.7%
Infection-associated NCDs	2 310	2 460	2 580	+0.6%	+0.5%	+1.3%	+1.2%	-0.7%	-0.7%
Road injury	1 180	1 250	1 200	+0.6%	-0.5%	+1.3%	+1.2%	-0.7%	-1.7%
Strongly tobacco-associated NCDs	4 530	5 050	5 810	+1.1%	+1.6%	+1.3%	+1.2%	-0.2%	+0.4%
Suicide	771	749	736	-0.3%	-0.2%	+1.3%	+1.2%	-1.6%	-1.4%

(B) Females	Deaths (thousands)			Deaths AARC		Population size AARC		Death rate AARC	
	2000	2010	2019	2000s	2010s	2000s	2010s	2000s	2010s
I-8	7 240	5 520	4 340	-2.7%	-2.6%	+1.3%	+1.2%	-3.9%	-3.8%
Childhood-cluster diseases	541	243	146	-7.7%	-5.5%	+1.3%	+1.2%	-8.9%	-6.6%
Diarrheal diseases	1 160	865	682	-2.9%	-2.6%	+1.3%	+1.2%	-4.1%	-3.8%
HIV/AIDS	815	603	308	-3.0%	-7.2%	+1.3%	+1.2%	-4.2%	-8.3%
Lower respiratory infections	1 370	1 230	1 250	-1.1%	+0.2%	+1.3%	+1.2%	-2.4%	-1.0%
Malaria	421	346	280	-1.9%	-2.3%	+1.3%	+1.2%	-3.2%	-3.5%
Maternal conditions	411	310	240	-2.8%	-2.8%	+1.3%	+1.2%	-4.0%	-4.0%
Neonatal conditions	1 490	1 180	919	-2.3%	-2.7%	+1.3%	+1.2%	-3.6%	-3.9%
Tuberculosis	1 030	745	516	-3.1%	-4.0%	+1.3%	+1.2%	-4.4%	-5.1%
NCDI-7	10 100	11 100	12 300	+0.9%	+1.2%	+1.3%	+1.2%	-0.4%	0.0%
Atherosclerotic CVD	4 610	5 180	5 740	+1.2%	+1.1%	+1.3%	+1.2%	-0.1%	0.0%
Diabetes	585	771	1 040	+2.8%	+3.4%	+1.3%	+1.2%	+1.5%	+2.2%
Hemorrhagic stroke	1 520	1 570	1 630	+0.3%	+0.4%	+1.3%	+1.2%	-1.0%	-0.7%
Infection-associated NCDs	1 060	1 090	1 160	+0.3%	+0.8%	+1.3%	+1.2%	-1.0%	-0.4%
Road injury	307	298	287	-0.3%	-0.4%	+1.3%	+1.2%	-1.6%	-1.6%
Strongly tobacco-associated NCDs	1 780	1 910	2 250	+0.7%	+1.8%	+1.3%	+1.2%	-0.6%	+0.7%
Suicide	270	248	228	-0.8%	-0.9%	+1.3%	+1.2%	-2.1%	-2.1%

(C) Males	Deaths (thousands)			Deaths AARC		Population size AARC		Death rate AARC	
	2000	2010	2019	2000s	2010s	2000s	2010s	2000s	2010s
I-8	7 730	6 110	4 830	-2.3%	-2.6%	+1.3%	+1.2%	-3.6%	-3.7%
Childhood-cluster diseases	537	242	146	-7.7%	-5.5%	+1.3%	+1.2%	-8.9%	-6.6%
Diarrheal diseases	1 140	864	642	-2.8%	-3.2%	+1.3%	+1.2%	-4.0%	-4.4%
HIV/AIDS	820	646	407	-2.4%	-5.0%	+1.3%	+1.2%	-3.6%	-6.1%
Lower respiratory infections	1 490	1 370	1 380	-0.8%	+0.1%	+1.3%	+1.2%	-2.1%	-1.1%
Malaria	445	367	298	-1.9%	-2.3%	+1.3%	+1.2%	-3.2%	-3.4%
Neonatal conditions	1 800	1 430	1 140	-2.3%	-2.5%	+1.3%	+1.2%	-3.5%	-3.7%
Tuberculosis	1 490	1 190	812	-2.3%	-4.1%	+1.3%	+1.2%	-3.5%	-5.3%
NCDI-7	11 800	13 800	15 500	+1.6%	+1.3%	+1.3%	+1.2%	+0.3%	+0.1%
Atherosclerotic CVD	4 370	5 410	6 320	+2.1%	+1.8%	+1.3%	+1.2%	+0.8%	+0.6%
Diabetes	498	703	967	+3.5%	+3.6%	+1.3%	+1.2%	+2.2%	+2.4%
Hemorrhagic stroke	1 540	1 760	1 860	+1.3%	+0.6%	+1.3%	+1.2%	0.0%	-0.6%
Infection-associated NCDs	1 260	1 370	1 410	+0.9%	+0.3%	+1.3%	+1.2%	-0.4%	-0.8%
Road injury	877	953	911	+0.8%	-0.5%	+1.3%	+1.2%	-0.5%	-1.7%
Strongly tobacco-associated NCDs	2 740	3 140	3 560	+1.4%	+1.4%	+1.3%	+1.2%	+0.1%	+0.2%
Suicide	502	502	509	0.0%	+0.2%	+1.3%	+1.2%	-1.3%	-1.0%

Note: AARC = Average annual rate of change (% per year); 2000s = 2000-2010; 2010s = 2010-2019 (2019 is substituted for 2020)

Figure A10. Decomposition of the average annual rate of change of the 15 priority conditions into changes in cause-specific mortality rates and changes in population size, World, 2000-2010, 2010-2019.

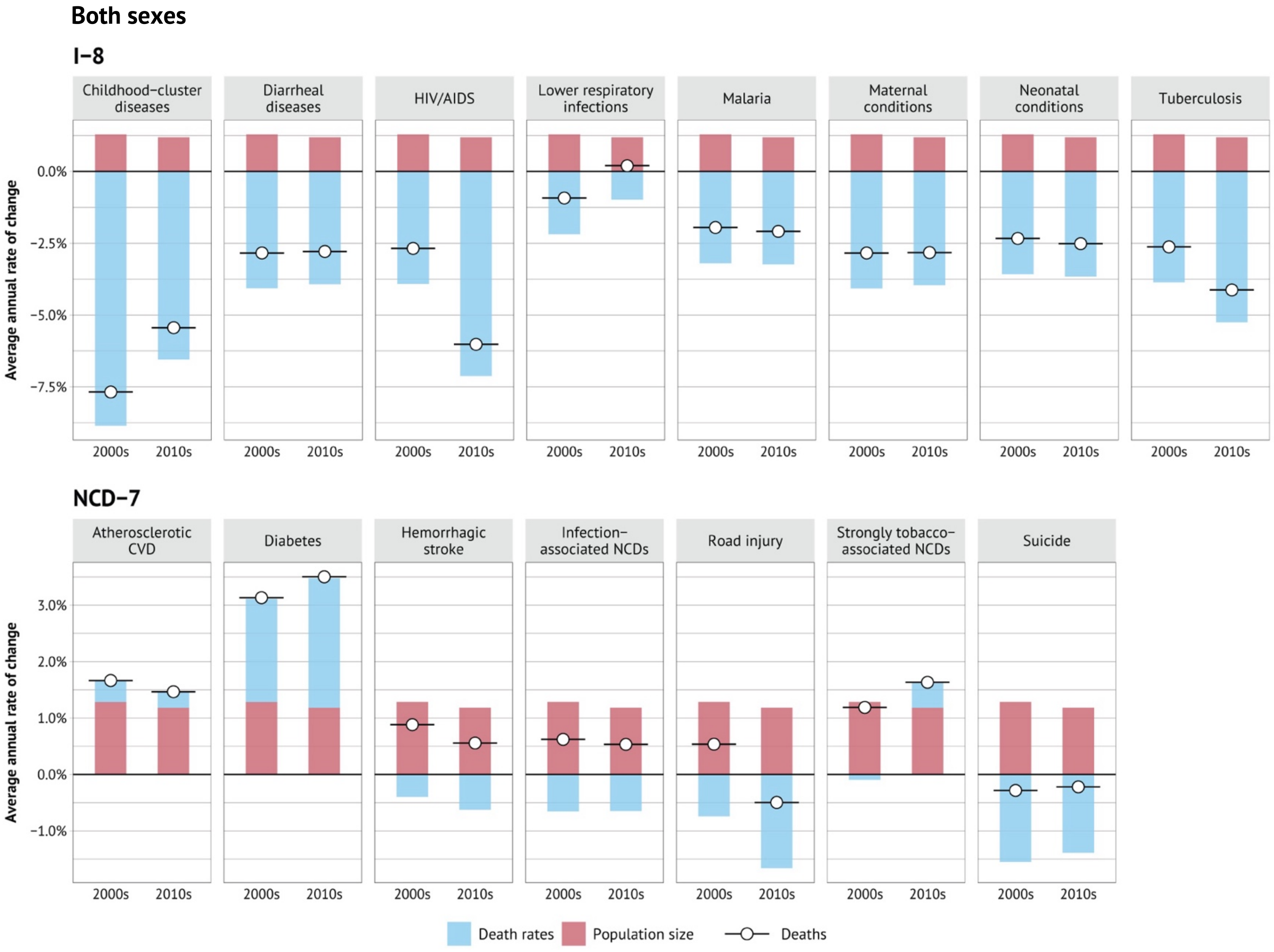

Note: 2000s = 2000-2010; 2010s = 2010-2019 (2019 is substituted for 2020 to avoid COVID-19 impacts)

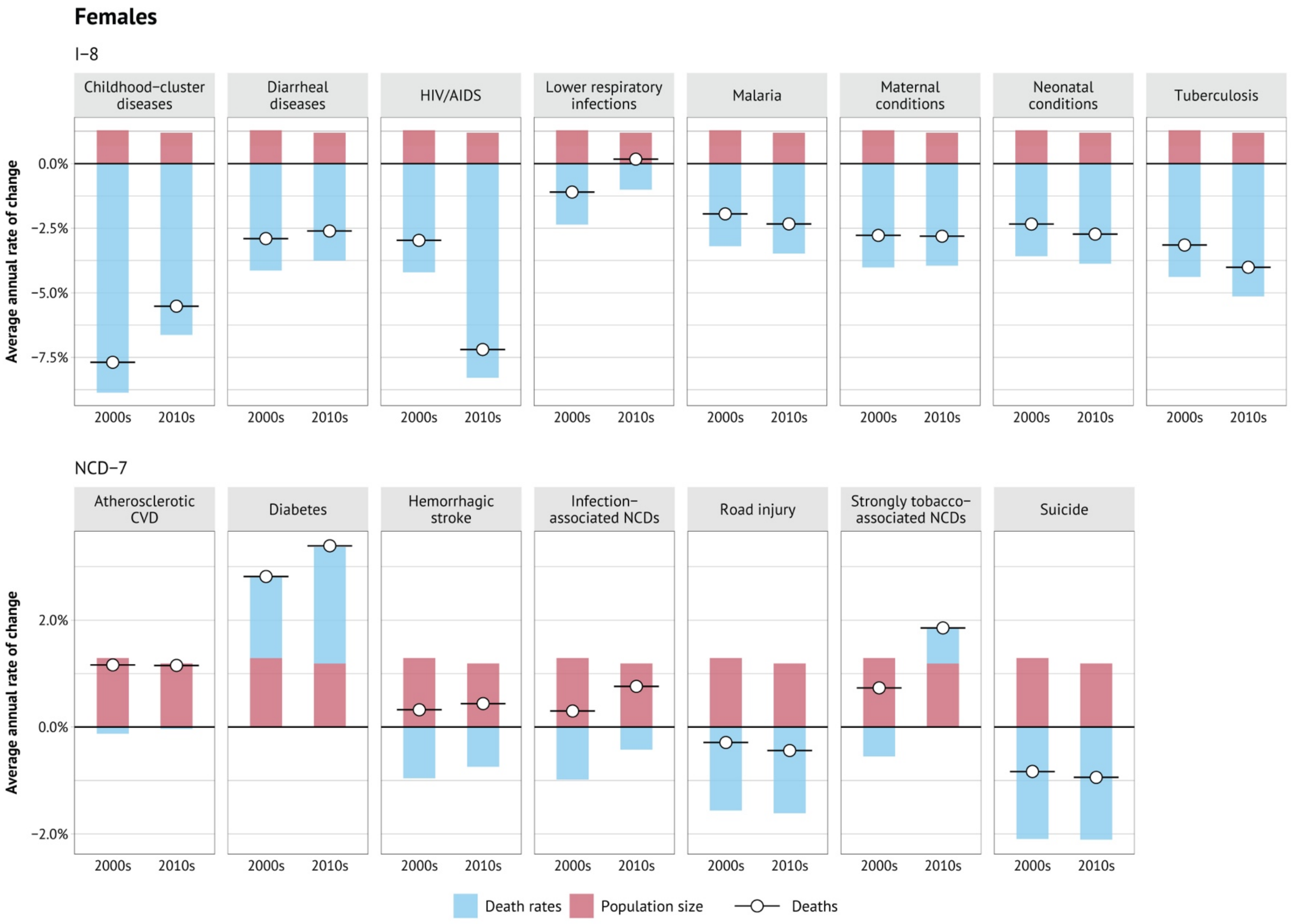

Females
I–8
Childhood–cluster diseases
Diarrheal diseases
HIV/AIDS
Lower respiratory infections
Malaria
Maternal conditions
Neonatal conditions
Tuberculosis
NCD–7
Atherosclerotic CVD
Diabetes
Hemorrhagic stroke
Infection–associated NCDs
Road injury
Strongly tobacco–associated NCDs
Suicide
Average annual rate of change
0.0%
−2.5%
−5.0%
−7.5%
2.0%
−2.0%
2000s
2010s
Death rates
Population size
Deaths

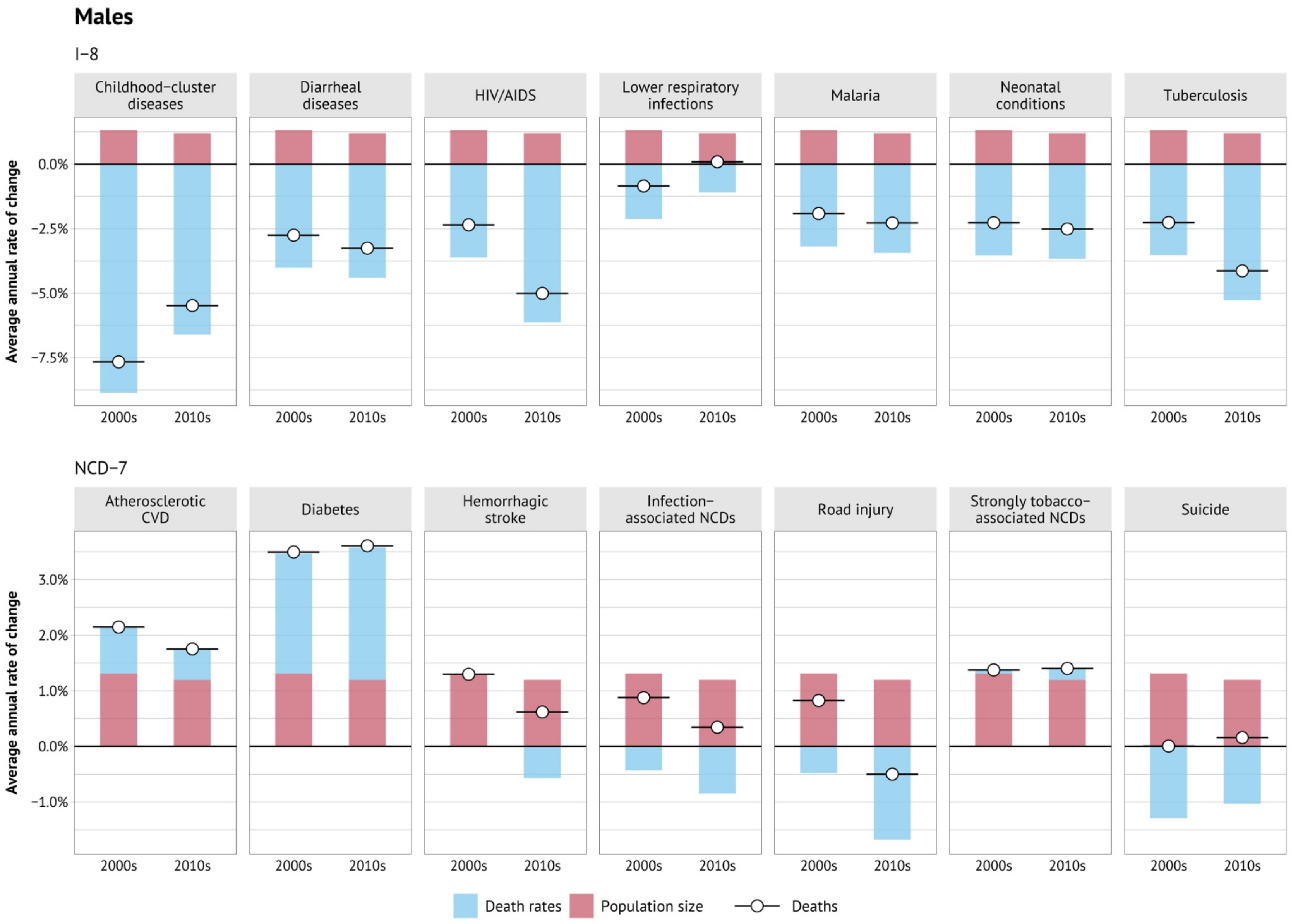
Males
I-8
Childhood-cluster diseases
Diarrheal diseases
HIV/AIDS
Lower respiratory infections
Malaria
Neonatal conditions
Tuberculosis
Average annual rate of change
0.0%
-2.5%
-5.0%
-7.5%
2000s
2010s
NCD-7
Atherosclerotic CVD
Diabetes
Hemorrhagic stroke
Infection-associated NCDs
Road injury
Strongly tobacco-associated NCDs
Suicide
3.0%
2.0%
1.0%
0.0%
-1.0%
Death rates
Population size
Deaths

Table A8. Rates of change in population size and cause-specific number of deaths and death rates for I-8 conditions, 2000-2010, 2010-2019, and 2000-2019.

Panel A: I-8
Panel B: Childhood-cluster diseases
Panel C: Diarrheal diseases
Panel D: HIV/AIDS
Panel E: Lower respiratory infections
Panel F: Malaria
Panel G: Maternal conditions
Panel H: Neonatal conditions
Panel I: Tuberculosis

Panel A1: I-8 - World

	Age group				
	0-14	15-49	50-69	70+	All ages
Population size (millions)					
2000	1,870	3,210	808	269	6,160
2010	1,910	3,670	1,080	355	7,010
2019	2,030	3,930	1,380	451	7,800
Rate of change in population size (% per year)					
2000-2010	+0.2%	+1.3%	+2.9%	+2.8%	+1.3%
2010-2019	+0.7%	+0.8%	+2.9%	+2.7%	+1.2%
2000-2019	+0.4%	+1.1%	+2.9%	+2.7%	+1.2%
Deaths (thousands)					
2000	8,740	2,650	1,640	1,940	15,000
2010	6,110	2,120	1,420	1,990	11,600
2019	4,400	1,430	1,250	2,100	9,170
Rate of change in deaths (% per year)					
2000-2010	-3.5%	-2.2%	-1.4%	+0.2%	-2.5%
2010-2019	-3.6%	-4.3%	-1.5%	+0.6%	-2.6%
2000-2019	-3.6%	-3.2%	-1.4%	+0.4%	-2.6%
Death rate (per 100 000 population per year)					
2000	467	82.6	203	721	243
2010	319	57.7	132	560	166
2019	217	36.3	89.9	465	118
Rate of change in death rate (% per year)					
2000-2010	-3.7%	-3.5%	-4.2%	-2.5%	-3.7%
2010-2019	-4.2%	-5.0%	-4.2%	-2.0%	-3.8%
2000-2019	-4.0%	-4.2%	-4.2%	-2.3%	-3.8%

Data source: Global Health Estimates 2021

Panel A2: I-8 - Central and Eastern Europe

	Age group				
	0-14	15-49	50-69	70+	All ages
Population size (millions)					
2000	63.2	181	70.2	29.2	343
2010	50.2	169	78.6	34.2	332
2019	54.6	153	85.8	35.2	329
Rate of change in population size (% per year)					
2000-2010	-2.3%	-0.7%	+1.1%	+1.6%	-0.3%
2010-2019	+0.9%	-1.1%	+1.0%	+0.3%	-0.1%
2000-2019	-0.8%	-0.9%	+1.1%	+1.0%	-0.2%
Deaths (thousands)					
2000	31.5	63.6	47.9	31.4	174
2010	18.2	62.5	47.5	33.3	161
2019	10.5	46.3	40.1	49.2	146
Rate of change in deaths (% per year)					
2000-2010	-5.3%	-0.2%	-0.1%	+0.6%	-0.8%
2010-2019	-6.0%	-3.3%	-1.8%	+4.4%	-1.1%
2000-2019	-5.6%	-1.7%	-0.9%	+2.4%	-0.9%
Death rate (per 100 000 population per year)					
2000	49.7	35.2	68.3	108	50.8
2010	36.2	37.0	60.4	97.5	48.7
2019	19.1	30.2	46.8	140	44.4
Rate of change in death rate (% per year)					
2000-2010	-3.1%	+0.5%	-1.2%	-1.0%	-0.4%
2010-2019	-6.8%	-2.2%	-2.8%	+4.1%	-1.0%
2000-2019	-4.9%	-0.8%	-2.0%	+1.4%	-0.7%

Data source: Global Health Estimates 2021

Panel A3: I-8 - Central Asia

	Age group				
	0-14	15-49	50-69	70+	All ages
Population size (millions)					
2000	100	115	21.7	5.4	242
2010	114	153	29.3	7.3	303
2019	131	177	39.7	8.8	357
Rate of change in population size (% per year)					
2000-2010	+1.3%	+2.9%	+3.0%	+3.1%	+2.3%
2010-2019	+1.6%	+1.6%	+3.4%	+2.1%	+1.8%
2000-2019	+1.4%	+2.3%	+3.2%	+2.6%	+2.1%
Deaths (thousands)					
2000	697	86.5	44.9	45.5	874
2010	618	84.9	40.2	44.5	788
2019	447	72.5	38.6	42.7	601
Rate of change in deaths (% per year)					
2000-2010	-1.2%	-0.2%	-1.1%	-0.2%	-1.0%
2010-2019	-3.5%	-1.7%	-0.5%	-0.5%	-3.0%
2000-2019	-2.3%	-0.9%	-0.8%	-0.3%	-2.0%
Death rate (per 100 000 population per year)					
2000	698	75.4	207	840	362
2010	544	55.4	137	608	260
2019	341	40.9	97.2	484	168
Rate of change in death rate (% per year)					
2000-2010	-2.5%	-3.0%	-4.0%	-3.2%	-3.3%
2010-2019	-5.1%	-3.3%	-3.8%	-2.5%	-4.7%
2000-2019	-3.7%	-3.2%	-3.9%	-2.9%	-3.9%

Data source: Global Health Estimates 2021

Panel A4: I-8 - China

	Age group				
	0-14	15-49	50-69	70+	All ages
Population size (millions)					
2000	312	723	180	54.9	1,270
2010	250	767	257	77.6	1,350
2019	259	708	354	103	1,420
Rate of change in population size (% per year)					
2000-2010	-2.2%	+0.6%	+3.6%	+3.5%	+0.6%
2010-2019	+0.4%	-0.9%	+3.6%	+3.1%	+0.6%
2000-2019	-1.0%	-0.1%	+3.6%	+3.3%	+0.6%
Deaths (thousands)					
2000	411	79.5	68.5	178	737
2010	152	71.5	49.6	174	447
2019	61.2	41.8	47.7	180	331
Rate of change in deaths (% per year)					
2000-2010	-9.4%	-1.1%	-3.2%	-0.3%	-4.9%
2010-2019	-9.6%	-5.8%	-0.4%	+0.4%	-3.3%
2000-2019	-9.5%	-3.3%	-1.9%	0.0%	-4.1%
Death rate (per 100 000 population per year)					
2000	132	11.0	38.0	325	58.1
2010	61.1	9.3	19.3	224	33.1
2019	23.6	5.9	13.5	175	23.2
Rate of change in death rate (% per year)					
2000-2010	-7.4%	-1.6%	-6.6%	-3.7%	-5.5%
2010-2019	-10%	-5.0%	-3.9%	-2.7%	-3.9%
2000-2019	-8.6%	-3.2%	-5.3%	-3.2%	-4.7%

Data source: Global Health Estimates 2021

Panel A5: I-8 - India

	Age group				
	0-14	15-49	50-69	70+	All ages
Population size (millions)					
2000	376	543	111	27.6	1,060
2010	390	659	157	37.8	1,240
2019	373	757	209	50.5	1,390
Rate of change in population size (% per year)					
2000-2010	+0.4%	+1.9%	+3.6%	+3.2%	+1.6%
2010-2019	-0.5%	+1.6%	+3.2%	+3.3%	+1.2%
2000-2019	0.0%	+1.8%	+3.4%	+3.2%	+1.4%
Deaths (thousands)					
2000	2,310	701	584	654	4,250
2010	1,350	470	468	614	2,910
2019	666	231	361	577	1,830
Rate of change in deaths (% per year)					
2000-2010	-5.2%	-3.9%	-2.2%	-0.6%	-3.7%
2010-2019	-7.6%	-7.6%	-2.8%	-0.7%	-5.0%
2000-2019	-6.3%	-5.7%	-2.5%	-0.7%	-4.3%
Death rate (per 100 000 population per year)					
2000	614	129	527	2,370	401
2010	347	71.4	298	1,620	234
2019	179	30.5	173	1,140	132
Rate of change in death rate (% per year)					
2000-2010	-5.5%	-5.7%	-5.5%	-3.7%	-5.3%
2010-2019	-7.1%	-9.0%	-5.9%	-3.8%	-6.1%
2000-2019	-6.3%	-7.3%	-5.7%	-3.8%	-5.7%

Data source: Global Health Estimates 2021

Panel A6: I-8 - Latin America and Caribbean

	Age group				
	0-14	15-49	50-69	70+	All ages
Population size (millions)					
2000	166	272	59.4	17.7	515
2010	161	312	83.7	25.4	583
2019	155	337	110	34.6	636
Rate of change in population size (% per year)					
2000-2010	-0.3%	+1.4%	+3.5%	+3.7%	+1.2%
2010-2019	-0.5%	+0.8%	+3.1%	+3.5%	+1.0%
2000-2019	-0.4%	+1.1%	+3.3%	+3.6%	+1.1%
Deaths (thousands)					
2000	256	78.4	38.9	76.7	450
2010	138	66.5	47.6	124	376
2019	107	59.5	65.9	193	425
Rate of change in deaths (% per year)					
2000-2010	-6.0%	-1.6%	+2.0%	+4.9%	-1.8%
2010-2019	-2.8%	-1.2%	+3.7%	+5.0%	+1.4%
2000-2019	-4.5%	-1.4%	+2.8%	+5.0%	-0.3%
Death rate (per 100 000 population per year)					
2000	154	28.9	65.6	434	87.4
2010	85.4	21.3	56.9	490	64.6
2019	69.1	17.7	59.7	556	66.8
Rate of change in death rate (% per year)					
2000-2010	-5.7%	-3.0%	-1.4%	+1.2%	-3.0%
2010-2019	-2.3%	-2.1%	+0.5%	+1.4%	+0.4%
2000-2019	-4.1%	-2.6%	-0.5%	+1.3%	-1.4%

Data source: Global Health Estimates 2021

Panel A7: I-8 - Middle East and North Africa

	Age group				
	0-14	15-49	50-69	70+	All ages
Population size (millions)					
2000	135	200	36.9	9.6	381
2010	140	259	53.6	13.8	467
2019	163	296	76.1	18.3	553
Rate of change in population size (% per year)					
2000-2010	+0.4%	+2.6%	+3.8%	+3.7%	+2.0%
2010-2019	+1.7%	+1.5%	+4.0%	+3.2%	+1.9%
2000-2019	+1.0%	+2.1%	+3.9%	+3.5%	+2.0%
Deaths (thousands)					
2000	293	25.5	13.7	20.8	353
2010	199	24.5	16.1	30.3	270
2019	152	22.3	20.8	44.2	240
Rate of change in deaths (% per year)					
2000-2010	-3.8%	-0.4%	+1.6%	+3.8%	-2.6%
2010-2019	-2.9%	-1.0%	+2.9%	+4.3%	-1.3%
2000-2019	-3.4%	-0.7%	+2.2%	+4.1%	-2.0%
Death rate (per 100 000 population per year)					
2000	217	12.8	37.1	216	92.5
2010	142	9.5	30.1	219	57.9
2019	93.7	7.5	27.3	241	43.3
Rate of change in death rate (% per year)					
2000-2010	-4.1%	-2.9%	-2.1%	+0.1%	-4.6%
2010-2019	-4.5%	-2.5%	-1.0%	+1.1%	-3.2%
2000-2019	-4.3%	-2.7%	-1.6%	+0.6%	-3.9%

Data source: Global Health Estimates 2021

Panel A8: I-8 - North Atlantic

	Age group				
	0-14	15-49	50-69	70+	All ages
Population size (millions)					
2000	72.0	209	93.2	47.7	422
2010	71.3	211	107	57.5	446
2019	72.1	202	122	67.8	464
Rate of change in population size (% per year)					
2000-2010	-0.1%	+0.1%	+1.4%	+1.9%	+0.6%
2010-2019	+0.1%	-0.4%	+1.5%	+1.9%	+0.4%
2000-2019	0.0%	-0.2%	+1.4%	+1.9%	+0.5%
Deaths (thousands)					
2000	11.8	9.9	14.7	156	193
2010	10.2	6.0	12.0	122	151
2019	10.2	3.7	14.0	151	179
Rate of change in deaths (% per year)					
2000-2010	-1.4%	-5.0%	-2.0%	-2.4%	-2.4%
2010-2019	0.0%	-5.1%	+1.7%	+2.4%	+1.9%
2000-2019	-0.8%	-5.0%	-0.2%	-0.2%	-0.4%
Death rate (per 100 000 population per year)					
2000	16.4	4.8	15.8	327	45.7
2010	14.4	2.8	11.3	213	33.7
2019	14.2	1.8	11.5	223	38.6
Rate of change in death rate (% per year)					
2000-2010	-1.3%	-5.1%	-3.3%	-4.2%	-3.0%
2010-2019	-0.1%	-4.7%	+0.2%	+0.5%	+1.5%
2000-2019	-0.8%	-4.9%	-1.6%	-2.0%	-0.9%

Data source: Global Health Estimates 2021

Panel A9: I-8 - Sub-Saharan Africa

	Age group				
	0-14	15-49	50-69	70+	All ages
Population size (millions)					
2000	305	313	52.5	11.8	682
2010	393	418	69.0	15.6	895
2019	482	544	93.4	20.4	1,140
Rate of change in population size (% per year)					
2000-2010	+2.6%	+2.9%	+2.8%	+2.8%	+2.8%
2010-2019	+2.3%	+3.0%	+3.4%	+3.0%	+2.7%
2000-2019	+2.4%	+3.0%	+3.1%	+2.9%	+2.7%
Deaths (thousands)					
2000	3,860	1,290	565	352	6,070
2010	3,120	1,090	509	362	5,090
2019	2,630	778	447	353	4,210
Rate of change in deaths (% per year)					
2000-2010	-2.1%	-1.7%	-1.0%	+0.3%	-1.8%
2010-2019	-1.9%	-3.7%	-1.4%	-0.3%	-2.1%
2000-2019	-2.0%	-2.6%	-1.2%	0.0%	-1.9%
Death rate (per 100 000 population per year)					
2000	1,270	413	1,080	2,980	891
2010	795	262	738	2,320	568
2019	546	143	478	1,730	369
Rate of change in death rate (% per year)					
2000-2010	-4.6%	-4.5%	-3.7%	-2.5%	-4.4%
2010-2019	-4.1%	-6.5%	-4.7%	-3.2%	-4.7%
2000-2019	-4.3%	-5.4%	-4.2%	-2.8%	-4.5%

Data source: Global Health Estimates 2021

Panel A10: I-8 - United States

	Age group				
	0-14	15-49	50-69	70+	All ages
Population size (millions)					
2000	59.6	146	51.4	24.9	281
2010	61.3	151	71.3	27.5	311
2019	62.9	157	82.2	35.5	338
Rate of change in population size (% per year)					
2000-2010	+0.3%	+0.4%	+3.3%	+1.0%	+1.0%
2010-2019	+0.3%	+0.4%	+1.6%	+2.9%	+0.9%
2000-2019	+0.3%	+0.4%	+2.5%	+1.9%	+1.0%
Deaths (thousands)					
2000	16.2	15.0	10.0	61.5	103
2010	13.9	7.7	12.9	51.0	85.4
2019	12.0	5.5	15.3	43.8	76.6
Rate of change in deaths (% per year)					
2000-2010	-1.5%	-6.4%	+2.5%	-1.9%	-1.8%
2010-2019	-1.6%	-3.6%	+2.0%	-1.7%	-1.2%
2000-2019	-1.6%	-5.1%	+2.2%	-1.8%	-1.5%
Death rate (per 100 000 population per year)					
2000	27.1	10.3	19.5	247	36.5
2010	22.7	5.1	18.0	185	27.5
2019	19.1	3.5	18.6	123	22.7
Rate of change in death rate (% per year)					
2000-2010	-1.8%	-6.8%	-0.8%	-2.9%	-2.8%
2010-2019	-1.9%	-4.0%	+0.4%	-4.4%	-2.1%
2000-2019	-1.8%	-5.5%	-0.2%	-3.6%	-2.5%

Data source: Global Health Estimates 2021

Panel A11: I-8 - Western Pacific and Southeast Asia

	Age group				
	0-14	15-49	50-69	70+	All ages
Population size (millions)					
2000	276	492	127	38.9	934
2010	275	549	163	56.0	1,040
2019	272	582	205	74.4	1,130
Rate of change in population size (% per year)					
2000-2010	0.0%	+1.1%	+2.5%	+3.7%	+1.1%
2010-2019	-0.1%	+0.6%	+2.6%	+3.2%	+0.9%
2000-2019	-0.1%	+0.9%	+2.5%	+3.5%	+1.0%
Deaths (thousands)					
2000	850	295	249	359	1,750
2010	476	228	217	422	1,340
2019	295	165	193	451	1,100
Rate of change in deaths (% per year)					
2000-2010	-5.6%	-2.5%	-1.4%	+1.6%	-2.6%
2010-2019	-5.2%	-3.6%	-1.3%	+0.7%	-2.2%
2000-2019	-5.4%	-3.0%	-1.3%	+1.2%	-2.4%
Death rate (per 100 000 population per year)					
2000	308	60.1	195	922	188
2010	173	41.6	133	754	129
2019	109	28.3	94.0	606	97.4
Rate of change in death rate (% per year)					
2000-2010	-5.6%	-3.6%	-3.7%	-2.0%	-3.7%
2010-2019	-5.1%	-4.2%	-3.8%	-2.4%	-3.1%
2000-2019	-5.3%	-3.9%	-3.8%	-2.2%	-3.4%

Data source: Global Health Estimates 2021

Panel B1: Childhood-cluster diseases - World

	Age group				
	0-14	15-49	50-69	70+	All ages
Population size (millions)					
2000	1,870	3,210	808	269	6,160
2010	1,910	3,670	1,080	355	7,010
2019	2,030	3,930	1,380	451	7,800
Rate of change in population size (% per year)					
2000-2010	+0.2%	+1.3%	+2.9%	+2.8%	+1.3%
2010-2019	+0.7%	+0.8%	+2.9%	+2.7%	+1.2%
2000-2019	+0.4%	+1.1%	+2.9%	+2.7%	+1.2%
Deaths (thousands)					
2000	1,020	38.2	10.7	5.2	1,080
2010	450	24.4	7.1	3.6	485
2019	267	16.9	5.2	2.5	291
Rate of change in deaths (% per year)					
2000-2010	-7.9%	-4.4%	-4.0%	-3.5%	-7.7%
2010-2019	-5.6%	-4.0%	-3.4%	-3.9%	-5.5%
2000-2019	-6.8%	-4.2%	-3.7%	-3.7%	-6.7%
Death rate (per 100 000 population per year)					
2000	54.7	1.2	1.3	1.9	17.5
2010	23.5	0.7	0.7	1.0	6.9
2019	13.2	0.4	0.4	0.6	3.7
Rate of change in death rate (% per year)					
2000-2010	-8.1%	-5.6%	-6.7%	-6.1%	-8.9%
2010-2019	-6.3%	-4.7%	-6.0%	-6.4%	-6.6%
2000-2019	-7.2%	-5.2%	-6.4%	-6.3%	-7.8%

Data source: Global Health Estimates 2021

Panel B2: Childhood-cluster diseases - Central and Eastern Europe

	Age group				
	0-14	15-49	50-69	70+	All ages
Population size (millions)					
2000	63.2	181	70.2	29.2	343
2010	50.2	169	78.6	34.2	332
2019	54.6	153	85.8	35.2	329
Rate of change in population size (% per year)					
2000-2010	-2.3%	-0.7%	+1.1%	+1.6%	-0.3%
2010-2019	+0.9%	-1.1%	+1.0%	+0.3%	-0.1%
2000-2019	-0.8%	-0.9%	+1.1%	+1.0%	-0.2%
Deaths (thousands)					
2000	0.5	0.3	<0.1	<0.1	0.9
2010	<0.1	<0.1	<0.1	<0.1	0.1
2019	<0.1	<0.1	<0.1	<0.1	0.1
Rate of change in deaths (% per year)					
2000-2010	-20%	-18%	.	.	-18%
2010-2019	.	.	.	.	+2.1%
2000-2019	-8.7%	-9.6%	.	.	-9.1%
Death rate (per 100 000 population per year)					
2000	0.8	0.2	<0.1	0.1	0.3
2010	0.1	<0.1	<0.1	<0.1	<0.1
2019	0.2	<0.1	<0.1	<0.1	<0.1
Rate of change in death rate (% per year)					
2000-2010	-18%	-18%	.	-11%	-18%
2010-2019	+4.2%	.	.	.	.
2000-2019	-8.0%	-8.8%	.	-8.3%	-8.9%

Data source: Global Health Estimates 2021

Panel B3: Childhood-cluster diseases - Central Asia

	Age group				
	0-14	15-49	50-69	70+	All ages
Population size (millions)					
2000	100	115	21.7	5.4	242
2010	114	153	29.3	7.3	303
2019	131	177	39.7	8.8	357
Rate of change in population size (% per year)					
2000-2010	+1.3%	+2.9%	+3.0%	+3.1%	+2.3%
2010-2019	+1.6%	+1.6%	+3.4%	+2.1%	+1.8%
2000-2019	+1.4%	+2.3%	+3.2%	+2.6%	+2.1%
Deaths (thousands)					
2000	108	3.7	1.1	0.7	114
2010	91.4	7.7	0.8	0.5	100
2019	32.8	3.0	0.5	0.3	36.6
Rate of change in deaths (% per year)					
2000-2010	-1.7%	+7.7%	-3.2%	-2.6%	-1.2%
2010-2019	-11%	-10%	-5.0%	-4.4%	-11%
2000-2019	-6.1%	-1.1%	-4.1%	-3.4%	-5.8%
Death rate (per 100 000 population per year)					
2000	108	3.2	5.2	12.3	47.0
2010	80.5	5.0	2.8	7.0	33.1
2019	25.0	1.7	1.3	3.9	10.3
Rate of change in death rate (% per year)					
2000-2010	-2.9%	+4.6%	-6.0%	-5.5%	-3.4%
2010-2019	-12%	-12%	-8.2%	-6.4%	-12%
2000-2019	-7.4%	-3.4%	-7.0%	-5.9%	-7.7%

Data source: Global Health Estimates 2021

Panel B4: Childhood-cluster diseases - China

	Age group				
	0-14	15-49	50-69	70+	All ages
Population size (millions)					
2000	312	723	180	54.9	1,270
2010	250	767	257	77.6	1,350
2019	259	708	354	103	1,420
Rate of change in population size (% per year)					
2000-2010	-2.2%	+0.6%	+3.6%	+3.5%	+0.6%
2010-2019	+0.4%	-0.9%	+3.6%	+3.1%	+0.6%
2000-2019	-1.0%	-0.1%	+3.6%	+3.3%	+0.6%
Deaths (thousands)					
2000	15.9	3.9	0.4	0.3	20.6
2010	4.5	2.1	0.2	0.2	7.0
2019	1.6	0.4	0.1	<0.1	2.2
Rate of change in deaths (% per year)					
2000-2010	-12%	-6.0%	-6.4%	-5.7%	-10%
2010-2019	-11%	-17%	-6.6%	-7.2%	-12%
2000-2019	-11%	-11%	-6.5%	-6.4%	-11%
Death rate (per 100 000 population per year)					
2000	5.1	0.5	0.2	0.6	1.6
2010	1.8	0.3	<0.1	0.2	0.5
2019	0.6	<0.1	<0.1	<0.1	0.2
Rate of change in death rate (% per year)					
2000-2010	-9.8%	-6.6%	-9.7%	-8.9%	-11%
2010-2019	-11%	-16%	.	-10.0%	-13%
2000-2019	-11%	-11%	-9.8%	-9.4%	-12%

Data source: Global Health Estimates 2021

Panel B5: Childhood-cluster diseases - India

	Age group				
	0-14	15-49	50-69	70+	All ages
Population size (millions)					
2000	376	543	111	27.6	1,060
2010	390	659	157	37.8	1,240
2019	373	757	209	50.5	1,390
Rate of change in population size (% per year)					
2000-2010	+0.4%	+1.9%	+3.6%	+3.2%	+1.6%
2010-2019	-0.5%	+1.6%	+3.2%	+3.3%	+1.2%
2000-2019	0.0%	+1.8%	+3.4%	+3.2%	+1.4%
Deaths (thousands)					
2000	324	5.6	2.4	1.0	333
2010	144	3.3	1.4	0.6	150
2019	33.6	1.5	0.7	0.3	36.1
Rate of change in deaths (% per year)					
2000-2010	-7.8%	-5.2%	-5.3%	-4.6%	-7.7%
2010-2019	-15%	-8.1%	-7.4%	-8.0%	-15%
2000-2019	-11%	-6.6%	-6.3%	-6.2%	-11%
Death rate (per 100 000 population per year)					
2000	86.1	1.0	2.1	3.7	31.4
2010	37.1	0.5	0.9	1.7	12.0
2019	9.0	0.2	0.3	0.6	2.6
Rate of change in death rate (% per year)					
2000-2010	-8.1%	-7.0%	-8.6%	-7.6%	-9.2%
2010-2019	-15%	-9.6%	-10%	-11%	-16%
2000-2019	-11%	-8.2%	-9.4%	-9.2%	-12%

Data source: Global Health Estimates 2021

Panel B6: Childhood-cluster diseases - Latin America and Caribbean

	Age group				
	0-14	15-49	50-69	70+	All ages
Population size (millions)					
2000	166	272	59.4	17.7	515
2010	161	312	83.7	25.4	583
2019	155	337	110	34.6	636
Rate of change in population size (% per year)					
2000-2010	-0.3%	+1.4%	+3.5%	+3.7%	+1.2%
2010-2019	-0.5%	+0.8%	+3.1%	+3.5%	+1.0%
2000-2019	-0.4%	+1.1%	+3.3%	+3.6%	+1.1%
Deaths (thousands)					
2000	3.7	0.2	0.2	0.1	4.2
2010	1.5	0.1	<0.1	<0.1	1.8
2019	1.4	<0.1	<0.1	<0.1	1.6
Rate of change in deaths (% per year)					
2000-2010	-8.8%	-5.8%	-5.9%	-6.0%	-8.4%
2010-2019	-0.7%	-4.2%	.	.	-1.1%
2000-2019	-5.1%	-5.1%	-4.4%	-4.4%	-5.0%
Death rate (per 100 000 population per year)					
2000	2.2	<0.1	0.3	0.7	0.8
2010	0.9	<0.1	0.1	0.2	0.3
2019	0.9	<0.1	<0.1	0.1	0.2
Rate of change in death rate (% per year)					
2000-2010	-8.6%	.	-9.0%	-9.4%	-9.5%
2010-2019	-0.2%	.	-5.6%	-6.0%	-2.1%
2000-2019	-4.7%	.	-7.4%	-7.8%	-6.1%

Data source: Global Health Estimates 2021

Panel B7: Childhood-cluster diseases - Middle East and North Africa

	Age group				
	0-14	15-49	50-69	70+	All ages
Population size (millions)					
2000	135	200	36.9	9.6	381
2010	140	259	53.6	13.8	467
2019	163	296	76.1	18.3	553
Rate of change in population size (% per year)					
2000-2010	+0.4%	+2.6%	+3.8%	+3.7%	+2.0%
2010-2019	+1.7%	+1.5%	+4.0%	+3.2%	+1.9%
2000-2019	+1.0%	+2.1%	+3.9%	+3.5%	+2.0%
Deaths (thousands)					
2000	17.3	3.1	0.1	<0.1	20.6
2010	5.3	0.6	<0.1	<0.1	6.0
2019	6.5	0.3	<0.1	<0.1	6.9
Rate of change in deaths (% per year)					
2000-2010	-11%	-16%	-3.1%	.	-12%
2010-2019	+2.3%	-6.0%	.	.	+1.6%
2000-2019	-5.0%	-11%	-1.8%	.	-5.6%
Death rate (per 100 000 population per year)					
2000	12.8	1.6	0.3	0.6	5.4
2010	3.8	0.2	0.1	0.3	1.3
2019	4.0	0.1	<0.1	0.2	1.2
Rate of change in death rate (% per year)					
2000-2010	-12%	-18%	-6.6%	-5.3%	-13%
2010-2019	+0.6%	-7.4%	-4.1%	-4.8%	-0.3%
2000-2019	-6.0%	-13%	-5.4%	-5.0%	-7.4%

Data source: Global Health Estimates 2021

Panel B8: Childhood-cluster diseases - North Atlantic

	Age group				
	0-14	15-49	50-69	70+	All ages
Population size (millions)					
2000	72.0	209	93.2	47.7	422
2010	71.3	211	107	57.5	446
2019	72.1	202	122	67.8	464
Rate of change in population size (% per year)					
2000-2010	-0.1%	+0.1%	+1.4%	+1.9%	+0.6%
2010-2019	+0.1%	-0.4%	+1.5%	+1.9%	+0.4%
2000-2019	0.0%	-0.2%	+1.4%	+1.9%	+0.5%
Deaths (thousands)					
2000	<0.1	<0.1	<0.1	<0.1	0.1
2010	<0.1	<0.1	<0.1	<0.1	<0.1
2019	<0.1	<0.1	<0.1	<0.1	<0.1
Rate of change in deaths (% per year)					
2000-2010	.	.	.	.	-5.0%
2010-2019	.	.	.	.	.
2000-2019	.	.	.	.	-2.3%
Death rate (per 100 000 population per year)					
2000	<0.1	<0.1	<0.1	0.2	<0.1
2010	<0.1	<0.1	<0.1	<0.1	<0.1
2019	<0.1	<0.1	<0.1	<0.1	<0.1
Rate of change in death rate (% per year)					
2000-2010	.	.	.	-7.2%	.
2010-2019	.	.	.	.	.
2000-2019	.	.	.	-3.9%	.

Data source: Global Health Estimates 2021

Panel B9: Childhood-cluster diseases - Sub-Saharan Africa

	Age group				
	0-14	15-49	50-69	70+	All ages
Population size (millions)					
2000	305	313	52.5	11.8	682
2010	393	418	69.0	15.6	895
2019	482	544	93.4	20.4	1,140
Rate of change in population size (% per year)					
2000-2010	+2.6%	+2.9%	+2.8%	+2.8%	+2.8%
2010-2019	+2.3%	+3.0%	+3.4%	+3.0%	+2.7%
2000-2019	+2.4%	+3.0%	+3.1%	+2.9%	+2.7%
Deaths (thousands)					
2000	477	12.8	3.3	1.4	495
2010	180	6.2	2.3	0.9	190
2019	178	8.8	2.0	0.8	190
Rate of change in deaths (% per year)					
2000-2010	-9.3%	-7.0%	-3.5%	-3.6%	-9.1%
2010-2019	-0.1%	+4.0%	-1.5%	-1.9%	0.0%
2000-2019	-5.0%	-1.9%	-2.6%	-2.8%	-4.9%
Death rate (per 100 000 population per year)					
2000	157	4.1	6.3	11.5	72.6
2010	45.9	1.5	3.3	6.0	21.2
2019	37.0	1.6	2.1	3.9	16.7
Rate of change in death rate (% per year)					
2000-2010	-12%	-9.6%	-6.1%	-6.2%	-12%
2010-2019	-2.4%	+0.9%	-4.8%	-4.8%	-2.6%
2000-2019	-7.3%	-4.8%	-5.5%	-5.6%	-7.4%

Data source: Global Health Estimates 2021

Panel B10: Childhood-cluster diseases - United States

	Age group				
	0-14	15-49	50-69	70+	All ages
Population size (millions)					
2000	59.6	146	51.4	24.9	281
2010	61.3	151	71.3	27.5	311
2019	62.9	157	82.2	35.5	338
Rate of change in population size (% per year)					
2000-2010	+0.3%	+0.4%	+3.3%	+1.0%	+1.0%
2010-2019	+0.3%	+0.4%	+1.6%	+2.9%	+0.9%
2000-2019	+0.3%	+0.4%	+2.5%	+1.9%	+1.0%
Deaths (thousands)					
2000	<0.1	<0.1	<0.1	<0.1	<0.1
2010	<0.1	<0.1	<0.1	<0.1	<0.1
2019	<0.1	<0.1	<0.1	<0.1	<0.1
Rate of change in deaths (% per year)					
2000-2010	.	.	.	.	.
2010-2019	.	.	.	.	.
2000-2019	.	.	.	.	.
Death rate (per 100 000 population per year)					
2000	<0.1	<0.1	<0.1	<0.1	<0.1
2010	<0.1	<0.1	<0.1	<0.1	<0.1
2019	<0.1	<0.1	<0.1	<0.1	<0.1
Rate of change in death rate (% per year)					
2000-2010	.	.	.	.	.
2010-2019	.	.	.	.	.
2000-2019	.	.	.	.	.

Data source: Global Health Estimates 2021

Panel B11: Childhood-cluster diseases - Western Pacific and Southeast Asia

	Age group				
	0-14	15-49	50-69	70+	All ages
Population size (millions)					
2000	276	492	127	38.9	934
2010	275	549	163	56.0	1,040
2019	272	582	205	74.4	1,130
Rate of change in population size (% per year)					
2000-2010	0.0%	+1.1%	+2.5%	+3.7%	+1.1%
2010-2019	-0.1%	+0.6%	+2.6%	+3.2%	+0.9%
2000-2019	-0.1%	+0.9%	+2.5%	+3.5%	+1.0%
Deaths (thousands)					
2000	77.1	8.6	3.1	1.5	90.2
2010	22.1	4.3	2.2	1.2	29.7
2019	12.4	2.7	1.7	0.9	17.7
Rate of change in deaths (% per year)					
2000-2010	-12%	-6.6%	-3.6%	-2.3%	-11%
2010-2019	-6.2%	-5.0%	-2.6%	-3.6%	-5.6%
2000-2019	-9.2%	-5.8%	-3.1%	-2.9%	-8.2%
Death rate (per 100 000 population per year)					
2000	27.9	1.7	2.4	3.8	9.7
2010	8.0	0.8	1.3	2.1	2.8
2019	4.5	0.5	0.8	1.1	1.6
Rate of change in death rate (% per year)					
2000-2010	-12%	-7.6%	-5.9%	-5.8%	-11%
2010-2019	-6.1%	-5.6%	-5.0%	-6.6%	-6.5%
2000-2019	-9.1%	-6.7%	-5.5%	-6.1%	-9.2%

Data source: Global Health Estimates 2021

Panel C1: Diarrheal diseases - World

	Age group				
	0-14	15-49	50-69	70+	All ages
Population size (millions)					
2000	1,870	3,210	808	269	6,160
2010	1,910	3,670	1,080	355	7,010
2019	2,030	3,930	1,380	451	7,800
Rate of change in population size (% per year)					
2000-2010	+0.2%	+1.3%	+2.9%	+2.8%	+1.3%
2010-2019	+0.7%	+0.8%	+2.9%	+2.7%	+1.2%
2000-2019	+0.4%	+1.1%	+2.9%	+2.7%	+1.2%
Deaths (thousands)					
2000	1,310	163	282	550	2,300
2010	803	144	240	542	1,730
2019	527	112	201	483	1,320
Rate of change in deaths (% per year)					
2000-2010	-4.8%	-1.2%	-1.6%	-0.1%	-2.8%
2010-2019	-4.6%	-2.8%	-2.0%	-1.3%	-2.9%
2000-2019	-4.7%	-2.0%	-1.8%	-0.7%	-2.9%
Death rate (per 100 000 population per year)					
2000	69.9	5.1	34.9	204	37.4
2010	42.0	3.9	22.4	153	24.7
2019	26.0	2.8	14.5	107	17.0
Rate of change in death rate (% per year)					
2000-2010	-5.0%	-2.5%	-4.4%	-2.9%	-4.1%
2010-2019	-5.2%	-3.5%	-4.7%	-3.9%	-4.1%
2000-2019	-5.1%	-3.0%	-4.5%	-3.3%	-4.1%

Data source: Global Health Estimates 2021

Panel C2: Diarrheal diseases - Central and Eastern Europe

	Age group				
	0-14	15-49	50-69	70+	All ages
Population size (millions)					
2000	63.2	181	70.2	29.2	343
2010	50.2	169	78.6	34.2	332
2019	54.6	153	85.8	35.2	329
Rate of change in population size (% per year)					
2000-2010	-2.3%	-0.7%	+1.1%	+1.6%	-0.3%
2010-2019	+0.9%	-1.1%	+1.0%	+0.3%	-0.1%
2000-2019	-0.8%	-0.9%	+1.1%	+1.0%	-0.2%
Deaths (thousands)					
2000	1.6	0.3	0.4	0.3	2.5
2010	0.4	0.1	0.2	0.5	1.3
2019	0.1	0.1	0.5	1.9	2.7
Rate of change in deaths (% per year)					
2000-2010	-12%	-10%	-4.7%	+5.4%	-6.4%
2010-2019	-12%	+0.7%	+9.7%	+15%	+8.3%
2000-2019	-12%	-5.3%	+1.9%	+9.9%	+0.3%
Death rate (per 100 000 population per year)					
2000	2.4	0.2	0.5	1.1	0.7
2010	0.9	<0.1	0.3	1.6	0.4
2019	0.3	<0.1	0.6	5.5	0.8
Rate of change in death rate (% per year)					
2000-2010	-9.8%	-9.8%	-5.8%	+3.7%	-6.1%
2010-2019	-13%	.	+8.6%	+15%	+8.4%
2000-2019	-11%	-4.5%	+0.8%	+8.8%	+0.5%

Data source: Global Health Estimates 2021

Panel C3: Diarrheal diseases - Central Asia

	Age group				
	0-14	15-49	50-69	70+	All ages
Population size (millions)					
2000	100	115	21.7	5.4	242
2010	114	153	29.3	7.3	303
2019	131	177	39.7	8.8	357
Rate of change in population size (% per year)					
2000-2010	+1.3%	+2.9%	+3.0%	+3.1%	+2.3%
2010-2019	+1.6%	+1.6%	+3.4%	+2.1%	+1.8%
2000-2019	+1.4%	+2.3%	+3.2%	+2.6%	+2.1%
Deaths (thousands)					
2000	99.3	7.0	10.1	21.9	138
2010	65.6	5.6	7.9	18.7	97.7
2019	40.9	4.4	6.2	15.7	67.2
Rate of change in deaths (% per year)					
2000-2010	-4.1%	-2.2%	-2.5%	-1.6%	-3.4%
2010-2019	-5.1%	-2.6%	-2.6%	-1.9%	-4.1%
2000-2019	-4.6%	-2.4%	-2.5%	-1.7%	-3.7%
Death rate (per 100 000 population per year)					
2000	99.3	6.1	46.4	404	57.2
2010	57.8	3.7	26.8	255	32.2
2019	31.2	2.5	15.6	177	18.8
Rate of change in death rate (% per year)					
2000-2010	-5.3%	-5.0%	-5.3%	-4.5%	-5.6%
2010-2019	-6.6%	-4.1%	-5.8%	-4.0%	-5.8%
2000-2019	-5.9%	-4.6%	-5.6%	-4.2%	-5.7%

Data source: Global Health Estimates 2021

Panel C4: Diarrheal diseases - China

	Age group				
	0-14	15-49	50-69	70+	All ages
Population size (millions)					
2000	312	723	180	54.9	1,270
2010	250	767	257	77.6	1,350
2019	259	708	354	103	1,420
Rate of change in population size (% per year)					
2000-2010	-2.2%	+0.6%	+3.6%	+3.5%	+0.6%
2010-2019	+0.4%	-0.9%	+3.6%	+3.1%	+0.6%
2000-2019	-1.0%	-0.1%	+3.6%	+3.3%	+0.6%
Deaths (thousands)					
2000	35.9	1.8	2.0	5.1	44.9
2010	10.5	0.7	0.9	2.7	14.9
2019	4.9	0.4	0.8	2.2	8.3
Rate of change in deaths (% per year)					
2000-2010	-12%	-9.0%	-7.5%	-6.2%	-10%
2010-2019	-8.1%	-6.2%	-1.9%	-2.1%	-6.2%
2000-2019	-9.9%	-7.7%	-4.9%	-4.3%	-8.5%
Death rate (per 100 000 population per year)					
2000	11.5	0.3	1.1	9.3	3.5
2010	4.2	<0.1	0.4	3.5	1.1
2019	1.9	<0.1	0.2	2.2	0.6
Rate of change in death rate (% per year)					
2000-2010	-9.6%	-9.5%	-11%	-9.4%	-11%
2010-2019	-8.5%	.	-5.4%	-5.1%	-6.8%
2000-2019	-9.1%	-7.6%	-8.2%	-7.4%	-9.0%

Data source: Global Health Estimates 2021

Panel C5: Diarrheal diseases - India

	Age group				
	0-14	15-49	50-69	70+	All ages
Population size (millions)					
2000	376	543	111	27.6	1,060
2010	390	659	157	37.8	1,240
2019	373	757	209	50.5	1,390
Rate of change in population size (% per year)					
2000-2010	+0.4%	+1.9%	+3.6%	+3.2%	+1.6%
2010-2019	-0.5%	+1.6%	+3.2%	+3.3%	+1.2%
2000-2019	0.0%	+1.8%	+3.4%	+3.2%	+1.4%
Deaths (thousands)					
2000	363	85.4	170	370	988
2010	167	68.8	141	354	730
2019	70.4	44.4	111	301	527
Rate of change in deaths (% per year)					
2000-2010	-7.5%	-2.1%	-1.9%	-0.4%	-3.0%
2010-2019	-9.1%	-4.7%	-2.6%	-1.8%	-3.6%
2000-2019	-8.3%	-3.4%	-2.2%	-1.1%	-3.3%
Death rate (per 100 000 population per year)					
2000	96.6	15.7	153	1,340	93.4
2010	42.8	10.4	89.5	936	58.7
2019	18.9	5.9	53.2	597	37.9
Rate of change in death rate (% per year)					
2000-2010	-7.8%	-4.0%	-5.2%	-3.5%	-4.5%
2010-2019	-8.7%	-6.2%	-5.6%	-4.9%	-4.7%
2000-2019	-8.2%	-5.1%	-5.4%	-4.2%	-4.6%

Data source: Global Health Estimates 2021

Panel C6: Diarrheal diseases - Latin America and Caribbean

	Age group				
	0-14	15-49	50-69	70+	All ages
Population size (millions)					
2000	166	272	59.4	17.7	515
2010	161	312	83.7	25.4	583
2019	155	337	110	34.6	636
Rate of change in population size (% per year)					
2000-2010	-0.3%	+1.4%	+3.5%	+3.7%	+1.2%
2010-2019	-0.5%	+0.8%	+3.1%	+3.5%	+1.0%
2000-2019	-0.4%	+1.1%	+3.3%	+3.6%	+1.1%
Deaths (thousands)					
2000	33.9	2.6	3.1	6.7	46.3
2010	10.3	1.9	2.8	7.6	22.6
2019	7.3	1.8	3.6	9.7	22.3
Rate of change in deaths (% per year)					
2000-2010	-11%	-2.9%	-1.1%	+1.2%	-6.9%
2010-2019	-3.8%	-0.9%	+3.0%	+2.8%	-0.1%
2000-2019	-7.8%	-2.0%	+0.8%	+1.9%	-3.8%
Death rate (per 100 000 population per year)					
2000	20.4	1.0	5.2	38.1	9.0
2010	6.4	0.6	3.3	29.9	3.9
2019	4.7	0.5	3.3	28.0	3.5
Rate of change in death rate (% per year)					
2000-2010	-11%	-4.2%	-4.4%	-2.4%	-8.1%
2010-2019	-3.3%	-1.7%	-0.1%	-0.7%	-1.1%
2000-2019	-7.4%	-3.1%	-2.4%	-1.6%	-4.8%

Data source: Global Health Estimates 2021

Panel C7: Diarrheal diseases - Middle East and North Africa

	Age group				
	0-14	15-49	50-69	70+	All ages
Population size (millions)					
2000	135	200	36.9	9.6	381
2010	140	259	53.6	13.8	467
2019	163	296	76.1	18.3	553
Rate of change in population size (% per year)					
2000-2010	+0.4%	+2.6%	+3.8%	+3.7%	+2.0%
2010-2019	+1.7%	+1.5%	+4.0%	+3.2%	+1.9%
2000-2019	+1.0%	+2.1%	+3.9%	+3.5%	+2.0%
Deaths (thousands)					
2000	37.2	0.7	0.7	1.6	40.2
2010	15.6	0.5	0.6	1.7	18.4
2019	8.0	0.5	0.6	1.9	11.1
Rate of change in deaths (% per year)					
2000-2010	-8.3%	-2.9%	-1.8%	+0.5%	-7.5%
2010-2019	-7.1%	-0.4%	+1.3%	+1.3%	-5.5%
2000-2019	-7.8%	-1.7%	-0.3%	+0.9%	-6.6%
Death rate (per 100 000 population per year)					
2000	27.6	0.3	1.8	16.9	10.6
2010	11.2	0.2	1.1	12.4	3.9
2019	5.0	0.2	0.8	10.4	2.0
Rate of change in death rate (% per year)					
2000-2010	-8.7%	-5.4%	-5.4%	-3.1%	-9.4%
2010-2019	-8.6%	-1.9%	-2.6%	-1.9%	-7.3%
2000-2019	-8.7%	-3.7%	-4.1%	-2.5%	-8.4%

Data source: Global Health Estimates 2021

Panel C8: Diarrheal diseases - North Atlantic

	Age group				
	0-14	15-49	50-69	70+	All ages
Population size (millions)					
2000	72.0	209	93.2	47.7	422
2010	71.3	211	107	57.5	446
2019	72.1	202	122	67.8	464
Rate of change in population size (% per year)					
2000-2010	-0.1%	+0.1%	+1.4%	+1.9%	+0.6%
2010-2019	+0.1%	-0.4%	+1.5%	+1.9%	+0.4%
2000-2019	0.0%	-0.2%	+1.4%	+1.9%	+0.5%
Deaths (thousands)					
2000	0.2	<0.1	0.2	2.5	3.0
2010	<0.1	0.1	0.8	9.9	11.0
2019	<0.1	0.2	1.2	12.2	13.7
Rate of change in deaths (% per year)					
2000-2010	-6.7%	+8.1%	+12%	+15%	+14%
2010-2019	.	+2.4%	+4.4%	+2.3%	+2.5%
2000-2019	-2.8%	+5.3%	+8.6%	+8.7%	+8.3%
Death rate (per 100 000 population per year)					
2000	0.2	<0.1	0.3	5.3	0.7
2010	0.1	<0.1	0.7	17.3	2.5
2019	0.1	<0.1	0.9	18.0	2.9
Rate of change in death rate (% per year)					
2000-2010	-6.6%	.	+11%	+13%	+13%
2010-2019	+1.6%	.	+2.9%	+0.4%	+2.0%
2000-2019	-2.8%	.	+7.0%	+6.7%	+7.8%

Data source: Global Health Estimates 2021

Panel C9: Diarrheal diseases - Sub-Saharan Africa

	Age group				
	0-14	15-49	50-69	70+	All ages
Population size (millions)					
2000	305	313	52.5	11.8	682
2010	393	418	69.0	15.6	895
2019	482	544	93.4	20.4	1,140
Rate of change in population size (% per year)					
2000-2010	+2.6%	+2.9%	+2.8%	+2.8%	+2.8%
2010-2019	+2.3%	+3.0%	+3.4%	+3.0%	+2.7%
2000-2019	+2.4%	+3.0%	+3.1%	+2.9%	+2.7%
Deaths (thousands)					
2000	597	48.4	66.0	76.1	788
2010	466	55.0	63.4	76.3	661
2019	360	51.7	55.4	68.2	535
Rate of change in deaths (% per year)					
2000-2010	-2.4%	+1.3%	-0.4%	0.0%	-1.7%
2010-2019	-2.8%	-0.7%	-1.5%	-1.2%	-2.3%
2000-2019	-2.6%	+0.3%	-0.9%	-0.6%	-2.0%
Death rate (per 100 000 population per year)					
2000	196	15.5	126	645	116
2010	119	13.2	91.9	490	73.8
2019	74.7	9.5	59.3	335	47.0
Rate of change in death rate (% per year)					
2000-2010	-4.9%	-1.6%	-3.1%	-2.7%	-4.4%
2010-2019	-5.0%	-3.6%	-4.7%	-4.1%	-4.9%
2000-2019	-4.9%	-2.5%	-3.9%	-3.4%	-4.6%

Data source: Global Health Estimates 2021

Panel C10: Diarrheal diseases - United States

	Age group				
	0-14	15-49	50-69	70+	All ages
Population size (millions)					
2000	59.6	146	51.4	24.9	281
2010	61.3	151	71.3	27.5	311
2019	62.9	157	82.2	35.5	338
Rate of change in population size (% per year)					
2000-2010	+0.3%	+0.4%	+3.3%	+1.0%	+1.0%
2010-2019	+0.3%	+0.4%	+1.6%	+2.9%	+0.9%
2000-2019	+0.3%	+0.4%	+2.5%	+1.9%	+1.0%
Deaths (thousands)					
2000	0.6	<0.1	0.2	1.2	2.0
2010	0.6	0.2	1.4	8.6	10.8
2019	0.4	0.2	1.6	5.4	7.6
Rate of change in deaths (% per year)					
2000-2010	-0.7%	+18%	+23%	+22%	+18%
2010-2019	-5.2%	+2.4%	+1.4%	-5.0%	-3.8%
2000-2019	-2.8%	+11%	+12%	+8.4%	+7.3%
Death rate (per 100 000 population per year)					
2000	1.0	<0.1	0.3	4.7	0.7
2010	1.0	0.1	2.0	31.2	3.5
2019	0.6	0.2	1.9	15.3	2.3
Rate of change in death rate (% per year)					
2000-2010	-0.9%	+18%	+19%	+21%	+17%
2010-2019	-5.4%	+2.0%	-0.2%	-7.6%	-4.7%
2000-2019	-3.1%	+10%	+9.5%	+6.4%	+6.3%

Data source: Global Health Estimates 2021

Panel C11: Diarrheal diseases - Western Pacific and Southeast Asia

	Age group				
	0-14	15-49	50-69	70+	All ages
Population size (millions)					
2000	276	492	127	38.9	934
2010	275	549	163	56.0	1,040
2019	272	582	205	74.4	1,130
Rate of change in population size (% per year)					
2000-2010	0.0%	+1.1%	+2.5%	+3.7%	+1.1%
2010-2019	-0.1%	+0.6%	+2.6%	+3.2%	+0.9%
2000-2019	-0.1%	+0.9%	+2.5%	+3.5%	+1.0%
Deaths (thousands)					
2000	139	16.6	29.8	65.3	250
2010	66.8	11.1	21.9	61.7	161
2019	35.0	8.3	20.2	64.4	128
Rate of change in deaths (% per year)					
2000-2010	-7.0%	-4.0%	-3.0%	-0.6%	-4.3%
2010-2019	-6.9%	-3.1%	-0.9%	+0.5%	-2.5%
2000-2019	-7.0%	-3.6%	-2.0%	-0.1%	-3.5%
Death rate (per 100 000 population per year)					
2000	50.2	3.4	23.4	168	26.8
2010	24.3	2.0	13.5	110	15.5
2019	12.9	1.4	9.9	86.6	11.3
Rate of change in death rate (% per year)					
2000-2010	-7.0%	-5.0%	-5.4%	-4.1%	-5.3%
2010-2019	-6.8%	-3.8%	-3.4%	-2.6%	-3.4%
2000-2019	-6.9%	-4.4%	-4.4%	-3.4%	-4.4%

Data source: Global Health Estimates 2021

Panel D1: HIV/AIDS - World

	Age group				
	0-14	15-49	50-69	70+	All ages
Population size (millions)					
2000	1,870	3,210	808	269	6,160
2010	1,910	3,670	1,080	355	7,010
2019	2,030	3,930	1,380	451	7,800
Rate of change in population size (% per year)					
2000-2010	+0.2%	+1.3%	+2.9%	+2.8%	+1.3%
2010-2019	+0.7%	+0.8%	+2.9%	+2.7%	+1.2%
2000-2019	+0.4%	+1.1%	+2.9%	+2.7%	+1.2%
Deaths (thousands)					
2000	328	1,030	242	28.0	1,630
2010	231	802	196	19.8	1,250
2019	104	460	139	11.8	715
Rate of change in deaths (% per year)					
2000-2010	-3.5%	-2.5%	-2.1%	-3.4%	-2.6%
2010-2019	-8.5%	-6.0%	-3.7%	-5.6%	-6.0%
2000-2019	-5.9%	-4.2%	-2.9%	-4.5%	-4.3%
Death rate (per 100 000 population per year)					
2000	17.5	32.2	30.0	10.4	26.5
2010	12.1	21.9	18.2	5.6	17.8
2019	5.1	11.7	10.0	2.6	9.2
Rate of change in death rate (% per year)					
2000-2010	-3.7%	-3.8%	-4.9%	-6.0%	-3.9%
2010-2019	-9.1%	-6.7%	-6.4%	-8.1%	-7.1%
2000-2019	-6.3%	-5.2%	-5.6%	-7.0%	-5.4%

Data source: Global Health Estimates 2021

Panel D2: HIV/AIDS - Central and Eastern Europe

	Age group				
	0-14	15-49	50-69	70+	All ages
Population size (millions)					
2000	63.2	181	70.2	29.2	343
2010	50.2	169	78.6	34.2	332
2019	54.6	153	85.8	35.2	329
Rate of change in population size (% per year)					
2000-2010	-2.3%	-0.7%	+1.1%	+1.6%	-0.3%
2010-2019	+0.9%	-1.1%	+1.0%	+0.3%	-0.1%
2000-2019	-0.8%	-0.9%	+1.1%	+1.0%	-0.2%
Deaths (thousands)					
2000	1.5	14.1	1.8	<0.1	17.4
2010	0.7	24.5	5.0	0.2	30.4
2019	1.0	27.1	5.6	0.2	33.8
Rate of change in deaths (% per year)					
2000-2010	-7.1%	+5.7%	+11%	+16%	+5.7%
2010-2019	+3.0%	+1.1%	+1.3%	-0.5%	+1.2%
2000-2019	-2.4%	+3.5%	+6.2%	+7.9%	+3.6%
Death rate (per 100 000 population per year)					
2000	2.4	7.8	2.5	0.1	5.1
2010	1.5	14.5	6.3	0.5	9.2
2019	1.8	17.6	6.5	0.5	10.3
Rate of change in death rate (% per year)					
2000-2010	-4.9%	+6.4%	+9.5%	+14%	+6.1%
2010-2019	+2.1%	+2.2%	+0.4%	-0.9%	+1.3%
2000-2019	-1.7%	+4.4%	+5.1%	+6.8%	+3.8%

Data source: Global Health Estimates 2021

Panel D3: HIV/AIDS - Central Asia

	Age group				
	0-14	15-49	50-69	70+	All ages
Population size (millions)					
2000	100	115	21.7	5.4	242
2010	114	153	29.3	7.3	303
2019	131	177	39.7	8.8	357
Rate of change in population size (% per year)					
2000-2010	+1.3%	+2.9%	+3.0%	+3.1%	+2.3%
2010-2019	+1.6%	+1.6%	+3.4%	+2.1%	+1.8%
2000-2019	+1.4%	+2.3%	+3.2%	+2.6%	+2.1%
Deaths (thousands)					
2000	<0.1	0.3	<0.1	<0.1	0.5
2010	0.8	3.8	1.0	<0.1	5.5
2019	1.4	10.3	2.8	<0.1	14.5
Rate of change in deaths (% per year)					
2000-2010	+26%	+27%	+31%	.	+27%
2010-2019	+6.8%	+12%	+13%	.	+11%
2000-2019	+16%	+20%	+22%	.	+20%
Death rate (per 100 000 population per year)					
2000	<0.1	0.3	0.3	<0.1	0.2
2010	0.7	2.4	3.3	0.4	1.8
2019	1.1	5.8	7.0	1.0	4.1
Rate of change in death rate (% per year)					
2000-2010	+24%	+23%	+27%	+23%	+25%
2010-2019	+5.1%	+10%	+8.8%	+12%	+9.4%
2000-2019	+15%	+17%	+18%	+17%	+17%

Data source: Global Health Estimates 2021

Panel D4: HIV/AIDS - China

	Age group				
	0-14	15-49	50-69	70+	All ages
Population size (millions)					
2000	312	723	180	54.9	1,270
2010	250	767	257	77.6	1,350
2019	259	708	354	103	1,420
Rate of change in population size (% per year)					
2000-2010	-2.2%	+0.6%	+3.6%	+3.5%	+0.6%
2010-2019	+0.4%	-0.9%	+3.6%	+3.1%	+0.6%
2000-2019	-1.0%	-0.1%	+3.6%	+3.3%	+0.6%
Deaths (thousands)					
2000	0.9	22.2	4.5	0.2	27.8
2010	0.9	42.1	10.6	0.5	54.1
2019	0.8	24.8	11.0	0.6	37.2
Rate of change in deaths (% per year)					
2000-2010	+0.1%	+6.6%	+9.0%	+9.2%	+6.9%
2010-2019	-2.2%	-5.7%	+0.5%	+0.3%	-4.1%
2000-2019	-1.0%	+0.6%	+4.9%	+4.9%	+1.5%
Death rate (per 100 000 population per year)					
2000	0.3	3.1	2.5	0.4	2.2
2010	0.4	5.5	4.1	0.7	4.0
2019	0.3	3.5	3.1	0.5	2.6
Rate of change in death rate (% per year)					
2000-2010	+2.3%	+6.0%	+5.2%	+5.5%	+6.2%
2010-2019	-2.6%	-4.9%	-3.0%	-2.8%	-4.6%
2000-2019	0.0%	+0.7%	+1.2%	+1.5%	+0.9%

Data source: Global Health Estimates 2021

Panel D5: HIV/AIDS - India

	Age group				
	0-14	15-49	50-69	70+	All ages
Population size (millions)					
2000	376	543	111	27.6	1,060
2010	390	659	157	37.8	1,240
2019	373	757	209	50.5	1,390
Rate of change in population size (% per year)					
2000-2010	+0.4%	+1.9%	+3.6%	+3.2%	+1.6%
2010-2019	-0.5%	+1.6%	+3.2%	+3.3%	+1.2%
2000-2019	0.0%	+1.8%	+3.4%	+3.2%	+1.4%
Deaths (thousands)					
2000	21.8	138	51.0	9.5	220
2010	11.5	109	39.4	7.2	167
2019	3.8	27.3	11.9	1.9	44.8
Rate of change in deaths (% per year)					
2000-2010	-6.2%	-2.3%	-2.5%	-2.7%	-2.7%
2010-2019	-12%	-14%	-12%	-14%	-14%
2000-2019	-8.8%	-8.2%	-7.4%	-8.2%	-8.0%
Death rate (per 100 000 population per year)					
2000	5.8	25.4	46.0	34.3	20.8
2010	3.0	16.6	25.1	19.1	13.5
2019	1.0	3.6	5.7	3.7	3.2
Rate of change in death rate (% per year)					
2000-2010	-6.5%	-4.2%	-5.9%	-5.7%	-4.3%
2010-2019	-11%	-16%	-15%	-17%	-15%
2000-2019	-8.7%	-9.8%	-10%	-11%	-9.3%

Data source: Global Health Estimates 2021

Panel D6: HIV/AIDS - Latin America and Caribbean

	Age group				
	0-14	15-49	50-69	70+	All ages
Population size (millions)					
2000	166	272	59.4	17.7	515
2010	161	312	83.7	25.4	583
2019	155	337	110	34.6	636
Rate of change in population size (% per year)					
2000-2010	-0.3%	+1.4%	+3.5%	+3.7%	+1.2%
2010-2019	-0.5%	+0.8%	+3.1%	+3.5%	+1.0%
2000-2019	-0.4%	+1.1%	+3.3%	+3.6%	+1.1%
Deaths (thousands)					
2000	5.9	39.3	7.8	0.7	53.7
2010	3.4	31.9	9.3	0.8	45.4
2019	1.9	25.0	10.8	1.2	38.9
Rate of change in deaths (% per year)					
2000-2010	-5.4%	-2.1%	+1.7%	+1.7%	-1.7%
2010-2019	-6.5%	-2.7%	+1.7%	+5.0%	-1.7%
2000-2019	-5.9%	-2.4%	+1.7%	+3.3%	-1.7%
Death rate (per 100 000 population per year)					
2000	3.6	14.5	13.2	3.8	10.4
2010	2.1	10.2	11.1	3.1	7.8
2019	1.2	7.4	9.8	3.5	6.1
Rate of change in death rate (% per year)					
2000-2010	-5.1%	-3.4%	-1.7%	-1.9%	-2.9%
2010-2019	-6.1%	-3.5%	-1.4%	+1.5%	-2.7%
2000-2019	-5.5%	-3.4%	-1.6%	-0.3%	-2.8%

Data source: Global Health Estimates 2021

Panel D7: HIV/AIDS - Middle East and North Africa

	Age group				
	0-14	15-49	50-69	70+	All ages
Population size (millions)					
2000	135	200	36.9	9.6	381
2010	140	259	53.6	13.8	467
2019	163	296	76.1	18.3	553
Rate of change in population size (% per year)					
2000-2010	+0.4%	+2.6%	+3.8%	+3.7%	+2.0%
2010-2019	+1.7%	+1.5%	+4.0%	+3.2%	+1.9%
2000-2019	+1.0%	+2.1%	+3.9%	+3.5%	+2.0%
Deaths (thousands)					
2000	0.3	1.4	0.2	<0.1	2.0
2010	0.5	4.2	0.8	<0.1	5.6
2019	0.5	3.6	0.9	<0.1	5.1
Rate of change in deaths (% per year)					
2000-2010	+4.3%	+12%	+13%	.	+11%
2010-2019	-0.7%	-1.6%	+1.4%	.	-1.0%
2000-2019	+1.9%	+5.2%	+7.5%	.	+5.1%
Death rate (per 100 000 population per year)					
2000	0.2	0.7	0.6	<0.1	0.5
2010	0.3	1.6	1.5	0.1	1.2
2019	0.3	1.2	1.2	0.1	0.9
Rate of change in death rate (% per year)					
2000-2010	+3.9%	+8.8%	+9.1%	+8.2%	+8.7%
2010-2019	-2.3%	-3.1%	-2.5%	+2.0%	-2.9%
2000-2019	+0.9%	+3.0%	+3.4%	+5.3%	+3.1%

Data source: Global Health Estimates 2021

Panel D8: HIV/AIDS - North Atlantic

	Age group				
	0-14	15-49	50-69	70+	All ages
Population size (millions)					
2000	72.0	209	93.2	47.7	422
2010	71.3	211	107	57.5	446
2019	72.1	202	122	67.8	464
Rate of change in population size (% per year)					
2000-2010	-0.1%	+0.1%	+1.4%	+1.9%	+0.6%
2010-2019	+0.1%	-0.4%	+1.5%	+1.9%	+0.4%
2000-2019	0.0%	-0.2%	+1.4%	+1.9%	+0.5%
Deaths (thousands)					
2000	<0.1	6.1	1.1	0.1	7.3
2010	<0.1	3.0	1.5	0.3	4.8
2019	<0.1	0.8	1.1	0.3	2.1
Rate of change in deaths (% per year)					
2000-2010	.	-7.0%	+3.3%	+7.5%	-4.2%
2010-2019	.	-14%	-4.0%	+0.4%	-8.5%
2000-2019	.	-10%	-0.2%	+4.1%	-6.3%
Death rate (per 100 000 population per year)					
2000	<0.1	2.9	1.2	0.3	1.7
2010	<0.1	1.4	1.4	0.5	1.1
2019	<0.1	0.4	0.9	0.4	0.5
Rate of change in death rate (% per year)					
2000-2010	.	-7.0%	+2.0%	+5.5%	-4.7%
2010-2019	.	-13%	-5.4%	-1.4%	-8.9%
2000-2019	.	-10%	-1.6%	+2.2%	-6.7%

Data source: Global Health Estimates 2021

Panel D9: HIV/AIDS - Sub-Saharan Africa

	Age group				
	0-14	15-49	50-69	70+	All ages
Population size (millions)					
2000	305	313	52.5	11.8	682
2010	393	418	69.0	15.6	895
2019	482	544	93.4	20.4	1,140
Rate of change in population size (% per year)					
2000-2010	+2.6%	+2.9%	+2.8%	+2.8%	+2.8%
2010-2019	+2.3%	+3.0%	+3.4%	+3.0%	+2.7%
2000-2019	+2.4%	+3.0%	+3.1%	+2.9%	+2.7%
Deaths (thousands)					
2000	293	735	168	17.0	1,210
2010	207	517	117	10.2	852
2019	90.9	289	83.0	6.7	469
Rate of change in deaths (% per year)					
2000-2010	-3.4%	-3.4%	-3.5%	-5.0%	-3.5%
2010-2019	-8.7%	-6.3%	-3.8%	-4.5%	-6.4%
2000-2019	-6.0%	-4.8%	-3.6%	-4.8%	-4.9%
Death rate (per 100 000 population per year)					
2000	96.1	235	320	144	178
2010	52.7	124	170	65.3	95.1
2019	18.9	53.1	88.9	32.9	41.2
Rate of change in death rate (% per year)					
2000-2010	-5.8%	-6.2%	-6.1%	-7.6%	-6.1%
2010-2019	-11%	-9.0%	-6.9%	-7.3%	-8.9%
2000-2019	-8.2%	-7.5%	-6.5%	-7.5%	-7.4%

Data source: Global Health Estimates 2021

Panel D10: HIV/AIDS - United States

	Age group				
	0-14	15-49	50-69	70+	All ages
Population size (millions)					
2000	59.6	146	51.4	24.9	281
2010	61.3	151	71.3	27.5	311
2019	62.9	157	82.2	35.5	338
Rate of change in population size (% per year)					
2000-2010	+0.3%	+0.4%	+3.3%	+1.0%	+1.0%
2010-2019	+0.3%	+0.4%	+1.6%	+2.9%	+0.9%
2000-2019	+0.3%	+0.4%	+2.5%	+1.9%	+1.0%
Deaths (thousands)					
2000	<0.1	11.7	3.2	0.2	15.2
2010	<0.1	4.6	3.7	0.3	8.6
2019	<0.1	1.9	2.8	0.6	5.2
Rate of change in deaths (% per year)					
2000-2010	.	-9.0%	+1.4%	+2.9%	-5.6%
2010-2019	.	-9.4%	-2.9%	+6.3%	-5.3%
2000-2019	.	-9.2%	-0.7%	+4.5%	-5.5%
Death rate (per 100 000 population per year)					
2000	0.1	8.0	6.2	1.0	5.4
2010	<0.1	3.0	5.2	1.1	2.8
2019	<0.1	1.2	3.4	1.6	1.6
Rate of change in death rate (% per year)					
2000-2010	-23%	-9.3%	-1.9%	+1.9%	-6.5%
2010-2019	.	-9.9%	-4.4%	+3.4%	-6.2%
2000-2019	-15%	-9.6%	-3.1%	+2.6%	-6.4%

Data source: Global Health Estimates 2021

Panel D11: HIV/AIDS - Western Pacific and Southeast Asia

	Age group				
	0-14	15-49	50-69	70+	All ages
Population size (millions)					
2000	276	492	127	38.9	934
2010	275	549	163	56.0	1,040
2019	272	582	205	74.4	1,130
Rate of change in population size (% per year)					
2000-2010	0.0%	+1.1%	+2.5%	+3.7%	+1.1%
2010-2019	-0.1%	+0.6%	+2.6%	+3.2%	+0.9%
2000-2019	-0.1%	+0.9%	+2.5%	+3.5%	+1.0%
Deaths (thousands)					
2000	4.9	66.0	4.2	0.1	75.2
2010	5.7	61.3	7.1	0.2	74.3
2019	3.8	50.4	8.9	0.2	63.4
Rate of change in deaths (% per year)					
2000-2010	+1.6%	-0.7%	+5.4%	+4.1%	-0.1%
2010-2019	-4.4%	-2.1%	+2.5%	+2.0%	-1.8%
2000-2019	-1.3%	-1.4%	+4.0%	+3.1%	-0.9%
Death rate (per 100 000 population per year)					
2000	1.8	13.4	3.3	0.3	8.1
2010	2.1	11.2	4.4	0.4	7.1
2019	1.4	8.7	4.3	0.3	5.6
Rate of change in death rate (% per year)					
2000-2010	+1.7%	-1.8%	+2.9%	+0.4%	-1.2%
2010-2019	-4.3%	-2.8%	-0.1%	-1.1%	-2.7%
2000-2019	-1.2%	-2.3%	+1.4%	-0.3%	-1.9%

Data source: Global Health Estimates 2021

Panel E1: Lower respiratory infections - World

	Age group				
	0-14	15-49	50-69	70+	All ages
Population size (millions)					
2000	1,870	3,210	808	269	6,160
2010	1,910	3,670	1,080	355	7,010
2019	2,030	3,930	1,380	451	7,800
Rate of change in population size (% per year)					
2000-2010	+0.2%	+1.3%	+2.9%	+2.8%	+1.3%
2010-2019	+0.7%	+0.8%	+2.9%	+2.7%	+1.2%
2000-2019	+0.4%	+1.1%	+2.9%	+2.7%	+1.2%
Deaths (thousands)					
2000	1,660	156	265	781	2,870
2010	1,170	174	308	951	2,600
2019	821	174	402	1,230	2,630
Rate of change in deaths (% per year)					
2000-2010	-3.5%	+1.1%	+1.5%	+2.0%	-1.0%
2010-2019	-3.8%	0.0%	+3.0%	+2.9%	+0.1%
2000-2019	-3.7%	+0.6%	+2.2%	+2.4%	-0.4%
Death rate (per 100 000 population per year)					
2000	88.9	4.9	32.8	290	46.5
2010	61.1	4.7	28.6	268	37.1
2019	40.4	4.4	29.0	273	33.7
Rate of change in death rate (% per year)					
2000-2010	-3.7%	-0.2%	-1.3%	-0.8%	-2.2%
2010-2019	-4.5%	-0.7%	+0.1%	+0.2%	-1.1%
2000-2019	-4.1%	-0.5%	-0.6%	-0.3%	-1.7%

Data source: Global Health Estimates 2021

Panel E2: Lower respiratory infections - Central and Eastern Europe

	Age group				
	0-14	15-49	50-69	70+	All ages
Population size (millions)					
2000	63.2	181	70.2	29.2	343
2010	50.2	169	78.6	34.2	332
2019	54.6	153	85.8	35.2	329
Rate of change in population size (% per year)					
2000-2010	-2.3%	-0.7%	+1.1%	+1.6%	-0.3%
2010-2019	+0.9%	-1.1%	+1.0%	+0.3%	-0.1%
2000-2019	-0.8%	-0.9%	+1.1%	+1.0%	-0.2%
Deaths (thousands)					
2000	7.3	21.5	23.5	26.1	78.4
2010	3.7	18.9	24.3	28.7	75.6
2019	1.8	12.4	24.4	44.7	83.4
Rate of change in deaths (% per year)					
2000-2010	-6.6%	-1.2%	+0.3%	+0.9%	-0.4%
2010-2019	-7.4%	-4.6%	0.0%	+5.1%	+1.1%
2000-2019	-7.0%	-2.8%	+0.2%	+2.9%	+0.3%
Death rate (per 100 000 population per year)					
2000	11.6	11.9	33.4	89.6	22.8
2010	7.4	11.2	30.9	83.9	22.8
2019	3.4	8.1	28.4	127	25.3
Rate of change in death rate (% per year)					
2000-2010	-4.4%	-0.6%	-0.8%	-0.7%	0.0%
2010-2019	-8.3%	-3.6%	-0.9%	+4.7%	+1.2%
2000-2019	-6.3%	-2.0%	-0.8%	+1.9%	+0.6%

Data source: Global Health Estimates 2021

Panel E3: Lower respiratory infections - Central Asia

	Age group				
	0-14	15-49	50-69	70+	All ages
Population size (millions)					
2000	100	115	21.7	5.4	242
2010	114	153	29.3	7.3	303
2019	131	177	39.7	8.8	357
Rate of change in population size (% per year)					
2000-2010	+1.3%	+2.9%	+3.0%	+3.1%	+2.3%
2010-2019	+1.6%	+1.6%	+3.4%	+2.1%	+1.8%
2000-2019	+1.4%	+2.3%	+3.2%	+2.6%	+2.1%
Deaths (thousands)					
2000	128	7.2	7.9	9.8	152
2010	98.8	7.0	8.5	12.8	127
2019	71.0	6.6	9.5	14.9	102
Rate of change in deaths (% per year)					
2000-2010	-2.5%	-0.2%	+0.8%	+2.7%	-1.8%
2010-2019	-3.6%	-0.7%	+1.2%	+1.7%	-2.4%
2000-2019	-3.0%	-0.4%	+1.0%	+2.2%	-2.1%
Death rate (per 100 000 population per year)					
2000	128	6.2	36.3	181	63.0
2010	87.0	4.6	29.1	174	41.9
2019	54.1	3.7	23.9	169	28.6
Rate of change in death rate (% per year)					
2000-2010	-3.8%	-3.0%	-2.2%	-0.4%	-4.0%
2010-2019	-5.1%	-2.3%	-2.2%	-0.4%	-4.2%
2000-2019	-4.4%	-2.7%	-2.2%	-0.4%	-4.1%

Data source: Global Health Estimates 2021

Panel E4: Lower respiratory infections - China

	Age group				
	0-14	15-49	50-69	70+	All ages
Population size (millions)					
2000	312	723	180	54.9	1,270
2010	250	767	257	77.6	1,350
2019	259	708	354	103	1,420
Rate of change in population size (% per year)					
2000-2010	-2.2%	+0.6%	+3.6%	+3.5%	+0.6%
2010-2019	+0.4%	-0.9%	+3.6%	+3.1%	+0.6%
2000-2019	-1.0%	-0.1%	+3.6%	+3.3%	+0.6%
Deaths (thousands)					
2000	113	14.8	27.1	134	289
2010	30.4	9.4	19.6	147	206
2019	13.0	7.1	22.0	160	202
Rate of change in deaths (% per year)					
2000-2010	-12%	-4.5%	-3.2%	+0.9%	-3.3%
2010-2019	-9.0%	-3.1%	+1.3%	+0.9%	-0.2%
2000-2019	-11%	-3.8%	-1.1%	+0.9%	-1.9%
Death rate (per 100 000 population per year)					
2000	36.2	2.0	15.0	244	22.7
2010	12.2	1.2	7.6	189	15.3
2019	5.0	1.0	6.2	156	14.2
Rate of change in death rate (% per year)					
2000-2010	-10%	-5.0%	-6.6%	-2.5%	-3.9%
2010-2019	-9.3%	-2.2%	-2.2%	-2.1%	-0.8%
2000-2019	-9.9%	-3.7%	-4.5%	-2.3%	-2.4%

Data source: Global Health Estimates 2021

Panel E5: Lower respiratory infections - India

	Age group				
	0-14	15-49	50-69	70+	All ages
Population size (millions)					
2000	376	543	111	27.6	1,060
2010	390	659	157	37.8	1,240
2019	373	757	209	50.5	1,390
Rate of change in population size (% per year)					
2000-2010	+0.4%	+1.9%	+3.6%	+3.2%	+1.6%
2010-2019	-0.5%	+1.6%	+3.2%	+3.3%	+1.2%
2000-2019	0.0%	+1.8%	+3.4%	+3.2%	+1.4%
Deaths (thousands)					
2000	435	24.7	54.7	82.8	597
2010	262	28.9	69.7	127	487
2019	113	27.7	102	186	428
Rate of change in deaths (% per year)					
2000-2010	-5.0%	+1.6%	+2.5%	+4.4%	-2.0%
2010-2019	-9.0%	-0.5%	+4.3%	+4.3%	-1.4%
2000-2019	-6.9%	+0.6%	+3.3%	+4.3%	-1.7%
Death rate (per 100 000 population per year)					
2000	116	4.5	49.4	300	56.5
2010	67.1	4.4	44.4	335	39.2
2019	30.2	3.7	48.9	368	30.8
Rate of change in death rate (% per year)					
2000-2010	-5.3%	-0.3%	-1.1%	+1.1%	-3.6%
2010-2019	-8.5%	-2.0%	+1.1%	+1.0%	-2.6%
2000-2019	-6.8%	-1.1%	0.0%	+1.1%	-3.1%

Data source: Global Health Estimates 2021

Panel E6: Lower respiratory infections - Latin America and Caribbean

	Age group				
	0-14	15-49	50-69	70+	All ages
Population size (millions)					
2000	166	272	59.4	17.7	515
2010	161	312	83.7	25.4	583
2019	155	337	110	34.6	636
Rate of change in population size (% per year)					
2000-2010	-0.3%	+1.4%	+3.5%	+3.7%	+1.2%
2010-2019	-0.5%	+0.8%	+3.1%	+3.5%	+1.0%
2000-2019	-0.4%	+1.1%	+3.3%	+3.6%	+1.1%
Deaths (thousands)					
2000	56.3	12.8	17.4	61.4	148
2010	27.8	14.7	26.9	109	179
2019	21.4	16.5	43.4	176	257
Rate of change in deaths (% per year)					
2000-2010	-6.8%	+1.4%	+4.5%	+5.9%	+1.9%
2010-2019	-2.9%	+1.3%	+5.4%	+5.4%	+4.1%
2000-2019	-5.0%	+1.3%	+4.9%	+5.7%	+2.9%
Death rate (per 100 000 population per year)					
2000	33.8	4.7	29.3	347	28.7
2010	17.2	4.7	32.1	430	30.6
2019	13.8	4.9	39.3	507	40.4
Rate of change in death rate (% per year)					
2000-2010	-6.5%	0.0%	+0.9%	+2.2%	+0.7%
2010-2019	-2.4%	+0.4%	+2.3%	+1.8%	+3.1%
2000-2019	-4.6%	+0.2%	+1.6%	+2.0%	+1.8%

Data source: Global Health Estimates 2021

Panel E7: Lower respiratory infections - Middle East and North Africa

	Age group				
	0-14	15-49	50-69	70+	All ages
Population size (millions)					
2000	135	200	36.9	9.6	381
2010	140	259	53.6	13.8	467
2019	163	296	76.1	18.3	553
Rate of change in population size (% per year)					
2000-2010	+0.4%	+2.6%	+3.8%	+3.7%	+2.0%
2010-2019	+1.7%	+1.5%	+4.0%	+3.2%	+1.9%
2000-2019	+1.0%	+2.1%	+3.9%	+3.5%	+2.0%
Deaths (thousands)					
2000	67.3	6.8	8.1	15.0	97.2
2010	42.2	7.6	10.9	24.1	84.8
2019	31.7	7.8	15.1	37.6	92.2
Rate of change in deaths (% per year)					
2000-2010	-4.6%	+1.1%	+3.0%	+4.9%	-1.4%
2010-2019	-3.1%	+0.3%	+3.7%	+5.0%	+0.9%
2000-2019	-3.9%	+0.7%	+3.3%	+5.0%	-0.3%
Death rate (per 100 000 population per year)					
2000	49.9	3.4	21.9	156	25.5
2010	30.1	2.9	20.3	175	18.2
2019	19.5	2.6	19.9	205	16.7
Rate of change in death rate (% per year)					
2000-2010	-4.9%	-1.6%	-0.8%	+1.2%	-3.3%
2010-2019	-4.7%	-1.2%	-0.2%	+1.8%	-0.9%
2000-2019	-4.8%	-1.4%	-0.5%	+1.5%	-2.2%

Data source: Global Health Estimates 2021

Panel E8: Lower respiratory infections - North Atlantic

	Age group				
	0-14	15-49	50-69	70+	All ages
Population size (millions)					
2000	72.0	209	93.2	47.7	422
2010	71.3	211	107	57.5	446
2019	72.1	202	122	67.8	464
Rate of change in population size (% per year)					
2000-2010	-0.1%	+0.1%	+1.4%	+1.9%	+0.6%
2010-2019	+0.1%	-0.4%	+1.5%	+1.9%	+0.4%
2000-2019	0.0%	-0.2%	+1.4%	+1.9%	+0.5%
Deaths (thousands)					
2000	0.7	2.9	12.4	150	166
2010	0.6	2.1	9.2	110	122
2019	0.5	2.1	11.3	137	151
Rate of change in deaths (% per year)					
2000-2010	-2.5%	-3.1%	-2.9%	-3.1%	-3.1%
2010-2019	-0.4%	0.0%	+2.4%	+2.5%	+2.4%
2000-2019	-1.5%	-1.6%	-0.5%	-0.5%	-0.5%
Death rate (per 100 000 population per year)					
2000	1.0	1.4	13.3	315	39.4
2010	0.8	1.0	8.6	191	27.3
2019	0.7	1.1	9.3	202	32.6
Rate of change in death rate (% per year)					
2000-2010	-2.4%	-3.2%	-4.2%	-4.9%	-3.6%
2010-2019	-0.6%	+0.5%	+0.9%	+0.6%	+2.0%
2000-2019	-1.5%	-1.5%	-1.9%	-2.3%	-1.0%

Data source: Global Health Estimates 2021

Panel E9: Lower respiratory infections - Sub-Saharan Africa

	Age group				
	0-14	15-49	50-69	70+	All ages
Population size (millions)					
2000	305	313	52.5	11.8	682
2010	393	418	69.0	15.6	895
2019	482	544	93.4	20.4	1,140
Rate of change in population size (% per year)					
2000-2010	+2.6%	+2.9%	+2.8%	+2.8%	+2.8%
2010-2019	+2.3%	+3.0%	+3.4%	+3.0%	+2.7%
2000-2019	+2.4%	+3.0%	+3.1%	+2.9%	+2.7%
Deaths (thousands)					
2000	674	42.4	67.5	85.8	869
2010	612	62.1	81.3	109	865
2019	517	69.1	95.7	138	819
Rate of change in deaths (% per year)					
2000-2010	-0.9%	+3.9%	+1.9%	+2.4%	-0.1%
2010-2019	-1.9%	+1.2%	+1.8%	+2.7%	-0.6%
2000-2019	-1.4%	+2.6%	+1.9%	+2.5%	-0.3%
Death rate (per 100 000 population per year)					
2000	221	13.6	128	727	128
2010	156	14.9	118	699	96.6
2019	107	12.7	102	679	71.9
Rate of change in death rate (% per year)					
2000-2010	-3.4%	+0.9%	-0.8%	-0.4%	-2.7%
2010-2019	-4.1%	-1.7%	-1.6%	-0.3%	-3.2%
2000-2019	-3.7%	-0.3%	-1.2%	-0.4%	-3.0%

Data source: Global Health Estimates 2021

Panel E10: Lower respiratory infections - United States

	Age group				
	0-14	15-49	50-69	70+	All ages
Population size (millions)					
2000	59.6	146	51.4	24.9	281
2010	61.3	151	71.3	27.5	311
2019	62.9	157	82.2	35.5	338
Rate of change in population size (% per year)					
2000-2010	+0.3%	+0.4%	+3.3%	+1.0%	+1.0%
2010-2019	+0.3%	+0.4%	+1.6%	+2.9%	+0.9%
2000-2019	+0.3%	+0.4%	+2.5%	+1.9%	+1.0%
Deaths (thousands)					
2000	1.1	2.6	6.4	59.6	69.7
2010	0.9	2.2	7.6	41.8	52.5
2019	0.8	2.6	10.7	37.5	51.6
Rate of change in deaths (% per year)					
2000-2010	-1.8%	-1.4%	+1.6%	-3.5%	-2.8%
2010-2019	-1.5%	+1.7%	+4.0%	-1.2%	-0.2%
2000-2019	-1.6%	+0.1%	+2.7%	-2.4%	-1.6%
Death rate (per 100 000 population per year)					
2000	1.9	1.8	12.5	239	24.8
2010	1.5	1.5	10.6	152	16.9
2019	1.3	1.6	13.0	106	15.3
Rate of change in death rate (% per year)					
2000-2010	-2.1%	-1.7%	-1.7%	-4.5%	-3.8%
2010-2019	-1.7%	+1.2%	+2.3%	-3.9%	-1.1%
2000-2019	-1.9%	-0.3%	+0.2%	-4.2%	-2.5%

Data source: Global Health Estimates 2021

Panel E11: Lower respiratory infections - Western Pacific and Southeast Asia

	Age group				
	0-14	15-49	50-69	70+	All ages
Population size (millions)					
2000	276	492	127	38.9	934
2010	275	549	163	56.0	1,040
2019	272	582	205	74.4	1,130
Rate of change in population size (% per year)					
2000-2010	0.0%	+1.1%	+2.5%	+3.7%	+1.1%
2010-2019	-0.1%	+0.6%	+2.6%	+3.2%	+0.9%
2000-2019	-0.1%	+0.9%	+2.5%	+3.5%	+1.0%
Deaths (thousands)					
2000	182	19.6	38.7	151	391
2010	88.8	20.3	48.4	232	390
2019	50.7	22.0	65.3	289	427
Rate of change in deaths (% per year)					
2000-2010	-6.9%	+0.4%	+2.3%	+4.4%	0.0%
2010-2019	-6.0%	+0.9%	+3.4%	+2.5%	+1.0%
2000-2019	-6.5%	+0.6%	+2.8%	+3.5%	+0.5%
Death rate (per 100 000 population per year)					
2000	65.9	4.0	30.4	389	41.9
2010	32.3	3.7	29.7	415	37.4
2019	18.7	3.8	31.9	389	37.7
Rate of change in death rate (% per year)					
2000-2010	-6.9%	-0.7%	-0.2%	+0.7%	-1.1%
2010-2019	-5.9%	+0.2%	+0.8%	-0.7%	+0.1%
2000-2019	-6.4%	-0.3%	+0.2%	0.0%	-0.6%

Data source: Global Health Estimates 2021

Panel F1: Malaria - World

	Age group				
	0-14	15-49	50-69	70+	All ages
Population size (millions)					
2000	1,870	3,210	808	269	6,160
2010	1,910	3,670	1,080	355	7,010
2019	2,030	3,930	1,380	451	7,800
Rate of change in population size (% per year)					
2000-2010	+0.2%	+1.3%	+2.9%	+2.8%	+1.3%
2010-2019	+0.7%	+0.8%	+2.9%	+2.7%	+1.2%
2000-2019	+0.4%	+1.1%	+2.9%	+2.7%	+1.2%
Deaths (thousands)					
2000	783	48.1	27.3	8.2	867
2010	615	54.5	33.1	10.7	713
2019	473	54.1	36.7	14.8	578
Rate of change in deaths (% per year)					
2000-2010	-2.4%	+1.3%	+1.9%	+2.7%	-1.9%
2010-2019	-2.9%	-0.1%	+1.2%	+3.7%	-2.3%
2000-2019	-2.6%	+0.6%	+1.6%	+3.2%	-2.1%
Death rate (per 100 000 population per year)					
2000	41.8	1.5	3.4	3.0	14.1
2010	32.2	1.5	3.1	3.0	10.2
2019	23.3	1.4	2.6	3.3	7.4
Rate of change in death rate (% per year)					
2000-2010	-2.6%	-0.1%	-0.9%	-0.1%	-3.2%
2010-2019	-3.5%	-0.9%	-1.6%	+1.0%	-3.5%
2000-2019	-3.0%	-0.4%	-1.3%	+0.4%	-3.3%

Data source: Global Health Estimates 2021

Panel F2: Malaria - Central and Eastern Europe

	Age group				
	0-14	15-49	50-69	70+	All ages
Population size (millions)					
2000	63.2	181	70.2	29.2	343
2010	50.2	169	78.6	34.2	332
2019	54.6	153	85.8	35.2	329
Rate of change in population size (% per year)					
2000-2010	-2.3%	-0.7%	+1.1%	+1.6%	-0.3%
2010-2019	+0.9%	-1.1%	+1.0%	+0.3%	-0.1%
2000-2019	-0.8%	-0.9%	+1.1%	+1.0%	-0.2%
Deaths (thousands)					
2000	<0.1	<0.1	<0.1	<0.1	<0.1
2010	<0.1	<0.1	<0.1	<0.1	<0.1
2019	<0.1	<0.1	<0.1	<0.1	<0.1
Rate of change in deaths (% per year)					
2000-2010	.	.	.	.	.
2010-2019	.	.	.	.	.
2000-2019	.	.	.	.	.
Death rate (per 100 000 population per year)					
2000	<0.1	<0.1	<0.1	<0.1	<0.1
2010	<0.1	<0.1	<0.1	<0.1	<0.1
2019	<0.1	<0.1	<0.1	<0.1	<0.1
Rate of change in death rate (% per year)					
2000-2010	.	.	.	.	.
2010-2019	.	.	.	.	.
2000-2019	.	.	.	.	.

Data source: Global Health Estimates 2021

Panel F3: Malaria - Central Asia

	Age group				
	0-14	15-49	50-69	70+	All ages
Population size (millions)					
2000	100	115	21.7	5.4	242
2010	114	153	29.3	7.3	303
2019	131	177	39.7	8.8	357
Rate of change in population size (% per year)					
2000-2010	+1.3%	+2.9%	+3.0%	+3.1%	+2.3%
2010-2019	+1.6%	+1.6%	+3.4%	+2.1%	+1.8%
2000-2019	+1.4%	+2.3%	+3.2%	+2.6%	+2.1%
Deaths (thousands)					
2000	0.9	0.6	0.4	<0.1	1.9
2010	2.0	0.5	0.2	<0.1	2.7
2019	0.4	0.2	<0.1	<0.1	0.7
Rate of change in deaths (% per year)					
2000-2010	+8.1%	-2.2%	-5.0%	.	+3.4%
2010-2019	-17%	-8.1%	-9.3%	.	-14%
2000-2019	-4.4%	-5.1%	-7.0%	.	-5.0%
Death rate (per 100 000 population per year)					
2000	0.9	0.5	1.6	1.4	0.8
2010	1.7	0.3	0.7	0.8	0.9
2019	0.3	0.1	0.2	0.3	0.2
Rate of change in death rate (% per year)					
2000-2010	+6.7%	-5.0%	-7.8%	-5.7%	+1.1%
2010-2019	-18%	-9.6%	-12%	-8.4%	-15%
2000-2019	-5.7%	-7.2%	-9.9%	-7.0%	-6.9%

Data source: Global Health Estimates 2021

Panel F4: Malaria - China

	Age group				
	0-14	15-49	50-69	70+	All ages
Population size (millions)					
2000	312	723	180	54.9	1,270
2010	250	767	257	77.6	1,350
2019	259	708	354	103	1,420
Rate of change in population size (% per year)					
2000-2010	-2.2%	+0.6%	+3.6%	+3.5%	+0.6%
2010-2019	+0.4%	-0.9%	+3.6%	+3.1%	+0.6%
2000-2019	-1.0%	-0.1%	+3.6%	+3.3%	+0.6%
Deaths (thousands)					
2000	<0.1	<0.1	<0.1	<0.1	<0.1
2010	<0.1	<0.1	<0.1	<0.1	<0.1
2019	<0.1	<0.1	<0.1	<0.1	<0.1
Rate of change in deaths (% per year)					
2000-2010	.	.	.	.	.
2010-2019	.	.	.	.	.
2000-2019	.	.	.	.	.
Death rate (per 100 000 population per year)					
2000	<0.1	<0.1	<0.1	<0.1	<0.1
2010	<0.1	<0.1	<0.1	<0.1	<0.1
2019	<0.1	<0.1	<0.1	<0.1	<0.1
Rate of change in death rate (% per year)					
2000-2010	.	.	.	.	.
2010-2019	.	.	.	.	.
2000-2019	.	.	.	.	.

Data source: Global Health Estimates 2021

Panel F5: Malaria - India

	Age group				
	0-14	15-49	50-69	70+	All ages
Population size (millions)					
2000	376	543	111	27.6	1,060
2010	390	659	157	37.8	1,240
2019	373	757	209	50.5	1,390
Rate of change in population size (% per year)					
2000-2010	+0.4%	+1.9%	+3.6%	+3.2%	+1.6%
2010-2019	-0.5%	+1.6%	+3.2%	+3.3%	+1.2%
2000-2019	0.0%	+1.8%	+3.4%	+3.2%	+1.4%
Deaths (thousands)					
2000	20.6	5.3	3.0	0.8	29.7
2010	17.8	7.1	4.5	1.2	30.7
2019	4.1	1.8	1.4	0.4	7.7
Rate of change in deaths (% per year)					
2000-2010	-1.4%	+3.1%	+4.2%	+4.4%	+0.3%
2010-2019	-15%	-14%	-12%	-11%	-14%
2000-2019	-8.1%	-5.6%	-3.8%	-3.3%	-6.8%
Death rate (per 100 000 population per year)					
2000	5.5	1.0	2.7	2.8	2.8
2010	4.6	1.1	2.8	3.2	2.5
2019	1.1	0.2	0.7	0.8	0.6
Rate of change in death rate (% per year)					
2000-2010	-1.8%	+1.1%	+0.6%	+1.2%	-1.3%
2010-2019	-15%	-16%	-15%	-14%	-15%
2000-2019	-8.1%	-7.2%	-6.9%	-6.3%	-8.2%

Data source: Global Health Estimates 2021

Panel F6: Malaria - Latin America and Caribbean

	Age group				
	0-14	15-49	50-69	70+	All ages
Population size (millions)					
2000	166	272	59.4	17.7	515
2010	161	312	83.7	25.4	583
2019	155	337	110	34.6	636
Rate of change in population size (% per year)					
2000-2010	-0.3%	+1.4%	+3.5%	+3.7%	+1.2%
2010-2019	-0.5%	+0.8%	+3.1%	+3.5%	+1.0%
2000-2019	-0.4%	+1.1%	+3.3%	+3.6%	+1.1%
Deaths (thousands)					
2000	0.3	0.4	0.1	<0.1	1.0
2010	0.1	0.2	<0.1	<0.1	0.5
2019	0.1	0.2	<0.1	<0.1	0.5
Rate of change in deaths (% per year)					
2000-2010	-7.8%	-5.7%	-5.6%	.	-6.4%
2010-2019	-1.5%	-0.3%	.	.	-0.5%
2000-2019	-4.9%	-3.1%	-2.6%	.	-3.6%
Death rate (per 100 000 population per year)					
2000	0.2	0.2	0.2	0.4	0.2
2010	<0.1	<0.1	<0.1	0.1	<0.1
2019	<0.1	<0.1	<0.1	<0.1	<0.1
Rate of change in death rate (% per year)					
2000-2010	-7.5%	-7.0%	-8.8%	-9.8%	-7.5%
2010-2019	.	.	.	-4.9%	.
2000-2019	-4.5%	-4.2%	-5.8%	-7.5%	-4.7%

Data source: Global Health Estimates 2021

Panel F7: Malaria - Middle East and North Africa

	Age group				
	0-14	15-49	50-69	70+	All ages
Population size (millions)					
2000	135	200	36.9	9.6	381
2010	140	259	53.6	13.8	467
2019	163	296	76.1	18.3	553
Rate of change in population size (% per year)					
2000-2010	+0.4%	+2.6%	+3.8%	+3.7%	+2.0%
2010-2019	+1.7%	+1.5%	+4.0%	+3.2%	+1.9%
2000-2019	+1.0%	+2.1%	+3.9%	+3.5%	+2.0%
Deaths (thousands)					
2000	1.1	1.3	0.3	<0.1	2.8
2010	1.2	1.5	0.3	0.1	3.1
2019	0.8	1.2	0.3	0.1	2.4
Rate of change in deaths (% per year)					
2000-2010	+0.1%	+1.6%	+0.8%	+2.4%	+0.9%
2010-2019	-3.9%	-2.8%	-0.9%	+1.5%	-2.8%
2000-2019	-1.8%	-0.6%	0.0%	+2.0%	-0.9%
Death rate (per 100 000 population per year)					
2000	0.8	0.7	0.7	0.9	0.7
2010	0.8	0.6	0.5	0.8	0.7
2019	0.5	0.4	0.4	0.7	0.4
Rate of change in death rate (% per year)					
2000-2010	-0.2%	-1.1%	-2.9%	-1.2%	-1.1%
2010-2019	-5.5%	-4.3%	-4.7%	-1.6%	-4.7%
2000-2019	-2.8%	-2.6%	-3.8%	-1.4%	-2.8%

Data source: Global Health Estimates 2021

Panel F8: Malaria - North Atlantic

	Age group				
	0-14	15-49	50-69	70+	All ages
Population size (millions)					
2000	72.0	209	93.2	47.7	422
2010	71.3	211	107	57.5	446
2019	72.1	202	122	67.8	464
Rate of change in population size (% per year)					
2000-2010	-0.1%	+0.1%	+1.4%	+1.9%	+0.6%
2010-2019	+0.1%	-0.4%	+1.5%	+1.9%	+0.4%
2000-2019	0.0%	-0.2%	+1.4%	+1.9%	+0.5%
Deaths (thousands)					
2000	<0.1	<0.1	<0.1	<0.1	<0.1
2010	<0.1	<0.1	<0.1	<0.1	<0.1
2019	<0.1	<0.1	<0.1	<0.1	<0.1
Rate of change in deaths (% per year)					
2000-2010	.	.	.	.	.
2010-2019	.	.	.	.	.
2000-2019	.	.	.	.	.
Death rate (per 100 000 population per year)					
2000	<0.1	<0.1	<0.1	<0.1	<0.1
2010	<0.1	<0.1	<0.1	<0.1	<0.1
2019	<0.1	<0.1	<0.1	<0.1	<0.1
Rate of change in death rate (% per year)					
2000-2010	.	.	.	.	.
2010-2019	.	.	.	.	.
2000-2019	.	.	.	.	.

Data source: Global Health Estimates 2021

Panel F9: Malaria - Sub-Saharan Africa

	Age group				
	0-14	15-49	50-69	70+	All ages
Population size (millions)					
2000	305	313	52.5	11.8	682
2010	393	418	69.0	15.6	895
2019	482	544	93.4	20.4	1,140
Rate of change in population size (% per year)					
2000-2010	+2.6%	+2.9%	+2.8%	+2.8%	+2.8%
2010-2019	+2.3%	+3.0%	+3.4%	+3.0%	+2.7%
2000-2019	+2.4%	+3.0%	+3.1%	+2.9%	+2.7%
Deaths (thousands)					
2000	754	36.1	21.9	6.9	819
2010	590	40.4	26.2	8.9	665
2019	466	48.9	34.1	14.1	563
Rate of change in deaths (% per year)					
2000-2010	-2.4%	+1.1%	+1.8%	+2.6%	-2.1%
2010-2019	-2.6%	+2.1%	+3.0%	+5.2%	-1.8%
2000-2019	-2.5%	+1.6%	+2.4%	+3.8%	-2.0%
Death rate (per 100 000 population per year)					
2000	248	11.5	41.7	58.2	120
2010	150	9.7	38.0	57.2	74.3
2019	96.6	9.0	36.5	69.0	49.4
Rate of change in death rate (% per year)					
2000-2010	-4.9%	-1.7%	-0.9%	-0.2%	-4.7%
2010-2019	-4.8%	-0.8%	-0.4%	+2.1%	-4.4%
2000-2019	-4.8%	-1.3%	-0.7%	+0.9%	-4.6%

Data source: Global Health Estimates 2021

Panel F10: Malaria - United States

	Age group				
	0-14	15-49	50-69	70+	All ages
Population size (millions)					
2000	59.6	146	51.4	24.9	281
2010	61.3	151	71.3	27.5	311
2019	62.9	157	82.2	35.5	338
Rate of change in population size (% per year)					
2000-2010	+0.3%	+0.4%	+3.3%	+1.0%	+1.0%
2010-2019	+0.3%	+0.4%	+1.6%	+2.9%	+0.9%
2000-2019	+0.3%	+0.4%	+2.5%	+1.9%	+1.0%
Deaths (thousands)					
2000	<0.1	<0.1	<0.1	<0.1	<0.1
2010	<0.1	<0.1	<0.1	<0.1	<0.1
2019	<0.1	<0.1	<0.1	<0.1	<0.1
Rate of change in deaths (% per year)					
2000-2010	.	.	.	.	.
2010-2019	.	.	.	.	.
2000-2019	.	.	.	.	.
Death rate (per 100 000 population per year)					
2000	<0.1	<0.1	<0.1	<0.1	<0.1
2010	<0.1	<0.1	<0.1	<0.1	<0.1
2019	<0.1	<0.1	<0.1	<0.1	<0.1
Rate of change in death rate (% per year)					
2000-2010	.	.	.	.	.
2010-2019	.	.	.	.	.
2000-2019	.	.	.	.	.

Data source: Global Health Estimates 2021

Panel F11: Malaria - Western Pacific and Southeast Asia

	Age group				
	0-14	15-49	50-69	70+	All ages
Population size (millions)					
2000	276	492	127	38.9	934
2010	275	549	163	56.0	1,040
2019	272	582	205	74.4	1,130
Rate of change in population size (% per year)					
2000-2010	0.0%	+1.1%	+2.5%	+3.7%	+1.1%
2010-2019	-0.1%	+0.6%	+2.6%	+3.2%	+0.9%
2000-2019	-0.1%	+0.9%	+2.5%	+3.5%	+1.0%
Deaths (thousands)					
2000	5.8	4.3	1.6	0.3	12.1
2010	4.1	4.7	1.8	0.3	10.9
2019	1.5	1.7	0.8	0.2	4.1
Rate of change in deaths (% per year)					
2000-2010	-3.5%	+0.8%	+1.1%	+0.4%	-1.0%
2010-2019	-10%	-11%	-9.3%	-7.6%	-10%
2000-2019	-6.8%	-4.8%	-4.0%	-3.4%	-5.5%
Death rate (per 100 000 population per year)					
2000	2.1	0.9	1.3	0.9	1.3
2010	1.5	0.9	1.1	0.6	1.0
2019	0.6	0.3	0.4	0.2	0.4
Rate of change in death rate (% per year)					
2000-2010	-3.5%	-0.3%	-1.4%	-3.1%	-2.1%
2010-2019	-10%	-11%	-12%	-10%	-11%
2000-2019	-6.7%	-5.6%	-6.4%	-6.7%	-6.4%

Data source: Global Health Estimates 2021

Panel G1: Maternal conditions - World

	Age group				
	0-14	15-49	50-69	70+	All ages
Population size (millions)					
2000	1,870	3,210	808	269	6,160
2010	1,910	3,670	1,080	355	7,010
2019	2,030	3,930	1,380	451	7,800
Rate of change in population size (% per year)					
2000-2010	+0.2%	+1.3%	+2.9%	+2.8%	+1.3%
2010-2019	+0.7%	+0.8%	+2.9%	+2.7%	+1.2%
2000-2019	+0.4%	+1.1%	+2.9%	+2.7%	+1.2%
Deaths (thousands)					
2000	<0.1	410	<0.1	<0.1	410
2010	<0.1	310	<0.1	<0.1	310
2019	<0.1	240	<0.1	<0.1	240
Rate of change in deaths (% per year)					
2000-2010	.	-2.8%	.	.	-2.8%
2010-2019	.	-2.8%	.	.	-2.8%
2000-2019	.	-2.8%	.	.	-2.8%
Death rate (per 100 000 population per year)					
2000	<0.1	12.8	<0.1	<0.1	6.7
2010	<0.1	8.4	<0.1	<0.1	4.4
2019	<0.1	6.1	<0.1	<0.1	3.1
Rate of change in death rate (% per year)					
2000-2010	.	-4.1%	.	.	-4.0%
2010-2019	.	-3.5%	.	.	-3.9%
2000-2019	.	-3.8%	.	.	-4.0%

Data source: Global Health Estimates 2021

Panel G2: Maternal conditions - Central and Eastern Europe

	Age group				
	0-14	15-49	50-69	70+	All ages
Population size (millions)					
2000	63.2	181	70.2	29.2	343
2010	50.2	169	78.6	34.2	332
2019	54.6	153	85.8	35.2	329
Rate of change in population size (% per year)					
2000-2010	-2.3%	-0.7%	+1.1%	+1.6%	-0.3%
2010-2019	+0.9%	-1.1%	+1.0%	+0.3%	-0.1%
2000-2019	-0.8%	-0.9%	+1.1%	+1.0%	-0.2%
Deaths (thousands)					
2000	<0.1	1.2	<0.1	<0.1	1.2
2010	<0.1	0.7	<0.1	<0.1	0.7
2019	<0.1	0.3	<0.1	<0.1	0.3
Rate of change in deaths (% per year)					
2000-2010	.	-4.6%	.	.	-4.6%
2010-2019	.	-7.7%	.	.	-7.7%
2000-2019	.	-6.1%	.	.	-6.1%
Death rate (per 100 000 population per year)					
2000	<0.1	0.6	<0.1	<0.1	0.3
2010	<0.1	0.4	<0.1	<0.1	0.2
2019	<0.1	0.2	<0.1	<0.1	0.1
Rate of change in death rate (% per year)					
2000-2010	.	-4.0%	.	.	-4.3%
2010-2019	.	-6.8%	.	.	-7.6%
2000-2019	.	-5.3%	.	.	-5.9%

Data source: Global Health Estimates 2021

Panel G3: Maternal conditions - Central Asia

	Age group				
	0-14	15-49	50-69	70+	All ages
Population size (millions)					
2000	100	115	21.7	5.4	242
2010	114	153	29.3	7.3	303
2019	131	177	39.7	8.8	357
Rate of change in population size (% per year)					
2000-2010	+1.3%	+2.9%	+3.0%	+3.1%	+2.3%
2010-2019	+1.6%	+1.6%	+3.4%	+2.1%	+1.8%
2000-2019	+1.4%	+2.3%	+3.2%	+2.6%	+2.1%
Deaths (thousands)					
2000	<0.1	34.3	<0.1	<0.1	34.3
2010	<0.1	32.5	<0.1	<0.1	32.5
2019	<0.1	27.0	<0.1	<0.1	27.0
Rate of change in deaths (% per year)					
2000-2010	.	-0.5%	.	.	-0.5%
2010-2019	.	-2.0%	.	.	-2.0%
2000-2019	.	-1.2%	.	.	-1.2%
Death rate (per 100 000 population per year)					
2000	<0.1	29.9	<0.1	<0.1	14.2
2010	<0.1	21.2	<0.1	<0.1	10.7
2019	<0.1	15.2	<0.1	<0.1	7.6
Rate of change in death rate (% per year)					
2000-2010	.	-3.4%	.	.	-2.8%
2010-2019	.	-3.6%	.	.	-3.8%
2000-2019	.	-3.5%	.	.	-3.3%

Data source: Global Health Estimates 2021

Panel G4: Maternal conditions - China

	Age group				
	0-14	15-49	50-69	70+	All ages
Population size (millions)					
2000	312	723	180	54.9	1,270
2010	250	767	257	77.6	1,350
2019	259	708	354	103	1,420
Rate of change in population size (% per year)					
2000-2010	-2.2%	+0.6%	+3.6%	+3.5%	+0.6%
2010-2019	+0.4%	-0.9%	+3.6%	+3.1%	+0.6%
2000-2019	-1.0%	-0.1%	+3.6%	+3.3%	+0.6%
Deaths (thousands)					
2000	<0.1	9.7	<0.1	<0.1	9.7
2010	<0.1	5.5	<0.1	<0.1	5.5
2019	<0.1	2.6	<0.1	<0.1	2.6
Rate of change in deaths (% per year)					
2000-2010	.	-5.5%	.	.	-5.5%
2010-2019	.	-7.8%	.	.	-7.8%
2000-2019	.	-6.6%	.	.	-6.6%
Death rate (per 100 000 population per year)					
2000	<0.1	1.3	<0.1	<0.1	0.8
2010	<0.1	0.7	<0.1	<0.1	0.4
2019	<0.1	0.4	<0.1	<0.1	0.2
Rate of change in death rate (% per year)					
2000-2010	.	-6.1%	.	.	-6.1%
2010-2019	.	-7.0%	.	.	-8.4%
2000-2019	.	-6.5%	.	.	-7.2%

Data source: Global Health Estimates 2021

Panel G5: Maternal conditions - India

	Age group				
	0-14	15-49	50-69	70+	All ages
Population size (millions)					
2000	376	543	111	27.6	1,060
2010	390	659	157	37.8	1,240
2019	373	757	209	50.5	1,390
Rate of change in population size (% per year)					
2000-2010	+0.4%	+1.9%	+3.6%	+3.2%	+1.6%
2010-2019	-0.5%	+1.6%	+3.2%	+3.3%	+1.2%
2000-2019	0.0%	+1.8%	+3.4%	+3.2%	+1.4%
Deaths (thousands)					
2000	<0.1	111	<0.1	<0.1	111
2010	<0.1	49.2	<0.1	<0.1	49.2
2019	<0.1	25.7	<0.1	<0.1	25.7
Rate of change in deaths (% per year)					
2000-2010	.	-7.8%	.	.	-7.8%
2010-2019	.	-7.0%	.	.	-7.0%
2000-2019	.	-7.4%	.	.	-7.4%
Death rate (per 100 000 population per year)					
2000	<0.1	20.5	<0.1	<0.1	10.5
2010	<0.1	7.5	<0.1	<0.1	4.0
2019	<0.1	3.4	<0.1	<0.1	1.8
Rate of change in death rate (% per year)					
2000-2010	.	-9.6%	.	.	-9.3%
2010-2019	.	-8.4%	.	.	-8.1%
2000-2019	.	-9.0%	.	.	-8.7%

Data source: Global Health Estimates 2021

Panel G6: Maternal conditions - Latin America and Caribbean

	Age group				
	0-14	15-49	50-69	70+	All ages
Population size (millions)					
2000	166	272	59.4	17.7	515
2010	161	312	83.7	25.4	583
2019	155	337	110	34.6	636
Rate of change in population size (% per year)					
2000-2010	-0.3%	+1.4%	+3.5%	+3.7%	+1.2%
2010-2019	-0.5%	+0.8%	+3.1%	+3.5%	+1.0%
2000-2019	-0.4%	+1.1%	+3.3%	+3.6%	+1.1%
Deaths (thousands)					
2000	<0.1	9.4	<0.1	<0.1	9.4
2010	<0.1	8.4	<0.1	<0.1	8.4
2019	<0.1	8.1	<0.1	<0.1	8.1
Rate of change in deaths (% per year)					
2000-2010	.	-1.2%	.	.	-1.2%
2010-2019	.	-0.3%	.	.	-0.3%
2000-2019	.	-0.8%	.	.	-0.8%
Death rate (per 100 000 population per year)					
2000	<0.1	3.5	<0.1	<0.1	1.8
2010	<0.1	2.7	<0.1	<0.1	1.4
2019	<0.1	2.4	<0.1	<0.1	1.3
Rate of change in death rate (% per year)					
2000-2010	.	-2.5%	.	.	-2.4%
2010-2019	.	-1.2%	.	.	-1.3%
2000-2019	.	-1.9%	.	.	-1.9%

Data source: Global Health Estimates 2021

Panel G7: Maternal conditions - Middle East and North Africa

	Age group				
	0-14	15-49	50-69	70+	All ages
Population size (millions)					
2000	135	200	36.9	9.6	381
2010	140	259	53.6	13.8	467
2019	163	296	76.1	18.3	553
Rate of change in population size (% per year)					
2000-2010	+0.4%	+2.6%	+3.8%	+3.7%	+2.0%
2010-2019	+1.7%	+1.5%	+4.0%	+3.2%	+1.9%
2000-2019	+1.0%	+2.1%	+3.9%	+3.5%	+2.0%
Deaths (thousands)					
2000	<0.1	7.6	<0.1	<0.1	7.6
2010	<0.1	6.2	<0.1	<0.1	6.2
2019	<0.1	5.4	<0.1	<0.1	5.4
Rate of change in deaths (% per year)					
2000-2010	.	-1.9%	.	.	-1.9%
2010-2019	.	-1.5%	.	.	-1.5%
2000-2019	.	-1.7%	.	.	-1.7%
Death rate (per 100 000 population per year)					
2000	<0.1	3.8	<0.1	<0.1	2.0
2010	<0.1	2.4	<0.1	<0.1	1.3
2019	<0.1	1.8	<0.1	<0.1	1.0
Rate of change in death rate (% per year)					
2000-2010	.	-4.5%	.	.	-3.9%
2010-2019	.	-3.0%	.	.	-3.4%
2000-2019	.	-3.7%	.	.	-3.6%

Data source: Global Health Estimates 2021

Panel G8: Maternal conditions - North Atlantic

	Age group				
	0-14	15-49	50-69	70+	All ages
Population size (millions)					
2000	72.0	209	93.2	47.7	422
2010	71.3	211	107	57.5	446
2019	72.1	202	122	67.8	464
Rate of change in population size (% per year)					
2000-2010	-0.1%	+0.1%	+1.4%	+1.9%	+0.6%
2010-2019	+0.1%	-0.4%	+1.5%	+1.9%	+0.4%
2000-2019	0.0%	-0.2%	+1.4%	+1.9%	+0.5%
Deaths (thousands)					
2000	<0.1	0.4	<0.1	<0.1	0.4
2010	<0.1	0.4	<0.1	<0.1	0.4
2019	<0.1	0.3	<0.1	<0.1	0.3
Rate of change in deaths (% per year)					
2000-2010	.	-0.8%	.	.	-0.8%
2010-2019	.	-2.5%	.	.	-2.5%
2000-2019	.	-1.6%	.	.	-1.6%
Death rate (per 100 000 population per year)					
2000	<0.1	0.2	<0.1	<0.1	<0.1
2010	<0.1	0.2	<0.1	<0.1	<0.1
2019	<0.1	0.1	<0.1	<0.1	<0.1
Rate of change in death rate (% per year)					
2000-2010	.	-0.9%	.	.	.
2010-2019	.	-2.0%	.	.	.
2000-2019	.	-1.5%	.	.	.

Data source: Global Health Estimates 2021

Panel G9: Maternal conditions - Sub-Saharan Africa

	Age group				
	0-14	15-49	50-69	70+	All ages
Population size (millions)					
2000	305	313	52.5	11.8	682
2010	393	418	69.0	15.6	895
2019	482	544	93.4	20.4	1,140
Rate of change in population size (% per year)					
2000-2010	+2.6%	+2.9%	+2.8%	+2.8%	+2.8%
2010-2019	+2.3%	+3.0%	+3.4%	+3.0%	+2.7%
2000-2019	+2.4%	+3.0%	+3.1%	+2.9%	+2.7%
Deaths (thousands)					
2000	<0.1	194	<0.1	<0.1	194
2010	<0.1	179	<0.1	<0.1	179
2019	<0.1	151	<0.1	<0.1	151
Rate of change in deaths (% per year)					
2000-2010	.	-0.8%	.	.	-0.8%
2010-2019	.	-1.8%	.	.	-1.8%
2000-2019	.	-1.3%	.	.	-1.3%
Death rate (per 100 000 population per year)					
2000	<0.1	62.0	<0.1	<0.1	28.4
2010	<0.1	42.8	<0.1	<0.1	19.9
2019	<0.1	27.8	<0.1	<0.1	13.3
Rate of change in death rate (% per year)					
2000-2010	.	-3.6%	.	.	-3.5%
2010-2019	.	-4.7%	.	.	-4.4%
2000-2019	.	-4.1%	.	.	-3.9%

Data source: Global Health Estimates 2021

Panel G10: Maternal conditions - United States

	Age group				
	0-14	15-49	50-69	70+	All ages
Population size (millions)					
2000	59.6	146	51.4	24.9	281
2010	61.3	151	71.3	27.5	311
2019	62.9	157	82.2	35.5	338
Rate of change in population size (% per year)					
2000-2010	+0.3%	+0.4%	+3.3%	+1.0%	+1.0%
2010-2019	+0.3%	+0.4%	+1.6%	+2.9%	+0.9%
2000-2019	+0.3%	+0.4%	+2.5%	+1.9%	+1.0%
Deaths (thousands)					
2000	<0.1	0.5	<0.1	<0.1	0.5
2010	<0.1	0.6	<0.1	<0.1	0.6
2019	<0.1	0.7	<0.1	<0.1	0.7
Rate of change in deaths (% per year)					
2000-2010	.	+1.7%	.	.	+1.7%
2010-2019	.	+3.1%	.	.	+3.1%
2000-2019	.	+2.4%	.	.	+2.4%
Death rate (per 100 000 population per year)					
2000	<0.1	0.3	<0.1	<0.1	0.2
2010	<0.1	0.4	<0.1	<0.1	0.2
2019	<0.1	0.5	<0.1	<0.1	0.2
Rate of change in death rate (% per year)					
2000-2010	.	+1.4%	.	.	+0.7%
2010-2019	.	+2.6%	.	.	+2.1%
2000-2019	.	+2.0%	.	.	+1.4%

Data source: Global Health Estimates 2021

Panel G11: Maternal conditions - Western Pacific and Southeast Asia

	Age group				
	0-14	15-49	50-69	70+	All ages
Population size (millions)					
2000	276	492	127	38.9	934
2010	275	549	163	56.0	1,040
2019	272	582	205	74.4	1,130
Rate of change in population size (% per year)					
2000-2010	0.0%	+1.1%	+2.5%	+3.7%	+1.1%
2010-2019	-0.1%	+0.6%	+2.6%	+3.2%	+0.9%
2000-2019	-0.1%	+0.9%	+2.5%	+3.5%	+1.0%
Deaths (thousands)					
2000	<0.1	42.2	<0.1	<0.1	42.2
2010	<0.1	27.6	<0.1	<0.1	27.6
2019	<0.1	18.4	<0.1	<0.1	18.4
Rate of change in deaths (% per year)					
2000-2010	.	-4.2%	.	.	-4.2%
2010-2019	.	-4.4%	.	.	-4.4%
2000-2019	.	-4.3%	.	.	-4.3%
Death rate (per 100 000 population per year)					
2000	<0.1	8.6	<0.1	<0.1	4.5
2010	<0.1	5.0	<0.1	<0.1	2.6
2019	<0.1	3.2	<0.1	<0.1	1.6
Rate of change in death rate (% per year)					
2000-2010	.	-5.2%	.	.	-5.2%
2010-2019	.	-5.0%	.	.	-5.3%
2000-2019	.	-5.1%	.	.	-5.2%

Data source: Global Health Estimates 2021

Panel H1: Neonatal conditions - World

	Age group				
	0-14	15-49	50-69	70+	All ages
Population size (millions)					
2000	1,870	3,210	808	269	6,160
2010	1,910	3,670	1,080	355	7,010
2019	2,030	3,930	1,380	451	7,800
Rate of change in population size (% per year)					
2000-2010	+0.2%	+1.3%	+2.9%	+2.8%	+1.3%
2010-2019	+0.7%	+0.8%	+2.9%	+2.7%	+1.2%
2000-2019	+0.4%	+1.1%	+2.9%	+2.7%	+1.2%
Deaths (thousands)					
2000	3,300	<0.1	<0.1	<0.1	3,300
2010	2,610	0.1	<0.1	<0.1	2,610
2019	2,060	0.2	0.1	<0.1	2,060
Rate of change in deaths (% per year)					
2000-2010	-2.3%	+5.7%	.	.	-2.3%
2010-2019	-2.6%	+6.0%	+17%	.	-2.6%
2000-2019	-2.4%	+5.9%	+15%	.	-2.4%
Death rate (per 100 000 population per year)					
2000	176	<0.1	<0.1	<0.1	53.5
2010	137	<0.1	<0.1	<0.1	37.3
2019	102	<0.1	<0.1	<0.1	26.4
Rate of change in death rate (% per year)					
2000-2010	-2.5%	.	.	.	-3.6%
2010-2019	-3.2%	.	.	.	-3.8%
2000-2019	-2.9%	.	.	.	-3.6%

Data source: Global Health Estimates 2021

Panel H2: Neonatal conditions - Central and Eastern Europe

	Age group				
	0-14	15-49	50-69	70+	All ages
Population size (millions)					
2000	63.2	181	70.2	29.2	343
2010	50.2	169	78.6	34.2	332
2019	54.6	153	85.8	35.2	329
Rate of change in population size (% per year)					
2000-2010	-2.3%	-0.7%	+1.1%	+1.6%	-0.3%
2010-2019	+0.9%	-1.1%	+1.0%	+0.3%	-0.1%
2000-2019	-0.8%	-0.9%	+1.1%	+1.0%	-0.2%
Deaths (thousands)					
2000	20.1	<0.1	<0.1	<0.1	20.1
2010	12.9	<0.1	<0.1	<0.1	13.0
2019	7.3	<0.1	<0.1	<0.1	7.3
Rate of change in deaths (% per year)					
2000-2010	-4.3%	.	.	.	-4.3%
2010-2019	-6.1%	.	.	.	-6.2%
2000-2019	-5.2%	.	.	.	-5.2%
Death rate (per 100 000 population per year)					
2000	31.8	<0.1	<0.1	<0.1	5.9
2010	25.8	<0.1	<0.1	0.1	3.9
2019	13.4	<0.1	<0.1	<0.1	2.2
Rate of change in death rate (% per year)					
2000-2010	-2.1%	.	.	-	-3.9%
2010-2019	-7.0%	.	.	-100%	-6.1%
2000-2019	-4.4%	.	.	.	-5.0%

Data source: Global Health Estimates 2021

Panel H3: Neonatal conditions - Central Asia

	Age group				
	0-14	15-49	50-69	70+	All ages
Population size (millions)					
2000	100	115	21.7	5.4	242
2010	114	153	29.3	7.3	303
2019	131	177	39.7	8.8	357
Rate of change in population size (% per year)					
2000-2010	+1.3%	+2.9%	+3.0%	+3.1%	+2.3%
2010-2019	+1.6%	+1.6%	+3.4%	+2.1%	+1.8%
2000-2019	+1.4%	+2.3%	+3.2%	+2.6%	+2.1%
Deaths (thousands)					
2000	347	<0.1	<0.1	<0.1	347
2010	347	<0.1	<0.1	<0.1	347
2019	292	<0.1	<0.1	<0.1	292
Rate of change in deaths (% per year)					
2000-2010	0.0%	.	.	.	0.0%
2010-2019	-1.9%	.	.	.	-1.9%
2000-2019	-0.9%	.	.	.	-0.9%
Death rate (per 100 000 population per year)					
2000	347	<0.1	<0.1	<0.1	143
2010	306	<0.1	<0.1	<0.1	114
2019	222	<0.1	<0.1	<0.1	81.7
Rate of change in death rate (% per year)					
2000-2010	-1.3%	.	.	.	-2.2%
2010-2019	-3.5%	.	.	.	-3.7%
2000-2019	-2.3%	.	.	.	-2.9%

Data source: Global Health Estimates 2021

Panel H4: Neonatal conditions - China

	Age group				
	0-14	15-49	50-69	70+	All ages
Population size (millions)					
2000	312	723	180	54.9	1,270
2010	250	767	257	77.6	1,350
2019	259	708	354	103	1,420
Rate of change in population size (% per year)					
2000-2010	-2.2%	+0.6%	+3.6%	+3.5%	+0.6%
2010-2019	+0.4%	-0.9%	+3.6%	+3.1%	+0.6%
2000-2019	-1.0%	-0.1%	+3.6%	+3.3%	+0.6%
Deaths (thousands)					
2000	239	<0.1	<0.1	<0.1	239
2010	105	<0.1	<0.1	<0.1	105
2019	40.5	<0.1	<0.1	<0.1	40.5
Rate of change in deaths (% per year)					
2000-2010	-7.9%	.	.	.	-7.9%
2010-2019	-10%	.	.	.	-10%
2000-2019	-8.9%	.	.	.	-8.9%
Death rate (per 100 000 population per year)					
2000	76.6	<0.1	<0.1	<0.1	18.8
2010	41.9	<0.1	<0.1	<0.1	7.8
2019	15.6	<0.1	<0.1	<0.1	2.8
Rate of change in death rate (% per year)					
2000-2010	-5.9%	.	.	.	-8.5%
2010-2019	-10%	.	.	.	-11%
2000-2019	-8.0%	.	.	.	-9.5%

Data source: Global Health Estimates 2021

Panel H5: Neonatal conditions - India

	Age group				
	0-14	15-49	50-69	70+	All ages
Population size (millions)					
2000	376	543	111	27.6	1,060
2010	390	659	157	37.8	1,240
2019	373	757	209	50.5	1,390
Rate of change in population size (% per year)					
2000-2010	+0.4%	+1.9%	+3.6%	+3.2%	+1.6%
2010-2019	-0.5%	+1.6%	+3.2%	+3.3%	+1.2%
2000-2019	0.0%	+1.8%	+3.4%	+3.2%	+1.4%
Deaths (thousands)					
2000	1,060	<0.1	<0.1	<0.1	1,060
2010	719	<0.1	<0.1	<0.1	719
2019	432	<0.1	<0.1	<0.1	432
Rate of change in deaths (% per year)					
2000-2010	-3.8%	.	.	.	-3.8%
2010-2019	-5.5%	.	.	.	-5.5%
2000-2019	-4.6%	.	.	.	-4.6%
Death rate (per 100 000 population per year)					
2000	282	<0.1	<0.1	<0.1	100
2010	184	<0.1	<0.1	<0.1	57.8
2019	116	<0.1	<0.1	<0.1	31.1
Rate of change in death rate (% per year)					
2000-2010	-4.2%	.	.	.	-5.4%
2010-2019	-5.0%	.	.	.	-6.7%
2000-2019	-4.6%	.	.	.	-6.0%

Data source: Global Health Estimates 2021

Panel H6: Neonatal conditions - Latin America and Caribbean

	Age group				
	0-14	15-49	50-69	70+	All ages
Population size (millions)					
2000	166	272	59.4	17.7	515
2010	161	312	83.7	25.4	583
2019	155	337	110	34.6	636
Rate of change in population size (% per year)					
2000-2010	-0.3%	+1.4%	+3.5%	+3.7%	+1.2%
2010-2019	-0.5%	+0.8%	+3.1%	+3.5%	+1.0%
2000-2019	-0.4%	+1.1%	+3.3%	+3.6%	+1.1%
Deaths (thousands)					
2000	153	<0.1	<0.1	<0.1	153
2010	93.7	<0.1	<0.1	<0.1	93.7
2019	74.2	<0.1	<0.1	<0.1	74.2
Rate of change in deaths (% per year)					
2000-2010	-4.8%	.	.	.	-4.8%
2010-2019	-2.6%	.	.	.	-2.6%
2000-2019	-3.7%	.	.	.	-3.7%
Death rate (per 100 000 population per year)					
2000	92.0	<0.1	<0.1	<0.1	29.7
2010	58.0	<0.1	<0.1	<0.1	16.1
2019	48.0	<0.1	<0.1	<0.1	11.7
Rate of change in death rate (% per year)					
2000-2010	-4.5%	.	.	.	-5.9%
2010-2019	-2.1%	.	.	.	-3.5%
2000-2019	-3.4%	.	.	.	-4.8%

Data source: Global Health Estimates 2021

Panel H7: Neonatal conditions - Middle East and North Africa

	Age group				
	0-14	15-49	50-69	70+	All ages
Population size (millions)					
2000	135	200	36.9	9.6	381
2010	140	259	53.6	13.8	467
2019	163	296	76.1	18.3	553
Rate of change in population size (% per year)					
2000-2010	+0.4%	+2.6%	+3.8%	+3.7%	+2.0%
2010-2019	+1.7%	+1.5%	+4.0%	+3.2%	+1.9%
2000-2019	+1.0%	+2.1%	+3.9%	+3.5%	+2.0%
Deaths (thousands)					
2000	167	<0.1	<0.1	<0.1	167
2010	133	<0.1	<0.1	<0.1	133
2019	104	<0.1	<0.1	<0.1	104
Rate of change in deaths (% per year)					
2000-2010	-2.2%	.	.	.	-2.2%
2010-2019	-2.7%	.	.	.	-2.7%
2000-2019	-2.5%	.	.	.	-2.5%
Death rate (per 100 000 population per year)					
2000	124	<0.1	<0.1	<0.1	43.8
2010	95.3	<0.1	<0.1	<0.1	28.6
2019	63.8	<0.1	<0.1	<0.1	18.8
Rate of change in death rate (% per year)					
2000-2010	-2.6%	.	.	.	-4.2%
2010-2019	-4.4%	.	.	.	-4.6%
2000-2019	-3.4%	.	.	.	-4.4%

Data source: Global Health Estimates 2021

Panel H8: Neonatal conditions - North Atlantic

	Age group				
	0-14	15-49	50-69	70+	All ages
Population size (millions)					
2000	72.0	209	93.2	47.7	422
2010	71.3	211	107	57.5	446
2019	72.1	202	122	67.8	464
Rate of change in population size (% per year)					
2000-2010	-0.1%	+0.1%	+1.4%	+1.9%	+0.6%
2010-2019	+0.1%	-0.4%	+1.5%	+1.9%	+0.4%
2000-2019	0.0%	-0.2%	+1.4%	+1.9%	+0.5%
Deaths (thousands)					
2000	10.9	<0.1	<0.1	<0.1	10.9
2010	9.6	<0.1	<0.1	<0.1	9.7
2019	9.6	0.1	<0.1	<0.1	9.8
Rate of change in deaths (% per year)					
2000-2010	-1.3%	.	.	.	-1.2%
2010-2019	0.0%	+8.6%	.	.	+0.2%
2000-2019	-0.7%	+7.0%	.	.	-0.6%
Death rate (per 100 000 population per year)					
2000	15.1	<0.1	<0.1	<0.1	2.6
2010	13.5	<0.1	<0.1	<0.1	2.2
2019	13.3	<0.1	<0.1	<0.1	2.1
Rate of change in death rate (% per year)					
2000-2010	-1.2%	.	.	.	-1.8%
2010-2019	-0.1%	.	.	.	-0.2%
2000-2019	-0.7%	.	.	.	-1.0%

Data source: Global Health Estimates 2021

Panel H9: Neonatal conditions - Sub-Saharan Africa

	Age group				
	0-14	15-49	50-69	70+	All ages
Population size (millions)					
2000	305	313	52.5	11.8	682
2010	393	418	69.0	15.6	895
2019	482	544	93.4	20.4	1,140
Rate of change in population size (% per year)					
2000-2010	+2.6%	+2.9%	+2.8%	+2.8%	+2.8%
2010-2019	+2.3%	+3.0%	+3.4%	+3.0%	+2.7%
2000-2019	+2.4%	+3.0%	+3.1%	+2.9%	+2.7%
Deaths (thousands)					
2000	889	<0.1	<0.1	<0.1	889
2010	913	<0.1	<0.1	<0.1	913
2019	906	<0.1	<0.1	<0.1	906
Rate of change in deaths (% per year)					
2000-2010	+0.3%	.	.	.	+0.3%
2010-2019	-0.1%	.	.	.	-0.1%
2000-2019	+0.1%	.	.	.	+0.1%
Death rate (per 100 000 population per year)					
2000	292	<0.1	<0.1	<0.1	130
2010	232	<0.1	<0.1	<0.1	102
2019	188	<0.1	<0.1	<0.1	79.5
Rate of change in death rate (% per year)					
2000-2010	-2.3%	.	.	.	-2.4%
2010-2019	-2.3%	.	.	.	-2.7%
2000-2019	-2.3%	.	.	.	-2.6%

Data source: Global Health Estimates 2021

Panel H10: Neonatal conditions - United States

	Age group				
	0-14	15-49	50-69	70+	All ages
Population size (millions)					
2000	59.6	146	51.4	24.9	281
2010	61.3	151	71.3	27.5	311
2019	62.9	157	82.2	35.5	338
Rate of change in population size (% per year)					
2000-2010	+0.3%	+0.4%	+3.3%	+1.0%	+1.0%
2010-2019	+0.3%	+0.4%	+1.6%	+2.9%	+0.9%
2000-2019	+0.3%	+0.4%	+2.5%	+1.9%	+1.0%
Deaths (thousands)					
2000	14.3	<0.1	<0.1	<0.1	14.4
2010	12.3	<0.1	<0.1	<0.1	12.4
2019	10.8	<0.1	<0.1	<0.1	10.8
Rate of change in deaths (% per year)					
2000-2010	-1.5%	.	.	.	-1.5%
2010-2019	-1.5%	.	.	.	-1.5%
2000-2019	-1.5%	.	.	.	-1.5%
Death rate (per 100 000 population per year)					
2000	24.0	<0.1	<0.1	<0.1	5.1
2010	20.1	<0.1	<0.1	<0.1	4.0
2019	17.2	<0.1	<0.1	<0.1	3.2
Rate of change in death rate (% per year)					
2000-2010	-1.8%	.	.	.	-2.4%
2010-2019	-1.8%	.	.	.	-2.4%
2000-2019	-1.8%	.	.	.	-2.4%

Data source: Global Health Estimates 2021

Panel H11: Neonatal conditions - Western Pacific and Southeast Asia

	Age group				
	0-14	15-49	50-69	70+	All ages
Population size (millions)					
2000	276	492	127	38.9	934
2010	275	549	163	56.0	1,040
2019	272	582	205	74.4	1,130
Rate of change in population size (% per year)					
2000-2010	0.0%	+1.1%	+2.5%	+3.7%	+1.1%
2010-2019	-0.1%	+0.6%	+2.6%	+3.2%	+0.9%
2000-2019	-0.1%	+0.9%	+2.5%	+3.5%	+1.0%
Deaths (thousands)					
2000	393	<0.1	<0.1	<0.1	393
2010	265	<0.1	<0.1	<0.1	265
2019	182	<0.1	<0.1	<0.1	182
Rate of change in deaths (% per year)					
2000-2010	-3.9%	.	.	.	-3.9%
2010-2019	-4.1%	.	.	.	-4.1%
2000-2019	-4.0%	.	.	.	-4.0%
Death rate (per 100 000 population per year)					
2000	142	<0.1	<0.1	<0.1	42.1
2010	96.2	<0.1	<0.1	<0.1	25.4
2019	67.1	<0.1	<0.1	<0.1	16.1
Rate of change in death rate (% per year)					
2000-2010	-3.8%	.	.	.	-4.9%
2010-2019	-3.9%	.	.	.	-4.9%
2000-2019	-3.9%	.	.	.	-4.9%

Data source: Global Health Estimates 2021

Panel I1: Tuberculosis - World

	Age group				
	0-14	15-49	50-69	70+	All ages
Population size (millions)					
2000	1,870	3,210	808	269	6,160
2010	1,910	3,670	1,080	355	7,010
2019	2,030	3,930	1,380	451	7,800
Rate of change in population size (% per year)					
2000-2010	+0.2%	+1.3%	+2.9%	+2.8%	+1.3%
2010-2019	+0.7%	+0.8%	+2.9%	+2.7%	+1.2%
2000-2019	+0.4%	+1.1%	+2.9%	+2.7%	+1.2%
Deaths (thousands)					
2000	338	800	811	569	2,520
2010	228	607	637	461	1,930
2019	144	369	461	352	1,330
Rate of change in deaths (% per year)					
2000-2010	-3.9%	-2.7%	-2.4%	-2.1%	-2.6%
2010-2019	-4.9%	-5.4%	-3.5%	-2.9%	-4.1%
2000-2019	-4.4%	-4.0%	-2.9%	-2.5%	-3.3%
Death rate (per 100 000 population per year)					
2000	18.1	24.9	100	211	40.9
2010	11.9	16.6	59.3	130	27.6
2019	7.1	9.4	33.3	78.1	17.0
Rate of change in death rate (% per year)					
2000-2010	-4.1%	-4.0%	-5.1%	-4.8%	-3.9%
2010-2019	-5.6%	-6.1%	-6.2%	-5.5%	-5.2%
2000-2019	-4.8%	-5.0%	-5.6%	-5.1%	-4.5%

Data source: Global Health Estimates 2021

Panel I2: Tuberculosis - Central and Eastern Europe

	Age group				
	0-14	15-49	50-69	70+	All ages
Population size (millions)					
2000	63.2	181	70.2	29.2	343
2010	50.2	169	78.6	34.2	332
2019	54.6	153	85.8	35.2	329
Rate of change in population size (% per year)					
2000-2010	-2.3%	-0.7%	+1.1%	+1.6%	-0.3%
2010-2019	+0.9%	-1.1%	+1.0%	+0.3%	-0.1%
2000-2019	-0.8%	-0.9%	+1.1%	+1.0%	-0.2%
Deaths (thousands)					
2000	0.5	26.3	22.3	4.8	53.8
2010	0.3	18.2	18.0	3.9	40.3
2019	<0.1	6.3	9.6	2.3	18.4
Rate of change in deaths (% per year)					
2000-2010	-5.0%	-3.6%	-2.1%	-2.2%	-2.9%
2010-2019	-12%	-11%	-6.7%	-5.6%	-8.4%
2000-2019	-8.4%	-7.2%	-4.3%	-3.8%	-5.5%
Death rate (per 100 000 population per year)					
2000	0.7	14.5	31.7	16.6	15.7
2010	0.6	10.8	22.8	11.3	12.1
2019	0.2	4.1	11.2	6.6	5.6
Rate of change in death rate (% per year)					
2000-2010	-2.8%	-2.9%	-3.2%	-3.8%	-2.5%
2010-2019	-13%	-10%	-7.6%	-5.9%	-8.3%
2000-2019	-7.6%	-6.4%	-5.3%	-4.8%	-5.3%

Data source: Global Health Estimates 2021

Panel I3: Tuberculosis - Central Asia

	Age group				
	0-14	15-49	50-69	70+	All ages
Population size (millions)					
2000	100	115	21.7	5.4	242
2010	114	153	29.3	7.3	303
2019	131	177	39.7	8.8	357
Rate of change in population size (% per year)					
2000-2010	+1.3%	+2.9%	+3.0%	+3.1%	+2.3%
2010-2019	+1.6%	+1.6%	+3.4%	+2.1%	+1.8%
2000-2019	+1.4%	+2.3%	+3.2%	+2.6%	+2.1%
Deaths (thousands)					
2000	14.5	33.4	25.4	13.1	86.4
2010	12.1	27.7	21.9	12.5	74.2
2019	9.0	21.0	19.5	11.7	61.2
Rate of change in deaths (% per year)					
2000-2010	-1.7%	-1.9%	-1.5%	-0.4%	-1.5%
2010-2019	-3.2%	-3.0%	-1.3%	-0.8%	-2.1%
2000-2019	-2.4%	-2.4%	-1.4%	-0.6%	-1.8%
Death rate (per 100 000 population per year)					
2000	14.5	29.1	117	241	35.7
2010	10.7	18.1	74.6	171	24.5
2019	6.9	11.8	49.1	132	17.2
Rate of change in death rate (% per year)					
2000-2010	-3.0%	-4.6%	-4.4%	-3.4%	-3.7%
2010-2019	-4.8%	-4.6%	-4.5%	-2.8%	-3.9%
2000-2019	-3.8%	-4.6%	-4.5%	-3.1%	-3.8%

Data source: Global Health Estimates 2021

Panel I4: Tuberculosis - China

	Age group				
	0-14	15-49	50-69	70+	All ages
Population size (millions)					
2000	312	723	180	54.9	1,270
2010	250	767	257	77.6	1,350
2019	259	708	354	103	1,420
Rate of change in population size (% per year)					
2000-2010	-2.2%	+0.6%	+3.6%	+3.5%	+0.6%
2010-2019	+0.4%	-0.9%	+3.6%	+3.1%	+0.6%
2000-2019	-1.0%	-0.1%	+3.6%	+3.3%	+0.6%
Deaths (thousands)					
2000	6.5	27.0	34.4	38.9	107
2010	1.4	11.7	18.3	23.5	54.9
2019	0.4	6.4	13.8	17.1	37.8
Rate of change in deaths (% per year)					
2000-2010	-14%	-8.0%	-6.1%	-4.9%	-6.4%
2010-2019	-13%	-6.4%	-3.1%	-3.4%	-4.1%
2000-2019	-13%	-7.3%	-4.7%	-4.2%	-5.3%
Death rate (per 100 000 population per year)					
2000	2.1	3.7	19.1	70.8	8.4
2010	0.6	1.5	7.1	30.3	4.1
2019	0.2	0.9	3.9	16.7	2.6
Rate of change in death rate (% per year)					
2000-2010	-12%	-8.6%	-9.4%	-8.1%	-7.0%
2010-2019	-13%	-5.6%	-6.5%	-6.4%	-4.6%
2000-2019	-13%	-7.2%	-8.0%	-7.3%	-5.9%

Data source: Global Health Estimates 2021

Panel I5: Tuberculosis - India

	Age group				
	0-14	15-49	50-69	70+	All ages
Population size (millions)					
2000	376	543	111	27.6	1,060
2010	390	659	157	37.8	1,240
2019	373	757	209	50.5	1,390
Rate of change in population size (% per year)					
2000-2010	+0.4%	+1.9%	+3.6%	+3.2%	+1.6%
2010-2019	-0.5%	+1.6%	+3.2%	+3.3%	+1.2%
2000-2019	0.0%	+1.8%	+3.4%	+3.2%	+1.4%
Deaths (thousands)					
2000	82.0	331	303	191	907
2010	31.9	204	212	124	572
2019	10.1	103	134	87.3	334
Rate of change in deaths (% per year)					
2000-2010	-9.0%	-4.7%	-3.5%	-4.2%	-4.5%
2010-2019	-12%	-7.3%	-5.0%	-3.8%	-5.8%
2000-2019	-10%	-6.0%	-4.2%	-4.0%	-5.1%
Death rate (per 100 000 population per year)					
2000	21.8	61.0	273	691	85.7
2010	8.2	30.9	135	327	46.0
2019	2.7	13.5	64.1	173	24.0
Rate of change in death rate (% per year)					
2000-2010	-9.3%	-6.6%	-6.8%	-7.2%	-6.0%
2010-2019	-12%	-8.8%	-7.9%	-6.8%	-7.0%
2000-2019	-10%	-7.6%	-7.3%	-7.0%	-6.5%

Data source: Global Health Estimates 2021

Panel I6: Tuberculosis - Latin America and Caribbean

	Age group				
	0-14	15-49	50-69	70+	All ages
Population size (millions)					
2000	166	272	59.4	17.7	515
2010	161	312	83.7	25.4	583
2019	155	337	110	34.6	636
Rate of change in population size (% per year)					
2000-2010	-0.3%	+1.4%	+3.5%	+3.7%	+1.2%
2010-2019	-0.5%	+0.8%	+3.1%	+3.5%	+1.0%
2000-2019	-0.4%	+1.1%	+3.3%	+3.6%	+1.1%
Deaths (thousands)					
2000	3.1	13.7	10.3	7.7	34.8
2010	1.0	9.2	8.5	6.6	25.2
2019	0.6	7.7	7.9	6.1	22.4
Rate of change in deaths (% per year)					
2000-2010	-11%	-3.9%	-2.0%	-1.5%	-3.2%
2010-2019	-4.5%	-1.9%	-0.7%	-0.9%	-1.3%
2000-2019	-8.0%	-3.0%	-1.4%	-1.2%	-2.3%
Death rate (per 100 000 population per year)					
2000	1.8	5.0	17.4	43.6	6.8
2010	0.6	3.0	10.1	26.1	4.3
2019	0.4	2.3	7.2	17.6	3.5
Rate of change in death rate (% per year)					
2000-2010	-11%	-5.2%	-5.3%	-5.0%	-4.3%
2010-2019	-4.0%	-2.8%	-3.7%	-4.3%	-2.3%
2000-2019	-7.6%	-4.0%	-4.6%	-4.7%	-3.4%

Data source: Global Health Estimates 2021

Panel I7: Tuberculosis - Middle East and North Africa

	Age group				
	0-14	15-49	50-69	70+	All ages
Population size (millions)					
2000	135	200	36.9	9.6	381
2010	140	259	53.6	13.8	467
2019	163	296	76.1	18.3	553
Rate of change in population size (% per year)					
2000-2010	+0.4%	+2.6%	+3.8%	+3.7%	+2.0%
2010-2019	+1.7%	+1.5%	+4.0%	+3.2%	+1.9%
2000-2019	+1.0%	+2.1%	+3.9%	+3.5%	+2.0%
Deaths (thousands)					
2000	2.6	4.5	4.3	4.0	15.4
2010	1.3	3.8	3.5	4.3	12.9
2019	1.0	3.4	3.8	4.6	12.8
Rate of change in deaths (% per year)					
2000-2010	-6.6%	-1.6%	-2.1%	+0.6%	-1.8%
2010-2019	-2.5%	-1.3%	+0.9%	+0.7%	-0.1%
2000-2019	-4.7%	-1.5%	-0.7%	+0.7%	-1.0%
Death rate (per 100 000 population per year)					
2000	1.9	2.3	11.7	41.9	4.0
2010	0.9	1.5	6.5	31.0	2.8
2019	0.6	1.1	5.0	24.9	2.3
Rate of change in death rate (% per year)					
2000-2010	-6.9%	-4.2%	-5.7%	-3.0%	-3.7%
2010-2019	-4.1%	-2.8%	-2.9%	-2.4%	-2.0%
2000-2019	-5.6%	-3.5%	-4.4%	-2.7%	-2.9%

Data source: Global Health Estimates 2021

Panel I8: Tuberculosis - North Atlantic

	Age group				
	0-14	15-49	50-69	70+	All ages
Population size (millions)					
2000	72.0	209	93.2	47.7	422
2010	71.3	211	107	57.5	446
2019	72.1	202	122	67.8	464
Rate of change in population size (% per year)					
2000-2010	-0.1%	+0.1%	+1.4%	+1.9%	+0.6%
2010-2019	+0.1%	-0.4%	+1.5%	+1.9%	+0.4%
2000-2019	0.0%	-0.2%	+1.4%	+1.9%	+0.5%
Deaths (thousands)					
2000	<0.1	0.4	1.0	3.2	4.6
2010	<0.1	0.3	0.5	2.2	3.0
2019	<0.1	0.2	0.4	1.4	2.0
Rate of change in deaths (% per year)					
2000-2010	.	-4.1%	-6.9%	-3.8%	-4.4%
2010-2019	.	-4.4%	-2.4%	-4.9%	-4.4%
2000-2019	.	-4.2%	-4.8%	-4.3%	-4.4%
Death rate (per 100 000 population per year)					
2000	<0.1	0.2	1.0	6.8	1.1
2010	<0.1	0.1	0.4	3.8	0.7
2019	<0.1	<0.1	0.3	2.1	0.4
Rate of change in death rate (% per year)					
2000-2010	.	-4.2%	-8.2%	-5.6%	-4.9%
2010-2019	.	-4.0%	-3.8%	-6.6%	-4.8%
2000-2019	.	-4.1%	-6.1%	-6.1%	-4.9%

Data source: Global Health Estimates 2021

Panel I9: Tuberculosis - Sub-Saharan Africa

	Age group				
	0-14	15-49	50-69	70+	All ages
Population size (millions)					
2000	305	313	52.5	11.8	682
2010	393	418	69.0	15.6	895
2019	482	544	93.4	20.4	1,140
Rate of change in population size (% per year)					
2000-2010	+2.6%	+2.9%	+2.8%	+2.8%	+2.8%
2010-2019	+2.3%	+3.0%	+3.4%	+3.0%	+2.7%
2000-2019	+2.4%	+3.0%	+3.1%	+2.9%	+2.7%
Deaths (thousands)					
2000	180	225	239	165	808
2010	156	233	219	156	764
2019	114	160	176	125	575
Rate of change in deaths (% per year)					
2000-2010	-1.5%	+0.4%	-0.9%	-0.5%	-0.6%
2010-2019	-3.4%	-4.1%	-2.3%	-2.5%	-3.1%
2000-2019	-2.4%	-1.8%	-1.6%	-1.4%	-1.8%
Death rate (per 100 000 population per year)					
2000	59.2	71.8	454	1,400	119
2010	39.6	55.8	317	1,000	85.3
2019	23.6	29.4	189	615	50.4
Rate of change in death rate (% per year)					
2000-2010	-4.0%	-2.5%	-3.5%	-3.2%	-3.2%
2010-2019	-5.6%	-6.9%	-5.6%	-5.3%	-5.7%
2000-2019	-4.7%	-4.6%	-4.5%	-4.2%	-4.4%

Data source: Global Health Estimates 2021

Panel I10: Tuberculosis - United States

	Age group				
	0-14	15-49	50-69	70+	All ages
Population size (millions)					
2000	59.6	146	51.4	24.9	281
2010	61.3	151	71.3	27.5	311
2019	62.9	157	82.2	35.5	338
Rate of change in population size (% per year)					
2000-2010	+0.3%	+0.4%	+3.3%	+1.0%	+1.0%
2010-2019	+0.3%	+0.4%	+1.6%	+2.9%	+0.9%
2000-2019	+0.3%	+0.4%	+2.5%	+1.9%	+1.0%
Deaths (thousands)					
2000	<0.1	0.1	0.2	0.6	0.9
2010	<0.1	<0.1	0.2	0.3	0.6
2019	<0.1	<0.1	0.2	0.3	0.6
Rate of change in deaths (% per year)					
2000-2010	.	-4.4%	-0.3%	-5.6%	-3.9%
2010-2019	.	.	-1.5%	-0.3%	-1.0%
2000-2019	.	-3.5%	-0.9%	-3.2%	-2.6%
Death rate (per 100 000 population per year)					
2000	<0.1	<0.1	0.4	2.3	0.3
2010	<0.1	<0.1	0.3	1.2	0.2
2019	<0.1	<0.1	0.2	0.9	0.2
Rate of change in death rate (% per year)					
2000-2010	.	.	-3.5%	-6.6%	-4.9%
2010-2019	.	.	-3.1%	-3.1%	-1.9%
2000-2019	.	.	-3.3%	-4.9%	-3.5%

Data source: Global Health Estimates 2021

Panel I11: Tuberculosis - Western Pacific and Southeast Asia

	Age group				
	0-14	15-49	50-69	70+	All ages
Population size (millions)					
2000	276	492	127	38.9	934
2010	275	549	163	56.0	1,040
2019	272	582	205	74.4	1,130
Rate of change in population size (% per year)					
2000-2010	0.0%	+1.1%	+2.5%	+3.7%	+1.1%
2010-2019	-0.1%	+0.6%	+2.6%	+3.2%	+0.9%
2000-2019	-0.1%	+0.9%	+2.5%	+3.5%	+1.0%
Deaths (thousands)					
2000	48.6	138	171	140	498
2010	24.4	99.0	136	126	385
2019	9.4	61.1	95.8	95.8	262
Rate of change in deaths (% per year)					
2000-2010	-6.7%	-3.3%	-2.3%	-1.0%	-2.5%
2010-2019	-10%	-5.2%	-3.8%	-3.0%	-4.2%
2000-2019	-8.3%	-4.2%	-3.0%	-2.0%	-3.3%
Death rate (per 100 000 population per year)					
2000	17.6	28.1	135	361	53.4
2010	8.9	18.0	83.3	226	36.9
2019	3.5	10.5	46.8	129	23.1
Rate of change in death rate (% per year)					
2000-2010	-6.6%	-4.3%	-4.7%	-4.6%	-3.6%
2010-2019	-9.9%	-5.8%	-6.2%	-6.0%	-5.1%
2000-2019	-8.2%	-5.0%	-5.4%	-5.3%	-4.3%

Data source: Global Health Estimates 2021

Table A9. Rates of change in population size and cause-specific number of deaths and death rates for NCD-7 conditions, 2000-2010, 2010-2019, and 2000-2019.

Panel A: NCD-7
Panel B: Atherosclerotic CVD
Panel C: Diabetes
Panel D: Hemorrhagic stroke
Panel E: Infection-associated NCDs
Panel F: Road injury
Panel G: Strongly tobacco-associated NCDs
Panel H: Suicide

Panel A1: NCD-7 - World

	Age group				
	0-14	15-49	50-69	70+	All ages
Population size (millions)					
2000	1,870	3,210	808	269	6,160
2010	1,910	3,670	1,080	355	7,010
2019	2,030	3,930	1,380	451	7,800
Rate of change in population size (% per year)					
2000-2010	+0.2%	+1.3%	+2.9%	+2.8%	+1.3%
2010-2019	+0.7%	+0.8%	+2.9%	+2.7%	+1.2%
2000-2019	+0.4%	+1.1%	+2.9%	+2.7%	+1.2%
Deaths (thousands)					
2000	253	2,960	6,790	11,900	21,900
2010	188	3,060	7,340	14,300	24,900
2019	158	2,880	8,790	16,000	27,900
Rate of change in deaths (% per year)					
2000-2010	-2.9%	+0.3%	+0.8%	+1.8%	+1.3%
2010-2019	-1.9%	-0.7%	+2.0%	+1.3%	+1.3%
2000-2019	-2.4%	-0.1%	+1.4%	+1.6%	+1.3%
Death rate (per 100 000 population per year)					
2000	13.5	92.1	840	4,430	356
2010	9.8	83.4	683	4,030	355
2019	7.8	73.3	635	3,560	358
Rate of change in death rate (% per year)					
2000-2010	-3.1%	-1.0%	-2.1%	-0.9%	0.0%
2010-2019	-2.5%	-1.4%	-0.8%	-1.4%	+0.1%
2000-2019	-2.8%	-1.2%	-1.5%	-1.1%	0.0%

Data source: Global Health Estimates 2021

Panel A2: NCD-7 - Central and Eastern Europe

	Age group				
	0-14	15-49	50-69	70+	All ages
Population size (millions)					
2000	63.2	181	70.2	29.2	343
2010	50.2	169	78.6	34.2	332
2019	54.6	153	85.8	35.2	329
Rate of change in population size (% per year)					
2000-2010	-2.3%	-0.7%	+1.1%	+1.6%	-0.3%
2010-2019	+0.9%	-1.1%	+1.0%	+0.3%	-0.1%
2000-2019	-0.8%	-0.9%	+1.1%	+1.0%	-0.2%
Deaths (thousands)					
2000	4.4	297	944	1,850	3,090
2010	2.2	204	735	1,900	2,840
2019	1.4	140	688	1,680	2,510
Rate of change in deaths (% per year)					
2000-2010	-6.8%	-3.7%	-2.5%	+0.3%	-0.9%
2010-2019	-4.6%	-4.0%	-0.7%	-1.4%	-1.4%
2000-2019	-5.8%	-3.9%	-1.7%	-0.5%	-1.1%
Death rate (per 100 000 population per year)					
2000	7.0	164	1,340	6,340	901
2010	4.4	121	934	5,560	855
2019	2.6	91.6	802	4,770	762
Rate of change in death rate (% per year)					
2000-2010	-4.6%	-3.0%	-3.6%	-1.3%	-0.5%
2010-2019	-5.5%	-3.0%	-1.7%	-1.7%	-1.3%
2000-2019	-5.0%	-3.0%	-2.7%	-1.5%	-0.9%

Data source: Global Health Estimates 2021

Panel A3: NCD-7 - Central Asia

	Age group				
	0-14	15-49	50-69	70+	All ages
Population size (millions)					
2000	100	115	21.7	5.4	242
2010	114	153	29.3	7.3	303
2019	131	177	39.7	8.8	357
Rate of change in population size (% per year)					
2000-2010	+1.3%	+2.9%	+3.0%	+3.1%	+2.3%
2010-2019	+1.6%	+1.6%	+3.4%	+2.1%	+1.8%
2000-2019	+1.4%	+2.3%	+3.2%	+2.6%	+2.1%
Deaths (thousands)					
2000	10.5	142	314	324	791
2010	10.3	165	335	423	934
2019	10.8	169	386	446	1,010
Rate of change in deaths (% per year)					
2000-2010	-0.3%	+1.5%	+0.7%	+2.7%	+1.7%
2010-2019	+0.5%	+0.2%	+1.6%	+0.6%	+0.9%
2000-2019	+0.1%	+0.9%	+1.1%	+1.7%	+1.3%
Death rate (per 100 000 population per year)					
2000	10.5	124	1,450	5,990	327
2010	9.0	108	1,140	5,780	308
2019	8.2	95.1	972	5,050	283
Rate of change in death rate (% per year)					
2000-2010	-1.5%	-1.4%	-2.3%	-0.3%	-0.6%
2010-2019	-1.1%	-1.4%	-1.8%	-1.5%	-0.9%
2000-2019	-1.3%	-1.4%	-2.1%	-0.9%	-0.8%

Data source: Global Health Estimates 2021

Panel A4: NCD-7 - China

	Age group				
	0-14	15-49	50-69	70+	All ages
Population size (millions)					
2000	312	723	180	54.9	1,270
2010	250	767	257	77.6	1,350
2019	259	708	354	103	1,420
Rate of change in population size (% per year)					
2000-2010	-2.2%	+0.6%	+3.6%	+3.5%	+0.6%
2010-2019	+0.4%	-0.9%	+3.6%	+3.1%	+0.6%
2000-2019	-1.0%	-0.1%	+3.6%	+3.3%	+0.6%
Deaths (thousands)					
2000	33.6	726	1,750	3,610	6,110
2010	15.7	658	1,800	4,710	7,190
2019	11.7	514	2,060	5,150	7,730
Rate of change in deaths (% per year)					
2000-2010	-7.3%	-1.0%	+0.3%	+2.7%	+1.6%
2010-2019	-3.2%	-2.7%	+1.5%	+1.0%	+0.8%
2000-2019	-5.4%	-1.8%	+0.9%	+1.9%	+1.2%
Death rate (per 100 000 population per year)					
2000	10.8	100	971	6,570	482
2010	6.3	85.8	703	6,070	532
2019	4.5	72.5	583	5,020	543
Rate of change in death rate (% per year)					
2000-2010	-5.2%	-1.6%	-3.2%	-0.8%	+1.0%
2010-2019	-3.6%	-1.8%	-2.1%	-2.1%	+0.2%
2000-2019	-4.5%	-1.7%	-2.7%	-1.4%	+0.6%

Data source: Global Health Estimates 2021

Panel A5: NCD-7 - India

	Age group				
	0-14	15-49	50-69	70+	All ages
Population size (millions)					
2000	376	543	111	27.6	1,060
2010	390	659	157	37.8	1,240
2019	373	757	209	50.5	1,390
Rate of change in population size (% per year)					
2000-2010	+0.4%	+1.9%	+3.6%	+3.2%	+1.6%
2010-2019	-0.5%	+1.6%	+3.2%	+3.3%	+1.2%
2000-2019	0.0%	+1.8%	+3.4%	+3.2%	+1.4%
Deaths (thousands)					
2000	50.6	557	987	918	2,510
2010	30.6	698	1,320	1,420	3,470
2019	18.3	699	1,960	2,100	4,770
Rate of change in deaths (% per year)					
2000-2010	-4.9%	+2.3%	+2.9%	+4.5%	+3.3%
2010-2019	-5.5%	0.0%	+4.5%	+4.4%	+3.6%
2000-2019	-5.2%	+1.2%	+3.7%	+4.4%	+3.4%
Death rate (per 100 000 population per year)					
2000	13.4	103	891	3,330	238
2010	7.8	106	838	3,770	279
2019	4.9	92.3	939	4,150	344
Rate of change in death rate (% per year)					
2000-2010	-5.2%	+0.3%	-0.6%	+1.2%	+1.6%
2010-2019	-5.1%	-1.5%	+1.3%	+1.1%	+2.3%
2000-2019	-5.2%	-0.6%	+0.3%	+1.2%	+2.0%

Data source: Global Health Estimates 2021

Panel A6: NCD-7 - Latin America and Caribbean

	Age group				
	0-14	15-49	50-69	70+	All ages
Population size (millions)					
2000	166	272	59.4	17.7	515
2010	161	312	83.7	25.4	583
2019	155	337	110	34.6	636
Rate of change in population size (% per year)					
2000-2010	-0.3%	+1.4%	+3.5%	+3.7%	+1.2%
2010-2019	-0.5%	+0.8%	+3.1%	+3.5%	+1.0%
2000-2019	-0.4%	+1.1%	+3.3%	+3.6%	+1.1%
Deaths (thousands)					
2000	13.0	179	360	584	1,140
2010	9.8	196	420	764	1,390
2019	6.8	196	501	913	1,620
Rate of change in deaths (% per year)					
2000-2010	-2.8%	+0.9%	+1.6%	+2.7%	+2.0%
2010-2019	-4.0%	0.0%	+2.0%	+2.0%	+1.7%
2000-2019	-3.4%	+0.5%	+1.8%	+2.4%	+1.9%
Death rate (per 100 000 population per year)					
2000	7.8	66.0	606	3,300	221
2010	6.1	62.7	501	3,010	238
2019	4.4	58.2	454	2,640	254
Rate of change in death rate (% per year)					
2000-2010	-2.5%	-0.5%	-1.9%	-0.9%	+0.8%
2010-2019	-3.5%	-0.8%	-1.1%	-1.5%	+0.7%
2000-2019	-3.0%	-0.7%	-1.5%	-1.2%	+0.8%

Data source: Global Health Estimates 2021

Panel A7: NCD-7 - Middle East and North Africa

	Age group				
	0-14	15-49	50-69	70+	All ages
Population size (millions)					
2000	135	200	36.9	9.6	381
2010	140	259	53.6	13.8	467
2019	163	296	76.1	18.3	553
Rate of change in population size (% per year)					
2000-2010	+0.4%	+2.6%	+3.8%	+3.7%	+2.0%
2010-2019	+1.7%	+1.5%	+4.0%	+3.2%	+1.9%
2000-2019	+1.0%	+2.1%	+3.9%	+3.5%	+2.0%
Deaths (thousands)					
2000	23.1	148	316	397	884
2010	18.8	173	386	549	1,130
2019	15.2	169	492	722	1,400
Rate of change in deaths (% per year)					
2000-2010	-2.0%	+1.5%	+2.0%	+3.3%	+2.5%
2010-2019	-2.3%	-0.2%	+2.7%	+3.1%	+2.4%
2000-2019	-2.2%	+0.7%	+2.4%	+3.2%	+2.4%
Death rate (per 100 000 population per year)					
2000	17.1	74.3	857	4,130	232
2010	13.4	66.6	721	3,970	241
2019	9.4	57.1	646	3,940	253
Rate of change in death rate (% per year)					
2000-2010	-2.4%	-1.1%	-1.7%	-0.4%	+0.4%
2010-2019	-3.9%	-1.7%	-1.2%	-0.1%	+0.5%
2000-2019	-3.1%	-1.4%	-1.5%	-0.2%	+0.4%

Data source: Global Health Estimates 2021

Panel A8: NCD-7 - North Atlantic

	Age group				
	0-14	15-49	50-69	70+	All ages
Population size (millions)					
2000	72.0	209	93.2	47.7	422
2010	71.3	211	107	57.5	446
2019	72.1	202	122	67.8	464
Rate of change in population size (% per year)					
2000-2010	-0.1%	+0.1%	+1.4%	+1.9%	+0.6%
2010-2019	+0.1%	-0.4%	+1.5%	+1.9%	+0.4%
2000-2019	0.0%	-0.2%	+1.4%	+1.9%	+0.5%
Deaths (thousands)					
2000	2.2	113	399	1,560	2,080
2010	1.1	81.8	335	1,400	1,820
2019	0.7	60.3	323	1,310	1,700
Rate of change in deaths (% per year)					
2000-2010	-7.1%	-3.2%	-1.7%	-1.1%	-1.3%
2010-2019	-5.2%	-3.3%	-0.4%	-0.7%	-0.8%
2000-2019	-6.2%	-3.3%	-1.1%	-0.9%	-1.1%
Death rate (per 100 000 population per year)					
2000	3.0	54.1	428	3,280	493
2010	1.5	38.8	314	2,440	408
2019	0.9	29.8	266	1,940	366
Rate of change in death rate (% per year)					
2000-2010	-7.0%	-3.3%	-3.1%	-2.9%	-1.9%
2010-2019	-5.3%	-2.9%	-1.8%	-2.5%	-1.2%
2000-2019	-6.2%	-3.1%	-2.5%	-2.7%	-1.6%

Data source: Global Health Estimates 2021

Panel A9: NCD-7 - Sub-Saharan Africa

	Age group				
	0-14	15-49	50-69	70+	All ages
Population size (millions)					
2000	305	313	52.5	11.8	682
2010	393	418	69.0	15.6	895
2019	482	544	93.4	20.4	1,140
Rate of change in population size (% per year)					
2000-2010	+2.6%	+2.9%	+2.8%	+2.8%	+2.8%
2010-2019	+2.3%	+3.0%	+3.4%	+3.0%	+2.7%
2000-2019	+2.4%	+3.0%	+3.1%	+2.9%	+2.7%
Deaths (thousands)					
2000	79.0	232	460	424	1,200
2010	71.1	303	549	548	1,470
2019	72.0	370	676	694	1,810
Rate of change in deaths (% per year)					
2000-2010	-1.0%	+2.7%	+1.8%	+2.6%	+2.1%
2010-2019	+0.1%	+2.2%	+2.3%	+2.7%	+2.3%
2000-2019	-0.5%	+2.5%	+2.0%	+2.6%	+2.2%
Death rate (per 100 000 population per year)					
2000	25.9	74.2	876	3,600	175
2010	18.1	72.5	796	3,520	164
2019	14.9	67.9	723	3,410	159
Rate of change in death rate (% per year)					
2000-2010	-3.5%	-0.2%	-0.9%	-0.2%	-0.6%
2010-2019	-2.1%	-0.7%	-1.1%	-0.3%	-0.4%
2000-2019	-2.9%	-0.5%	-1.0%	-0.3%	-0.5%

Data source: Global Health Estimates 2021

Panel A10: NCD-7 - United States

	Age group				
	0-14	15-49	50-69	70+	All ages
Population size (millions)					
2000	59.6	146	51.4	24.9	281
2010	61.3	151	71.3	27.5	311
2019	62.9	157	82.2	35.5	338
Rate of change in population size (% per year)					
2000-2010	+0.3%	+0.4%	+3.3%	+1.0%	+1.0%
2010-2019	+0.3%	+0.4%	+1.6%	+2.9%	+0.9%
2000-2019	+0.3%	+0.4%	+2.5%	+1.9%	+1.0%
Deaths (thousands)					
2000	3.0	97.6	274	925	1,300
2010	1.9	86.9	300	752	1,140
2019	2.0	86.2	330	793	1,210
Rate of change in deaths (% per year)					
2000-2010	-4.8%	-1.2%	+0.9%	-2.1%	-1.3%
2010-2019	+0.9%	-0.1%	+1.1%	+0.6%	+0.7%
2000-2019	-2.1%	-0.6%	+1.0%	-0.8%	-0.4%
Death rate (per 100 000 population per year)					
2000	5.1	67.0	534	3,720	462
2010	3.0	57.6	420	2,730	366
2019	3.2	54.8	401	2,240	359
Rate of change in death rate (% per year)					
2000-2010	-5.0%	-1.5%	-2.4%	-3.0%	-2.3%
2010-2019	+0.6%	-0.5%	-0.5%	-2.2%	-0.2%
2000-2019	-2.4%	-1.1%	-1.5%	-2.6%	-1.3%

Data source: Global Health Estimates 2021

Panel A11: NCD-7 - Western Pacific and Southeast Asia

	Age group				
	0-14	15-49	50-69	70+	All ages
Population size (millions)					
2000	276	492	127	38.9	934
2010	275	549	163	56.0	1,040
2019	272	582	205	74.4	1,130
Rate of change in population size (% per year)					
2000-2010	0.0%	+1.1%	+2.5%	+3.7%	+1.1%
2010-2019	-0.1%	+0.6%	+2.6%	+3.2%	+0.9%
2000-2019	-0.1%	+0.9%	+2.5%	+3.5%	+1.0%
Deaths (thousands)					
2000	33.1	451	958	1,290	2,730
2010	26.3	480	1,130	1,790	3,430
2019	19.3	468	1,340	2,180	4,000
Rate of change in deaths (% per year)					
2000-2010	-2.3%	+0.6%	+1.7%	+3.3%	+2.3%
2010-2019	-3.4%	-0.3%	+1.9%	+2.2%	+1.7%
2000-2019	-2.8%	+0.2%	+1.8%	+2.8%	+2.0%
Death rate (per 100 000 population per year)					
2000	12.0	91.6	753	3,320	293
2010	9.6	87.5	695	3,200	329
2019	7.1	80.5	655	2,920	354
Rate of change in death rate (% per year)					
2000-2010	-2.2%	-0.5%	-0.8%	-0.4%	+1.2%
2010-2019	-3.2%	-0.9%	-0.7%	-1.0%	+0.8%
2000-2019	-2.7%	-0.7%	-0.7%	-0.7%	+1.0%

Data source: Global Health Estimates 2021

Panel B1: Atherosclerotic CVD - World

	Age group				
	0-14	15-49	50-69	70+	All ages
Population size (millions)					
2000	1,870	3,210	808	269	6,160
2010	1,910	3,670	1,080	355	7,010
2019	2,030	3,930	1,380	451	7,800
Rate of change in population size (% per year)					
2000-2010	+0.2%	+1.3%	+2.9%	+2.8%	+1.3%
2010-2019	+0.7%	+0.8%	+2.9%	+2.7%	+1.2%
2000-2019	+0.4%	+1.1%	+2.9%	+2.7%	+1.2%
Deaths (thousands)					
2000	3.9	565	2,350	6,070	8,990
2010	3.2	633	2,580	7,380	10,600
2019	2.7	639	3,190	8,230	12,100
Rate of change in deaths (% per year)					
2000-2010	-2.0%	+1.2%	+0.9%	+2.0%	+1.6%
2010-2019	-1.8%	+0.1%	+2.4%	+1.2%	+1.5%
2000-2019	-1.9%	+0.6%	+1.6%	+1.6%	+1.6%
Death rate (per 100 000 population per year)					
2000	0.2	17.6	292	2,250	146
2010	0.2	17.3	240	2,080	151
2019	0.1	16.3	231	1,820	155
Rate of change in death rate (% per year)					
2000-2010	-2.2%	-0.2%	-1.9%	-0.8%	+0.3%
2010-2019	-2.4%	-0.7%	-0.4%	-1.4%	+0.3%
2000-2019	-2.3%	-0.4%	-1.2%	-1.1%	+0.3%

Data source: Global Health Estimates 2021

Panel B2: Atherosclerotic CVD - Central and Eastern Europe

	Age group				
	0-14	15-49	50-69	70+	All ages
Population size (millions)					
2000	63.2	181	70.2	29.2	343
2010	50.2	169	78.6	34.2	332
2019	54.6	153	85.8	35.2	329
Rate of change in population size (% per year)					
2000-2010	-2.3%	-0.7%	+1.1%	+1.6%	-0.3%
2010-2019	+0.9%	-1.1%	+1.0%	+0.3%	-0.1%
2000-2019	-0.8%	-0.9%	+1.1%	+1.0%	-0.2%
Deaths (thousands)					
2000	<0.1	87.9	536	1,500	2,120
2010	<0.1	58.3	420	1,570	2,050
2019	<0.1	38.0	379	1,350	1,770
Rate of change in deaths (% per year)					
2000-2010	.	-4.0%	-2.4%	+0.5%	-0.3%
2010-2019	.	-4.7%	-1.1%	-1.6%	-1.6%
2000-2019	.	-4.3%	-1.8%	-0.5%	-0.9%
Death rate (per 100 000 population per year)					
2000	<0.1	48.7	764	5,140	618
2010	<0.1	34.5	534	4,600	618
2019	<0.1	24.7	442	3,840	538
Rate of change in death rate (% per year)					
2000-2010	.	-3.4%	-3.5%	-1.1%	0.0%
2010-2019	.	-3.6%	-2.1%	-2.0%	-1.5%
2000-2019	.	-3.5%	-2.8%	-1.5%	-0.7%

Data source: Global Health Estimates 2021

Panel B3: Atherosclerotic CVD - Central Asia

	Age group				
	0-14	15-49	50-69	70+	All ages
Population size (millions)					
2000	100	115	21.7	5.4	242
2010	114	153	29.3	7.3	303
2019	131	177	39.7	8.8	357
Rate of change in population size (% per year)					
2000-2010	+1.3%	+2.9%	+3.0%	+3.1%	+2.3%
2010-2019	+1.6%	+1.6%	+3.4%	+2.1%	+1.8%
2000-2019	+1.4%	+2.3%	+3.2%	+2.6%	+2.1%
Deaths (thousands)					
2000	0.2	39.5	144	193	377
2010	0.2	44.8	150	259	454
2019	0.2	47.5	178	271	497
Rate of change in deaths (% per year)					
2000-2010	-0.7%	+1.3%	+0.5%	+3.0%	+1.9%
2010-2019	+1.1%	+0.7%	+1.9%	+0.5%	+1.0%
2000-2019	+0.2%	+1.0%	+1.1%	+1.8%	+1.5%
Death rate (per 100 000 population per year)					
2000	0.2	34.4	661	3,570	156
2010	0.2	29.3	513	3,540	150
2019	0.2	26.8	449	3,070	139
Rate of change in death rate (% per year)					
2000-2010	-1.9%	-1.6%	-2.5%	-0.1%	-0.4%
2010-2019	-0.5%	-1.0%	-1.5%	-1.6%	-0.8%
2000-2019	-1.3%	-1.3%	-2.0%	-0.8%	-0.6%

Data source: Global Health Estimates 2021

Panel B4: Atherosclerotic CVD - China

	Age group				
	0-14	15-49	50-69	70+	All ages
Population size (millions)					
2000	312	723	180	54.9	1,270
2010	250	767	257	77.6	1,350
2019	259	708	354	103	1,420
Rate of change in population size (% per year)					
2000-2010	-2.2%	+0.6%	+3.6%	+3.5%	+0.6%
2010-2019	+0.4%	-0.9%	+3.6%	+3.1%	+0.6%
2000-2019	-1.0%	-0.1%	+3.6%	+3.3%	+0.6%
Deaths (thousands)					
2000	0.3	90.4	357	1,030	1,480
2010	0.2	111	464	1,860	2,430
2019	<0.1	94.8	587	2,260	2,940
Rate of change in deaths (% per year)					
2000-2010	-4.4%	+2.0%	+2.7%	+6.1%	+5.1%
2010-2019	-7.2%	-1.7%	+2.6%	+2.2%	+2.1%
2000-2019	-5.7%	+0.2%	+2.7%	+4.2%	+3.7%
Death rate (per 100 000 population per year)					
2000	<0.1	12.5	198	1,880	117
2010	<0.1	14.4	181	2,400	180
2019	<0.1	13.4	166	2,210	207
Rate of change in death rate (% per year)					
2000-2010	.	+1.4%	-0.9%	+2.5%	+4.4%
2010-2019	.	-0.8%	-0.9%	-0.9%	+1.5%
2000-2019	.	+0.4%	-0.9%	+0.8%	+3.1%

Data source: Global Health Estimates 2021

Panel B5: Atherosclerotic CVD - India

	Age group				
	0-14	15-49	50-69	70+	All ages
Population size (millions)					
2000	376	543	111	27.6	1,060
2010	390	659	157	37.8	1,240
2019	373	757	209	50.5	1,390
Rate of change in population size (% per year)					
2000-2010	+0.4%	+1.9%	+3.6%	+3.2%	+1.6%
2010-2019	-0.5%	+1.6%	+3.2%	+3.3%	+1.2%
2000-2019	0.0%	+1.8%	+3.4%	+3.2%	+1.4%
Deaths (thousands)					
2000	0.7	118	360	371	850
2010	0.3	168	506	586	1,260
2019	0.2	194	786	888	1,870
Rate of change in deaths (% per year)					
2000-2010	-6.5%	+3.6%	+3.5%	+4.7%	+4.0%
2010-2019	-7.8%	+1.6%	+5.0%	+4.7%	+4.5%
2000-2019	-7.1%	+2.7%	+4.2%	+4.7%	+4.2%
Death rate (per 100 000 population per year)					
2000	0.2	21.7	325	1,350	80.3
2010	<0.1	25.6	322	1,550	101
2019	<0.1	25.7	377	1,760	135
Rate of change in death rate (% per year)					
2000-2010	-6.8%	+1.6%	-0.1%	+1.4%	+2.4%
2010-2019	.	0.0%	+1.8%	+1.4%	+3.2%
2000-2019	-7.1%	+0.9%	+0.8%	+1.4%	+2.8%

Data source: Global Health Estimates 2021

Panel B6: Atherosclerotic CVD - Latin America and Caribbean

	Age group				
	0-14	15-49	50-69	70+	All ages
Population size (millions)					
2000	166	272	59.4	17.7	515
2010	161	312	83.7	25.4	583
2019	155	337	110	34.6	636
Rate of change in population size (% per year)					
2000-2010	-0.3%	+1.4%	+3.5%	+3.7%	+1.2%
2010-2019	-0.5%	+0.8%	+3.1%	+3.5%	+1.0%
2000-2019	-0.4%	+1.1%	+3.3%	+3.6%	+1.1%
Deaths (thousands)					
2000	0.2	32.4	129	316	478
2010	0.2	32.7	144	401	578
2019	0.1	35.7	176	477	689
Rate of change in deaths (% per year)					
2000-2010	-3.0%	+0.1%	+1.1%	+2.4%	+1.9%
2010-2019	-2.7%	+1.0%	+2.3%	+1.9%	+2.0%
2000-2019	-2.9%	+0.5%	+1.6%	+2.2%	+1.9%
Death rate (per 100 000 population per year)					
2000	0.1	11.9	218	1,790	92.7
2010	<0.1	10.5	171	1,580	99.1
2019	<0.1	10.6	160	1,380	108
Rate of change in death rate (% per year)					
2000-2010	-2.7%	-1.3%	-2.4%	-1.2%	+0.7%
2010-2019	.	+0.1%	-0.8%	-1.5%	+1.0%
2000-2019	-2.5%	-0.6%	-1.6%	-1.4%	+0.8%

Data source: Global Health Estimates 2021

Panel B7: Atherosclerotic CVD - Middle East and North Africa

	Age group				
	0-14	15-49	50-69	70+	All ages
Population size (millions)					
2000	135	200	36.9	9.6	381
2010	140	259	53.6	13.8	467
2019	163	296	76.1	18.3	553
Rate of change in population size (% per year)					
2000-2010	+0.4%	+2.6%	+3.8%	+3.7%	+2.0%
2010-2019	+1.7%	+1.5%	+4.0%	+3.2%	+1.9%
2000-2019	+1.0%	+2.1%	+3.9%	+3.5%	+2.0%
Deaths (thousands)					
2000	0.8	49.0	166	259	475
2010	0.6	53.4	194	355	604
2019	0.4	56.0	249	471	776
Rate of change in deaths (% per year)					
2000-2010	-3.7%	+0.9%	+1.6%	+3.2%	+2.4%
2010-2019	-3.5%	+0.5%	+2.8%	+3.2%	+2.8%
2000-2019	-3.6%	+0.7%	+2.1%	+3.2%	+2.6%
Death rate (per 100 000 population per year)					
2000	0.6	24.5	451	2,690	125
2010	0.4	20.6	363	2,570	129
2019	0.2	18.9	327	2,570	140
Rate of change in death rate (% per year)					
2000-2010	-4.0%	-1.7%	-2.1%	-0.4%	+0.4%
2010-2019	-5.1%	-1.0%	-1.2%	0.0%	+0.9%
2000-2019	-4.5%	-1.4%	-1.7%	-0.2%	+0.6%

Data source: Global Health Estimates 2021

Panel B8: Atherosclerotic CVD - North Atlantic

	Age group				
	0-14	15-49	50-69	70+	All ages
Population size (millions)					
2000	72.0	209	93.2	47.7	422
2010	71.3	211	107	57.5	446
2019	72.1	202	122	67.8	464
Rate of change in population size (% per year)					
2000-2010	-0.1%	+0.1%	+1.4%	+1.9%	+0.6%
2010-2019	+0.1%	-0.4%	+1.5%	+1.9%	+0.4%
2000-2019	0.0%	-0.2%	+1.4%	+1.9%	+0.5%
Deaths (thousands)					
2000	<0.1	20.7	156	1,010	1,190
2010	<0.1	15.8	106	824	946
2019	<0.1	10.3	94.9	708	813
Rate of change in deaths (% per year)					
2000-2010	.	-2.7%	-3.8%	-2.0%	-2.3%
2010-2019	.	-4.6%	-1.2%	-1.7%	-1.7%
2000-2019	.	-3.6%	-2.6%	-1.9%	-2.0%
Death rate (per 100 000 population per year)					
2000	<0.1	9.9	167	2,120	282
2010	<0.1	7.5	99.1	1,430	212
2019	<0.1	5.1	78.0	1,040	175
Rate of change in death rate (% per year)					
2000-2010	.	-2.8%	-5.1%	-3.9%	-2.8%
2010-2019	.	-4.2%	-2.6%	-3.5%	-2.1%
2000-2019	.	-3.4%	-3.9%	-3.7%	-2.5%

Data source: Global Health Estimates 2021

Panel B9: Atherosclerotic CVD - Sub-Saharan Africa

	Age group				
	0-14	15-49	50-69	70+	All ages
Population size (millions)					
2000	305	313	52.5	11.8	682
2010	393	418	69.0	15.6	895
2019	482	544	93.4	20.4	1,140
Rate of change in population size (% per year)					
2000-2010	+2.6%	+2.9%	+2.8%	+2.8%	+2.8%
2010-2019	+2.3%	+3.0%	+3.4%	+3.0%	+2.7%
2000-2019	+2.4%	+3.0%	+3.1%	+2.9%	+2.7%
Deaths (thousands)					
2000	1.2	27.1	125	188	341
2010	1.4	37.8	153	249	441
2019	1.3	47.4	193	321	563
Rate of change in deaths (% per year)					
2000-2010	+1.6%	+3.4%	+2.1%	+2.8%	+2.6%
2010-2019	-0.3%	+2.5%	+2.6%	+2.9%	+2.7%
2000-2019	+0.7%	+3.0%	+2.3%	+2.9%	+2.7%
Death rate (per 100 000 population per year)					
2000	0.4	8.7	237	1,600	50.0
2010	0.3	9.0	221	1,600	49.3
2019	0.3	8.7	207	1,580	49.4
Rate of change in death rate (% per year)					
2000-2010	-1.0%	+0.4%	-0.7%	0.0%	-0.2%
2010-2019	-2.6%	-0.4%	-0.8%	-0.1%	0.0%
2000-2019	-1.7%	0.0%	-0.7%	-0.1%	-0.1%

Data source: Global Health Estimates 2021

Panel B10: Atherosclerotic CVD - United States

	Age group				
	0-14	15-49	50-69	70+	All ages
Population size (millions)					
2000	59.6	146	51.4	24.9	281
2010	61.3	151	71.3	27.5	311
2019	62.9	157	82.2	35.5	338
Rate of change in population size (% per year)					
2000-2010	+0.3%	+0.4%	+3.3%	+1.0%	+1.0%
2010-2019	+0.3%	+0.4%	+1.6%	+2.9%	+0.9%
2000-2019	+0.3%	+0.4%	+2.5%	+1.9%	+1.0%
Deaths (thousands)					
2000	<0.1	21.6	116	598	736
2010	<0.1	18.3	110	427	555
2019	<0.1	14.8	122	430	567
Rate of change in deaths (% per year)					
2000-2010	.	-1.6%	-0.6%	-3.3%	-2.8%
2010-2019	.	-2.3%	+1.2%	+0.1%	+0.2%
2000-2019	.	-2.0%	+0.3%	-1.7%	-1.4%
Death rate (per 100 000 population per year)					
2000	0.1	14.8	226	2,400	261
2010	<0.1	12.1	154	1,550	179
2019	<0.1	9.4	149	1,210	168
Rate of change in death rate (% per year)					
2000-2010	-5.2%	-2.0%	-3.8%	-4.3%	-3.7%
2010-2019	.	-2.8%	-0.4%	-2.7%	-0.7%
2000-2019	-2.9%	-2.4%	-2.2%	-3.5%	-2.3%

Data source: Global Health Estimates 2021

Panel B11: Atherosclerotic CVD - Western Pacific and Southeast Asia

	Age group				
	0-14	15-49	50-69	70+	All ages
Population size (millions)					
2000	276	492	127	38.9	934
2010	275	549	163	56.0	1,040
2019	272	582	205	74.4	1,130
Rate of change in population size (% per year)					
2000-2010	0.0%	+1.1%	+2.5%	+3.7%	+1.1%
2010-2019	-0.1%	+0.6%	+2.6%	+3.2%	+0.9%
2000-2019	-0.1%	+0.9%	+2.5%	+3.5%	+1.0%
Deaths (thousands)					
2000	0.5	76.5	260	582	919
2010	0.4	91.7	327	821	1,240
2019	0.4	98.9	421	1,020	1,540
Rate of change in deaths (% per year)					
2000-2010	-2.6%	+1.8%	+2.3%	+3.5%	+3.0%
2010-2019	+0.1%	+0.8%	+2.9%	+2.4%	+2.4%
2000-2019	-1.3%	+1.4%	+2.6%	+3.0%	+2.8%
Death rate (per 100 000 population per year)					
2000	0.2	15.6	204	1,500	98.4
2010	0.1	16.7	201	1,470	119
2019	0.1	17.0	206	1,370	136
Rate of change in death rate (% per year)					
2000-2010	-2.6%	+0.7%	-0.2%	-0.2%	+1.9%
2010-2019	+0.2%	+0.2%	+0.3%	-0.7%	+1.5%
2000-2019	-1.3%	+0.5%	0.0%	-0.5%	+1.7%

Data source: Global Health Estimates 2021

Panel C1: Diabetes - World

	Age group				
	0-14	15-49	50-69	70+	All ages
Population size (millions)					
2000	1,870	3,210	808	269	6,160
2010	1,910	3,670	1,080	355	7,010
2019	2,030	3,930	1,380	451	7,800
Rate of change in population size (% per year)					
2000-2010	+0.2%	+1.3%	+2.9%	+2.8%	+1.3%
2010-2019	+0.7%	+0.8%	+2.9%	+2.7%	+1.2%
2000-2019	+0.4%	+1.1%	+2.9%	+2.7%	+1.2%
Deaths (thousands)					
2000	6.7	117	418	542	1,080
2010	5.9	137	545	786	1,470
2019	5.7	159	769	1,070	2,010
Rate of change in deaths (% per year)					
2000-2010	-1.3%	+1.7%	+2.7%	+3.8%	+3.1%
2010-2019	-0.3%	+1.6%	+3.9%	+3.5%	+3.5%
2000-2019	-0.8%	+1.7%	+3.3%	+3.7%	+3.3%
Death rate (per 100 000 population per year)					
2000	0.4	3.6	51.7	201	17.6
2010	0.3	3.8	50.7	221	21.0
2019	0.3	4.0	55.6	238	25.8
Rate of change in death rate (% per year)					
2000-2010	-1.5%	+0.3%	-0.2%	+1.0%	+1.8%
2010-2019	-1.0%	+0.9%	+1.0%	+0.8%	+2.3%
2000-2019	-1.3%	+0.6%	+0.4%	+0.9%	+2.0%

Data source: Global Health Estimates 2021

Panel C2: Diabetes - Central and Eastern Europe

	Age group				
	0-14	15-49	50-69	70+	All ages
Population size (millions)					
2000	63.2	181	70.2	29.2	343
2010	50.2	169	78.6	34.2	332
2019	54.6	153	85.8	35.2	329
Rate of change in population size (% per year)					
2000-2010	-2.3%	-0.7%	+1.1%	+1.6%	-0.3%
2010-2019	+0.9%	-1.1%	+1.0%	+0.3%	-0.1%
2000-2019	-0.8%	-0.9%	+1.1%	+1.0%	-0.2%
Deaths (thousands)					
2000	<0.1	4.2	15.9	19.6	39.8
2010	<0.1	2.5	13.4	26.1	42.1
2019	<0.1	3.0	24.4	57.1	84.6
Rate of change in deaths (% per year)					
2000-2010	.	-5.0%	-1.7%	+2.9%	+0.6%
2010-2019	.	+2.0%	+6.9%	+9.1%	+8.1%
2000-2019	.	-1.8%	+2.3%	+5.8%	+4.0%
Death rate (per 100 000 population per year)					
2000	0.1	2.4	22.7	67.2	11.6
2010	<0.1	1.5	17.1	76.3	12.7
2019	0.1	2.0	28.5	162	25.7
Rate of change in death rate (% per year)					
2000-2010	-4.4%	-4.3%	-2.8%	+1.3%	+0.9%
2010-2019	+3.3%	+3.1%	+5.8%	+8.7%	+8.2%
2000-2019	-0.9%	-0.9%	+1.2%	+4.7%	+4.3%

Data source: Global Health Estimates 2021

Panel C3: Diabetes - Central Asia

	Age group				
	0-14	15-49	50-69	70+	All ages
Population size (millions)					
2000	100	115	21.7	5.4	242
2010	114	153	29.3	7.3	303
2019	131	177	39.7	8.8	357
Rate of change in population size (% per year)					
2000-2010	+1.3%	+2.9%	+3.0%	+3.1%	+2.3%
2010-2019	+1.6%	+1.6%	+3.4%	+2.1%	+1.8%
2000-2019	+1.4%	+2.3%	+3.2%	+2.6%	+2.1%
Deaths (thousands)					
2000	0.2	6.3	22.3	17.8	46.6
2010	0.2	8.0	30.8	27.8	66.8
2019	0.3	9.1	39.4	35.7	84.6
Rate of change in deaths (% per year)					
2000-2010	-0.8%	+2.4%	+3.3%	+4.6%	+3.7%
2010-2019	+1.5%	+1.5%	+2.8%	+2.8%	+2.6%
2000-2019	+0.3%	+2.0%	+3.0%	+3.7%	+3.2%
Death rate (per 100 000 population per year)					
2000	0.2	5.5	103	328	19.3
2010	0.2	5.2	105	379	22.0
2019	0.2	5.1	99.4	405	23.7
Rate of change in death rate (% per year)					
2000-2010	-2.1%	-0.5%	+0.2%	+1.5%	+1.3%
2010-2019	-0.1%	-0.1%	-0.6%	+0.7%	+0.8%
2000-2019	-1.1%	-0.3%	-0.2%	+1.1%	+1.1%

Data source: Global Health Estimates 2021

Panel C4: Diabetes - China

	Age group				
	0-14	15-49	50-69	70+	All ages
Population size (millions)					
2000	312	723	180	54.9	1,270
2010	250	767	257	77.6	1,350
2019	259	708	354	103	1,420
Rate of change in population size (% per year)					
2000-2010	-2.2%	+0.6%	+3.6%	+3.5%	+0.6%
2010-2019	+0.4%	-0.9%	+3.6%	+3.1%	+0.6%
2000-2019	-1.0%	-0.1%	+3.6%	+3.3%	+0.6%
Deaths (thousands)					
2000	0.4	25.5	65.5	92.9	184
2010	0.2	23.5	74.1	139	237
2019	0.2	19.4	93.3	172	285
Rate of change in deaths (% per year)					
2000-2010	-6.3%	-0.8%	+1.2%	+4.1%	+2.5%
2010-2019	-3.5%	-2.1%	+2.6%	+2.4%	+2.1%
2000-2019	-5.0%	-1.4%	+1.9%	+3.3%	+2.3%
Death rate (per 100 000 population per year)					
2000	0.1	3.5	36.4	169	14.5
2010	<0.1	3.1	28.8	179	17.5
2019	<0.1	2.7	26.4	168	20.0
Rate of change in death rate (% per year)					
2000-2010	-4.2%	-1.4%	-2.3%	+0.6%	+1.9%
2010-2019	.	-1.2%	-1.0%	-0.7%	+1.5%
2000-2019	-4.1%	-1.3%	-1.7%	0.0%	+1.7%

Data source: Global Health Estimates 2021

Panel C5: Diabetes - India

	Age group				
	0-14	15-49	50-69	70+	All ages
Population size (millions)					
2000	376	543	111	27.6	1,060
2010	390	659	157	37.8	1,240
2019	373	757	209	50.5	1,390
Rate of change in population size (% per year)					
2000-2010	+0.4%	+1.9%	+3.6%	+3.2%	+1.6%
2010-2019	-0.5%	+1.6%	+3.2%	+3.3%	+1.2%
2000-2019	0.0%	+1.8%	+3.4%	+3.2%	+1.4%
Deaths (thousands)					
2000	1.3	16.6	65.6	58.8	142
2010	0.9	23.6	100	103	228
2019	0.6	30.8	178	178	388
Rate of change in deaths (% per year)					
2000-2010	-3.1%	+3.6%	+4.3%	+5.8%	+4.8%
2010-2019	-5.5%	+3.0%	+6.6%	+6.2%	+6.1%
2000-2019	-4.3%	+3.3%	+5.4%	+6.0%	+5.4%
Death rate (per 100 000 population per year)					
2000	0.3	3.1	59.2	213	13.4
2010	0.2	3.6	63.6	273	18.3
2019	0.1	4.1	85.4	352	27.9
Rate of change in death rate (% per year)					
2000-2010	-3.5%	+1.6%	+0.7%	+2.5%	+3.1%
2010-2019	-5.1%	+1.5%	+3.3%	+2.9%	+4.8%
2000-2019	-4.2%	+1.5%	+1.9%	+2.7%	+3.9%

Data source: Global Health Estimates 2021

Panel C6: Diabetes - Latin America and Caribbean

	Age group				
	0-14	15-49	50-69	70+	All ages
Population size (millions)					
2000	166	272	59.4	17.7	515
2010	161	312	83.7	25.4	583
2019	155	337	110	34.6	636
Rate of change in population size (% per year)					
2000-2010	-0.3%	+1.4%	+3.5%	+3.7%	+1.2%
2010-2019	-0.5%	+0.8%	+3.1%	+3.5%	+1.0%
2000-2019	-0.4%	+1.1%	+3.3%	+3.6%	+1.1%
Deaths (thousands)					
2000	0.5	13.2	58.6	68.1	140
2010	0.3	16.7	82.2	111	210
2019	0.3	20.3	103	140	264
Rate of change in deaths (% per year)					
2000-2010	-3.7%	+2.4%	+3.4%	+5.0%	+4.1%
2010-2019	-1.0%	+2.2%	+2.6%	+2.6%	+2.6%
2000-2019	-2.4%	+2.3%	+3.0%	+3.9%	+3.4%
Death rate (per 100 000 population per year)					
2000	0.3	4.9	98.6	385	27.2
2010	0.2	5.4	98.1	438	36.1
2019	0.2	6.0	93.6	405	41.5
Rate of change in death rate (% per year)					
2000-2010	-3.4%	+1.0%	0.0%	+1.3%	+2.8%
2010-2019	-0.5%	+1.3%	-0.5%	-0.8%	+1.6%
2000-2019	-2.0%	+1.1%	-0.3%	+0.3%	+2.2%

Data source: Global Health Estimates 2021

Panel C7: Diabetes - Middle East and North Africa

	Age group				
	0-14	15-49	50-69	70+	All ages
Population size (millions)					
2000	135	200	36.9	9.6	381
2010	140	259	53.6	13.8	467
2019	163	296	76.1	18.3	553
Rate of change in population size (% per year)					
2000-2010	+0.4%	+2.6%	+3.8%	+3.7%	+2.0%
2010-2019	+1.7%	+1.5%	+4.0%	+3.2%	+1.9%
2000-2019	+1.0%	+2.1%	+3.9%	+3.5%	+2.0%
Deaths (thousands)					
2000	0.7	4.7	20.8	24.7	50.9
2010	0.5	6.2	32.0	44.4	83.1
2019	0.4	7.5	46.7	64.4	119
Rate of change in deaths (% per year)					
2000-2010	-2.9%	+2.9%	+4.4%	+6.0%	+5.0%
2010-2019	-2.2%	+2.1%	+4.3%	+4.2%	+4.1%
2000-2019	-2.6%	+2.5%	+4.3%	+5.2%	+4.6%
Death rate (per 100 000 population per year)					
2000	0.5	2.3	56.5	257	13.4
2010	0.4	2.4	59.6	322	17.8
2019	0.3	2.5	61.3	351	21.5
Rate of change in death rate (% per year)					
2000-2010	-3.3%	+0.3%	+0.5%	+2.2%	+2.9%
2010-2019	-3.9%	+0.6%	+0.3%	+1.0%	+2.1%
2000-2019	-3.6%	+0.4%	+0.4%	+1.6%	+2.5%

Data source: Global Health Estimates 2021

Panel C8: Diabetes - North Atlantic

	Age group				
	0-14	15-49	50-69	70+	All ages
Population size (millions)					
2000	72.0	209	93.2	47.7	422
2010	71.3	211	107	57.5	446
2019	72.1	202	122	67.8	464
Rate of change in population size (% per year)					
2000-2010	-0.1%	+0.1%	+1.4%	+1.9%	+0.6%
2010-2019	+0.1%	-0.4%	+1.5%	+1.9%	+0.4%
2000-2019	0.0%	-0.2%	+1.4%	+1.9%	+0.5%
Deaths (thousands)					
2000	<0.1	2.4	16.5	74.0	92.8
2010	<0.1	2.2	14.5	84.9	102
2019	<0.1	1.8	14.9	89.9	107
Rate of change in deaths (% per year)					
2000-2010	.	-0.8%	-1.3%	+1.4%	+0.9%
2010-2019	.	-1.8%	+0.3%	+0.6%	+0.5%
2000-2019	.	-1.3%	-0.5%	+1.0%	+0.7%
Death rate (per 100 000 population per year)					
2000	<0.1	1.1	17.7	155	22.0
2010	<0.1	1.0	13.6	148	22.8
2019	<0.1	0.9	12.2	133	23.0
Rate of change in death rate (% per year)					
2000-2010	.	-0.9%	-2.6%	-0.5%	+0.3%
2010-2019	.	-1.4%	-1.2%	-1.2%	+0.1%
2000-2019	.	-1.1%	-1.9%	-0.8%	+0.2%

Data source: Global Health Estimates 2021

Panel C9: Diabetes - Sub-Saharan Africa

	Age group				
	0-14	15-49	50-69	70+	All ages
Population size (millions)					
2000	305	313	52.5	11.8	682
2010	393	418	69.0	15.6	895
2019	482	544	93.4	20.4	1,140
Rate of change in population size (% per year)					
2000-2010	+2.6%	+2.9%	+2.8%	+2.8%	+2.8%
2010-2019	+2.3%	+3.0%	+3.4%	+3.0%	+2.7%
2000-2019	+2.4%	+3.0%	+3.1%	+2.9%	+2.7%
Deaths (thousands)					
2000	2.8	14.5	54.7	49.8	122
2010	3.0	21.6	74.2	72.8	172
2019	3.3	28.0	96.2	98.3	226
Rate of change in deaths (% per year)					
2000-2010	+0.7%	+4.0%	+3.1%	+3.9%	+3.5%
2010-2019	+1.0%	+2.9%	+2.9%	+3.4%	+3.1%
2000-2019	+0.9%	+3.5%	+3.0%	+3.6%	+3.3%
Death rate (per 100 000 population per year)					
2000	0.9	4.6	104	422	17.9
2010	0.8	5.2	108	467	19.2
2019	0.7	5.1	103	483	19.8
Rate of change in death rate (% per year)					
2000-2010	-1.8%	+1.1%	+0.3%	+1.0%	+0.7%
2010-2019	-1.2%	-0.1%	-0.5%	+0.4%	+0.4%
2000-2019	-1.6%	+0.5%	-0.1%	+0.7%	+0.5%

Data source: Global Health Estimates 2021

Panel C10: Diabetes - United States

	Age group				
	0-14	15-49	50-69	70+	All ages
Population size (millions)					
2000	59.6	146	51.4	24.9	281
2010	61.3	151	71.3	27.5	311
2019	62.9	157	82.2	35.5	338
Rate of change in population size (% per year)					
2000-2010	+0.3%	+0.4%	+3.3%	+1.0%	+1.0%
2010-2019	+0.3%	+0.4%	+1.6%	+2.9%	+0.9%
2000-2019	+0.3%	+0.4%	+2.5%	+1.9%	+1.0%
Deaths (thousands)					
2000	<0.1	4.8	19.3	46.9	71.1
2010	<0.1	4.9	22.7	42.7	70.4
2019	<0.1	6.0	31.2	52.9	90.2
Rate of change in deaths (% per year)					
2000-2010	.	+0.2%	+1.6%	-0.9%	-0.1%
2010-2019	.	+2.3%	+3.6%	+2.4%	+2.8%
2000-2019	.	+1.2%	+2.6%	+0.6%	+1.3%
Death rate (per 100 000 population per year)					
2000	<0.1	3.3	37.6	189	25.3
2010	<0.1	3.3	31.9	155	22.6
2019	<0.1	3.8	38.0	149	26.7
Rate of change in death rate (% per year)					
2000-2010	.	-0.2%	-1.6%	-1.9%	-1.1%
2010-2019	.	+1.8%	+2.0%	-0.4%	+1.9%
2000-2019	.	+0.8%	+0.1%	-1.2%	+0.3%

Data source: Global Health Estimates 2021

Panel C11: Diabetes - Western Pacific and Southeast Asia

	Age group				
	0-14	15-49	50-69	70+	All ages
Population size (millions)					
2000	276	492	127	38.9	934
2010	275	549	163	56.0	1,040
2019	272	582	205	74.4	1,130
Rate of change in population size (% per year)					
2000-2010	0.0%	+1.1%	+2.5%	+3.7%	+1.1%
2010-2019	-0.1%	+0.6%	+2.6%	+3.2%	+0.9%
2000-2019	-0.1%	+0.9%	+2.5%	+3.5%	+1.0%
Deaths (thousands)					
2000	0.6	23.2	73.3	79.1	176
2010	0.6	27.3	96.4	122	246
2019	0.6	32.2	136	170	339
Rate of change in deaths (% per year)					
2000-2010	-1.2%	+1.6%	+2.8%	+4.4%	+3.4%
2010-2019	+0.8%	+1.8%	+3.9%	+3.8%	+3.6%
2000-2019	-0.2%	+1.7%	+3.3%	+4.1%	+3.5%
Death rate (per 100 000 population per year)					
2000	0.2	4.7	57.6	203	18.9
2010	0.2	5.0	59.3	218	23.6
2019	0.2	5.5	66.3	229	29.9
Rate of change in death rate (% per year)					
2000-2010	-1.1%	+0.5%	+0.3%	+0.7%	+2.3%
2010-2019	+0.9%	+1.2%	+1.3%	+0.6%	+2.7%
2000-2019	-0.2%	+0.8%	+0.8%	+0.6%	+2.5%

Data source: Global Health Estimates 2021

Panel D1: Hemorrhagic stroke - World

	Age group				
	0-14	15-49	50-69	70+	All ages
Population size (millions)					
2000	1,870	3,210	808	269	6,160
2010	1,910	3,670	1,080	355	7,010
2019	2,030	3,930	1,380	451	7,800
Rate of change in population size (% per year)					
2000-2010	+0.2%	+1.3%	+2.9%	+2.8%	+1.3%
2010-2019	+0.7%	+0.8%	+2.9%	+2.7%	+1.2%
2000-2019	+0.4%	+1.1%	+2.9%	+2.7%	+1.2%
Deaths (thousands)					
2000	30.2	324	1,170	1,540	3,060
2010	20.3	337	1,200	1,770	3,330
2019	15.9	309	1,350	1,810	3,490
Rate of change in deaths (% per year)					
2000-2010	-3.9%	+0.4%	+0.2%	+1.4%	+0.8%
2010-2019	-2.7%	-0.9%	+1.3%	+0.3%	+0.5%
2000-2019	-3.3%	-0.2%	+0.7%	+0.9%	+0.7%
Death rate (per 100 000 population per year)					
2000	1.6	10.1	145	570	49.8
2010	1.1	9.2	112	497	47.5
2019	0.8	7.9	97.2	402	44.7
Rate of change in death rate (% per year)					
2000-2010	-4.1%	-0.9%	-2.6%	-1.4%	-0.5%
2010-2019	-3.4%	-1.7%	-1.5%	-2.3%	-0.7%
2000-2019	-3.7%	-1.3%	-2.1%	-1.8%	-0.6%

Data source: Global Health Estimates 2021

Panel D2: Hemorrhagic stroke - Central and Eastern Europe

	Age group				
	0-14	15-49	50-69	70+	All ages
Population size (millions)					
2000	63.2	181	70.2	29.2	343
2010	50.2	169	78.6	34.2	332
2019	54.6	153	85.8	35.2	329
Rate of change in population size (% per year)					
2000-2010	-2.3%	-0.7%	+1.1%	+1.6%	-0.3%
2010-2019	+0.9%	-1.1%	+1.0%	+0.3%	-0.1%
2000-2019	-0.8%	-0.9%	+1.1%	+1.0%	-0.2%
Deaths (thousands)					
2000	0.2	25.2	104	115	244
2010	0.1	16.8	67.9	94.1	179
2019	<0.1	13.1	56.0	81.7	151
Rate of change in deaths (% per year)					
2000-2010	-6.9%	-4.0%	-4.2%	-1.9%	-3.1%
2010-2019	-6.3%	-2.8%	-2.1%	-1.6%	-1.9%
2000-2019	-6.6%	-3.4%	-3.2%	-1.8%	-2.5%
Death rate (per 100 000 population per year)					
2000	0.4	14.0	148	392	71.1
2010	0.2	10.0	86.3	275	53.9
2019	0.1	8.5	65.2	232	45.8
Rate of change in death rate (% per year)					
2000-2010	-4.7%	-3.3%	-5.3%	-3.5%	-2.7%
2010-2019	-7.2%	-1.7%	-3.1%	-1.9%	-1.8%
2000-2019	-5.9%	-2.6%	-4.2%	-2.7%	-2.3%

Data source: Global Health Estimates 2021

Panel D3: Hemorrhagic stroke - Central Asia

	Age group				
	0-14	15-49	50-69	70+	All ages
Population size (millions)					
2000	100	115	21.7	5.4	242
2010	114	153	29.3	7.3	303
2019	131	177	39.7	8.8	357
Rate of change in population size (% per year)					
2000-2010	+1.3%	+2.9%	+3.0%	+3.1%	+2.3%
2010-2019	+1.6%	+1.6%	+3.4%	+2.1%	+1.8%
2000-2019	+1.4%	+2.3%	+3.2%	+2.6%	+2.1%
Deaths (thousands)					
2000	1.8	14.1	50.6	37.0	104
2010	1.5	15.1	50.8	45.2	113
2019	1.4	14.8	53.1	41.5	111
Rate of change in deaths (% per year)					
2000-2010	-1.9%	+0.6%	0.0%	+2.0%	+0.8%
2010-2019	-0.4%	-0.2%	+0.5%	-0.9%	-0.2%
2000-2019	-1.2%	+0.2%	+0.3%	+0.6%	+0.4%
Death rate (per 100 000 population per year)					
2000	1.8	12.3	233	684	42.8
2010	1.3	9.8	173	617	37.1
2019	1.1	8.3	134	470	31.1
Rate of change in death rate (% per year)					
2000-2010	-3.2%	-2.2%	-2.9%	-1.0%	-1.4%
2010-2019	-2.0%	-1.8%	-2.8%	-3.0%	-2.0%
2000-2019	-2.6%	-2.0%	-2.9%	-2.0%	-1.7%

Data source: Global Health Estimates 2021

Panel D4: Hemorrhagic stroke - China

	Age group				
	0-14	15-49	50-69	70+	All ages
Population size (millions)					
2000	312	723	180	54.9	1,270
2010	250	767	257	77.6	1,350
2019	259	708	354	103	1,420
Rate of change in population size (% per year)					
2000-2010	-2.2%	+0.6%	+3.6%	+3.5%	+0.6%
2010-2019	+0.4%	-0.9%	+3.6%	+3.1%	+0.6%
2000-2019	-1.0%	-0.1%	+3.6%	+3.3%	+0.6%
Deaths (thousands)					
2000	3.1	101	415	788	1,310
2010	1.5	99.9	395	902	1,400
2019	0.7	72.9	400	863	1,340
Rate of change in deaths (% per year)					
2000-2010	-6.9%	-0.1%	-0.5%	+1.4%	+0.7%
2010-2019	-8.8%	-3.4%	+0.1%	-0.5%	-0.5%
2000-2019	-7.8%	-1.7%	-0.2%	+0.5%	+0.1%
Death rate (per 100 000 population per year)					
2000	1.0	14.0	230	1,440	103
2010	0.6	13.0	154	1,160	103
2019	0.3	10.3	113	841	93.9
Rate of change in death rate (% per year)					
2000-2010	-4.8%	-0.7%	-4.0%	-2.1%	0.0%
2010-2019	-9.2%	-2.6%	-3.3%	-3.5%	-1.1%
2000-2019	-6.9%	-1.6%	-3.7%	-2.8%	-0.5%

Data source: Global Health Estimates 2021

Panel D5: Hemorrhagic stroke - India

	Age group				
	0-14	15-49	50-69	70+	All ages
Population size (millions)					
2000	376	543	111	27.6	1,060
2010	390	659	157	37.8	1,240
2019	373	757	209	50.5	1,390
Rate of change in population size (% per year)					
2000-2010	+0.4%	+1.9%	+3.6%	+3.2%	+1.6%
2010-2019	-0.5%	+1.6%	+3.2%	+3.3%	+1.2%
2000-2019	0.0%	+1.8%	+3.4%	+3.2%	+1.4%
Deaths (thousands)					
2000	6.0	35.2	144	87.6	273
2010	3.3	46.1	179	117	346
2019	1.6	48.9	258	155	463
Rate of change in deaths (% per year)					
2000-2010	-5.8%	+2.7%	+2.2%	+3.0%	+2.4%
2010-2019	-7.7%	+0.7%	+4.1%	+3.1%	+3.3%
2000-2019	-6.7%	+1.7%	+3.1%	+3.0%	+2.8%
Death rate (per 100 000 population per year)					
2000	1.6	6.5	130	318	25.8
2010	0.8	7.0	114	310	27.8
2019	0.4	6.4	124	307	33.3
Rate of change in death rate (% per year)					
2000-2010	-6.2%	+0.8%	-1.3%	-0.2%	+0.8%
2010-2019	-7.2%	-0.9%	+0.9%	-0.1%	+2.0%
2000-2019	-6.7%	0.0%	-0.3%	-0.2%	+1.4%

Data source: Global Health Estimates 2021

Panel D6: Hemorrhagic stroke - Latin America and Caribbean

	Age group				
	0-14	15-49	50-69	70+	All ages
Population size (millions)					
2000	166	272	59.4	17.7	515
2010	161	312	83.7	25.4	583
2019	155	337	110	34.6	636
Rate of change in population size (% per year)					
2000-2010	-0.3%	+1.4%	+3.5%	+3.7%	+1.2%
2010-2019	-0.5%	+0.8%	+3.1%	+3.5%	+1.0%
2000-2019	-0.4%	+1.1%	+3.3%	+3.6%	+1.1%
Deaths (thousands)					
2000	1.5	22.8	52.6	47.0	124
2010	1.2	19.3	52.9	55.5	129
2019	0.9	17.9	56.5	62.7	138
Rate of change in deaths (% per year)					
2000-2010	-2.4%	-1.7%	+0.1%	+1.7%	+0.4%
2010-2019	-3.3%	-0.8%	+0.7%	+1.4%	+0.8%
2000-2019	-2.8%	-1.3%	+0.4%	+1.5%	+0.6%
Death rate (per 100 000 population per year)					
2000	0.9	8.4	88.6	266	24.1
2010	0.7	6.2	63.2	219	22.1
2019	0.6	5.3	51.2	181	21.7
Rate of change in death rate (% per year)					
2000-2010	-2.2%	-3.0%	-3.3%	-1.9%	-0.8%
2010-2019	-2.8%	-1.7%	-2.3%	-2.1%	-0.2%
2000-2019	-2.5%	-2.4%	-2.9%	-2.0%	-0.6%

Data source: Global Health Estimates 2021

Panel D7: Hemorrhagic stroke - Middle East and North Africa

	Age group				
	0-14	15-49	50-69	70+	All ages
Population size (millions)					
2000	135	200	36.9	9.6	381
2010	140	259	53.6	13.8	467
2019	163	296	76.1	18.3	553
Rate of change in population size (% per year)					
2000-2010	+0.4%	+2.6%	+3.8%	+3.7%	+2.0%
2010-2019	+1.7%	+1.5%	+4.0%	+3.2%	+1.9%
2000-2019	+1.0%	+2.1%	+3.9%	+3.5%	+2.0%
Deaths (thousands)					
2000	4.7	12.9	29.1	28.8	75.6
2010	2.1	11.7	27.6	32.4	73.9
2019	1.4	11.6	32.7	39.1	84.9
Rate of change in deaths (% per year)					
2000-2010	-7.6%	-1.0%	-0.6%	+1.2%	-0.2%
2010-2019	-4.3%	-0.1%	+1.9%	+2.1%	+1.6%
2000-2019	-6.1%	-0.6%	+0.6%	+1.6%	+0.6%
Death rate (per 100 000 population per year)					
2000	3.5	6.5	79.0	300	19.8
2010	1.5	4.5	51.5	235	15.8
2019	0.9	3.9	43.0	213	15.3
Rate of change in death rate (% per year)					
2000-2010	-7.9%	-3.5%	-4.2%	-2.4%	-2.2%
2010-2019	-5.9%	-1.6%	-2.0%	-1.1%	-0.3%
2000-2019	-7.0%	-2.6%	-3.2%	-1.8%	-1.3%

Data source: Global Health Estimates 2021

Panel D8: Hemorrhagic stroke - North Atlantic

	Age group				
	0-14	15-49	50-69	70+	All ages
Population size (millions)					
2000	72.0	209	93.2	47.7	422
2010	71.3	211	107	57.5	446
2019	72.1	202	122	67.8	464
Rate of change in population size (% per year)					
2000-2010	-0.1%	+0.1%	+1.4%	+1.9%	+0.6%
2010-2019	+0.1%	-0.4%	+1.5%	+1.9%	+0.4%
2000-2019	0.0%	-0.2%	+1.4%	+1.9%	+0.5%
Deaths (thousands)					
2000	0.2	7.0	29.7	89.0	126
2010	0.1	5.2	21.9	88.6	116
2019	<0.1	3.8	20.2	83.1	107
Rate of change in deaths (% per year)					
2000-2010	-4.3%	-3.0%	-3.0%	0.0%	-0.8%
2010-2019	-3.3%	-3.2%	-0.9%	-0.7%	-0.8%
2000-2019	-3.8%	-3.1%	-2.0%	-0.4%	-0.8%
Death rate (per 100 000 population per year)					
2000	0.2	3.4	31.8	187	29.8
2010	0.1	2.4	20.6	154	26.0
2019	0.1	1.9	16.6	123	23.1
Rate of change in death rate (% per year)					
2000-2010	-4.3%	-3.1%	-4.3%	-1.9%	-1.4%
2010-2019	-3.4%	-2.8%	-2.4%	-2.5%	-1.3%
2000-2019	-3.9%	-3.0%	-3.4%	-2.2%	-1.3%

Data source: Global Health Estimates 2021

Panel D9: Hemorrhagic stroke - Sub-Saharan Africa

	Age group				
	0-14	15-49	50-69	70+	All ages
Population size (millions)					
2000	305	313	52.5	11.8	682
2010	393	418	69.0	15.6	895
2019	482	544	93.4	20.4	1,140
Rate of change in population size (% per year)					
2000-2010	+2.6%	+2.9%	+2.8%	+2.8%	+2.8%
2010-2019	+2.3%	+3.0%	+3.4%	+3.0%	+2.7%
2000-2019	+2.4%	+3.0%	+3.1%	+2.9%	+2.7%
Deaths (thousands)					
2000	8.0	30.0	106	78.9	223
2010	7.2	37.5	120	92.8	258
2019	6.4	44.0	141	111	302
Rate of change in deaths (% per year)					
2000-2010	-1.1%	+2.3%	+1.3%	+1.6%	+1.5%
2010-2019	-1.3%	+1.8%	+1.8%	+2.0%	+1.8%
2000-2019	-1.2%	+2.0%	+1.5%	+1.8%	+1.6%
Death rate (per 100 000 population per year)					
2000	2.6	9.6	201	668	32.6
2010	1.8	9.0	174	596	28.8
2019	1.3	8.1	151	543	26.5
Rate of change in death rate (% per year)					
2000-2010	-3.6%	-0.6%	-1.4%	-1.1%	-1.2%
2010-2019	-3.5%	-1.2%	-1.6%	-1.0%	-0.9%
2000-2019	-3.6%	-0.9%	-1.5%	-1.1%	-1.1%

Data source: Global Health Estimates 2021

Panel D10: Hemorrhagic stroke - United States

	Age group				
	0-14	15-49	50-69	70+	All ages
Population size (millions)					
2000	59.6	146	51.4	24.9	281
2010	61.3	151	71.3	27.5	311
2019	62.9	157	82.2	35.5	338
Rate of change in population size (% per year)					
2000-2010	+0.3%	+0.4%	+3.3%	+1.0%	+1.0%
2010-2019	+0.3%	+0.4%	+1.6%	+2.9%	+0.9%
2000-2019	+0.3%	+0.4%	+2.5%	+1.9%	+1.0%
Deaths (thousands)					
2000	0.2	5.8	17.1	40.1	63.2
2010	0.2	4.8	18.7	36.3	60.0
2019	0.2	4.3	20.9	39.8	65.2
Rate of change in deaths (% per year)					
2000-2010	+0.7%	-2.0%	+0.9%	-1.0%	-0.5%
2010-2019	-1.1%	-1.2%	+1.3%	+1.0%	+0.9%
2000-2019	-0.2%	-1.6%	+1.1%	0.0%	+0.2%
Death rate (per 100 000 population per year)					
2000	0.3	4.0	33.3	161	22.4
2010	0.3	3.2	26.2	132	19.3
2019	0.3	2.7	25.5	112	19.3
Rate of change in death rate (% per year)					
2000-2010	+0.4%	-2.3%	-2.4%	-2.0%	-1.5%
2010-2019	-1.4%	-1.6%	-0.3%	-1.8%	0.0%
2000-2019	-0.5%	-2.0%	-1.4%	-1.9%	-0.8%

Data source: Global Health Estimates 2021

Panel D11: Hemorrhagic stroke - Western Pacific and Southeast Asia

	Age group				
	0-14	15-49	50-69	70+	All ages
Population size (millions)					
2000	276	492	127	38.9	934
2010	275	549	163	56.0	1,040
2019	272	582	205	74.4	1,130
Rate of change in population size (% per year)					
2000-2010	0.0%	+1.1%	+2.5%	+3.7%	+1.1%
2010-2019	-0.1%	+0.6%	+2.6%	+3.2%	+0.9%
2000-2019	-0.1%	+0.9%	+2.5%	+3.5%	+1.0%
Deaths (thousands)					
2000	4.5	68.2	222	220	515
2010	3.2	79.3	266	297	645
2019	3.1	77.0	305	333	719
Rate of change in deaths (% per year)					
2000-2010	-3.4%	+1.5%	+1.8%	+3.0%	+2.3%
2010-2019	-0.1%	-0.3%	+1.5%	+1.3%	+1.2%
2000-2019	-1.8%	+0.6%	+1.7%	+2.2%	+1.8%
Death rate (per 100 000 population per year)					
2000	1.6	13.9	175	565	55.2
2010	1.1	14.4	163	531	61.9
2019	1.2	13.2	149	448	63.4
Rate of change in death rate (% per year)					
2000-2010	-3.4%	+0.4%	-0.7%	-0.6%	+1.2%
2010-2019	+0.1%	-1.0%	-1.0%	-1.9%	+0.3%
2000-2019	-1.8%	-0.2%	-0.8%	-1.2%	+0.7%

Data source: Global Health Estimates 2021

Panel E1: Infection-associated NCDs - World

	Age group				
	0-14	15-49	50-69	70+	All ages
Population size (millions)					
2000	1,870	3,210	808	269	6,160
2010	1,910	3,670	1,080	355	7,010
2019	2,030	3,930	1,380	451	7,800
Rate of change in population size (% per year)					
2000-2010	+0.2%	+1.3%	+2.9%	+2.8%	+1.3%
2010-2019	+0.7%	+0.8%	+2.9%	+2.7%	+1.2%
2000-2019	+0.4%	+1.1%	+2.9%	+2.7%	+1.2%
Deaths (thousands)					
2000	24.5	534	976	778	2,310
2010	14.3	528	1,010	905	2,460
2019	10.4	487	1,110	965	2,580
Rate of change in deaths (% per year)					
2000-2010	-5.2%	-0.1%	+0.3%	+1.5%	+0.6%
2010-2019	-3.5%	-0.9%	+1.1%	+0.7%	+0.5%
2000-2019	-4.4%	-0.5%	+0.7%	+1.1%	+0.6%
Death rate (per 100 000 population per year)					
2000	1.3	16.6	121	289	37.5
2010	0.8	14.4	94.0	255	35.1
2019	0.5	12.4	80.4	214	33.0
Rate of change in death rate (% per year)					
2000-2010	-5.4%	-1.4%	-2.5%	-1.2%	-0.7%
2010-2019	-4.1%	-1.7%	-1.7%	-1.9%	-0.7%
2000-2019	-4.8%	-1.5%	-2.1%	-1.6%	-0.7%

Data source: Global Health Estimates 2021

Panel E2: Infection-associated NCDs - Central and Eastern Europe

	Age group				
	0-14	15-49	50-69	70+	All ages
Population size (millions)					
2000	63.2	181	70.2	29.2	343
2010	50.2	169	78.6	34.2	332
2019	54.6	153	85.8	35.2	329
Rate of change in population size (% per year)					
2000-2010	-2.3%	-0.7%	+1.1%	+1.6%	-0.3%
2010-2019	+0.9%	-1.1%	+1.0%	+0.3%	-0.1%
2000-2019	-0.8%	-0.9%	+1.1%	+1.0%	-0.2%
Deaths (thousands)					
2000	<0.1	30.0	77.6	56.5	164
2010	<0.1	25.4	65.6	54.1	145
2019	<0.1	20.7	59.2	45.3	125
Rate of change in deaths (% per year)					
2000-2010	.	-1.7%	-1.7%	-0.4%	-1.2%
2010-2019	.	-2.3%	-1.1%	-2.0%	-1.6%
2000-2019	.	-1.9%	-1.4%	-1.2%	-1.4%
Death rate (per 100 000 population per year)					
2000	0.1	16.6	111	194	47.8
2010	<0.1	15.1	83.4	158	43.8
2019	<0.1	13.5	69.0	129	38.1
Rate of change in death rate (% per year)					
2000-2010	-6.0%	-1.0%	-2.8%	-2.0%	-0.9%
2010-2019	.	-1.2%	-2.1%	-2.3%	-1.5%
2000-2019	-7.1%	-1.1%	-2.4%	-2.1%	-1.2%

Data source: Global Health Estimates 2021

Panel E3: Infection-associated NCDs - Central Asia

	Age group				
	0-14	15-49	50-69	70+	All ages
Population size (millions)					
2000	100	115	21.7	5.4	242
2010	114	153	29.3	7.3	303
2019	131	177	39.7	8.8	357
Rate of change in population size (% per year)					
2000-2010	+1.3%	+2.9%	+3.0%	+3.1%	+2.3%
2010-2019	+1.6%	+1.6%	+3.4%	+2.1%	+1.8%
2000-2019	+1.4%	+2.3%	+3.2%	+2.6%	+2.1%
Deaths (thousands)					
2000	2.3	33.9	42.7	22.5	101
2010	1.6	37.9	45.8	27.4	113
2019	2.0	36.4	49.6	27.9	116
Rate of change in deaths (% per year)					
2000-2010	-3.5%	+1.1%	+0.7%	+2.0%	+1.1%
2010-2019	+2.3%	-0.4%	+0.9%	+0.2%	+0.3%
2000-2019	-0.8%	+0.4%	+0.8%	+1.1%	+0.7%
Death rate (per 100 000 population per year)					
2000	2.3	29.5	196	416	41.9
2010	1.4	24.8	156	374	37.1
2019	1.5	20.5	125	316	32.5
Rate of change in death rate (% per year)					
2000-2010	-4.7%	-1.8%	-2.3%	-1.0%	-1.2%
2010-2019	+0.7%	-2.1%	-2.4%	-1.9%	-1.5%
2000-2019	-2.2%	-1.9%	-2.4%	-1.4%	-1.3%

Data source: Global Health Estimates 2021

Panel E4: Infection-associated NCDs - China

	Age group				
	0-14	15-49	50-69	70+	All ages
Population size (millions)					
2000	312	723	180	54.9	1,270
2010	250	767	257	77.6	1,350
2019	259	708	354	103	1,420
Rate of change in population size (% per year)					
2000-2010	-2.2%	+0.6%	+3.6%	+3.5%	+0.6%
2010-2019	+0.4%	-0.9%	+3.6%	+3.1%	+0.6%
2000-2019	-1.0%	-0.1%	+3.6%	+3.3%	+0.6%
Deaths (thousands)					
2000	0.9	153	327	295	775
2010	0.3	127	313	342	782
2019	0.2	98.0	334	346	779
Rate of change in deaths (% per year)					
2000-2010	-9.7%	-1.8%	-0.4%	+1.5%	+0.1%
2010-2019	-5.5%	-2.8%	+0.7%	+0.1%	0.0%
2000-2019	-7.7%	-2.3%	+0.1%	+0.8%	0.0%
Death rate (per 100 000 population per year)					
2000	0.3	21.1	182	536	61.1
2010	0.1	16.5	122	440	57.8
2019	<0.1	13.8	94.6	337	54.7
Rate of change in death rate (% per year)					
2000-2010	-7.6%	-2.4%	-3.9%	-2.0%	-0.5%
2010-2019	-5.9%	-2.0%	-2.8%	-2.9%	-0.6%
2000-2019	-6.8%	-2.2%	-3.4%	-2.4%	-0.6%

Data source: Global Health Estimates 2021

Panel E5: Infection-associated NCDs - India

	Age group				
	0-14	15-49	50-69	70+	All ages
Population size (millions)					
2000	376	543	111	27.6	1,060
2010	390	659	157	37.8	1,240
2019	373	757	209	50.5	1,390
Rate of change in population size (% per year)					
2000-2010	+0.4%	+1.9%	+3.6%	+3.2%	+1.6%
2010-2019	-0.5%	+1.6%	+3.2%	+3.3%	+1.2%
2000-2019	0.0%	+1.8%	+3.4%	+3.2%	+1.4%
Deaths (thousands)					
2000	12.8	101	126	62.9	303
2010	6.0	117	149	89.6	362
2019	2.8	107	188	110	408
Rate of change in deaths (% per year)					
2000-2010	-7.2%	+1.5%	+1.7%	+3.6%	+1.8%
2010-2019	-8.3%	-1.0%	+2.6%	+2.3%	+1.3%
2000-2019	-7.7%	+0.3%	+2.1%	+3.0%	+1.6%
Death rate (per 100 000 population per year)					
2000	3.4	18.6	114	228	28.6
2010	1.6	17.7	95.1	237	29.1
2019	0.7	14.1	90.2	218	29.3
Rate of change in death rate (% per year)					
2000-2010	-7.6%	-0.5%	-1.8%	+0.4%	+0.2%
2010-2019	-7.8%	-2.5%	-0.6%	-0.9%	+0.1%
2000-2019	-7.7%	-1.5%	-1.2%	-0.2%	+0.1%

Data source: Global Health Estimates 2021

Panel E6: Infection-associated NCDs - Latin America and Caribbean

	Age group				
	0-14	15-49	50-69	70+	All ages
Population size (millions)					
2000	166	272	59.4	17.7	515
2010	161	312	83.7	25.4	583
2019	155	337	110	34.6	636
Rate of change in population size (% per year)					
2000-2010	-0.3%	+1.4%	+3.5%	+3.7%	+1.2%
2010-2019	-0.5%	+0.8%	+3.1%	+3.5%	+1.0%
2000-2019	-0.4%	+1.1%	+3.3%	+3.6%	+1.1%
Deaths (thousands)					
2000	0.4	26.8	42.5	37.2	107
2010	0.2	24.7	46.9	44.3	116
2019	0.2	24.8	54.0	50.6	130
Rate of change in deaths (% per year)					
2000-2010	-5.8%	-0.8%	+1.0%	+1.8%	+0.8%
2010-2019	-2.8%	+0.1%	+1.6%	+1.5%	+1.2%
2000-2019	-4.4%	-0.4%	+1.3%	+1.6%	+1.0%
Death rate (per 100 000 population per year)					
2000	0.2	9.9	71.6	210	20.7
2010	0.1	7.9	56.1	175	19.9
2019	0.1	7.4	49.0	146	20.4
Rate of change in death rate (% per year)					
2000-2010	-5.5%	-2.2%	-2.4%	-1.8%	-0.4%
2010-2019	-2.3%	-0.8%	-1.5%	-2.0%	+0.2%
2000-2019	-4.0%	-1.5%	-2.0%	-1.9%	-0.1%

Data source: Global Health Estimates 2021

Panel E7: Infection-associated NCDs - Middle East and North Africa

	Age group				
	0-14	15-49	50-69	70+	All ages
Population size (millions)					
2000	135	200	36.9	9.6	381
2010	140	259	53.6	13.8	467
2019	163	296	76.1	18.3	553
Rate of change in population size (% per year)					
2000-2010	+0.4%	+2.6%	+3.8%	+3.7%	+2.0%
2010-2019	+1.7%	+1.5%	+4.0%	+3.2%	+1.9%
2000-2019	+1.0%	+2.1%	+3.9%	+3.5%	+2.0%
Deaths (thousands)					
2000	1.6	19.7	44.5	36.4	102
2010	1.0	20.1	61.5	45.2	128
2019	0.7	19.3	71.6	53.7	145
Rate of change in deaths (% per year)					
2000-2010	-4.6%	+0.2%	+3.3%	+2.2%	+2.3%
2010-2019	-3.5%	-0.4%	+1.7%	+1.9%	+1.4%
2000-2019	-4.1%	-0.1%	+2.5%	+2.1%	+1.9%
Death rate (per 100 000 population per year)					
2000	1.2	9.8	121	379	26.8
2010	0.7	7.8	115	327	27.4
2019	0.4	6.5	94.0	293	26.3
Rate of change in death rate (% per year)					
2000-2010	-5.0%	-2.3%	-0.5%	-1.5%	+0.2%
2010-2019	-5.1%	-1.9%	-2.2%	-1.2%	-0.5%
2000-2019	-5.0%	-2.1%	-1.3%	-1.4%	-0.1%

Data source: Global Health Estimates 2021

Panel E8: Infection-associated NCDs - North Atlantic

	Age group				
	0-14	15-49	50-69	70+	All ages
Population size (millions)					
2000	72.0	209	93.2	47.7	422
2010	71.3	211	107	57.5	446
2019	72.1	202	122	67.8	464
Rate of change in population size (% per year)					
2000-2010	-0.1%	+0.1%	+1.4%	+1.9%	+0.6%
2010-2019	+0.1%	-0.4%	+1.5%	+1.9%	+0.4%
2000-2019	0.0%	-0.2%	+1.4%	+1.9%	+0.5%
Deaths (thousands)					
2000	<0.1	10.8	40.2	74.6	126
2010	<0.1	8.8	34.1	70.5	113
2019	<0.1	6.7	33.6	71.1	111
Rate of change in deaths (% per year)					
2000-2010	.	-2.0%	-1.6%	-0.6%	-1.0%
2010-2019	.	-3.0%	-0.2%	+0.1%	-0.2%
2000-2019	.	-2.5%	-0.9%	-0.2%	-0.6%
Death rate (per 100 000 population per year)					
2000	<0.1	5.2	43.1	156	29.8
2010	<0.1	4.2	32.0	123	25.4
2019	<0.1	3.3	27.6	105	24.0
Rate of change in death rate (% per year)					
2000-2010	.	-2.1%	-2.9%	-2.4%	-1.6%
2010-2019	.	-2.6%	-1.6%	-1.7%	-0.6%
2000-2019	.	-2.3%	-2.3%	-2.1%	-1.1%

Data source: Global Health Estimates 2021

Panel E9: Infection-associated NCDs - Sub-Saharan Africa

	Age group				
	0-14	15-49	50-69	70+	All ages
Population size (millions)					
2000	305	313	52.5	11.8	682
2010	393	418	69.0	15.6	895
2019	482	544	93.4	20.4	1,140
Rate of change in population size (% per year)					
2000-2010	+2.6%	+2.9%	+2.8%	+2.8%	+2.8%
2010-2019	+2.3%	+3.0%	+3.4%	+3.0%	+2.7%
2000-2019	+2.4%	+3.0%	+3.1%	+2.9%	+2.7%
Deaths (thousands)					
2000	3.4	54.4	87.8	40.2	186
2010	3.1	68.5	97.5	47.9	217
2019	3.0	82.0	115	55.7	255
Rate of change in deaths (% per year)					
2000-2010	-1.1%	+2.3%	+1.0%	+1.8%	+1.6%
2010-2019	-0.5%	+2.0%	+1.8%	+1.7%	+1.8%
2000-2019	-0.8%	+2.2%	+1.4%	+1.7%	+1.7%
Death rate (per 100 000 population per year)					
2000	1.1	17.4	167	341	27.3
2010	0.8	16.4	141	308	24.2
2019	0.6	15.1	123	274	22.4
Rate of change in death rate (% per year)					
2000-2010	-3.6%	-0.6%	-1.7%	-1.0%	-1.2%
2010-2019	-2.7%	-0.9%	-1.5%	-1.3%	-0.9%
2000-2019	-3.2%	-0.8%	-1.6%	-1.1%	-1.0%

Data source: Global Health Estimates 2021

Panel E10: Infection-associated NCDs - United States

	Age group				
	0-14	15-49	50-69	70+	All ages
Population size (millions)					
2000	59.6	146	51.4	24.9	281
2010	61.3	151	71.3	27.5	311
2019	62.9	157	82.2	35.5	338
Rate of change in population size (% per year)					
2000-2010	+0.3%	+0.4%	+3.3%	+1.0%	+1.0%
2010-2019	+0.3%	+0.4%	+1.6%	+2.9%	+0.9%
2000-2019	+0.3%	+0.4%	+2.5%	+1.9%	+1.0%
Deaths (thousands)					
2000	<0.1	7.7	15.1	20.7	43.6
2010	<0.1	6.8	21.1	19.4	47.3
2019	<0.1	7.0	24.7	23.6	55.4
Rate of change in deaths (% per year)					
2000-2010	.	-1.3%	+3.4%	-0.7%	+0.8%
2010-2019	.	+0.4%	+1.8%	+2.2%	+1.8%
2000-2019	.	-0.5%	+2.6%	+0.7%	+1.3%
Death rate (per 100 000 population per year)					
2000	<0.1	5.3	29.3	83.4	15.5
2010	<0.1	4.5	29.6	70.3	15.2
2019	<0.1	4.5	30.1	66.5	16.4
Rate of change in death rate (% per year)					
2000-2010	.	-1.6%	+0.1%	-1.7%	-0.2%
2010-2019	.	-0.1%	+0.2%	-0.6%	+0.8%
2000-2019	.	-0.9%	+0.1%	-1.2%	+0.3%

Data source: Global Health Estimates 2021

Panel E11: Infection-associated NCDs - Western Pacific and Southeast Asia

	Age group				
	0-14	15-49	50-69	70+	All ages
Population size (millions)					
2000	276	492	127	38.9	934
2010	275	549	163	56.0	1,040
2019	272	582	205	74.4	1,130
Rate of change in population size (% per year)					
2000-2010	0.0%	+1.1%	+2.5%	+3.7%	+1.1%
2010-2019	-0.1%	+0.6%	+2.6%	+3.2%	+0.9%
2000-2019	-0.1%	+0.9%	+2.5%	+3.5%	+1.0%
Deaths (thousands)					
2000	2.9	93.4	168	127	391
2010	2.0	89.7	170	159	421
2019	1.6	83.8	179	175	439
Rate of change in deaths (% per year)					
2000-2010	-3.6%	-0.4%	+0.1%	+2.3%	+0.7%
2010-2019	-2.4%	-0.8%	+0.5%	+1.1%	+0.5%
2000-2019	-3.0%	-0.6%	+0.3%	+1.7%	+0.6%
Death rate (per 100 000 population per year)					
2000	1.0	19.0	132	326	41.9
2010	0.7	16.3	105	284	40.4
2019	0.6	14.4	87.2	235	38.8
Rate of change in death rate (% per year)					
2000-2010	-3.5%	-1.5%	-2.3%	-1.4%	-0.4%
2010-2019	-2.3%	-1.4%	-2.0%	-2.1%	-0.4%
2000-2019	-3.0%	-1.5%	-2.2%	-1.7%	-0.4%

Data source: Global Health Estimates 2021

Panel F1: Road injury - World

	Age group				
	0-14	15-49	50-69	70+	All ages
Population size (millions)					
2000	1,870	3,210	808	269	6,160
2010	1,910	3,670	1,080	355	7,010
2019	2,030	3,930	1,380	451	7,800
Rate of change in population size (% per year)					
2000-2010	+0.2%	+1.3%	+2.9%	+2.8%	+1.3%
2010-2019	+0.7%	+0.8%	+2.9%	+2.7%	+1.2%
2000-2019	+0.4%	+1.1%	+2.9%	+2.7%	+1.2%
Deaths (thousands)					
2000	173	689	222	99.7	1,180
2010	133	729	272	118	1,250
2019	112	640	312	134	1,200
Rate of change in deaths (% per year)					
2000-2010	-2.6%	+0.6%	+2.1%	+1.7%	+0.6%
2010-2019	-1.8%	-1.4%	+1.5%	+1.4%	-0.5%
2000-2019	-2.2%	-0.4%	+1.8%	+1.5%	+0.1%
Death rate (per 100 000 population per year)					
2000	9.2	21.5	27.5	37.0	19.2
2010	7.0	19.9	25.3	33.1	17.9
2019	5.5	16.3	22.5	29.6	15.4
Rate of change in death rate (% per year)					
2000-2010	-2.8%	-0.8%	-0.8%	-1.1%	-0.7%
2010-2019	-2.5%	-2.2%	-1.3%	-1.2%	-1.7%
2000-2019	-2.7%	-1.5%	-1.0%	-1.2%	-1.2%

Data source: Global Health Estimates 2021

Panel F2: Road injury - Central and Eastern Europe

	Age group				
	0-14	15-49	50-69	70+	All ages
Population size (millions)					
2000	63.2	181	70.2	29.2	343
2010	50.2	169	78.6	34.2	332
2019	54.6	153	85.8	35.2	329
Rate of change in population size (% per year)					
2000-2010	-2.3%	-0.7%	+1.1%	+1.6%	-0.3%
2010-2019	+0.9%	-1.1%	+1.0%	+0.3%	-0.1%
2000-2019	-0.8%	-0.9%	+1.1%	+1.0%	-0.2%
Deaths (thousands)					
2000	3.2	48.2	16.3	6.7	74.6
2010	1.7	35.2	13.5	5.8	56.2
2019	1.1	21.1	10.7	4.5	37.3
Rate of change in deaths (% per year)					
2000-2010	-6.5%	-3.1%	-1.9%	-1.5%	-2.8%
2010-2019	-4.8%	-5.5%	-2.5%	-2.7%	-4.4%
2000-2019	-5.7%	-4.3%	-2.2%	-2.1%	-3.6%
Death rate (per 100 000 population per year)					
2000	5.1	26.7	23.3	23.1	21.7
2010	3.3	20.9	17.2	16.9	16.9
2019	2.0	13.7	12.5	12.8	11.3
Rate of change in death rate (% per year)					
2000-2010	-4.3%	-2.4%	-3.0%	-3.1%	-2.5%
2010-2019	-5.7%	-4.5%	-3.5%	-3.0%	-4.3%
2000-2019	-5.0%	-3.4%	-3.2%	-3.1%	-3.4%

Data source: Global Health Estimates 2021

Panel F3: Road injury - Central Asia

	Age group				
	0-14	15-49	50-69	70+	All ages
Population size (millions)					
2000	100	115	21.7	5.4	242
2010	114	153	29.3	7.3	303
2019	131	177	39.7	8.8	357
Rate of change in population size (% per year)					
2000-2010	+1.3%	+2.9%	+3.0%	+3.1%	+2.3%
2010-2019	+1.6%	+1.6%	+3.4%	+2.1%	+1.8%
2000-2019	+1.4%	+2.3%	+3.2%	+2.6%	+2.1%
Deaths (thousands)					
2000	5.4	20.6	5.5	1.8	33.3
2010	6.2	29.1	7.8	2.7	45.7
2019	6.2	30.2	9.2	2.9	48.5
Rate of change in deaths (% per year)					
2000-2010	+1.5%	+3.5%	+3.5%	+4.2%	+3.2%
2010-2019	0.0%	+0.4%	+1.9%	+0.8%	+0.6%
2000-2019	+0.8%	+2.0%	+2.7%	+2.6%	+2.0%
Death rate (per 100 000 population per year)					
2000	5.4	17.9	25.5	33.1	13.8
2010	5.5	19.0	26.6	36.9	15.1
2019	4.7	17.0	23.2	33.0	13.6
Rate of change in death rate (% per year)					
2000-2010	+0.2%	+0.6%	+0.4%	+1.1%	+0.9%
2010-2019	-1.6%	-1.2%	-1.5%	-1.2%	-1.2%
2000-2019	-0.7%	-0.3%	-0.5%	0.0%	-0.1%

Data source: Global Health Estimates 2021

Panel F4: Road injury - China

	Age group				
	0-14	15-49	50-69	70+	All ages
Population size (millions)					
2000	312	723	180	54.9	1,270
2010	250	767	257	77.6	1,350
2019	259	708	354	103	1,420
Rate of change in population size (% per year)					
2000-2010	-2.2%	+0.6%	+3.6%	+3.5%	+0.6%
2010-2019	+0.4%	-0.9%	+3.6%	+3.1%	+0.6%
2000-2019	-1.0%	-0.1%	+3.6%	+3.3%	+0.6%
Deaths (thousands)					
2000	26.5	175	54.1	23.2	279
2010	12.7	165	71.3	30.4	280
2019	9.9	124	82.2	34.9	251
Rate of change in deaths (% per year)					
2000-2010	-7.1%	-0.6%	+2.8%	+2.7%	0.0%
2010-2019	-2.7%	-3.2%	+1.6%	+1.6%	-1.2%
2000-2019	-5.0%	-1.8%	+2.2%	+2.2%	-0.6%
Death rate (per 100 000 population per year)					
2000	8.5	24.2	30.0	42.3	22.0
2010	5.1	21.6	27.8	39.1	20.7
2019	3.8	17.5	23.2	34.1	17.6
Rate of change in death rate (% per year)					
2000-2010	-5.0%	-1.2%	-0.8%	-0.8%	-0.6%
2010-2019	-3.1%	-2.3%	-2.0%	-1.5%	-1.8%
2000-2019	-4.1%	-1.7%	-1.3%	-1.1%	-1.2%

Data source: Global Health Estimates 2021

Panel F5: Road injury - India

	Age group				
	0-14	15-49	50-69	70+	All ages
Population size (millions)					
2000	376	543	111	27.6	1,060
2010	390	659	157	37.8	1,240
2019	373	757	209	50.5	1,390
Rate of change in population size (% per year)					
2000-2010	+0.4%	+1.9%	+3.6%	+3.2%	+1.6%
2010-2019	-0.5%	+1.6%	+3.2%	+3.3%	+1.2%
2000-2019	0.0%	+1.8%	+3.4%	+3.2%	+1.4%
Deaths (thousands)					
2000	24.3	110	31.6	10.2	177
2010	15.6	137	44.2	14.3	211
2019	9.9	122	59.9	20.0	212
Rate of change in deaths (% per year)					
2000-2010	-4.4%	+2.2%	+3.4%	+3.4%	+1.8%
2010-2019	-4.9%	-1.3%	+3.4%	+3.8%	0.0%
2000-2019	-4.6%	+0.5%	+3.4%	+3.6%	+1.0%
Death rate (per 100 000 population per year)					
2000	6.5	20.3	28.5	37.1	16.7
2010	4.0	20.8	28.1	37.8	17.0
2019	2.7	16.1	28.7	39.7	15.2
Rate of change in death rate (% per year)					
2000-2010	-4.7%	+0.2%	-0.1%	+0.2%	+0.2%
2010-2019	-4.4%	-2.8%	+0.2%	+0.5%	-1.2%
2000-2019	-4.6%	-1.2%	0.0%	+0.3%	-0.5%

Data source: Global Health Estimates 2021

Panel F6: Road injury - Latin America and Caribbean

	Age group				
	0-14	15-49	50-69	70+	All ages
Population size (millions)					
2000	166	272	59.4	17.7	515
2010	161	312	83.7	25.4	583
2019	155	337	110	34.6	636
Rate of change in population size (% per year)					
2000-2010	-0.3%	+1.4%	+3.5%	+3.7%	+1.2%
2010-2019	-0.5%	+0.8%	+3.1%	+3.5%	+1.0%
2000-2019	-0.4%	+1.1%	+3.3%	+3.6%	+1.1%
Deaths (thousands)					
2000	9.8	54.2	14.4	6.1	84.4
2010	7.2	69.6	20.4	8.8	106
2019	4.4	60.1	21.6	9.2	95.3
Rate of change in deaths (% per year)					
2000-2010	-3.1%	+2.5%	+3.6%	+3.7%	+2.3%
2010-2019	-5.2%	-1.6%	+0.6%	+0.6%	-1.2%
2000-2019	-4.1%	+0.6%	+2.2%	+2.2%	+0.6%
Death rate (per 100 000 population per year)					
2000	5.9	19.9	24.2	34.4	16.4
2010	4.4	22.3	24.4	34.5	18.2
2019	2.8	17.8	19.5	26.7	15.0
Rate of change in death rate (% per year)					
2000-2010	-2.8%	+1.1%	+0.1%	0.0%	+1.0%
2010-2019	-4.8%	-2.4%	-2.4%	-2.8%	-2.1%
2000-2019	-3.7%	-0.6%	-1.1%	-1.3%	-0.5%

Data source: Global Health Estimates 2021

Panel F7: Road injury - Middle East and North Africa

	Age group				
	0-14	15-49	50-69	70+	All ages
Population size (millions)					
2000	135	200	36.9	9.6	381
2010	140	259	53.6	13.8	467
2019	163	296	76.1	18.3	553
Rate of change in population size (% per year)					
2000-2010	+0.4%	+2.6%	+3.8%	+3.7%	+2.0%
2010-2019	+1.7%	+1.5%	+4.0%	+3.2%	+1.9%
2000-2019	+1.0%	+2.1%	+3.9%	+3.5%	+2.0%
Deaths (thousands)					
2000	14.7	41.1	11.3	4.1	71.3
2010	14.1	58.2	16.1	6.4	94.8
2019	11.9	50.6	18.7	7.5	88.7
Rate of change in deaths (% per year)					
2000-2010	-0.4%	+3.5%	+3.6%	+4.6%	+2.9%
2010-2019	-1.9%	-1.5%	+1.7%	+1.7%	-0.7%
2000-2019	-1.1%	+1.1%	+2.7%	+3.2%	+1.2%
Death rate (per 100 000 population per year)					
2000	10.9	20.6	30.7	42.8	18.7
2010	10.1	22.4	30.0	46.7	20.3
2019	7.3	17.1	24.6	41.0	16.0
Rate of change in death rate (% per year)					
2000-2010	-0.8%	+0.9%	-0.2%	+0.9%	+0.8%
2010-2019	-3.5%	-3.0%	-2.2%	-1.4%	-2.6%
2000-2019	-2.1%	-1.0%	-1.2%	-0.2%	-0.8%

Data source: Global Health Estimates 2021

Panel F8: Road injury - North Atlantic

	Age group				
	0-14	15-49	50-69	70+	All ages
Population size (millions)					
2000	72.0	209	93.2	47.7	422
2010	71.3	211	107	57.5	446
2019	72.1	202	122	67.8	464
Rate of change in population size (% per year)					
2000-2010	-0.1%	+0.1%	+1.4%	+1.9%	+0.6%
2010-2019	+0.1%	-0.4%	+1.5%	+1.9%	+0.4%
2000-2019	0.0%	-0.2%	+1.4%	+1.9%	+0.5%
Deaths (thousands)					
2000	1.7	29.3	9.1	7.8	48.0
2010	0.7	14.5	5.5	5.2	26.0
2019	0.4	9.2	5.4	5.3	20.3
Rate of change in deaths (% per year)					
2000-2010	-8.5%	-6.8%	-4.9%	-3.9%	-6.0%
2010-2019	-6.9%	-4.9%	-0.1%	+0.1%	-2.7%
2000-2019	-7.8%	-5.9%	-2.7%	-2.0%	-4.4%
Death rate (per 100 000 population per year)					
2000	2.4	14.0	9.8	16.3	11.4
2010	1.0	6.9	5.2	9.1	5.8
2019	0.5	4.5	4.5	7.8	4.4
Rate of change in death rate (% per year)					
2000-2010	-8.4%	-6.9%	-6.2%	-5.7%	-6.5%
2010-2019	-7.0%	-4.5%	-1.6%	-1.8%	-3.1%
2000-2019	-7.8%	-5.8%	-4.0%	-3.8%	-4.9%

Data source: Global Health Estimates 2021

Panel F9: Road injury - Sub-Saharan Africa

	Age group				
	0-14	15-49	50-69	70+	All ages
Population size (millions)					
2000	305	313	52.5	11.8	682
2010	393	418	69.0	15.6	895
2019	482	544	93.4	20.4	1,140
Rate of change in population size (% per year)					
2000-2010	+2.6%	+2.9%	+2.8%	+2.8%	+2.8%
2010-2019	+2.3%	+3.0%	+3.4%	+3.0%	+2.7%
2000-2019	+2.4%	+3.0%	+3.1%	+2.9%	+2.7%
Deaths (thousands)					
2000	61.3	70.7	32.4	13.9	178
2010	54.0	88.4	38.0	17.1	197
2019	54.6	106	46.9	21.7	229
Rate of change in deaths (% per year)					
2000-2010	-1.3%	+2.2%	+1.6%	+2.1%	+1.0%
2010-2019	+0.1%	+2.0%	+2.4%	+2.7%	+1.7%
2000-2019	-0.6%	+2.1%	+2.0%	+2.4%	+1.3%
Death rate (per 100 000 population per year)					
2000	20.1	22.6	61.6	117	26.1
2010	13.7	21.2	55.0	110	22.1
2019	11.3	19.4	50.2	106	20.1
Rate of change in death rate (% per year)					
2000-2010	-3.7%	-0.7%	-1.1%	-0.7%	-1.7%
2010-2019	-2.1%	-0.9%	-1.0%	-0.4%	-1.0%
2000-2019	-3.0%	-0.8%	-1.1%	-0.5%	-1.4%

Data source: Global Health Estimates 2021

Panel F10: Road injury - United States

	Age group				
	0-14	15-49	50-69	70+	All ages
Population size (millions)					
2000	59.6	146	51.4	24.9	281
2010	61.3	151	71.3	27.5	311
2019	62.9	157	82.2	35.5	338
Rate of change in population size (% per year)					
2000-2010	+0.3%	+0.4%	+3.3%	+1.0%	+1.0%
2010-2019	+0.3%	+0.4%	+1.6%	+2.9%	+0.9%
2000-2019	+0.3%	+0.4%	+2.5%	+1.9%	+1.0%
Deaths (thousands)					
2000	2.4	28.3	7.5	6.1	44.3
2010	1.3	21.4	8.9	4.7	36.3
2019	1.2	22.1	11.4	6.2	40.9
Rate of change in deaths (% per year)					
2000-2010	-6.1%	-2.8%	+1.7%	-2.5%	-2.0%
2010-2019	-1.0%	+0.3%	+2.8%	+3.0%	+1.3%
2000-2019	-3.7%	-1.3%	+2.2%	+0.1%	-0.4%
Death rate (per 100 000 population per year)					
2000	4.1	19.4	14.6	24.4	15.7
2010	2.1	14.2	12.5	17.2	11.7
2019	1.9	14.0	13.9	17.4	12.1
Rate of change in death rate (% per year)					
2000-2010	-6.3%	-3.1%	-1.6%	-3.4%	-2.9%
2010-2019	-1.3%	-0.1%	+1.2%	+0.2%	+0.4%
2000-2019	-4.0%	-1.7%	-0.3%	-1.7%	-1.4%

Data source: Global Health Estimates 2021

Panel F11: Road injury - Western Pacific and Southeast Asia

	Age group				
	0-14	15-49	50-69	70+	All ages
Population size (millions)					
2000	276	492	127	38.9	934
2010	275	549	163	56.0	1,040
2019	272	582	205	74.4	1,130
Rate of change in population size (% per year)					
2000-2010	0.0%	+1.1%	+2.5%	+3.7%	+1.1%
2010-2019	-0.1%	+0.6%	+2.6%	+3.2%	+0.9%
2000-2019	-0.1%	+0.9%	+2.5%	+3.5%	+1.0%
Deaths (thousands)					
2000	23.6	109	38.9	19.3	191
2010	19.2	108	45.6	21.5	195
2019	12.7	93.9	44.8	20.6	172
Rate of change in deaths (% per year)					
2000-2010	-2.0%	0.0%	+1.6%	+1.1%	+0.2%
2010-2019	-4.5%	-1.6%	-0.2%	-0.5%	-1.4%
2000-2019	-3.2%	-0.8%	+0.8%	+0.3%	-0.6%
Death rate (per 100 000 population per year)					
2000	8.5	22.2	30.5	49.4	20.4
2010	7.0	19.8	28.1	38.3	18.7
2019	4.7	16.1	21.9	27.6	15.2
Rate of change in death rate (% per year)					
2000-2010	-2.0%	-1.2%	-0.8%	-2.5%	-0.9%
2010-2019	-4.4%	-2.2%	-2.7%	-3.6%	-2.3%
2000-2019	-3.1%	-1.7%	-1.7%	-3.0%	-1.6%

Data source: Global Health Estimates 2021

Panel G1: Strongly tobacco-associated NCDs - World

	Age group				
	0-14	15-49	50-69	70+	All ages
Population size (millions)					
2000	1,870	3,210	808	269	6,160
2010	1,910	3,670	1,080	355	7,010
2019	2,030	3,930	1,380	451	7,800
Rate of change in population size (% per year)					
2000-2010	+0.2%	+1.3%	+2.9%	+2.8%	+1.3%
2010-2019	+0.7%	+0.8%	+2.9%	+2.7%	+1.2%
2000-2019	+0.4%	+1.1%	+2.9%	+2.7%	+1.2%
Deaths (thousands)					
2000	1.5	249	1,470	2,810	4,530
2010	1.3	242	1,550	3,260	5,050
2019	1.3	231	1,860	3,720	5,810
Rate of change in deaths (% per year)					
2000-2010	-1.7%	-0.3%	+0.6%	+1.5%	+1.1%
2010-2019	-0.3%	-0.5%	+2.0%	+1.5%	+1.6%
2000-2019	-1.0%	-0.4%	+1.3%	+1.5%	+1.3%
Death rate (per 100 000 population per year)					
2000	<0.1	7.8	181	1,040	73.5
2010	<0.1	6.6	144	918	72.1
2019	<0.1	5.9	134	825	74.5
Rate of change in death rate (% per year)					
2000-2010	.	-1.6%	-2.3%	-1.3%	-0.2%
2010-2019	.	-1.3%	-0.8%	-1.2%	+0.4%
2000-2019	.	-1.5%	-1.6%	-1.2%	+0.1%

Data source: Global Health Estimates 2021

Panel G2: Strongly tobacco-associated NCDs - Central and Eastern Europe

	Age group				
	0-14	15-49	50-69	70+	All ages
Population size (millions)					
2000	63.2	181	70.2	29.2	343
2010	50.2	169	78.6	34.2	332
2019	54.6	153	85.8	35.2	329
Rate of change in population size (% per year)					
2000-2010	-2.3%	-0.7%	+1.1%	+1.6%	-0.3%
2010-2019	+0.9%	-1.1%	+1.0%	+0.3%	-0.1%
2000-2019	-0.8%	-0.9%	+1.1%	+1.0%	-0.2%
Deaths (thousands)					
2000	<0.1	24.2	160	140	324
2010	<0.1	14.5	128	135	278
2019	<0.1	11.3	138	127	276
Rate of change in deaths (% per year)					
2000-2010	.	-5.0%	-2.2%	-0.4%	-1.5%
2010-2019	.	-2.8%	+0.8%	-0.7%	-0.1%
2000-2019	.	-3.9%	-0.8%	-0.5%	-0.8%
Death rate (per 100 000 population per year)					
2000	<0.1	13.4	227	481	94.4
2010	<0.1	8.6	163	396	83.7
2019	<0.1	7.3	161	361	84.0
Rate of change in death rate (% per year)					
2000-2010	.	-4.3%	-3.3%	-1.9%	-1.2%
2010-2019	.	-1.7%	-0.2%	-1.0%	0.0%
2000-2019	.	-3.1%	-1.8%	-1.5%	-0.6%

Data source: Global Health Estimates 2021

Panel G3: Strongly tobacco-associated NCDs - Central Asia

	Age group				
	0-14	15-49	50-69	70+	All ages
Population size (millions)					
2000	100	115	21.7	5.4	242
2010	114	153	29.3	7.3	303
2019	131	177	39.7	8.8	357
Rate of change in population size (% per year)					
2000-2010	+1.3%	+2.9%	+3.0%	+3.1%	+2.3%
2010-2019	+1.6%	+1.6%	+3.4%	+2.1%	+1.8%
2000-2019	+1.4%	+2.3%	+3.2%	+2.6%	+2.1%
Deaths (thousands)					
2000	<0.1	10.4	46.1	50.9	107
2010	<0.1	11.3	46.6	59.9	118
2019	<0.1	11.9	52.4	65.2	130
Rate of change in deaths (% per year)					
2000-2010	.	+0.9%	+0.1%	+1.7%	+0.9%
2010-2019	.	+0.5%	+1.3%	+0.9%	+1.1%
2000-2019	.	+0.7%	+0.7%	+1.3%	+1.0%
Death rate (per 100 000 population per year)					
2000	<0.1	9.1	212	939	44.4
2010	<0.1	7.4	159	819	38.9
2019	<0.1	6.7	132	738	36.3
Rate of change in death rate (% per year)					
2000-2010	.	-2.0%	-2.8%	-1.4%	-1.3%
2010-2019	.	-1.1%	-2.0%	-1.1%	-0.8%
2000-2019	.	-1.6%	-2.5%	-1.3%	-1.1%

Data source: Global Health Estimates 2021

Panel G4: Strongly tobacco-associated NCDs - China

	Age group				
	0-14	15-49	50-69	70+	All ages
Population size (millions)					
2000	312	723	180	54.9	1,270
2010	250	767	257	77.6	1,350
2019	259	708	354	103	1,420
Rate of change in population size (% per year)					
2000-2010	-2.2%	+0.6%	+3.6%	+3.5%	+0.6%
2010-2019	+0.4%	-0.9%	+3.6%	+3.1%	+0.6%
2000-2019	-1.0%	-0.1%	+3.6%	+3.3%	+0.6%
Deaths (thousands)					
2000	0.2	85.6	476	1,330	1,890
2010	<0.1	76.1	445	1,390	1,910
2019	<0.1	64.6	520	1,430	2,010
Rate of change in deaths (% per year)					
2000-2010	-13%	-1.2%	-0.7%	+0.4%	+0.1%
2010-2019	.	-1.8%	+1.7%	+0.3%	+0.6%
2000-2019	-8.2%	-1.5%	+0.5%	+0.4%	+0.3%
Death rate (per 100 000 population per year)					
2000	<0.1	11.8	265	2,420	149
2010	<0.1	9.9	173	1,790	142
2019	<0.1	9.1	147	1,390	141
Rate of change in death rate (% per year)					
2000-2010	.	-1.8%	-4.1%	-3.0%	-0.5%
2010-2019	.	-0.9%	-1.8%	-2.8%	0.0%
2000-2019	.	-1.4%	-3.0%	-2.9%	-0.3%

Data source: Global Health Estimates 2021

Panel G5: Strongly tobacco-associated NCDs - India

	Age group				
	0-14	15-49	50-69	70+	All ages
Population size (millions)					
2000	376	543	111	27.6	1,060
2010	390	659	157	37.8	1,240
2019	373	757	209	50.5	1,390
Rate of change in population size (% per year)					
2000-2010	+0.4%	+1.9%	+3.6%	+3.2%	+1.6%
2010-2019	-0.5%	+1.6%	+3.2%	+3.3%	+1.2%
2000-2019	0.0%	+1.8%	+3.4%	+3.2%	+1.4%
Deaths (thousands)					
2000	0.3	38.4	240	321	600
2010	0.2	47.6	311	505	864
2019	0.2	55.5	451	734	1,240
Rate of change in deaths (% per year)					
2000-2010	-3.0%	+2.2%	+2.6%	+4.6%	+3.7%
2010-2019	-1.6%	+1.7%	+4.2%	+4.2%	+4.1%
2000-2019	-2.3%	+1.9%	+3.4%	+4.4%	+3.9%
Death rate (per 100 000 population per year)					
2000	<0.1	7.1	217	1,160	56.7
2010	<0.1	7.2	198	1,340	69.5
2019	<0.1	7.3	216	1,450	89.3
Rate of change in death rate (% per year)					
2000-2010	.	+0.2%	-0.9%	+1.4%	+2.0%
2010-2019	.	+0.1%	+1.0%	+0.9%	+2.8%
2000-2019	.	+0.2%	0.0%	+1.2%	+2.4%

Data source: Global Health Estimates 2021

Panel G6: Strongly tobacco-associated NCDs - Latin America and Caribbean

	Age group				
	0-14	15-49	50-69	70+	All ages
Population size (millions)					
2000	166	272	59.4	17.7	515
2010	161	312	83.7	25.4	583
2019	155	337	110	34.6	636
Rate of change in population size (% per year)					
2000-2010	-0.3%	+1.4%	+3.5%	+3.7%	+1.2%
2010-2019	-0.5%	+0.8%	+3.1%	+3.5%	+1.0%
2000-2019	-0.4%	+1.1%	+3.3%	+3.6%	+1.1%
Deaths (thousands)					
2000	0.1	10.4	57.0	108	175
2010	0.1	10.0	67.2	140	217
2019	<0.1	9.0	79.7	169	258
Rate of change in deaths (% per year)					
2000-2010	+0.2%	-0.4%	+1.7%	+2.7%	+2.2%
2010-2019	-5.5%	-1.2%	+1.9%	+2.1%	+1.9%
2000-2019	-2.6%	-0.7%	+1.8%	+2.4%	+2.1%
Death rate (per 100 000 population per year)					
2000	<0.1	3.8	96.0	608	34.0
2010	<0.1	3.2	80.2	552	37.3
2019	<0.1	2.7	72.2	488	40.5
Rate of change in death rate (% per year)					
2000-2010	.	-1.7%	-1.8%	-1.0%	+0.9%
2010-2019	.	-2.0%	-1.2%	-1.4%	+0.9%
2000-2019	.	-1.8%	-1.5%	-1.2%	+0.9%

Data source: Global Health Estimates 2021

Panel G7: Strongly tobacco-associated NCDs - Middle East and North Africa

	Age group				
	0-14	15-49	50-69	70+	All ages
Population size (millions)					
2000	135	200	36.9	9.6	381
2010	140	259	53.6	13.8	467
2019	163	296	76.1	18.3	553
Rate of change in population size (% per year)					
2000-2010	+0.4%	+2.6%	+3.8%	+3.7%	+2.0%
2010-2019	+1.7%	+1.5%	+4.0%	+3.2%	+1.9%
2000-2019	+1.0%	+2.1%	+3.9%	+3.5%	+2.0%
Deaths (thousands)					
2000	0.1	10.0	42.6	43.3	96.0
2010	0.1	11.8	53.0	64.3	129
2019	0.1	12.5	71.1	85.3	169
Rate of change in deaths (% per year)					
2000-2010	-0.4%	+1.6%	+2.2%	+4.0%	+3.0%
2010-2019	-0.3%	+0.7%	+3.3%	+3.2%	+3.0%
2000-2019	-0.4%	+1.2%	+2.7%	+3.6%	+3.0%
Death rate (per 100 000 population per year)					
2000	0.1	5.0	115	451	25.2
2010	<0.1	4.5	98.8	466	27.7
2019	<0.1	4.2	93.4	465	30.5
Rate of change in death rate (% per year)					
2000-2010	-0.8%	-1.0%	-1.5%	+0.3%	+0.9%
2010-2019	.	-0.8%	-0.6%	0.0%	+1.1%
2000-2019	-1.3%	-0.9%	-1.1%	+0.2%	+1.0%

Data source: Global Health Estimates 2021

Panel G8: Strongly tobacco-associated NCDs - North Atlantic

	Age group				
	0-14	15-49	50-69	70+	All ages
Population size (millions)					
2000	72.0	209	93.2	47.7	422
2010	71.3	211	107	57.5	446
2019	72.1	202	122	67.8	464
Rate of change in population size (% per year)					
2000-2010	-0.1%	+0.1%	+1.4%	+1.9%	+0.6%
2010-2019	+0.1%	-0.4%	+1.5%	+1.9%	+0.4%
2000-2019	0.0%	-0.2%	+1.4%	+1.9%	+0.5%
Deaths (thousands)					
2000	<0.1	16.5	133	293	442
2010	<0.1	12.4	136	316	465
2019	<0.1	7.8	136	343	488
Rate of change in deaths (% per year)					
2000-2010	.	-2.8%	+0.3%	+0.8%	+0.5%
2010-2019	.	-5.0%	0.0%	+0.9%	+0.5%
2000-2019	.	-3.9%	+0.1%	+0.8%	+0.5%
Death rate (per 100 000 population per year)					
2000	<0.1	7.9	142	615	105
2010	<0.1	5.9	128	551	104
2019	<0.1	3.8	112	506	105
Rate of change in death rate (% per year)					
2000-2010	.	-2.9%	-1.1%	-1.1%	-0.1%
2010-2019	.	-4.6%	-1.5%	-0.9%	+0.1%
2000-2019	.	-3.7%	-1.2%	-1.0%	0.0%

Data source: Global Health Estimates 2021

Panel G9: Strongly tobacco-associated NCDs - Sub-Saharan Africa

	Age group				
	0-14	15-49	50-69	70+	All ages
Population size (millions)					
2000	305	313	52.5	11.8	682
2010	393	418	69.0	15.6	895
2019	482	544	93.4	20.4	1,140
Rate of change in population size (% per year)					
2000-2010	+2.6%	+2.9%	+2.8%	+2.8%	+2.8%
2010-2019	+2.3%	+3.0%	+3.4%	+3.0%	+2.7%
2000-2019	+2.4%	+3.0%	+3.1%	+2.9%	+2.7%
Deaths (thousands)					
2000	0.4	9.6	41.6	47.0	98.5
2010	0.4	12.6	50.1	60.3	123
2019	0.5	15.9	63.0	75.8	155
Rate of change in deaths (% per year)					
2000-2010	-0.4%	+2.8%	+1.9%	+2.5%	+2.3%
2010-2019	+3.2%	+2.6%	+2.6%	+2.6%	+2.6%
2000-2019	+1.3%	+2.7%	+2.2%	+2.6%	+2.4%
Death rate (per 100 000 population per year)					
2000	0.1	3.0	79.2	398	14.5
2010	0.1	3.0	72.6	387	13.8
2019	0.1	2.9	67.4	373	13.6
Rate of change in death rate (% per year)					
2000-2010	-2.9%	-0.1%	-0.9%	-0.3%	-0.5%
2010-2019	+0.9%	-0.4%	-0.8%	-0.4%	-0.1%
2000-2019	-1.1%	-0.2%	-0.8%	-0.3%	-0.3%

Data source: Global Health Estimates 2021

Panel G10: Strongly tobacco-associated NCDs - United States

	Age group				
	0-14	15-49	50-69	70+	All ages
Population size (millions)					
2000	59.6	146	51.4	24.9	281
2010	61.3	151	71.3	27.5	311
2019	62.9	157	82.2	35.5	338
Rate of change in population size (% per year)					
2000-2010	+0.3%	+0.4%	+3.3%	+1.0%	+1.0%
2010-2019	+0.3%	+0.4%	+1.6%	+2.9%	+0.9%
2000-2019	+0.3%	+0.4%	+2.5%	+1.9%	+1.0%
Deaths (thousands)					
2000	<0.1	10.2	92.3	209	311
2010	<0.1	8.1	105	217	330
2019	<0.1	4.6	103	234	342
Rate of change in deaths (% per year)					
2000-2010	.	-2.2%	+1.3%	+0.4%	+0.6%
2010-2019	.	-6.1%	-0.2%	+0.9%	+0.4%
2000-2019	.	-4.1%	+0.6%	+0.6%	+0.5%
Death rate (per 100 000 population per year)					
2000	<0.1	7.0	179	840	111
2010	<0.1	5.4	148	787	106
2019	<0.1	2.9	126	659	101
Rate of change in death rate (% per year)					
2000-2010	.	-2.6%	-1.9%	-0.6%	-0.4%
2010-2019	.	-6.6%	-1.8%	-1.9%	-0.5%
2000-2019	.	-4.5%	-1.9%	-1.3%	-0.5%

Data source: Global Health Estimates 2021

Panel G11: Strongly tobacco-associated NCDs - Western Pacific and Southeast Asia

	Age group				
	0-14	15-49	50-69	70+	All ages
Population size (millions)					
2000	276	492	127	38.9	934
2010	275	549	163	56.0	1,040
2019	272	582	205	74.4	1,130
Rate of change in population size (% per year)					
2000-2010	0.0%	+1.1%	+2.5%	+3.7%	+1.1%
2010-2019	-0.1%	+0.6%	+2.6%	+3.2%	+0.9%
2000-2019	-0.1%	+0.9%	+2.5%	+3.5%	+1.0%
Deaths (thousands)					
2000	0.3	32.3	172	255	460
2010	0.2	35.4	200	356	591
2019	0.2	36.5	232	443	711
Rate of change in deaths (% per year)					
2000-2010	-1.6%	+0.9%	+1.5%	+3.4%	+2.5%
2010-2019	-2.9%	+0.3%	+1.7%	+2.4%	+2.1%
2000-2019	-2.2%	+0.6%	+1.6%	+2.9%	+2.3%
Death rate (per 100 000 population per year)					
2000	<0.1	6.6	135	655	49.3
2010	<0.1	6.4	123	636	56.7
2019	<0.1	6.3	113	595	62.8
Rate of change in death rate (% per year)					
2000-2010	.	-0.2%	-1.0%	-0.3%	+1.4%
2010-2019	.	-0.3%	-0.9%	-0.7%	+1.1%
2000-2019	.	-0.2%	-0.9%	-0.5%	+1.3%

Data source: Global Health Estimates 2021

Panel H1: Suicide - World

	Age group				
	0-14	15-49	50-69	70+	All ages
Population size (millions)					
2000	1,870	3,210	808	269	6,160
2010	1,910	3,670	1,080	355	7,010
2019	2,030	3,930	1,380	451	7,800
Rate of change in population size (% per year)					
2000-2010	+0.2%	+1.3%	+2.9%	+2.8%	+1.3%
2010-2019	+0.7%	+0.8%	+2.9%	+2.7%	+1.2%
2000-2019	+0.4%	+1.1%	+2.9%	+2.7%	+1.2%
Deaths (thousands)					
2000	13.0	478	177	103	771
2010	10.1	450	180	109	749
2019	10.1	415	196	114	736
Rate of change in deaths (% per year)					
2000-2010	-2.5%	-0.6%	+0.2%	+0.6%	-0.3%
2010-2019	0.0%	-0.9%	+1.0%	+0.5%	-0.2%
2000-2019	-1.3%	-0.7%	+0.5%	+0.6%	-0.2%
Death rate (per 100 000 population per year)					
2000	0.7	14.9	21.9	38.1	12.5
2010	0.5	12.3	16.8	30.7	10.7
2019	0.5	10.6	14.2	25.3	9.4
Rate of change in death rate (% per year)					
2000-2010	-2.7%	-1.9%	-2.7%	-2.1%	-1.6%
2010-2019	-0.7%	-1.7%	-1.8%	-2.1%	-1.4%
2000-2019	-1.7%	-1.8%	-2.3%	-2.1%	-1.5%

Data source: Global Health Estimates 2021

Panel H2: Suicide - Central and Eastern Europe

	Age group				
	0-14	15-49	50-69	70+	All ages
Population size (millions)					
2000	63.2	181	70.2	29.2	343
2010	50.2	169	78.6	34.2	332
2019	54.6	153	85.8	35.2	329
Rate of change in population size (% per year)					
2000-2010	-2.3%	-0.7%	+1.1%	+1.6%	-0.3%
2010-2019	+0.9%	-1.1%	+1.0%	+0.3%	-0.1%
2000-2019	-0.8%	-0.9%	+1.1%	+1.0%	-0.2%
Deaths (thousands)					
2000	0.8	77.0	33.9	12.9	125
2010	0.3	50.7	25.7	11.5	88.2
2019	0.2	33.4	20.5	9.7	63.8
Rate of change in deaths (% per year)					
2000-2010	-8.3%	-4.1%	-2.7%	-1.2%	-3.4%
2010-2019	-4.4%	-4.5%	-2.5%	-1.9%	-3.5%
2000-2019	-6.5%	-4.3%	-2.6%	-1.5%	-3.5%
Death rate (per 100 000 population per year)					
2000	1.2	42.6	48.3	44.3	36.3
2010	0.6	30.0	32.7	33.6	26.6
2019	0.4	21.8	23.9	27.5	19.4
Rate of change in death rate (% per year)					
2000-2010	-6.2%	-3.4%	-3.8%	-2.7%	-3.1%
2010-2019	-5.3%	-3.5%	-3.4%	-2.2%	-3.5%
2000-2019	-5.8%	-3.5%	-3.6%	-2.5%	-3.2%

Data source: Global Health Estimates 2021

Panel H3: Suicide - Central Asia

	Age group				
	0-14	15-49	50-69	70+	All ages
Population size (millions)					
2000	100	115	21.7	5.4	242
2010	114	153	29.3	7.3	303
2019	131	177	39.7	8.8	357
Rate of change in population size (% per year)					
2000-2010	+1.3%	+2.9%	+3.0%	+3.1%	+2.3%
2010-2019	+1.6%	+1.6%	+3.4%	+2.1%	+1.8%
2000-2019	+1.4%	+2.3%	+3.2%	+2.6%	+2.1%
Deaths (thousands)					
2000	0.5	17.7	3.4	1.0	22.5
2010	0.4	18.7	3.3	1.1	23.6
2019	0.6	18.8	3.7	1.1	24.2
Rate of change in deaths (% per year)					
2000-2010	-1.7%	+0.6%	-0.2%	+1.7%	+0.5%
2010-2019	+3.3%	0.0%	+1.3%	-0.1%	+0.3%
2000-2019	+0.6%	+0.3%	+0.5%	+0.9%	+0.4%
Death rate (per 100 000 population per year)					
2000	0.5	15.4	15.4	17.9	9.3
2010	0.4	12.2	11.2	15.7	7.8
2019	0.5	10.6	9.3	12.9	6.8
Rate of change in death rate (% per year)					
2000-2010	-3.0%	-2.3%	-3.1%	-1.3%	-1.8%
2010-2019	+1.6%	-1.6%	-2.1%	-2.1%	-1.5%
2000-2019	-0.8%	-2.0%	-2.6%	-1.7%	-1.7%

Data source: Global Health Estimates 2021

Panel H4: Suicide - China

	Age group				
	0-14	15-49	50-69	70+	All ages
Population size (millions)					
2000	312	723	180	54.9	1,270
2010	250	767	257	77.6	1,350
2019	259	708	354	103	1,420
Rate of change in population size (% per year)					
2000-2010	-2.2%	+0.6%	+3.6%	+3.5%	+0.6%
2010-2019	+0.4%	-0.9%	+3.6%	+3.1%	+0.6%
2000-2019	-1.0%	-0.1%	+3.6%	+3.3%	+0.6%
Deaths (thousands)					
2000	2.2	94.7	53.3	46.6	197
2010	0.7	55.9	42.0	44.5	143
2019	0.6	40.4	42.7	40.9	125
Rate of change in deaths (% per year)					
2000-2010	-10%	-5.1%	-2.4%	-0.4%	-3.1%
2010-2019	-2.2%	-3.5%	+0.2%	-0.9%	-1.5%
2000-2019	-6.6%	-4.4%	-1.2%	-0.7%	-2.4%
Death rate (per 100 000 population per year)					
2000	0.7	13.1	29.6	84.8	15.5
2010	0.3	7.3	16.3	57.3	10.6
2019	0.2	5.7	12.1	39.9	8.8
Rate of change in death rate (% per year)					
2000-2010	-8.3%	-5.7%	-5.8%	-3.8%	-3.7%
2010-2019	-2.6%	-2.7%	-3.3%	-4.0%	-2.1%
2000-2019	-5.7%	-4.3%	-4.6%	-3.9%	-3.0%

Data source: Global Health Estimates 2021

Panel H5: Suicide - India

	Age group				
	0-14	15-49	50-69	70+	All ages
Population size (millions)					
2000	376	543	111	27.6	1,060
2010	390	659	157	37.8	1,240
2019	373	757	209	50.5	1,390
Rate of change in population size (% per year)					
2000-2010	+0.4%	+1.9%	+3.6%	+3.2%	+1.6%
2010-2019	-0.5%	+1.6%	+3.2%	+3.3%	+1.2%
2000-2019	0.0%	+1.8%	+3.4%	+3.2%	+1.4%
Deaths (thousands)					
2000	5.2	137	20.6	5.7	169
2010	4.2	158	26.8	8.5	198
2019	3.1	141	38.0	12.2	194
Rate of change in deaths (% per year)					
2000-2010	-2.1%	+1.4%	+2.7%	+4.1%	+1.6%
2010-2019	-3.3%	-1.3%	+3.9%	+4.1%	-0.2%
2000-2019	-2.7%	+0.1%	+3.3%	+4.1%	+0.8%
Death rate (per 100 000 population per year)					
2000	1.4	25.2	18.6	20.7	15.9
2010	1.1	24.0	17.1	22.5	15.9
2019	0.8	18.6	18.2	24.1	14.0
Rate of change in death rate (% per year)					
2000-2010	-2.5%	-0.5%	-0.8%	+0.8%	0.0%
2010-2019	-2.8%	-2.8%	+0.7%	+0.8%	-1.4%
2000-2019	-2.6%	-1.6%	-0.1%	+0.8%	-0.7%

Data source: Global Health Estimates 2021

Panel H6: Suicide - Latin America and Caribbean

	Age group				
	0-14	15-49	50-69	70+	All ages
Population size (millions)					
2000	166	272	59.4	17.7	515
2010	161	312	83.7	25.4	583
2019	155	337	110	34.6	636
Rate of change in population size (% per year)					
2000-2010	-0.3%	+1.4%	+3.5%	+3.7%	+1.2%
2010-2019	-0.5%	+0.8%	+3.1%	+3.5%	+1.0%
2000-2019	-0.4%	+1.1%	+3.3%	+3.6%	+1.1%
Deaths (thousands)					
2000	0.6	19.6	5.3	2.4	28.0
2010	0.7	22.6	6.5	3.1	32.9
2019	0.9	28.0	9.8	4.1	42.8
Rate of change in deaths (% per year)					
2000-2010	+1.6%	+1.4%	+1.9%	+2.5%	+1.6%
2010-2019	+2.9%	+2.4%	+4.7%	+3.2%	+3.0%
2000-2019	+2.2%	+1.9%	+3.2%	+2.8%	+2.3%
Death rate (per 100 000 population per year)					
2000	0.3	7.2	9.0	13.7	5.4
2010	0.4	7.2	7.7	12.2	5.6
2019	0.6	8.3	8.9	11.8	6.7
Rate of change in death rate (% per year)					
2000-2010	+1.9%	0.0%	-1.5%	-1.2%	+0.4%
2010-2019	+3.4%	+1.6%	+1.5%	-0.3%	+2.0%
2000-2019	+2.6%	+0.8%	-0.1%	-0.8%	+1.1%

Data source: Global Health Estimates 2021

Panel H7: Suicide - Middle East and North Africa

	Age group				
	0-14	15-49	50-69	70+	All ages
Population size (millions)					
2000	135	200	36.9	9.6	381
2010	140	259	53.6	13.8	467
2019	163	296	76.1	18.3	553
Rate of change in population size (% per year)					
2000-2010	+0.4%	+2.6%	+3.8%	+3.7%	+2.0%
2010-2019	+1.7%	+1.5%	+4.0%	+3.2%	+1.9%
2000-2019	+1.0%	+2.1%	+3.9%	+3.5%	+2.0%
Deaths (thousands)					
2000	0.4	10.9	1.4	0.5	13.3
2010	0.3	11.2	1.7	0.6	13.9
2019	0.3	11.5	2.4	0.8	15.0
Rate of change in deaths (% per year)					
2000-2010	-2.9%	+0.3%	+2.1%	+1.6%	+0.5%
2010-2019	-2.8%	+0.3%	+3.7%	+3.3%	+0.8%
2000-2019	-2.9%	+0.3%	+2.9%	+2.4%	+0.6%
Death rate (per 100 000 population per year)					
2000	0.3	5.5	3.8	5.6	3.5
2010	0.2	4.3	3.2	4.6	3.0
2019	0.2	3.9	3.2	4.6	2.7
Rate of change in death rate (% per year)					
2000-2010	-3.3%	-2.3%	-1.6%	-2.0%	-1.6%
2010-2019	-4.4%	-1.2%	-0.3%	0.0%	-1.0%
2000-2019	-3.8%	-1.8%	-1.0%	-1.0%	-1.3%

Data source: Global Health Estimates 2021

Panel H8: Suicide - North Atlantic

	Age group				
	0-14	15-49	50-69	70+	All ages
Population size (millions)					
2000	72.0	209	93.2	47.7	422
2010	71.3	211	107	57.5	446
2019	72.1	202	122	67.8	464
Rate of change in population size (% per year)					
2000-2010	-0.1%	+0.1%	+1.4%	+1.9%	+0.6%
2010-2019	+0.1%	-0.4%	+1.5%	+1.9%	+0.4%
2000-2019	0.0%	-0.2%	+1.4%	+1.9%	+0.5%
Deaths (thousands)					
2000	0.2	26.2	14.8	12.0	53.2
2010	0.2	23.0	16.6	11.9	51.6
2019	0.1	20.6	17.8	13.4	52.0
Rate of change in deaths (% per year)					
2000-2010	-1.9%	-1.3%	+1.1%	-0.1%	-0.3%
2010-2019	-0.8%	-1.2%	+0.8%	+1.3%	+0.1%
2000-2019	-1.4%	-1.3%	+1.0%	+0.6%	-0.1%
Death rate (per 100 000 population per year)					
2000	0.3	12.5	15.9	25.1	12.6
2010	0.2	10.9	15.6	20.7	11.6
2019	0.2	10.2	14.7	19.7	11.2
Rate of change in death rate (% per year)					
2000-2010	-1.8%	-1.4%	-0.2%	-1.9%	-0.9%
2010-2019	-0.9%	-0.8%	-0.7%	-0.5%	-0.4%
2000-2019	-1.4%	-1.1%	-0.4%	-1.3%	-0.6%

Data source: Global Health Estimates 2021

Panel H9: Suicide - Sub-Saharan Africa

	Age group				
	0-14	15-49	50-69	70+	All ages
Population size (millions)					
2000	305	313	52.5	11.8	682
2010	393	418	69.0	15.6	895
2019	482	544	93.4	20.4	1,140
Rate of change in population size (% per year)					
2000-2010	+2.6%	+2.9%	+2.8%	+2.8%	+2.8%
2010-2019	+2.3%	+3.0%	+3.4%	+3.0%	+2.7%
2000-2019	+2.4%	+3.0%	+3.1%	+2.9%	+2.7%
Deaths (thousands)					
2000	1.9	25.7	13.5	6.4	47.5
2010	2.1	36.2	16.4	8.0	62.8
2019	2.9	46.7	20.5	10.2	80.3
Rate of change in deaths (% per year)					
2000-2010	+1.3%	+3.5%	+2.0%	+2.3%	+2.8%
2010-2019	+3.5%	+2.9%	+2.5%	+2.7%	+2.8%
2000-2019	+2.3%	+3.2%	+2.2%	+2.5%	+2.8%
Death rate (per 100 000 population per year)					
2000	0.6	8.2	25.7	54.0	7.0
2010	0.5	8.7	23.8	51.5	7.0
2019	0.6	8.6	21.9	50.0	7.0
Rate of change in death rate (% per year)					
2000-2010	-1.3%	+0.5%	-0.8%	-0.5%	+0.1%
2010-2019	+1.2%	-0.1%	-0.9%	-0.3%	0.0%
2000-2019	-0.1%	+0.2%	-0.8%	-0.4%	+0.1%

Data source: Global Health Estimates 2021

Panel H10: Suicide - United States

	Age group				
	0-14	15-49	50-69	70+	All ages
Population size (millions)					
2000	59.6	146	51.4	24.9	281
2010	61.3	151	71.3	27.5	311
2019	62.9	157	82.2	35.5	338
Rate of change in population size (% per year)					
2000-2010	+0.3%	+0.4%	+3.3%	+1.0%	+1.0%
2010-2019	+0.3%	+0.4%	+1.6%	+2.9%	+0.9%
2000-2019	+0.3%	+0.4%	+2.5%	+1.9%	+1.0%
Deaths (thousands)					
2000	0.3	19.2	6.7	4.9	31.2
2010	0.3	22.6	13.1	4.7	40.7
2019	0.6	27.4	15.8	6.9	50.6
Rate of change in deaths (% per year)					
2000-2010	-1.3%	+1.6%	+6.9%	-0.5%	+2.7%
2010-2019	+8.7%	+2.1%	+2.0%	+4.3%	+2.4%
2000-2019	+3.3%	+1.9%	+4.6%	+1.8%	+2.6%
Death rate (per 100 000 population per year)					
2000	0.5	13.2	13.1	19.9	11.1
2010	0.4	15.0	18.4	17.1	13.1
2019	0.9	17.4	19.2	19.5	15.0
Rate of change in death rate (% per year)					
2000-2010	-1.6%	+1.3%	+3.5%	-1.5%	+1.7%
2010-2019	+8.4%	+1.7%	+0.4%	+1.4%	+1.5%
2000-2019	+3.0%	+1.5%	+2.0%	-0.1%	+1.6%

Data source: Global Health Estimates 2021

Panel H11: Suicide - Western Pacific and Southeast Asia

	Age group				
	0-14	15-49	50-69	70+	All ages
Population size (millions)					
2000	276	492	127	38.9	934
2010	275	549	163	56.0	1,040
2019	272	582	205	74.4	1,130
Rate of change in population size (% per year)					
2000-2010	0.0%	+1.1%	+2.5%	+3.7%	+1.1%
2010-2019	-0.1%	+0.6%	+2.6%	+3.2%	+0.9%
2000-2019	-0.1%	+0.9%	+2.5%	+3.5%	+1.0%
Deaths (thousands)					
2000	0.8	47.8	23.2	9.5	81.4
2010	0.8	48.6	26.6	14.1	90.1
2019	0.8	45.9	23.7	13.9	84.2
Rate of change in deaths (% per year)					
2000-2010	-0.3%	+0.2%	+1.4%	+4.0%	+1.0%
2010-2019	-0.4%	-0.6%	-1.3%	-0.2%	-0.7%
2000-2019	-0.3%	-0.2%	+0.1%	+2.0%	+0.2%
Death rate (per 100 000 population per year)					
2000	0.3	9.7	18.3	24.4	8.7
2010	0.3	8.8	16.3	25.2	8.6
2019	0.3	7.9	11.6	18.7	7.4
Rate of change in death rate (% per year)					
2000-2010	-0.2%	-0.9%	-1.1%	+0.3%	-0.1%
2010-2019	-0.3%	-1.3%	-3.8%	-3.3%	-1.6%
2000-2019	-0.2%	-1.1%	-2.4%	-1.4%	-0.8%

Data source: Global Health Estimates 2021

Table A10. Decomposition of I-8 and NCDI-7 deaths into component (changes in population size, population structure, and age-specific mortality rates) contributions, by sex for World, 2000-2010, 2010-2019.

(A) Females I-8 deaths

	**Deaths, year 1**	**Change in deaths**	**Number of deaths contributed by component (N) and share of total component effects (%) over the decade**					
			Population size		Population structure		Age-specific mortality rates	
	N, thousands	N, thousands	N, thousands	(%)	N, thousands	(%)	N, thousands	(%)
2000-2010								
Childhood-cluster diseases	541	-298	+51	(13%)	-26	(7%)	-323	(81%)
Diarrheal diseases	1 160	-296	+131	(21%)	+34	(5%)	-462	(74%)
HIV/AIDS	815	-212	+92	(22%)	+13	(3%)	-317	(75%)
Lower respiratory infections	1 370	-143	+167	(28%)	+56	(9%)	-367	(62%)
Malaria	421	-75	+50	(28%)	-19	(11%)	-106	(61%)
Maternal conditions	411	-101	+47	(24%)	-1	(0%)	-147	(76%)
Neonatal conditions	1 490	-315	+172	(26%)	-77	(12%)	-410	(62%)
Tuberculosis	1 030	-281	+116	(18%)	+60	(10%)	-457	(72%)
I-8	7 240	-1 720	+826	(24%)	+40	(1%)	-2 590	(75%)
2010-2019								
Childhood-cluster diseases	243	-97	+21	(15%)	-12	(9%)	-106	(76%)
Diarrheal diseases	865	-183	+83	(20%)	+34	(8%)	-301	(72%)
HIV/AIDS	603	-295	+49	(12%)	-1	(0%)	-343	(87%)
Lower respiratory infections	1 230	+19	+131	(34%)	+74	(19%)	-187	(48%)
Malaria	346	-66	+33	(25%)	-20	(15%)	-79	(60%)
Maternal conditions	310	-70	+29	(23%)	-12	(9%)	-88	(68%)
Neonatal conditions	1 180	-260	+112	(23%)	-125	(26%)	-247	(51%)
Tuberculosis	745	-230	+68	(15%)	+41	(9%)	-339	(76%)
I-8	5 520	-1 180	+527	(24%)	-21	(1%)	-1 690	(76%)

(B) Females NCDI-7 deaths

	Deaths, year 1	Change in deaths	Number of deaths contributed by component (N) and share of total component effects (%) over the decade					
			Population size		Population structure		Age-specific mortality rates	
	N, thousands	N, thousands	N, thousands	(%)	N, thousands	(%)	N, thousands	(%)
2000-2010								
Atherosclerotic CVD	4 610	+565	+629	(28%)	+793	(35%)	-857	(38%)
Diabetes	585	+187	+86	(46%)	+88	(47%)	+13	(7%)
Hemorrhagic stroke	1 520	+50	+199	(26%)	+206	(27%)	-355	(47%)
Infection-associated NCDs	1 060	+32	+138	(28%)	+126	(25%)	-232	(47%)
Road injury	307	-9	+39	(36%)	+11	(10%)	-58	(54%)
Strongly tobacco-associated NCDs	1 780	+134	+237	(27%)	+275	(31%)	-378	(42%)
Suicide	270	-22	+33	(29%)	+14	(12%)	-69	(59%)
NCDI-7	10 100	+937	+1 360	(28%)	+1 510	(31%)	-1 940	(40%)
2010-2019								
Atherosclerotic CVD	5 180	+562	+582	(25%)	+863	(37%)	-883	(38%)
Diabetes	771	+270	+96	(35%)	+128	(48%)	+46	(17%)
Hemorrhagic stroke	1 570	+63	+171	(23%)	+227	(31%)	-335	(46%)
Infection-associated NCDs	1 090	+77	+120	(28%)	+131	(31%)	-174	(41%)
Road injury	298	-12	+31	(33%)	+11	(11%)	-53	(56%)
Strongly tobacco-associated NCDs	1 910	+343	+221	(30%)	+314	(43%)	-192	(26%)
Suicide	248	-20	+25	(29%)	+8	(9%)	-53	(62%)
NCDI-7	11 100	+1 280	+1 250	(27%)	+1 680	(37%)	-1 640	(36%)

(C) Males I-8 deaths

	Deaths, year 1	Change in deaths	Number of deaths contributed by component (N) and share of total component effects (%) over the decade					
			Population size		Population structure		Age-specific mortality rates	
	N, thousands	N, thousands	N, thousands	(%)	N, thousands	(%)	N, thousands	(%)
2000-2010								
Childhood-cluster diseases	537	-295	+52	(13%)	-25	(6%)	-322	(81%)
Diarrheal diseases	1 140	-278	+132	(22%)	+34	(6%)	-445	(73%)
HIV/AIDS	820	-174	+97	(24%)	+22	(5%)	-292	(71%)
Lower respiratory infections	1 490	-122	+188	(29%)	+79	(12%)	-388	(59%)
Malaria	445	-78	+53	(29%)	-20	(11%)	-111	(60%)
Neonatal conditions	1 800	-370	+212	(27%)	-98	(12%)	-484	(61%)
Tuberculosis	1 490	-305	+178	(19%)	+131	(14%)	-614	(66%)
I-8	7 730	-1 620	+912	(25%)	+122	(3%)	-2 660	(72%)
2010-2019								
Childhood-cluster diseases	242	-96	+21	(15%)	-14	(10%)	-103	(75%)
Diarrheal diseases	864	-223	+82	(19%)	+24	(6%)	-328	(76%)
HIV/AIDS	646	-239	+57	(16%)	+7	(2%)	-303	(83%)
Lower respiratory infections	1 370	+12	+148	(32%)	+90	(20%)	-227	(49%)
Malaria	367	-69	+36	(26%)	-24	(17%)	-81	(58%)
Neonatal conditions	1 430	-293	+138	(24%)	-167	(29%)	-265	(46%)
Tuberculosis	1 190	-376	+109	(14%)	+89	(12%)	-574	(74%)
I-8	6 110	-1 280	+591	(24%)	+6	(0%)	-1 880	(76%)

(D) Males NCDI-7 deaths

	Deaths, year 1	Change in deaths	Number of deaths contributed by component (N) and share of total component effects (%) over the decade					
			Population size		Population structure		Age-specific mortality rates	
	N, thousands	N, thousands	N, thousands	(%)	N, thousands	(%)	N, thousands	(%)
2000-2010								
Atherosclerotic CVD	4 370	+1 040	+636	(32%)	+888	(44%)	-489	(24%)
Diabetes	498	+204	+77	(38%)	+94	(46%)	+33	(16%)
Hemorrhagic stroke	1 540	+213	+215	(29%)	+259	(35%)	-262	(36%)
Infection-associated NCDs	1 260	+115	+172	(30%)	+177	(30%)	-234	(40%)
Road injury	877	+75	+119	(50%)	+37	(16%)	-81	(34%)
Strongly tobacco-associated NCDs	2 740	+401	+384	(27%)	+522	(37%)	-506	(36%)
Suicide	502	0	+66	(32%)	+36	(18%)	-101	(50%)
NCDI-7	11 800	+2 040	+1 670	(31%)	+2 010	(38%)	-1 640	(31%)
2010-2019								
Atherosclerotic CVD	5 410	+916	+629	(26%)	+1 030	(43%)	-741	(31%)
Diabetes	703	+264	+89	(34%)	+132	(50%)	+44	(17%)
Hemorrhagic stroke	1 760	+100	+195	(23%)	+285	(33%)	-379	(44%)
Infection-associated NCDs	1 370	+43	+150	(24%)	+185	(30%)	-292	(47%)
Road injury	953	-42	+100	(34%)	+25	(9%)	-167	(57%)
Strongly tobacco-associated NCDs	3 140	+419	+360	(24%)	+594	(40%)	-534	(36%)
Suicide	502	+7	+54	(36%)	+25	(16%)	-72	(48%)
NCDI-7	13 800	+1 710	+1 580	(26%)	+2 270	(38%)	-2 140	(36%)

Note: Year 1 = The first year of the decadal period (2000 for 2000s, 2010 for 2010s)

Figure A11. Decomposition of the number of deaths due to the 15 priority conditions into changes in age-specific mortality rates and changes in population size and structure, by sex for the World, 2000-2010, 2010-2019.

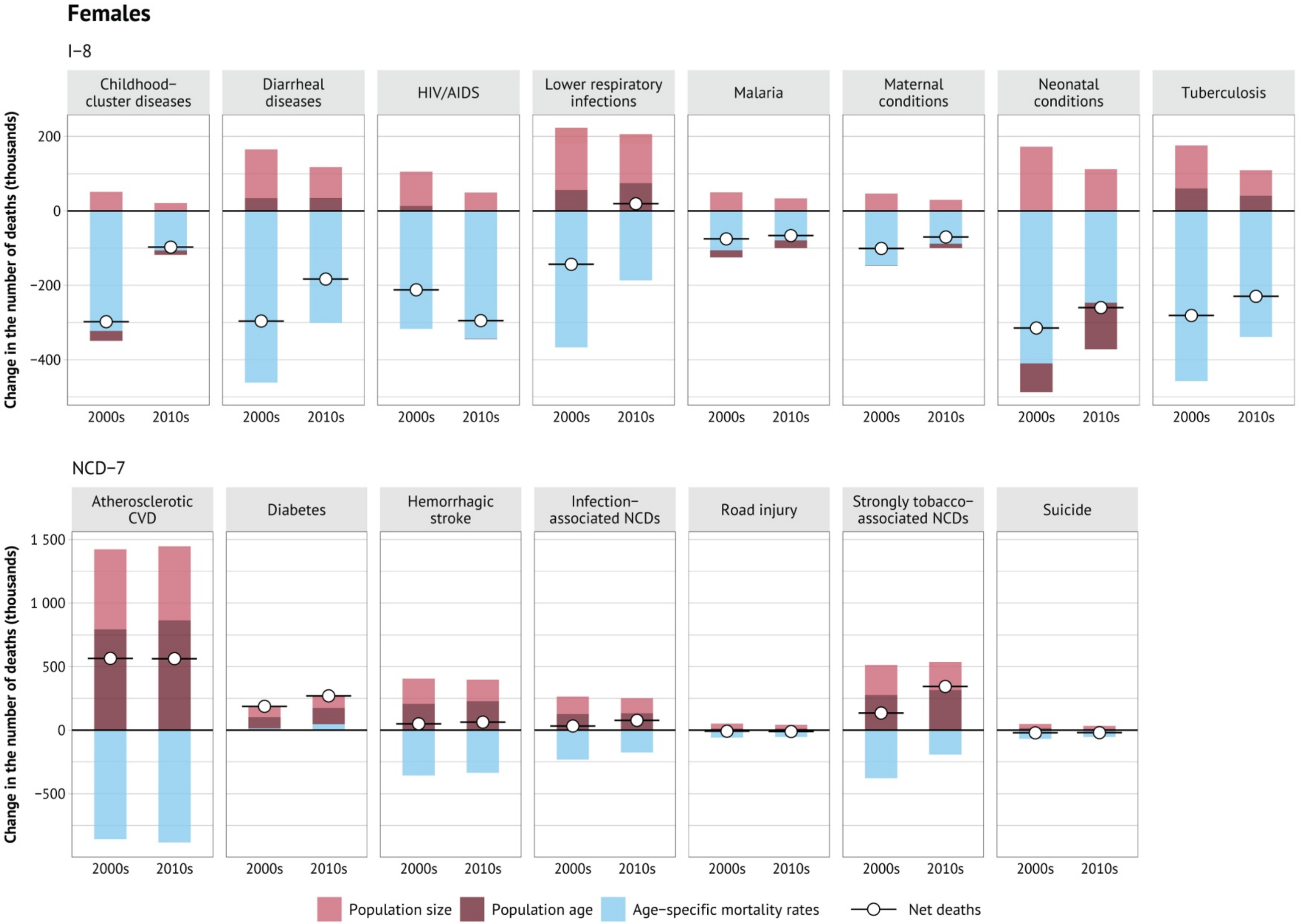

Males

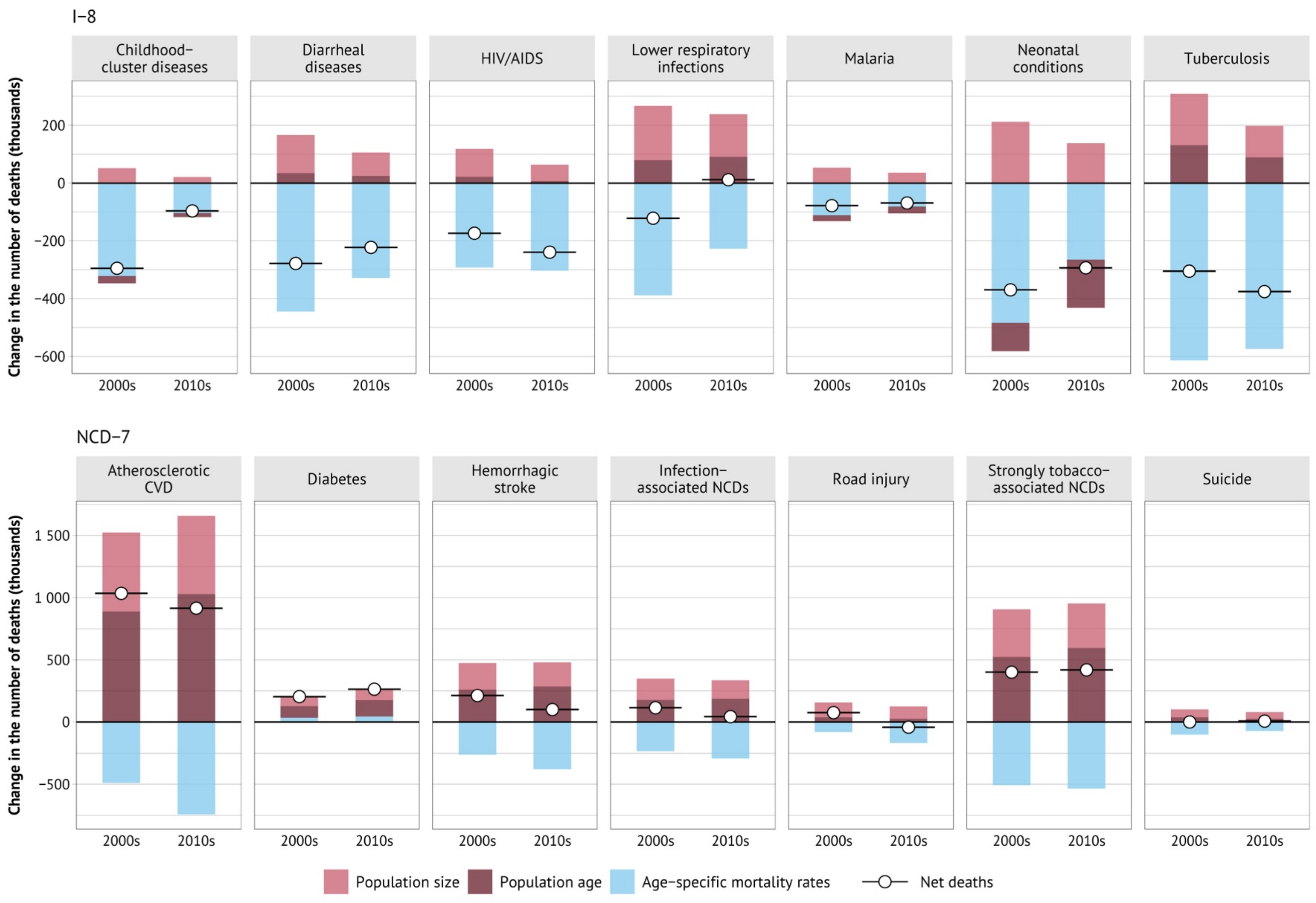

I–8
Childhood–cluster diseases
Diarrheal diseases
HIV/AIDS
Lower respiratory infections
Malaria
Neonatal conditions
Tuberculosis
Change in the number of deaths (thousands)
200
0
−200
−400
−600
2000s
2010s
NCD–7
Atherosclerotic CVD
Diabetes
Hemorrhagic stroke
Infection–associated NCDs
Road injury
Strongly tobacco–associated NCDs
Suicide
Change in the number of deaths (thousands)
1 500
1 000
500
0
−500
Population size
Population age
Age–specific mortality rates
Net deaths

Table A11. Decomposition I-8 deaths into component (changes in population size, population structure, and age-specific mortality rates) contributions by region, 2000-2010 and 2010-2019.

	Total deaths				Deaths due to changes in population size				Deaths due to changes in population structure				Deaths due to changes in age-specific mortality rates			
	2000	2019	Δ 2000-2010	Δ 2010-2019	Δ 2000-2010		Δ 2010-2019		Δ 2000-2010		Δ 2010-2019		Δ 2000-2010		Δ 2010-2019	
	N	N	N	N	N	%	N	%	N	%	N	%	N	%	N	%
World																
Childhood-cluster diseases	1080	291	-593	-193	+103	13%	+41.8	15%	-51.3	6%	-26.0	9%	-644	81%	-209	76%
Diarrheal diseases	2300	1320	-574	-406	+264	21%	+165	19%	+66.2	5%	+57.8	7%	-904	73%	-628	74%
HIV/AIDS	1630	715	-384	-534	+189	23%	+107	14%	+34.9	4%	+5.2	1%	-608	73%	-646	85%
Lower respiratory infections	2870	2630	-266	+29.4	+355	29%	+279	33%	+128	10%	+158	19%	-749	61%	-408	48%
Malaria	867	578	-153	-135	+103	29%	+69.2	25%	-39.4	11%	-44.2	16%	-217	60%	-160	58%
Maternal conditions	410	240	-101	-69.6	+47.0	24%	+29.5	23%	+0.1	0%	-10.3	8%	-148	76%	-88.8	69%
Neonatal conditions	3300	2060	-685	-553	+384	26%	+250	24%	-175	12%	-291	28%	-894	62%	-512	49%
Tuberculosis	2520	1330	-586	-606	+293	19%	+177	15%	+185	12%	+125	10%	-1060	69%	-909	75%
I-8	15000	9170	-3340	-2470	+1740	24%	+1120	24%	+148	2%	-24.8	1%	-5230	74%	-3560	76%
Central and Eastern Europe																
Childhood-cluster diseases	0.9	0.1	-0.8	0.0	0.0	2%	0.0	4%	-0.1	9%	0.0	4%	-0.7	89%	0.0	92%
Diarrheal diseases	2.5	2.7	-1.2	+1.4	-0.1	4%	0.0	1%	+0.3	17%	+0.3	20%	-1.5	79%	+1.1	79%
HIV/AIDS	17.4	33.8	+13.0	+3.4	-0.8	6%	-0.3	6%	+0.5	3%	-0.1	3%	+13.3	91%	+3.8	90%
Lower respiratory infections	78.4	83.4	-2.8	+7.8	-2.6	12%	-0.7	6%	+9.8	44%	+9.6	84%	-10.0	44%	-1.1	10%
Malaria	0.0	0.0	0.0	0.0	0.0	-	0.0	-	0.0	-	0.0	-	0.0	-	0.0	-
Maternal conditions	1.2	0.3	-0.4	-0.4	0.0	7%	0.0	1%	0.0	3%	0.0	12%	-0.4	90%	-0.3	87%
Neonatal conditions	20.1	7.3	-7.1	-5.7	-0.6	4%	-0.1	2%	+3.8	26%	-1.2	21%	-10.3	70%	-4.4	78%
Tuberculosis	53.8	18.4	-13.5	-21.9	-1.6	7%	-0.2	1%	+4.2	19%	+0.4	2%	-16.1	74%	-22.1	97%
I-8	174	146	-12.9	-15.4	-5.8	12%	-1.3	4%	+18.5	37%	+8.9	27%	-25.7	51%	-23.0	69%
Central Asia																
Childhood-cluster diseases	114	36.6	-13.1	-63.8	+24.5	40%	+11.6	13%	-7.1	11%	-2.8	3%	-30.5	49%	-72.5	83%
Diarrheal diseases	138	67.2	-40.5	-30.5	+27.4	29%	+13.7	24%	-1.9	2%	-1.3	2%	-66.0	69%	-42.9	74%
HIV/AIDS	0.5	14.5	+5.0	+9.0	+0.6	12%	+1.6	18%	+0.1	2%	+0.3	4%	+4.3	85%	+7.1	79%
Lower respiratory infections	152	102	-25.3	-25.2	+32.2	36%	+18.9	30%	-4.4	5%	-4.2	7%	-53.1	59%	-39.8	63%
Malaria	1.9	0.7	+0.8	-2.0	+0.5	54%	+0.3	11%	-0.1	10%	0.0	0%	+0.3	35%	-2.3	88%
Maternal conditions	34.3	27.0	-1.8	-5.5	+7.7	36%	+4.9	32%	+2.1	10%	-0.1	1%	-11.6	54%	-10.3	67%

	Total deaths				Deaths due to changes in population size				Deaths due to changes in population structure				Deaths due to changes in age-specific mortality rates			
	2000	2019	Δ 2000-2010	Δ 2010-2019	Δ 2000-2010		Δ 2010-2019		Δ 2000-2010		Δ 2010-2019		Δ 2000-2010		Δ 2010-2019	
	N	N	N	N	N	%	N	%	N	%	N	%	N	%	N	%
Neonatal conditions	347	292	+0.3	-55.6	+79.2	50%	+52.5	33%	-14.4	9%	-30.8	19%	-64.6	41%	-77.2	48%
Tuberculosis	86.4	61.2	-12.2	-13.0	+18.6	33%	+11.2	27%	+3.3	6%	+3.2	8%	-34.0	61%	-27.4	66%
I-8	874	601	-86.8	-187	+191	41%	+115	28%	-22.3	5%	-35.9	9%	-255	54%	-265	64%
China																
Childhood-cluster diseases	20.6	2.2	-13.5	-4.8	+0.9	6%	+0.2	5%	-1.0	7%	-0.4	7%	-13.4	88%	-4.7	88%
Diarrheal diseases	44.9	8.3	-30.0	-6.5	+1.9	5%	+0.6	8%	+1.4	4%	-0.2	3%	-33.3	91%	-6.9	89%
HIV/AIDS	27.8	37.2	+26.4	-17.0	+2.5	10%	+2.4	11%	+3.7	14%	+0.1	1%	+20.2	76%	-19.5	88%
Lower respiratory infections	289	202	-82.5	-4.1	+15.8	7%	+10.7	9%	+63.3	26%	+49.3	40%	-162	67%	-64.1	52%
Malaria	0.0	0.0	0.0	0.0	0.0	8%	0.0	2%	0.0	2%	0.0	2%	0.0	91%	0.0	95%
Maternal conditions	9.7	2.6	-4.2	-2.8	+0.5	9%	+0.2	6%	-0.4	8%	-0.5	16%	-4.3	82%	-2.5	78%
Neonatal conditions	239	40.5	-134	-64.2	+10.9	7%	+3.7	5%	-1.0	1%	-15.9	22%	-144	92%	-52.0	73%
Tuberculosis	107	37.8	-51.9	-17.1	+5.3	5%	+2.5	6%	+18.3	18%	+9.3	23%	-75.5	76%	-28.8	71%
I-8	737	331	-290	-117	+37.7	7%	+20.3	8%	+84.1	16%	+41.7	17%	-412	77%	-179	74%
India																
Childhood-cluster diseases	333	36.1	-183	-114	+39.5	15%	+10.3	8%	-40.7	16%	-15.5	12%	-182	69%	-108	81%
Diarrheal diseases	988	527	-258	-203	+141	23%	+71.1	14%	+42.5	7%	+88.7	17%	-441	71%	-363	69%
HIV/AIDS	220	44.8	-52.7	-123	+32.0	22%	+12.4	7%	+13.9	10%	+9.5	6%	-98.6	68%	-145	87%
Lower respiratory infections	597	428	-110	-59.0	+87.9	31%	+50.4	26%	-41.8	15%	+18.8	10%	-156	55%	-128	65%
Malaria	29.7	7.7	+1.0	-22.9	+4.9	56%	+2.2	8%	-1.7	19%	-0.8	3%	-2.2	25%	-24.3	89%
Maternal conditions	111	25.7	-62.0	-23.6	+13.5	14%	+4.2	13%	+2.7	3%	+0.8	2%	-78.2	83%	-28.6	85%
Neonatal conditions	1060	432	-342	-287	+145	23%	+63.8	15%	-191	30%	-116	28%	-296	47%	-235	57%
Tuberculosis	907	334	-335	-238	+124	17%	+51.7	11%	+73.8	10%	+62.6	13%	-533	73%	-352	76%
I-8	4250	1830	-1340	-1070	+587	23%	+266	16%	-142	6%	+48.5	3%	-1790	71%	-1380	82%
Latin America and Caribbean																
Childhood-cluster diseases	4.2	1.6	-2.5	-0.2	+0.4	12%	+0.1	32%	-0.4	12%	-0.2	37%	-2.4	76%	-0.1	31%
Diarrheal diseases	46.3	22.3	-23.7	-0.2	+4.3	13%	+2.0	27%	-1.0	3%	+1.5	21%	-26.9	84%	-3.8	52%
HIV/AIDS	53.7	38.9	-8.3	-6.6	+6.2	23%	+3.7	20%	+3.2	12%	+2.3	12%	-17.6	65%	-12.6	68%
Lower respiratory infections	148	257	+30.8	+78.2	+19.9	36%	+18.9	24%	+23.5	42%	+39.5	50%	-12.6	22%	+19.8	25%
Malaria	1.0	0.5	-0.5	0.0	+0.1	14%	0.0	40%	0.0	1%	0.0	0%	-0.6	85%	-0.1	60%

	Total deaths				Deaths due to changes in population size				Deaths due to changes in population structure				Deaths due to changes in age-specific mortality rates			
	2000	2019	Δ 2000-2010	Δ 2010-2019	Δ 2000-2010		Δ 2010-2019		Δ 2000-2010		Δ 2010-2019		Δ 2000-2010		Δ 2010-2019	
	N	N	N	N	N	%	N	%	N	%	N	%	N	%	N	%
Maternal conditions	9.4	8.1	-1.0	-0.3	+1.1	32%	+0.7	42%	+0.1	3%	-0.1	6%	-2.2	65%	-0.9	51%
Neonatal conditions	153	74.2	-59.2	-19.5	+15.3	17%	+7.4	22%	-22.7	25%	-13.0	38%	-51.8	58%	-13.9	41%
Tuberculosis	34.8	22.4	-9.5	-2.9	+3.8	15%	+2.1	16%	+4.2	17%	+3.2	24%	-17.5	69%	-8.2	61%
I-8	450	425	-73.9	+48.6	+50.9	27%	+35.1	40%	+6.9	4%	+33.3	38%	-132	70%	-19.8	22%
Middle East and North Africa																
Childhood-cluster diseases	20.6	6.9	-14.6	+0.9	+2.8	14%	+1.1	67%	-1.2	6%	-0.4	21%	-16.2	80%	+0.2	12%
Diarrheal diseases	40.2	11.1	-21.8	-7.3	+6.2	18%	+2.6	21%	-0.7	2%	-0.7	5%	-27.3	80%	-9.2	74%
HIV/AIDS	2.0	5.1	+3.6	-0.5	+0.7	20%	+0.9	30%	+0.4	11%	+0.4	12%	+2.5	69%	-1.8	58%
Lower respiratory infections	97.2	92.2	-12.4	+7.4	+18.6	33%	+15.1	48%	+3.1	6%	+4.2	14%	-34.2	61%	-11.8	38%
Malaria	2.8	2.4	+0.3	-0.7	+0.6	61%	+0.5	29%	0.0	3%	-0.1	8%	-0.4	36%	-1.0	64%
Maternal conditions	7.6	5.4	-1.3	-0.8	+1.4	27%	+1.0	36%	+0.5	10%	-0.2	8%	-3.3	63%	-1.6	56%
Neonatal conditions	167	104	-33.5	-29.6	+30.9	32%	+20.4	29%	-3.9	4%	-15.3	22%	-60.5	64%	-34.7	49%
Tuberculosis	15.4	12.8	-2.5	-0.1	+2.9	25%	+2.2	30%	+1.7	14%	+1.4	19%	-7.1	61%	-3.7	51%
I-8	353	240	-82.3	-30.6	+64.2	30%	+43.7	37%	-0.1	0%	-10.8	9%	-146	70%	-63.6	54%
North Atlantic																
Childhood-cluster diseases	0.1	0.1	0.0	0.0	0.0	7%	0.0	17%	0.0	15%	0.0	47%	-0.1	79%	0.0	36%
Diarrheal diseases	3.0	13.7	+8.0	+2.7	+0.4	5%	+0.5	18%	+1.2	15%	+2.1	80%	+6.4	80%	+0.1	3%
HIV/AIDS	7.3	2.1	-2.6	-2.6	+0.3	10%	+0.1	5%	0.0	1%	0.0	1%	-2.9	89%	-2.7	94%
Lower respiratory infections	166	151	-44.5	+29.4	+8.3	7%	+5.3	17%	+28.3	24%	+24.7	80%	-81.0	69%	-0.7	2%
Malaria	0.0	0.0	0.0	0.0	0.0	-	0.0	-	0.0	-	0.0	-	0.0	-	0.0	-
Maternal conditions	0.4	0.3	0.0	-0.1	0.0	28%	0.0	13%	0.0	44%	0.0	24%	0.0	28%	-0.1	63%
Neonatal conditions	10.9	9.8	-1.2	+0.2	+0.6	24%	+0.4	14%	0.0	0%	-1.3	47%	-1.8	76%	+1.1	39%
Tuberculosis	4.6	2.0	-1.7	-1.0	+0.2	7%	+0.1	5%	+0.5	17%	+0.3	18%	-2.4	76%	-1.4	77%
I-8	193	179	-42.1	+28.5	+9.8	8%	+6.4	18%	+30.0	25%	+25.8	72%	-81.9	67%	-3.8	10%
Sub-Saharan Africa																
Childhood-cluster diseases	495	190	-305	+0.2	+99.8	20%	+46.2	50%	-7.3	1%	-13.5	15%	-397	79%	-32.5	35%
Diarrheal diseases	788	535	-127	-125	+202	38%	+147	35%	-15.0	3%	-30.0	7%	-314	59%	-243	58%
HIV/AIDS	1210	469	-361	-382	+292	30%	+167	22%	+13.7	1%	+14.6	2%	-667	68%	-564	76%
Lower respiratory infections	869	819	-4.8	-45.1	+239	50%	+205	45%	-17.7	4%	-40.0	9%	-226	47%	-211	46%

	Total deaths				Deaths due to changes in population size				Deaths due to changes in population structure				Deaths due to changes in age-specific mortality rates			
	2000	2019	Δ 2000-2010	Δ 2010-2019	Δ 2000-2010		Δ 2010-2019		Δ 2000-2010		Δ 2010-2019		Δ 2000-2010		Δ 2010-2019	
	N	N	N	N	N	%	N	%	N	%	N	%	N	%	N	%
Malaria	819	563	-154	-103	+207	36%	+151	37%	-19.5	3%	-46.1	11%	-341	60%	-207	51%
Maternal conditions	194	151	-15.2	-27.2	+51.7	41%	+40.7	34%	+4.2	3%	+4.9	4%	-71.1	56%	-72.9	62%
Neonatal conditions	889	906	+24.1	-7.0	+248	53%	+222	49%	-42.3	9%	-104	23%	-182	38%	-125	28%
Tuberculosis	808	575	-44.8	-188	+218	45%	+166	30%	+3.0	1%	+14.8	3%	-266	55%	-370	67%
I-8	6070	4210	-987	-878	+1560	38%	+1150	36%	-80.8	2%	-199	6%	-2460	60%	-1820	58%
United States																
Childhood-cluster diseases	0.0	0.0	0.0	0.0	0.0	29%	0.0	14%	0.0	9%	0.0	13%	0.0	62%	0.0	73%
Diarrheal diseases	2.0	7.6	+8.8	-3.2	+0.6	7%	+0.8	13%	+0.5	5%	+0.7	11%	+7.7	88%	-4.6	76%
HIV/AIDS	15.2	5.2	-6.6	-3.3	+1.2	13%	+0.6	13%	-0.1	1%	0.0	0%	-7.7	86%	-3.9	87%
Lower respiratory infections	69.7	51.6	-17.2	-0.9	+6.2	15%	+4.3	26%	+6.3	15%	+3.7	22%	-29.7	70%	-8.8	53%
Malaria	0.0	0.0	0.0	0.0	0.0	-	0.0	-	0.0	-	0.0	-	0.0	-	0.0	-
Maternal conditions	0.5	0.7	+0.1	+0.2	+0.1	32%	+0.1	28%	0.0	22%	0.0	4%	+0.1	46%	+0.1	68%
Neonatal conditions	14.4	10.8	-2.0	-1.6	+1.3	29%	+1.0	28%	-0.9	20%	-1.6	47%	-2.4	52%	-0.9	25%
Tuberculosis	0.9	0.6	-0.3	-0.1	+0.1	13%	0.0	20%	+0.1	10%	+0.1	20%	-0.4	76%	-0.2	60%
I-8	103	76.6	-17.3	-8.8	+9.5	20%	+6.7	24%	+5.7	12%	+2.8	10%	-32.5	68%	-18.3	66%
Western Pacific and Southeast Asia																
Childhood-cluster diseases	90.2	17.7	-60.5	-12.1	+6.7	9%	+2.0	12%	-5.4	7%	-1.5	10%	-61.8	84%	-12.5	78%
Diarrheal diseases	250	128	-88.8	-33.4	+23.1	15%	+12.0	15%	+10.1	7%	+11.9	15%	-122	79%	-57.4	71%
HIV/AIDS	75.2	63.4	-0.9	-11.0	+8.3	37%	+5.7	24%	+2.4	11%	+0.5	2%	-11.6	52%	-17.2	73%
Lower respiratory infections	391	427	-1.4	+37.4	+43.1	22%	+33.8	19%	+53.0	27%	+73.1	41%	-97.5	50%	-69.6	39%
Malaria	12.1	4.1	-1.2	-6.8	+1.3	34%	+0.6	8%	-0.4	10%	-0.2	2%	-2.1	56%	-7.2	90%
Maternal conditions	42.2	18.4	-14.6	-9.1	+3.9	17%	+1.9	15%	-0.3	2%	-0.7	5%	-18.2	81%	-10.4	80%
Neonatal conditions	393	182	-129	-82.5	+36.5	18%	+18.5	16%	-42.8	21%	-32.6	27%	-122	61%	-68.4	57%
Tuberculosis	498	262	-113	-123	+49.8	15%	+27.3	10%	+57.5	18%	+40.6	16%	-220	67%	-191	74%
I-8	1750	1100	-409	-241	+173	19%	+102	16%	+74.0	8%	+91.2	15%	-656	73%	-434	69%

Notes:
Δ 2000-2010 = Change over the period 2000-2010
Δ 2010-2019 = Change over the period 2010-2019
% = Share of component to total component contributions (%)
N = Number of deaths, thousands

Table A12. Decomposition NCD-7 deaths into component (changes in population size, population structure, and age-specific mortality rates) contributions by region, 2000-2010 and 2010-2019.

	Total deaths				Deaths due to changes in population size				Deaths due to changes in population structure				Deaths due to changes in age-specific mortality rates			
	2000	2019	Δ 2000-2010	Δ 2010-2019	Δ 2000-2010		Δ 2010-2019		Δ 2000-2010		Δ 2010-2019		Δ 2000-2010		Δ 2010-2019	
	N	N	N	N	N	%	N	%	N	%	N	%	N	%	N	%
World																
Atherosclerotic CVD	8990	12 100	+1600	+1470	+1270	30%	+1210	26%	+1660	39%	+1870	40%	-1330	31%	-1610	34%
Diabetes	1080	2010	+392	+533	+164	42%	+184	35%	+180	46%	+258	48%	+47.7	12%	+91.0	17%
Hemorrhagic stroke	3060	3490	+261	+161	+414	28%	+365	23%	+457	31%	+503	32%	-611	41%	-708	45%
Infection-associated NCDs	2310	2580	+146	+119	+309	29%	+270	26%	+296	28%	+311	30%	-460	43%	-462	44%
Road injury	1180	1200	+66.9	-54.1	+157	47%	+131	34%	+45.1	13%	+32.2	8%	-136	40%	-217	57%
Strongly tobacco-associated NCDs	4530	5810	+526	+758	+621	28%	+580	27%	+757	34%	+874	41%	-851	38%	-696	32%
Suicide	771	736	-21.5	-13.5	+98.8	31%	+79.5	34%	+47.9	15%	+30.3	13%	-168	53%	-123	53%
NCD-7	21 900	27 900	+2970	+2970	+3030	30%	+2820	27%	+3440	34%	+3880	37%	-3510	35%	-3720	36%
Central and Eastern Europe																
Atherosclerotic CVD	2120	1770	-73.1	-280	-71.6	8%	-16.3	2%	+396	46%	+363	36%	-398	46%	-626	62%
Diabetes	39.8	84.6	+2.2	+42.5	-1.4	16%	-0.5	1%	+5.4	63%	+9.0	21%	-1.8	21%	+34.0	78%
Hemorrhagic stroke	244	151	-65.3	-28.1	-7.3	6%	-1.4	2%	+28.4	23%	+21.8	30%	-86.5	71%	-48.4	68%
Infection-associated NCDs	164	125	-19.1	-20.0	-5.3	10%	-1.1	3%	+16.6	32%	+12.9	28%	-30.4	58%	-31.8	69%
Road injury	74.6	37.3	-18.4	-18.8	-2.2	10%	-0.4	2%	+2.2	10%	-1.3	7%	-18.4	81%	-17.1	91%
Strongly tobacco-associated NCDs	324	276	-46.3	-1.6	-10.3	8%	-2.4	3%	+38.2	31%	+35.9	49%	-74.2	60%	-35.1	48%
Suicide	125	63.8	-36.3	-24.5	-3.6	8%	-0.6	3%	+5.7	12%	+0.2	1%	-38.4	80%	-24.1	96%
NCD-7	3090	2510	-256	-330	-102	8%	-22.8	2%	+493	40%	+441	36%	-647	52%	-749	62%
Central Asia																
Atherosclerotic CVD	377	497	+77.8	+42.7	+93.9	58%	+77.8	38%	+25.4	16%	+45.9	22%	-41.5	26%	-80.9	40%
Diabetes	46.6	84.6	+20.2	+17.7	+12.7	63%	+12.3	57%	+2.7	13%	+7.3	34%	+4.8	24%	-1.9	9%
Hemorrhagic stroke	104	111	+9.0	-1.7	+24.6	49%	+18.4	31%	+5.1	10%	+10.6	18%	-20.7	41%	-30.7	51%
Infection-associated NCDs	101	116	+11.3	+3.2	+24.3	50%	+18.7	36%	+5.6	12%	+8.9	17%	-18.7	38%	-24.5	47%
Road injury	33.3	48.5	+12.5	+2.7	+8.9	71%	+7.7	53%	+1.6	13%	+1.0	7%	+2.0	16%	-6.0	41%
Strongly tobacco-associated NCDs	107	130	+10.5	+11.7	+25.7	48%	+20.3	38%	+6.2	12%	+12.0	23%	-21.3	40%	-20.6	39%
Suicide	22.5	24.2	+1.1	+0.6	+5.3	42%	+3.9	50%	+1.5	12%	+0.3	4%	-5.7	46%	-3.6	46%
NCD-7	791	1010	+142	+77.0	+195	57%	+159	38%	+48.1	14%	+86.1	21%	-101	29%	-168	41%

	Total deaths				Deaths due to changes in population size				Deaths due to changes in population structure				Deaths due to changes in age-specific mortality rates			
	2000	2019	Δ 2000-2010	Δ 2010-2019	Δ 2000-2010		Δ 2010-2019		Δ 2000-2010		Δ 2010-2019		Δ 2000-2010		Δ 2010-2019	
	N	N	N	N	N	%	N	%	N	%	N	%	N	%	N	%
China																
Atherosclerotic CVD	1480	2940	+955	+510	+121	13%	+140	11%	+632	66%	+743	59%	+202	21%	-373	30%
Diabetes	184	285	+52.7	+47.9	+13.2	15%	+13.6	12%	+57.8	65%	+65.0	60%	-18.2	20%	-30.6	28%
Hemorrhagic stroke	1310	1340	+90.3	-61.5	+85.9	9%	+72.1	8%	+415	46%	+360	39%	-410	45%	-494	53%
Infection-associated NCDs	775	779	+6.6	-3.3	+49.4	10%	+40.9	9%	+204	41%	+177	40%	-247	49%	-222	50%
Road injury	279	251	+0.8	-29.1	+17.5	20%	+13.8	17%	+25.8	30%	+11.6	14%	-42.5	50%	-54.5	68%
Strongly tobacco-associated NCDs	1890	2010	+20.9	+101	+122	8%	+103	9%	+628	42%	+541	46%	-729	49%	-544	46%
Suicide	197	125	-53.7	-18.6	+10.9	8%	+7.0	10%	+30.0	22%	+17.6	26%	-94.6	70%	-43.2	64%
NCD-7	6110	7730	+1070	+547	+419	11%	+391	10%	+1990	53%	+1920	47%	-1340	36%	-1760	43%
India																
Atherosclerotic CVD	850	1870	+411	+608	+168	41%	+171	28%	+150	36%	+303	50%	+93.1	23%	+133	22%
Diabetes	142	388	+85.5	+160	+29.3	34%	+33.4	21%	+25.9	30%	+59.9	38%	+30.2	35%	+66.5	42%
Hemorrhagic stroke	273	463	+72.9	+117	+49.8	46%	+44.5	36%	+40.7	38%	+75.6	62%	-17.6	16%	-2.8	2%
Infection-associated NCDs	303	408	+59.1	+45.7	+53.6	42%	+42.6	27%	+39.2	31%	+60.0	38%	-33.6	27%	-57.0	36%
Road injury	177	212	+34.6	+0.6	+31.2	64%	+23.4	30%	+10.7	22%	+15.9	20%	-7.3	15%	-38.8	50%
Strongly tobacco-associated NCDs	600	1240	+264	+377	+117	44%	+115	31%	+108	41%	+220	58%	+39.3	15%	+41.2	11%
Suicide	169	194	+29.2	-3.6	+29.5	60%	+21.7	31%	+9.6	20%	+11.2	16%	-10.0	20%	-36.5	53%
NCD-7	2510	4770	+956	+1300	+479	50%	+452	35%	+383	40%	+747	57%	+94.2	10%	+106	8%
Latin America and Caribbean																
Atherosclerotic CVD	478	689	+99.9	+112	+65.3	21%	+55.9	19%	+138	45%	+145	50%	-104	34%	-88.8	31%
Diabetes	140	264	+69.9	+54.1	+21.3	30%	+20.9	24%	+39.2	56%	+50.0	57%	+9.3	13%	-16.8	19%
Hemorrhagic stroke	124	138	+4.9	+9.1	+15.7	20%	+11.8	18%	+26.5	33%	+26.0	39%	-37.3	47%	-28.8	43%
Infection-associated NCDs	107	130	+9.2	+13.5	+13.8	22%	+10.9	20%	+22.5	35%	+22.3	42%	-27.1	43%	-19.7	37%
Road injury	84.4	95.3	+21.6	-10.7	+11.7	54%	+8.9	24%	+5.5	26%	+4.7	12%	+4.4	20%	-24.3	64%
Strongly tobacco-associated NCDs	175	258	+42.3	+40.5	+24.2	23%	+21.0	19%	+49.2	47%	+53.8	49%	-31.0	30%	-34.3	31%
Suicide	28.0	42.8	+4.9	+9.9	+3.8	52%	+3.3	33%	+2.3	32%	+1.9	20%	-1.2	16%	+4.7	47%
NCD-7	1140	1620	+253	+228	+156	25%	+133	21%	+284	45%	+303	47%	-187	30%	-208	32%
Middle East and North Africa																
Atherosclerotic CVD	475	776	+129	+173	+109	40%	+117	41%	+91.8	34%	+113	39%	-71.8	26%	-57.4	20%

	Total deaths				Deaths due to changes in population size				Deaths due to changes in population structure				Deaths due to changes in age-specific mortality rates			
	2000	2019	Δ 2000-2010	Δ 2010-2019	Δ 2000-2010		Δ 2010-2019		Δ 2000-2010		Δ 2010-2019		Δ 2000-2010		Δ 2010-2019	
	N	N	N	N	N	%	N	%	N	%	N	%	N	%	N	%
Diabetes	50.9	119	+32.1	+35.9	+13.3	41%	+17.0	47%	+10.2	32%	+16.1	45%	+8.6	27%	+2.8	8%
Hemorrhagic stroke	75.6	84.9	-1.7	+11.0	+15.4	28%	+13.6	35%	+10.7	20%	+11.5	29%	-27.8	52%	-14.1	36%
Infection-associated NCDs	102	145	+25.7	+17.4	+23.3	42%	+23.4	33%	+17.0	31%	+20.9	29%	-14.6	27%	-26.9	38%
Road injury	71.3	88.7	+23.5	-6.1	+16.7	71%	+15.8	38%	+4.2	18%	+2.1	5%	+2.6	11%	-24.0	57%
Strongly tobacco-associated NCDs	96.0	169	+33.2	+39.8	+22.6	48%	+25.3	44%	+17.5	37%	+23.5	41%	-6.9	15%	-8.9	16%
Suicide	13.3	15.0	+0.6	+1.1	+2.8	41%	+2.5	64%	+0.9	13%	-0.1	3%	-3.0	45%	-1.2	32%
NCD-7	884	1400	+242	+272	+203	43%	+214	40%	+152	32%	+187	35%	-113	24%	-130	24%
North Atlantic																
Atherosclerotic CVD	1190	813	-245	-132	+60.9	8%	+34.8	7%	+195	26%	+152	30%	-500	66%	-319	63%
Diabetes	92.8	107	+8.8	+5.0	+5.5	16%	+4.1	11%	+15.8	47%	+16.3	46%	-12.5	37%	-15.4	43%
Hemorrhagic stroke	126	107	-10.1	-8.6	+6.8	12%	+4.4	9%	+17.1	30%	+15.8	32%	-34.1	59%	-28.8	59%
Infection-associated NCDs	126	111	-12.1	-2.1	+6.8	12%	+4.4	12%	+14.8	27%	+13.1	35%	-33.7	61%	-19.6	53%
Road injury	48.0	20.3	-22.0	-5.7	+2.1	8%	+0.9	11%	+0.2	1%	+0.3	4%	-24.3	91%	-6.9	85%
Strongly tobacco-associated NCDs	442	488	+22.8	+22.3	+25.5	17%	+18.7	13%	+60.9	41%	+62.3	45%	-63.6	42%	-58.6	42%
Suicide	53.2	52.0	-1.6	+0.3	+2.9	24%	+2.0	30%	+2.3	19%	+1.5	23%	-6.8	57%	-3.2	48%
NCD-7	2080	1700	-259	-121	+110	10%	+69.3	9%	+306	28%	+262	33%	-675	62%	-452	58%
Sub-Saharan Africa																
Atherosclerotic CVD	341	563	+100.0	+122	+106	88%	+121	72%	+4.4	4%	+24.4	14%	-10.5	9%	-23.2	14%
Diabetes	122	226	+49.7	+54.2	+39.6	80%	+47.7	79%	+0.8	2%	+9.5	16%	+9.4	19%	-3.0	5%
Hemorrhagic stroke	223	302	+35.3	+44.5	+65.6	68%	+67.8	58%	+0.2	0%	+12.7	11%	-30.6	32%	-36.0	31%
Infection-associated NCDs	186	255	+31.1	+38.4	+55.0	68%	+57.1	58%	+1.2	2%	+11.8	12%	-25.1	31%	-30.5	31%
Road injury	178	229	+19.2	+31.5	+51.5	61%	+51.6	65%	+0.5	1%	+3.6	5%	-32.8	39%	-23.7	30%
Strongly tobacco-associated NCDs	98.5	155	+24.8	+31.9	+30.2	82%	+33.5	69%	+0.7	2%	+6.7	14%	-6.0	16%	-8.3	17%
Suicide	47.5	80.3	+15.3	+17.5	+14.9	94%	+17.2	77%	+0.6	4%	+2.8	12%	-0.2	2%	-2.5	11%
NCD-7	1200	1810	+275	+340	+363	78%	+396	67%	+8.4	2%	+71.4	12%	-95.9	20%	-127	21%
United States																
Atherosclerotic CVD	736	567	-180	+11.7	+65.5	15%	+46.3	27%	+68.1	15%	+44.5	26%	-314	70%	-79.1	47%
Diabetes	71.1	90.2	-0.7	+19.8	+7.1	25%	+6.6	33%	+6.6	23%	+8.2	42%	-14.4	51%	+5.0	25%
Hemorrhagic stroke	63.2	65.2	-3.2	+5.2	+6.2	23%	+5.2	30%	+5.5	21%	+6.0	35%	-14.8	56%	-6.0	35%

	Total deaths				Deaths due to changes in population size				Deaths due to changes in population structure				Deaths due to changes in age-specific mortality rates			
	2000	2019	Δ 2000-2010	Δ 2010-2019	Δ 2000-2010		Δ 2010-2019		Δ 2000-2010		Δ 2010-2019		Δ 2000-2010		Δ 2010-2019	
	N	N	N	N	N	%	N	%	N	%	N	%	N	%	N	%
Infection-associated NCDs	43.6	55.4	+3.7	+8.1	+4.5	33%	+4.2	45%	+4.2	30%	+4.5	48%	-5.0	36%	-0.6	6%
Road injury	44.3	40.9	-8.0	+4.6	+4.0	24%	+3.2	69%	+0.5	3%	+0.4	9%	-12.6	73%	+1.0	22%
Strongly tobacco-associated NCDs	311	342	+18.5	+11.9	+32.1	31%	+27.8	22%	+29.2	28%	+41.8	33%	-42.8	41%	-57.8	45%
Suicide	31.2	50.6	+9.5	+9.9	+3.6	38%	+3.8	38%	+0.8	8%	+0.5	5%	+5.2	54%	+5.7	57%
NCD-7	1300	1210	-161	+71.1	+123	19%	+97.0	29%	+115	18%	+106	32%	-399	63%	-132	39%
Western Pacific and Southeast Asia																
Atherosclerotic CVD	919	1540	+321	+301	+119	27%	+115	22%	+261	59%	+293	57%	-59.3	14%	-107	21%
Diabetes	176	339	+70.0	+92.8	+23.1	33%	+24.0	26%	+40.2	57%	+51.3	55%	+6.7	10%	+17.4	19%
Hemorrhagic stroke	515	719	+130	+73.3	+64.0	30%	+56.5	21%	+107	50%	+115	43%	-41.0	19%	-98.5	36%
Infection-associated NCDs	391	439	+29.6	+18.1	+45.1	23%	+35.7	19%	+68.0	35%	+66.1	36%	-83.5	42%	-83.7	45%
Road injury	191	172	+4.1	-22.9	+21.4	38%	+15.2	23%	+8.4	15%	+7.0	10%	-25.7	46%	-45.1	67%
Strongly tobacco-associated NCDs	460	711	+131	+120	+58.0	27%	+53.8	22%	+113	54%	+128	53%	-40.1	19%	-61.4	25%
Suicide	81.4	84.2	+8.7	-5.8	+9.5	39%	+7.2	22%	+7.1	29%	+6.0	19%	-7.9	32%	-19.1	59%
NCD-7	2730	4000	+694	+576	+340	28%	+307	22%	+605	51%	+667	49%	-251	21%	-398	29%

Notes:
Δ 2000-2010 = Change over the period 2000-2010
Δ 2010-2019 = Change over the period 2010-2019
% = Share of component to total component contributions (%)
N = Number of deaths, thousands
